# Integrated care of severe infectious diseases to people with substance use disorders; a systematic review

**DOI:** 10.1186/s12879-019-3918-2

**Published:** 2019-04-04

**Authors:** Jørn Henrik Vold, Christer Aas, Rafael Alexander Leiva, Peter Vickerman, Fatemeh Chalabianloo, Else-Marie Løberg, Kjell Arne Johansson, Lars Thore Fadnes

**Affiliations:** 1 Department of Addiction Medicine, Haukeland University Hospital, Bergen, Norway; 2 Department of Global Public Health and Primary Care, University of Bergen, Bergen, Norway; 3 Department of Internal Medicine, Haukeland University Hospital, Bergen, Norway; 4 University of Bristol, Bristol, UK; 5 Division of Psychiatry, Haukeland University Hospital, Bergen, Norway; 6 Department of Clinical Psychology, University of Bergen, Bergen, Norway

**Keywords:** Hepatitis C, Human immunodeficiency virus, *Mycobacterium tuberculosis*, Integrated care, Collaborative care, Substance use disorder, Epidemiology, Systematic review

## Abstract

**Background:**

Various integrated care models have been used to improve treatment completion of medications for chronic hepatitis B virus (HBV), chronic hepatitis C virus (HCV), *Mycobacterium tuberculosis* (TB), and Human immunodeficiency virus (HIV) among people with substance use disorders (SUD). We have conducted a systematic review to evaluate whether integrated models have impacts of the treatment of infectious diseases among marginalized people with SUD.

**Methods:**

We searched MEDLINE/PubMed (1946 to 2018, on July 26, 2018) and Embase (from 1974 to 2018, on July 26, 2018) for randomized controlled trials (RCTs) and cohort studies evaluating diverse integrated models’ effects on sustained virological response (SVR), HIV suppression, HBV curation or suppression, completion of TB treatment regimen among people with SUD. The included studies were assessed qualitatively.

**Results:**

Altogether, 1640 studies, and references to 1135 related reviews and RCTs were considered, and only seven RCTs and three cohort studies fulfilled the inclusion criteria. We identified nine integrated care models. Two studies, one RCT and one cohort study, showed a significant effect of their integrated models. The RCT evaluated psychosocial treatment, opioid agonist treatment (OAT) and directly observed TB treatment, and found a significant increase in TB treatment completions among intervention group compared to control group (60% versus 13%, *p* < 0.01). The cohort study including OAT and TB treatments had an effect on TB treatment completion in hospitalized patients (89% versus 73%, *p* = 0.03). Eight out of ten studies showed no significant effects of their integrated care models on defined outcomes. One of which having included 363 participants in a RCT showed no effect on SVR compared to the control group when the results adjusted for active substance use and alcohol dependence in a post-hoc analysis (11% versus 7%, *p* = 0.49).

**Conclusions:**

The findings indicate uncertainty on the effects of integrated care models’ on treatment for severe infectious diseases among people with SUD. Some studies point toward that integrated models could improve care of people with SUD, yet high-quality studies and preferably, sufficiently sized clinical trials are needed to conclude on the degree of impact.

**Electronic supplementary material:**

The online version of this article (10.1186/s12879-019-3918-2) contains supplementary material, which is available to authorized users.

## Background

Comorbid Hepatitis C virus (HCV) and hepatitis B virus (HBV), human immunodeficiency virus (HIV), and *Mycobacterium tuberculosis* (TB) infections are common among people with substance use disorders (SUDs). Due to a low level of attention to personal health care needs, treatment of such severe infectious diseases may be difficult for people who inject drugs (PWID). Additionally, PWID have a sustained high risk of transmitting these severe infections [1–3]. Globally, 1.7 million PWID were estimated to be HIV positive in 2016 [4]. Injection substance use is estimated to account for 1% of new HBV infections and 23% of new HCV infections, respectively [5]. A new global strategy to eliminate viral hepatitis that aims to reduce HCV incidence by 90% and mortality by 65% by 2030 was endorsed by the World Health Assembly in 2016 [5]. The World Health Organization (WHO) and the United Nations Sustainable Development Goals (SDGs) have also decided that the global strategies are to end the acquired immune deficiency syndrome (AIDS) epidemic by 2030 and the TB epidemic by 2035 [6–8].To succeed with such ambitious goals, high availability of coordinated care between medical treatment and prevention, psychological care and social-related services for people with SUD is pivotal. A systematic search to identify efficacy of integrated care models on treatment outcomes of severe infectious diseases among people with SUD is required.

Integrated models may improve treatment completion and adherence among people who often are marginalized and hard to reach within current health services systems [2, 3, 9, 10]. Comparing the efficacy of these models is therefore of high importance. Different integrated treatment models may improve the access for completing treatment or achieving virus suppression over time but most studies are small and not sufficiently powered to quantify the effect size, and the patterns have been slightly mixed [11–15].

Definitions of the concept of “integrated care” vary. In the hospital setting, WHO defined integrated care as: “*an approach to strengthen people-centred health systems through the promotion of the comprehensive delivery of quality services across the life-course, designed according to the multidimensional needs of the population and the individual and delivered by a coordinated multidisciplinary team of providers working across settings and levels of care (…)*” [16]. However, this definition is arguably too broad and unspecific, hence, for the purpose of this paper we have chosen to alter the definition of integrated care to “comprehensive set of patient-centered health services that involve the care of chronic infectious disease as a part of coordinated services for people with SUD.” Integrate care models and collaborative health services may improve health outcomes to medical care, as well as reduce the burden of disease among people with physical and mental disorders [17], prevent severe infectious diseases [18], improve engagement in TB services [19], and increase patients adherence and staff satisfaction [11]. Systematic reviews suggest that the composition of the integrated models must be described in more detail to identify effective integrated care models that can be implemented in various settings [11, 18–20]. Previous systematic reviews have mostly identified descriptive reports or cohort studies, and only few randomized controlled trials (RCTs) [11, 17, 21]. Also, a number of these studies have methodological limitations such as short follow up period, loss-to-follow-up rate on more than 30%, unclear design and mixed outcome indicators [11, 20]. In light of the SDGs and the World Health Assembly global strategy for viral hepatitis, TB and AIDS epidemics, a systematic review is imperative to increase knowledge and understanding how to best reach universal coverage of these severe infectious diseases among people with SUD.

In this systematic review, we will examine the effect of integrated health care for people with SUD receiving treatment for HCV, HBV, HIV, and TB. Randomized controlled trials and cohort studies presenting quantitative comparative estimates will be compared to relevant outcomes for each infectious disease. We hypothesized that integrated care models might be useful to reach marginalized low adherent people with SUD, although a high risk of bias and loss-to-follow-up could underpowered the results. This systematic review will summarize the impact of integrated care models on the treatment of severe infectious diseases among people with SUD and will grade the evidence base on methodological quality.

## Methods

### Search strategy and selection criteria

We did a systematic search in MEDLINE/PubMed (1946 to 2018, on July 26, 2018) and Embase (from 1974 to 2018, on July 26, 2018) and Embase online search engines. The search was built up of three main terms; 1) infectious diseases (HCV, HBV, HIV, and TB), 2) substance use disorder and 3) integrated treatment. Sources included MeSH-terms and all components in keywords, title and abstract. The following search terms were employed; 1) infectious diseases: “hepatitis C”, OR “hepatitis B”, OR “human immunodeficiency virus”, OR “tuberculosis”, 2) substance use disorder: “substance-related disorder” OR “narcotics agent” OR “withdrawal syndrome” OR “substance abuse” OR “drug” in combination with “dependence”, “abuse”, “use”, “addict” OR “disorder” and, 3) “delivery of health care, integrated” OR “integrated health care system” (Additional files 1 and 2). The search strategy was developed in collaboration with a certified university librarian.

The three main terms were defined as follow:

1. Severe infectious diseases defined studies where the study population was subject to treatment for HCV, HIV, HBV, or TB.
2. Substance use disorder or behavior was defined as people who use illegal or legal substances, including alcohol, leading to dependency and clinically significant impairment or distress, and being in need of medical treatment in an institution or outpatient clinic for at least one SUD or the complication of the SUD.
3. Integrated treatment among marginalized people with SUD was defined as “comprehensive set of patient-centered health services that involve the care of chronic infectious disease as a part of coordinated services for people with SUD.”

WHO has recommended direct observatory therapy (DOT) for TB infected to increase treatment completion and decreased transmission rate [3]. However, for the purpose of this systematic review, and in light of the above definition of integrated care, studies employing solely DOT that was not part of an integrated care model were excluded.

In this paper, only randomized controlled trials and cohort studies were included. Cross-sectional studies, case reports, commentaries, review papers, modeling analysis, and reports without primary data were excluded, as studies lacking control groups. Studies were included for detailed assessment if sub-groups of participants met or were assumed to meet the criteria for substance use disorder. To further validate the literature search, and particularly to ensure that no papers that fulfilled the inclusion criteria were overlooked, we also did an additional search on MEDLINE/PubMed to screen for references of relevant reviews, and RCTs. To ensure that all integrated treatment models were taken into account, we did a broad search, which included MESH-terms from two of the three main terms; substance use disorder and severe infectious diseases (Additional file 1). Then, the relevant RCTs, as well as relevant references and cross-references from the reviews, were selected in line with the inclusion criteria. Studies not including patients with SUD were excluded. The proportion of participants with SUD is given in Table 1.

**Table 1:** Summery of included studies

Author/year of publication Study description	Interventions with opioid agonist treatment						Interventions without opioid agonist treatment			
	*Achmad, Y.M. et al (2009)*	*Batki, S.L. et al (2002)*	*Bruce, D.R. et al (2012)*	*Lucas, G.M. et al (2010)*	*Morozova, O. et al (2013)*	*Tetrault, J.M. et al (2012)*	*Groessl, E.J. et al (2017)*	*Ho, S.B. et al (2015)*	*Sánchez, G.C. et al (2012)*	*Simoni, J.M. et al (2007)*
*Aim*	To measure viral suppression among participants undergoing OAT and receiving ART.	To evaluate drug use counseling and directly observed methadone and isoniazid treatments affect the TB treatment completion.	To evaluate modified DOT compared to self-administered therapy (SOT) among HCV infected in OAT.	To evalute OAT and HIV drug delivered in the HIV outpatient clinic versus specialized opioid treatment program	To evaluate the effect of OAT compared to no OAT on people with substance use disorder hospitalized for TB treatment	To evaluate a psychosocial intervention's effects on HIV viral suppression among OAT patients	To measure the effect of a psychological intervention on SVR among participants undergoing HCV treatment	To measure the effect of a psychological intervention on SVR among patients undergoing HCV treatment.	To evaluate the effect of ART on HIV infected transmitted through inject drugs compared to HIV infected transmitted through intercourse.	To compare the effect of an integrated intervention to standard care among HIV infected.
*Method*	Cohort study. All participants were HIV infected. OAT is given as an intervention together with antiretrovirals in the OAT clinic. The control group receives antiretroviral without OAT.	RCT. The intervention group receives directly observed methadone and isoniazid treatments and drug use disorder counseling. Additionally, psychiatric treatment and social work referrals as needed. The intervention group is compared in two different control groups. One of them the participants receive directly observed methadone and isoniazid/pyridoxine, but they received no counseling or any other services. The second control group receives routine care including a 6-month course of isoniazid preventive therapy.	Pilot RCT. The intervention group receives HCV drugs and OAT in the OAT clinic. The control group self-administrated their HCV drugs.	RCT. Participants with opioid dependence and HIV infection are included. The intervention group receives OAT and antiretroviral drug in the HIV clinic. Participants randomized to the control group are referred to the usual opioid treatment program.	Cohort study. All participants are hospitalized and have opioid dependence are inlcuded. The intervention group receives OAT during completion of TB treatment. The control group receives TB medication without OAT.	RCT. All participants have an opioid dependence and receive OAT. The intervention group receives enhanced medical management for up to 45 minutes per session delivered by a nurse.	RCT. The intervention group receives psychological interventions which have a focus on motivating for treatment completion. The control group receives usual care in the treatment of HCV in the clinic.	RCT. The intervention group receives psychological intervention and care management provided in a multidisciplinary team. The control group receives standard care and follow treatment guidelines within the HCV clinic.	Cohort study. ART-naive and active substance users are admitted for drug dependence treatment and start antiviral medication. Those transmitted through sexual intercourse initiate antiretroviral treatment in a reference hospital. Participants receive DOT are excluded. The antiviral therapy is delivered monthly.	RCT. The intervention group receives regular follow-up meetings and weekly phone calls from peers. The control group receives social and mental health referrals when requested, but no additional adherence assistance beyond the clinic's typical is offered.
*Integrated care*	The intervention group receives OAT and antiretrovirals in the OAT clinic. The clinic offers multidisciplinary teams, counseling, HIV testing, CD4 cell count measurements and assessments for HIV. HIV assessment and treatment are integrated in the OAT clinic.	The treatment is integrated team services offering counseling, social work, and psychiatric treatment.	The OAT clinic offers a multidisciplinary team consists of medical and social workers. Participants are screened for mental illness, and test for severe infectious diseases such as HIV, HBV, and HCV. The assessments and treatments are integrated into the clinic.	The intervention considers a collaborative model where opioid dependents are assessed and treated for OAT in the HIV clinic on regular basis. A multidisciplinary team is established and has weekly meetings to discuss the individual program for the participants.	All recruited participants are hospitalized and offered integrated care of OAT and TB treatments.	Onsite treatment of opioid dependence with OAT is integrated into the HIV primary care clinic. The clinic offers integrated psychiatric services, social work services, hepatitis C treatment, and dependence treatment.	The intervention group receives psychological interventions delivered by a mental health provider before and during antiviral treatment. The mental health provider collaborates with different health professionals. Health professionals meet on a regular basis to discuss treatment progress.	The intervention group receives integrated psychological interventions and care management provided in collaboration with clinic physicians, nurses, and other mental health providers. Additionally, training, monthly conference calls, patient discussion, frequent communications, and meetings are offered.	The intervention group is followed up by a multidisciplinary team of two psychiatrists, one infectious-disease consultant, one social worker, one psychologist, and four trained nurses. The team designs, according to individual characteristics, substance use treatment, i.e., detoxification, and OAT assessment.	The intervention group receives group meetings and weekly phone calls.
*Study population and basic characteristics*	The study recruits 223 clients. Of those, 175 without OAT and 35 OAT participants. Basic characteristics between the groups are equal, except CD4 cell count. CD4 cell count for those on OAT (intervention) is 212 versus 224 for those without OAT, p = 0.05.	Of the 111 individuals who are randomized, 37 are assigned to Standard Methadone Treatment, 35 to Minimal Methadone Treatment and 39 to Routine Care. All individuals injecting drugs. The basic characteristics are significant differences in the three groups on the following variables: age (p = 0,.047), The Addiction Severity Index psychiatric composite score (p = 0.03), and Beck Depression Inventory scores (p = 0.02).	Twenty-one patients are recruited, 12 receive mDOT and nine self-administrated therapy. Differences in basic characteristics are not measured. In both groups, three participants are coinfected with HIV. Mean age of years are 40 and 43 in the intervention group and the control group, respectively. Fifty-eight percent in the intervention group and 33 % the control group are females. Nine mDOT participants and seven randomized to the control group have cocaine dependence.	Ninety-three individuals are randomized. Forty-six assigned to the intervention and 47 to referred treatment. Basic characteristics are equal between the groups, expect injection drug use and co-infection with hepatitis C.	Of the 110 participants enrolled, 57 in the intervention group and 53 in the control group. Basic charactersitics are significant differences in follow terms: site, age of years (OAT+TB: 61 %, > 36 year old, TB: 36 %, > 36 years old, p < 0.01), lifetime duration of substance use > 17 years (OAT+TB: 65 %, TB: 32 %, p < 0.01), amphetamine use 30 days prior to hospitalization (OAT+TB: 5 %, TB: 25 %, p < 0.01), < 4 TB drugs prescribed (OAT+TB: 39 %, TB: 18 %, p = 0.02), > 75 days inpatient stay at the baseline date (173 ± 170, TB: 74 ± 90, p < 0.01).	Forty-seven participants are recruited. Of those, 22 are randomized to the intervention group and 25 to the control group. Basic characteristics are equal, expect duration of HIV infection (IG:9 years (SD = 6.5), CG: 15 years (SD = 4.6), p < 0.01). Benzodiazepine addicted are excluded.	Of the 79 participants recruited, 39 are randomized to the control group and 40 to the intervention group. Basic characteristics are equal between the groups, expect age (p = 0.01).	Of the 363 participants recruited, 182 are randomized to the intervention group and 181 participants to the control group. The basic characteristics between both groups are equal, expect single status (IG: 26.1 % versus CG: 19.4 % (p = 0.045)).	Of the 119 participants, 71 active substance users and 48 participants transmitted through intercourse, are recruited. Basic characteristics are significantly different in the following variables: Origin (p = 0.01), age (p < 0.01), gender (p = 0.05), education level (p = 0.02), incarceration (p < 0.01), unemployment (p < 0.01), psychiatric disorders (p < 0.01), level of fibrosis (p < 0.01), hepatitis C coinfection (p < 0.01), and CD4 cell counts (p < 0.01). Participants with low compliance and homeless are not included.	Of the 136 participants recruited, 71 are randomized to the intervention group and 65 to the control group. No significant differences in any sociodemographic or outcome variable are found, expect satisfaction with a social report (p < 0.05). Participants who have psychosis or dementia are excluded.
*The poportion of participants with SUD*	100%	100%	100%	100%	100%	100%	In the intervention group and the control group, 48 % and 46 % report substance dependence, respectively.	In the intervention group and the control group 65 % and 67 % report substance dependence, respectively.	All included participants in the intervention group (60 %)	Lifetime heavy substance use of crack or heroin are reported in 49 % in the intervention group and 54 % in the control group. A heavy lifetime alcohol use is reported in 48 % in both groups.

*Abbreviations: ART* Antiretroviral therapy, *CG* Control group, *DOT* Direct observatory therapy, *HBV* Hepatitis B virus, *HCV* Hepatitis C virus, *HIV* Human immunodeficiency virus, *IG* Intervention group, *mDOT* Modified direct observatory therapy, *OAT* Opioid agonist therapy, *RCT* Randomized controlled trial, *SD* Standard deviation, *SUD* Substance use disorder, *SVR* Sustained virological response, *TB* Mycobactrium tuberculosis

We employed the Newcastle-Ottawa quality assessment scale (NOS) and the Consolidated Standards of Reporting Trials (CONSORT) 2010 checklist for assessing the quality of the studies. The NOS was graded in eight methodological aspects, and a summary score for selection, comparability, and outcome was ranked in low, medium or high risk of low quality [22]. The CONSORT 2010 checklist was used to assess design, conduct, analysis and interpretation, and the validity of the results [23]. Two independent authors selected included studies, and any discrepancies in the selection process were discussed within a review team of three members to achieve consensus. Duplicate references were removed.

The search was last updated on July 26, 2018. The Preferred Reporting Items for Systematic reviews and Meta-Analysis (PRISMA) criteria were followed throughout the process (Additional file 3) [24].

### Outcomes

Our outcomes were quantitative measures of treatment effect on at least one of the infectious diseases tested in an integrated care model. For HCV, sustained virological response (SVR) at least 12 weeks after the end of treatment was used as a primary outcome. For HIV care, we used HIV viral suppression, and for TB, we evaluated the completion of the recommended treatment regimen. For HBV, we planned to use clearance of HBsAg or HBeAg or suppression of HBV DNA as an indirect measure of intervention effect as outcomes.

### Data analysis

We were prevented from conducting quantitative meta-analysis due to heterogeneity among study populations, incomparability among integrated care models, few comparable studies, and different outcomes measures. Consequently, all studies were assessed qualitatively. All relevant quantitative outcomes of interest were assessed where appropriate. Odds ratio (OR) or relative risk (RR) with 95% confidence intervals, probability measures (p–values), and descriptive analyses of outcomes of interest were referred if it was stated in the articles. For comparison, we chose to categorize the included studies into two groups based on whether the participants’ received opioid agonist treatment (OAT) or not.

OAT as a multidisciplinary treatment approach has shown to improve HIV treatment and care among PWID [25], and might also serve to influence the effect of integrated care models. Unfortunately, comparability analyses between the groups were impossible due to incomparable studies, and different study populations. In addition, participants with all dependencies were included independent of groups.

## Results

**Fig. 1:**
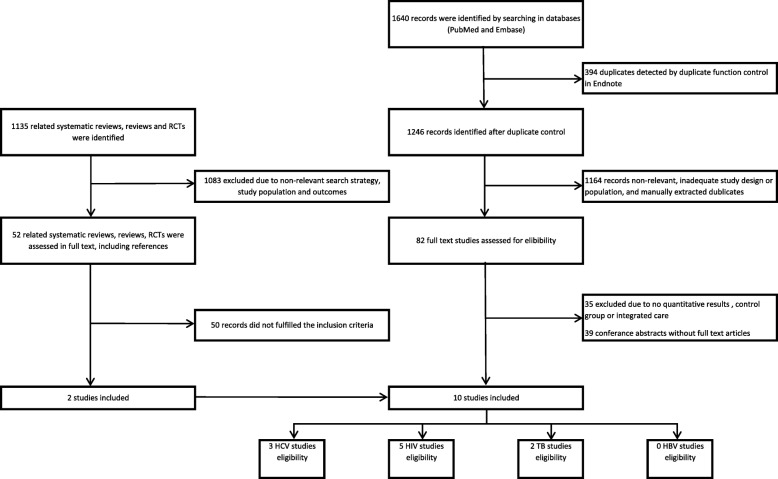
Flow diagram

Altogether, 1640 studies were identified in Embase and PubMed, and references to 1135 related reviews and RCTs were considered (Additional file 2). However, only three studies on HCV [13, 26, 27], five on HIV [14, 15, 28–30], and two on TB [31, 32] fulfilled the inclusion criteria (Fig. 1). Nine integrated models in ten studies were identified, whereas two studies assessed a similar integrated model. We did not identify any relevant studies on hepatitis B. The CONSORT 2010 checklist or the NOS were employed for assessing the quality of the included studies (Additional files 4 and 5). All included studies assessed integrated models of care when participants underwent medical treatment for HIV, HCV or TB. Nine studies recruited patients from either a primary health care centers for people with SUD, OAT outpatient clinics or specialist outpatient clinics for HIV or HCV infected [13–15, 26–30, 32]. One study evaluated TB treatment among hospitalized patients [31]. Four studies also included people without SUD [26–29]. Six out of ten studies were considered to have a loss-to-follow-up rate on more than 30% (Table 2) [13, 15, 26, 27, 29, 32].

**Table 2:** Overview of main results of integrated models

Group	Author/year of publication	Disease	Study design	Number of individuals	Loss-to-follow-up (%)	Main results
Interventions with opioid agonist treatment	Achmad, Y.M. et al (2009)	HIV	Cohort	223	No difference between the intervention group and the control group. No data are available.	Virological response (HIV viral load): IG: 97 % versus CG: 90 %, p = 0.27 Immunological response (CD4 cell count): IG: 69 % versus CG: 57 %, p = 0.93.
	Batki, S.L. et al (2002)	TB	RCT	111	52%	TBC completion (≥ 80 % of the doses administrated): 59.5 % in Standard Methadone Treatment, 77.1 % in Minimal Methadone Treatment and 13.1 % in Routine Care, p < 0.01.
	Bruce, D.R. et al (2012)	HCV	RCT	21	67%	SVR: Six of ten in the intervention group and one of four in the control group, achieved SVR
	Lucas, G.M. et al (2010)	HIV	RCT	93	17%	HIV RNA and CD4 cell count: No significant differences between opioid agonist therapy in the clinic-based buprenorphine strategy versus specialized opioid treatment program were found (p = 0.31 and p = 0.16, respectively)
	Morozova, O. et al (2013)	TB	Cohort	110	18 %	Treatment completion: IG: 90 % versus CG: 74 %, p = 0.03.
	Tetrault, J.M. et al (2012)	HIV	RCT	47	30%	Virological response (HIV viral load): Descreased from 58 % at baseline to 43 % at 12 weeks in IG, and from 56 % to 35 % in CG, p = 0.84 and p = 0.27, respectively.
Interventions without opioid agonist treatment	Groessl, E.J. et al (2017)	HCV	RCT	79	37%	SVR: 67 % in IG versus 55 % in CG, p = 0.23.
	Ho, S.B. et al (2015)	HCV	RCT	363	53%	SVR: 16 % and 8 % in IG and in CG, respectively, achieved SVR. OR 2.26 (95 % CI: 1.15-4.44, p = 0.02) SVR (active substance users and alcohol dependents): IG: 11 % versus CG: 7 %, p = 0.49.
	Sánchez, G.C. et al (2012)	HIV	Cohort	119	In IG 13 % versus in CG 8 % permanently discontinued treatment.	Virological response (HIV RNA load < 50 copies/mL) at week 48: 93 % (95 % CI = 87 % - 99 %) in IG versus 94 % (95% CI = 84 % - 100 %) in CG. Virological response (HIV RNA load < 50 copies/mL) at week 96: 87 % (95 % CI = 79 % - 95 %) in IG, and 88 % (95% CI = 78 % - 97 %) in CG. No significant differences were found, Kaplan-Meier estimates, log rank test, p =0.965.
	Simoni, J.M. et al (2007)	HIV	RCT	136	All recruited were followed up, but only 59 % at baseline, 65 % at three months, and 61 % at six months reported adherence on 95 % or more.	HIV RNA: HIV suppression was not found significantly different between the integrated group and the control group at baseline, three months and six months follow up.

*Abbreviations: CG* Control group, *HCV* Hepatitis C virus, *HIV* Human immunodeficiency virus, *IG* Intervention group, *RCT* Randomized controlled trial, *SD* Standard deviation, *SUD* Substance use disorder, *SVR* Sustained virological response, *TB* Mycobactrium tuberculosis

Different integrated care models were identified. All studies assessed integrated care in multidisciplinary teams or collaborative care teams. Three studies evaluated various psychosocial interventions integrated with treatment of an infectious disease in an OAT program delivery by DOT [13, 31, 32]. Seven studies evaluated the efficacy of brief psychological interventions, regular telephone calls, or social and mental health services in collaboration with other health professionals [14, 15, 26, 28–30, 33].

Nine integrated care models were identified, which were subsequently divided into two groups; (1) integrated care with OAT; and (2) integrated care without OAT.

### Integrated care and opioid agonist treatment

Out of the six studies on integrated care with OAT [13–15, 30–32], only two studies found an effect of the integrated care intervention [31, 32]. Hospitalized patients infected with TB receiving OAT in an integrated care setting showed a significant improvement on TB treatment completion (90% versus 74%, *p* = 0.03) and the adherence to TB medication (97% versus 86%; *p* < 0.01) after controlling for death and dropouts [31]. Additionally, Batki et al. (2002) found a significant effect on HIV suppression when integrating directly administrated OAT and antiretroviral therapy (ART) against HIV, counseling, psychiatric treatment and social works were compared to standard routine care in an outpatient clinic in people with SUD (60% versus 13%; p < 0.01) [32]. The remaining four studies showed no significant effect of integrated care on HIV suppression or achievement of HCV SVR when OAT and antiviral therapy against HIV or HCV were integrated at outpatient clinics [13–15, 30].

In the TB study that showed significant effects, integrated care was conducted among hospitalized TB infected [31]. The results indicated that OAT increased the adherence and completion to treatment in a protected environment. The intervention group was recruited from three districts among patients receiving OAT and outpatients TB treatment. Similarity, the control group was recruited from three districts where people with opioid use disorder were undergoing TB treatment, however, without OAT. Fifty-seven participants were recruited to integrated treatment compared to 53 participants in the control group. DOT was used for TB treatment, and this was integrated with OAT. Basic characteristics were entirely different between the intervention group and the control group, indicating that the study was underpowered or had biases in the randomization. There were differences in the proportion of people above 36 years of age (the intervention group: 61%, the control group: 36%, *p* < 0.01), use of amphetamine in the last 30 days (the intervention group: 5%, the control group: 25%, p < 0.01), and lifetime duration of substances used more than 17 years (the intervention group: 65%, the control group: 32%, p < 0.01).

Batki et al. (2002) conducted a randomized controlled trial showing a significant effect on preventive TB treatment completion. TB and OAT treatments were integrated into DOT models with or without psychosocial interventions with 6 months follow-up. TB treatment completion was defined as 80% or greater of all doses taken. Seventy-seven percent (*n* = 27) of participants randomized into the DOT model without psychosocial interventions achieved treatment completion compared to 60% (*n* = 22) of participants randomized into DOT model with integrated psychosocial interventions. In the control group, 14% (*n* = 5) completed the TB treatment. The two OAT treatment groups had significantly higher TB treatment completion rate compared to the control group (*p* < 0.01). However, there were no significant differences between the OAT treatment groups in the rate of TB completion. Characteristics of participants at the baseline were equal between the groups, except age (*p* = 0.047), and scores assessing the severity of problems in individuals with SUD (The Addiction Severity Index psychiatric composite score (*p* = 0.027) and the severity of depression (Beck Depression Inventory scores (*p* = 0.022)). Unfortunately, the loss-to-follow-up rate was 52%. The results may indicate a limited effect of psychosocial interventions add to directly administrated therapy to increase treatment completion of TB.

Moreover, a RCT study evaluated a clinic-based integrated treatment model in a HIV clinic [30]. All included participants received antiretroviral therapy in the clinic and had an opioid use disorder. The study evaluated the effect of assessment and initiation of OAT in the HIV clinic on HIV suppression. All opioid addicted who qualified to induct OAT were randomized. Participants randomly assigned to the control group were encouraged to be referred to the opioid treatment program. Participants randomized to the intervention delivered their OAT medications in the HIV clinic during 12 months follow-up. After 12 months, changes from baseline of HIV RNA and CD4 cell counts did not significantly differ between the groups (*p* = 0.31 and *p* = 0.16, respectively). However, the clinic-based intervention group had higher participation in OAT over 12 months of follow-up. This study enrolled only 78% of the estimated sample size, which is a limitation of the study’s validity. Also, a relatively high loss-to-follow-up rate on 17% and inclusion of only one single-center impairs the generality of the study’s results.

Two more studies evaluated integrated HIV care [14, 15]. HIV suppression and self-reported adherence to ART were evaluated in a non-randomized cohort study that compared integrated HIV treatment in an outpatient clinic among OAT patients to providing ART in an HIV outpatient clinic to patients with SUD [14]. Thirty-five patients were recruited to receive integrated care, and 175 patients recruited to the control group. The virological response was defined as HIV suppression below 400 copies/mL, and the immunological response as CD4 cell count above 200 cells/μL after at least 6 months. Among patients enrolled in OAT 97% achieved virological response compared to 90% in the control group (*p* = 0.27). Approximately 69% had an immunological response compared with 57% in the control group (*p* = 0.93). No significant differences were established among self-reported adherence and virological and immunological response between these groups. Participants in the intervention group received ART weekly and the self-reported number of medications taken the last week was reported. Basic characteristics at baseline and loss-to-follow-up were not significantly different between the groups.

Another randomized controlled trial compared an integrated intervention on HIV suppression among people with opioid use disorder to standard treatment [15, 34]. Integrated care was psychosocial intervention conducted by nurses. Twenty-two participants were randomized to integrated care and 25 participants received standard care. After the participants were included, and 2 weeks before randomization, all participants were stabilized on buprenorphine-naloxone. The outcome was changes in viral suppression 12 weeks after the intervention was initiated. After 12 weeks the number of HIV copies decreased from 58% at baseline to 43% at 12 week in the intervention group, and from 56 to 35% in the control group, *p* = 0.84 and *p* = 0.27, respectively, probably due to low sample size. The integrated psychosocial interventions were completed by a mean of 3.0 (standard deviation (SD) = 1.2) sessions per months in 3 months with a mean session length of 47 min (SD = 5.3). All participants provided consultation with a physician bi-weekly. The adherence to ART and the buprenorphine-naloxone medications was monitored by a medical event monitoring system, micro-electro-mechanical-system (MEMS® –cap). Baseline characteristics were equal in both groups, except years of HIV infection among participants (the intervention group: 8.7 (SD = 6.5), the control group: 15.4 (SD = 4.6), *p* < 0.01). The loss-to-follow-up was 30%. Some participants received the HIV medication in a different clinical site than that buprenorphine-naloxone was provided, representing a high risk of bias.

A small randomized controlled trial on HCV evaluated integrated care that included modified DOT compared to self-administrated HCV treatment among patients in OAT [13]. Twenty-one participants were recruited, 12 and nine were randomized to the intervention group and the control group, respectively. The primary outcome was SVR. The modified DOT models were organized in such a way that the hepatitis C medications and the OAT were taken under observation at the same time. Patients got an injection of peg-Interferon weekly and received the morning doses of Ribavirin together with an opioid agonist medicine. To monitor adherence, participants received a take-home evening doses in MEMS®-cap. Six out of ten and one out of four obtained SVR in the intervention group and the control group, respectively. The loss-to-follow-up was 67%, out of a small initial population. In addition to a prone to selection bias, the results were underpowered to evaluate the effect of the intervention in this study. Small sample size, selection bias, and inconclusive outcomes lead to a careful assessment of the results.

### Integrated care without opioid agonist treatment

We identified four studies with integrated care without OAT [26–29]. Substance use disorders were reported from 46 to 100% of the participants in the included studies (Table 1). Two of four studies used the terms “substance use disorder”, and “substance use”, however; the studies did not notice the type of substance dependencies [26, 27]. These two studies reported that heroin and cocaine were used in 87 and 13%, and 50 and 52% of the participants, respectively [28, 29]. One study found a significant effect of brief psychological intervention care having 29 (16%) participants in the intervention group and 14 (8%) participants in the control group that achieved SVR (OR = 2.26, 95% CI, 1.15–4.44, *p* = 0.02) on patients that were screened and that tested positive for depression, post-traumatic stress disorder (PTSD) and/or substance use disorder. A post-hoc analysis comparing people with and without SUD did not find a significant effect on SVR, having 20 (11%) in the intervention group and 14 (7%) in the control group that achieved SVR, *p* = 0.49) [27]. Integrated care seems to improve SVR, but as this study was not powered for subgroup analysis of SUD patients the results are not significant and may also have been achieved by change. In contrast, three studies failed to show any effect at all [26, 28, 29].

A RCT in a HIV primary care outpatient clinic compared an integrated care (*n* = 65) versus standard care (*n* = 71) to HIV infected [29]. The intervention consisted of six one-hour group session, twice monthly, where treatment barriers, ART adherence, and HIV stigma barriers such as a sexual and romantic relationship, substance use and mental health problems were discussed. The control group received standard care and was given social and mental health referrals. The primary outcome was suppression of HIV RNA at baseline, at 3 and 6 months. A post-hoc analysis revealed no significant differences on HIV suppression between baseline, compared to 3 months and 6 months follow up after treatment initiation. Baseline characteristics were similar in both groups. Only half of the study population had a heavy lifetime substance use of either alcohol, cocaine or heroin. Only 59% follow-up at baseline, 65% at 3 months, and 61% at 6 months, reported taking 95% or more of their prescribed medications.

Furthermore, a cohort study evaluated HIV suppression between HIV infected transmitted through substance use (intervention) or intercourse (control) followed up with blood samples at baseline, weeks 48 and 96 [28]. Active and recent people with SUD were recruited to the integrated care group where they received medical care, drug use disorder treatment, and psychosocial support. People transmitted through intercourse were received standard medical support at the infectious diseases unit of the reference hospital. The primary outcome of the study was time to loss of virological response defined as HIV RNA above 500 copies/mL after getting HIV RNA below 50 copies/mL, or not getting an HIV RNA below 50 copies/mL after week 24. Seventy-one people transmitted through substance use and 48 matched infected through sexual transmission were included. HIV suppression was measured by HIV RNA. At week 48, 93% (95% CI, 87 - 99%) of the intervention group and 94% (95% CI, 84 - 100%) of the control group achieved virus suppression, respectively. At week 96, 87% (95% CI, 79 - 95%) and 88% (95% CI, 78 - 97%) achieved virus suppression, respectively. No significant differences between substance users and people transmitted through intercourse were found at week 48 (*p* = 0.13) and week 96 (*p* = 0.24) compared to baseline Kaplan-Meier estimates (log-rank test) of time to loss of virological response and was not significantly different between the groups (*p* = 0.97). Basic characteristics were entirely different such as CD4 cell count (209 cells/μL, range, 3–427 cells/μL versus 294 cells/μL, range, 29–465 cells/μL, *p* < 0.01) and psychiatric comorbidities (personality disorder, depression, psychotic disorder, and bipolar disorder, p < 0.01). Loss-to-follow-up was indifferent in the intervention versus control groups, 13 and 8% (*p* = 0.18), respectively.

Two studies evaluated integrated versus standard HCV care in a hepatitis C medical center with HCV infected veterans with psychiatric diseases or SUD [26, 27]. The primary outcome was SVR. Participants randomized to integrated care were seen by mental health providers (one marriage and family therapist and two psychologists) who were educated in cognitive behavioral therapy (CBT) and motivational interviewing (MI). The participants received regular individual appointments, briefing before and under the antiviral treatment. The participants were contacted by the provider monthly and more frequently if they had side effects and worsening of psychiatric illness and substance use disorder during treatment.

In the first randomized HCV cohort study, 363 participants were randomized in total (*n* = 182 for integrated care and *n* = 181for standard care) [27]. Of those, 58 in the intervention group and 34 in the standard care group initiated treatment, respectively. In the intervention group, 14 participants achieved SVR (OR 2.26, 95% CI, 1.15–4.44, *p* = 0.02). To compare people with and without SUD, a post-hoc analysis on 20 (11%) in the intervention group and 14 (7%) in the control group was done. No significant effect on SVR was found between these groups, *p* = 0.49, but this post-hoc analysis was not powered for this subgroup analysis and the results could be random. Baseline characteristics were equal. Substance use disorder was detected in 66% of the participants, and 47% had active substance use. People loss-to-follow-up was 53%.

In the second randomized HCV cohort study, altogether 79 participants were recruited (*n* = 40 in the intervention group and *n* = 39 in the control group) [26]. Of those, only 18 and 9 initiated treatment, and 12 and 8 participants achieved SVR (*p* = 0.23), respectively. SVR was not significantly different between these two groups. Both studies used a combination of interferon-based treatment and direct-acting antivirals (DAA). Baseline characteristics were equal, except age (intervention group age = 54.0 (SD = 8.7) and the control group age = 57.4 (SD = 7.7), *p* = 0.01). Substance use disorder was identified in only 47% of the participants, and in 32% of those using substances actively. Loss-to-follow-up was again considerable at 59.3%.

Overall, all the included studies, with or without OAT as a part of integrated care model had a high loss-to-follow-up rate [13, 15, 26, 27, 32], unclear description of the integrated intervention [14, 31], or the primary outcome was based on self-reporting of adherence to medication and substance use [14, 15]. Five studies had a loss-to-follow rate of more than 30% [13, 15, 26, 27, 32] and for one study the quantitative rate was not given [14]. In this review, one out of four studies without OAT showed significant effects of the interventions. However, this study also included people without SUD [27]. When the results were adjusted for people with SUD or alcohol dependence in a post-hoc analysis, no significant effect on the intervention was found. In total, three out of the ten included studies used psychological interventions as a major part of the study intervention [15, 26, 27]; none of which measured a significant effect on people with SUD compared to the control groups.

## Discussion

We assessed the impact of integrated care models among patients with SUD and severe infectious diseases. Many relevant studies were excluded due to a low level of collaborative care involvement, or measurements of outcomes that were not relevant to our review. Overall, most of the included studies were considered to have a high risk of biases and both the results and methodology were heterogeneous. A recurrent finding was a high loss-to-follow-up rate, unclear description of the interventions, and underpowered studies. Moreover, differences in potential confounders between groups in cohort studies including age, proportion of homelessness, proportion of people who inject drugs, employment status, level of education, and severe psychiatric disorders to name but a few. Potential confounders may further bias the interpretation of the results.

The impact of integrated treatment among people with SUD on viral suppression, HCV SVR and TB treatment completion is uncertain according to our findings. In our review, a quantitative meta-analysis has not been performed due to limited comparable studies of high quality. Only two studies found an effect of integrated treatment. One of them found an effect of integrated OAT given to hospitalized TB-infected patients on treatment completion and medication adherence [31]. The second one found an effect on collaborative care when antivirals and OAT were given as DOT with or without psychosocial interventions [32]. Other OAT integrated models had uncertain effects. A meta-analysis published in 2016 suggested that OAT was associated with an increase in ART coverage and adherence to ART, and limited evidence for OAT decreasing mortality for PWID on ART [25]. A similar impact of OAT on adherence to hepatitis C and TB treatment has not been shown. However, Altice, FL, et al. (2011) evaluated in a multicenter study an integrated OAT model in a HIV clinical care settings, and found no association between integrated OAT and viral suppression [35]. An important limitation was the lack of a control group, which was the reason why we did not include it in our review.

The adherence to medical treatment in an integrated care model may be influenced by many other factors affecting the patient’s ability to follow up treatment as prescribed. Personal characteristics such as age, gender, mobility, lack of motivation, somatic and psychiatric comorbidities, and previous experiences, are some of the examples [36]. OAT as a part of an integrated care model may have an impact on patient’s ability and interest to follow up medical treatment. Comprehensively integrated care models, including OAT, may consist of different medical, psychological and social approaches influencing the effect on integrated care. The efficacy of these approaches is uncertain and must be explored more in detail in the future RCTs with sufficient power in order to study more precisely the effectiveness of integrated care and how it influences adherence and treatment completion.

Three studies that included psychological intervention by clinical psychologists or psychosocial intervention by nurses under clinical guidance of a clinical psychologist as a part of the integrated care were included in this literature review. The effects of psychological intervention on our outcomes are too weak to measure quantitatively. A previous systematic review has measured the effects of psychosocial interventions on people with SUD and severe mental illnesses quantitatively independently of infectious disease treatment [21]. Different psychosocial interventions were assessed, including MI, CBT, and skills training. Overall, the results were affected by low to very low rate of evidence and unclear risk of bias. Based on 32 RCTs, no compelling evidence to reduce substance use or improve mental state in patients with severe mental illnesses was found. However, a systematic review and meta-analysis on collaborative care models have shown to be useful to enhance mental and physical outcomes among patients with mental disorders [17]. Three studies included people with SUD, but no intervention was qualified to be included as collaborative care models. The main focus of the studies was integrating or coordinating primary care with an ongoing psychosocial oriented substance use treatment program. It is uncertain whether a psychological intervention that is used in the treatment of severe infectious diseases among patients with SUDs improves the viral suppression or treatment completion. Again, there is an urgent need for high-quality RCTs with a detailed description and definition of integrated treatment models effect on quantitatively outcomes measurements, and where the risk of bias is reduced.

Three of the included studies in this systematic review evaluated DOT as a tool to increase treatment completion [13, 31, 32]. Generally, DOT did not meet our definition of integrated care but was included as one of several approaches on three of the integrated care models. In two of the cases, it is uncertain how much the DOT element per se affected the results. In our review, two studies including DOT in an integrated setting showed a significant effect on TB treatment completion. DOT is a controversial method due to challenges with patient’s autonomy and could leave the patients as a passive recipient of therapy [37]. However, DOT is a part of the WHO strategy, “Directly Observed Therapy Short course,” whose proposing to increase the completion of the TB treatment, and reduce contamination [38]. DOT has been less commonly implemented in HIV, HBV, and HCV strategies due to lack of compelling evidence in support for this treatment. DOT has been evaluated in a systematic Cochrane review as a treatment modality to affect treatment completion on TB infected [12]. Interestingly, DOT was compared to self-administration of therapy and compared between PWIDs receiving TB prophylaxis as DOT to standard care. DOT compared to self-administration showed a similar pattern to completion of TB cure, but stratified analyses on people with SUD or marginalized people were not surveyed. Similarity, one study that was included in the Cochrane review compared DOT to standard care among PWIDs [39]. Three hundred PWID received TB prophylaxis delivered by DOT or no observation. Treatment completion was not significantly different between DOT compared to self-administration.

The etiological treatment for hepatitis C and HIV have improved significantly in the last decade. New DAAs against chronic hepatitis C are better tolerated and have a better efficacy than traditional interferon-based treatment, and could therefore markedly reduce the global chronic hepatitis C epidemic [5, 40]. A high cost has limited the distribution to not only low- and middle-income countries, but also high income countries such as Norway [5, 41]. From February 1, 2018, however, all chronic HCV patients in Norway have given an opportunity to receive treatment with DAAs regardless of genotype and liver fibrosis level. Among recent advances in ART against HIV are increased potency making single tablet treatment feasible, better tolerance, and improved viral suppression [42, 43]. In our review, DAAs were not tested systematically in a separate study, and stratified analysis based on this item is not surveyed. However, Norton et al. (2017) have evaluated new DAAs among people who actively use drugs and received OAT in an integrated model [44]. The results of this non-controlled study showed that substance use and OAT are independently associated to a high SVR rate (> 90, 95% CI: 42 - 99%) when DAAs are given in a coordinated service. Due to the small sample size and lack of control group, the results are uncertain and must be confirmed in high-quality studies. Further integrated care models and new antivirals may contribute to ensuring better availability and treatment completion.

The chief limitation of this review is the lack of relevant studies, which fulfilled our inclusion criteria. Thus, we were unable to conduct a meta-analysis. Heterogeneity of these studies and methodological shortcomings also made it more challenging to synthesize clear patterns of evidence. In an attempt to minimize methodological shortcomings, we opted a strict definition for inclusion. A wider definition of search strategy will include a wider range of approaches but also approaches where the health care is not substantially different from more traditional given health care. This could make it more difficult to assess meaningful effects from comparisons. Thus, trying to balance these aspects we have opted for slightly narrower definition of integrated care than Haldane et al. (2017) have used in a review focusing on HIV, substance use and service integration [11]. Nevertheless, integrated treatment, as well as substance use disorder, are frequently used terms that are used with a range of definitions. Several synonyms and terms applied in the literature, makes it challenging to create a search strategy that identify all terms and synonyms used without using extremely broad search strategies. The additional pragmatic search on references and cross-references of relevant reviews, and RCTs, including two more relevant studies, is a high proportion of studies compared to those identified in the main search. Still, we cannot rule out that some studies lack. Integrated treatment can be seen as a continuous scale from studies comparing models with limited differences from conventional strategies to the studies with pronounced differences. Choosing where to set the exact threshold of the “borderline” studies with limited differences can be difficult. However, the studies with limited differences between more conventional and integrated are unlikely to inform as much on the effects of integrated treatment as studies with more pronounced differences.

Furthermore, one could argue that RCTs and cohort studies measuring other outcomes than HIV viral suppression, HCV SVR and TB treatment completions should be included for a wider range of integrated models. On the other hand, subjective outcomes such as self-reported adherence to treatment approaches, change of substance use, and as well as self-reported change of risk behavior are substantiality uncertain outcomes, which are affected by weaknesses. These risks are potentially more pronounced among people who actively use drugs than others due to the drugs effect on cognitive functions [45]. Due to these argumentations, in this review we want to identify all high-quality studies with hard outcomes on at least one of the infectious diseases.

Finally, one could argue that studies assessing non-SUD patients groups could be included in the assessment, but that could further add to the heterogeneity, and it would be uncertain to what extent the findings could be generalized to people with SUD.

## Conclusion

This systematic review identified various integrated care models to reach and treat a group of marginalized patients with substance use disorders and severe infectious diseases. Even most of the included studies had high risks of biases and presented uncertain results. Hospitalized patients, who received integrated treatment for opioid use disorder during TB treatment, seemed to improve medication adherence and treatment completion. Additionally, integrated psychosocial interventions and directly observed OAT together with ART have been shown to improve the HIV viral suppression. For chronic HCV infected, no studies yet have shown compelling evidence of its benefits.

## Additional files

Additional file 1: Comprehensive details of the search strategy. (PDF 274 kb)

Additional file 2: Assessed articles, references and cross-references, excluded duplicates. (PDF 1228 kb)

Additional file 3: PRISMA Checklist 2009. (PDF 424 kb)

Additional file 4: CONSORT 2010. Evaluation of design, conduct, analysis, and interpretation, and the validity of the results in included RCTs. (PDF 357 kb)

Additional file 5: Newcastle-Ottawa scale. Assessment of the quality of included cohort studies. (PDF 427 kb)

## Abbreviations

AIDS: Acquired immune deficiency syndrome
AOR: Adjusted odds ratio
ART: Antiretroviral therapy
CBT: Cognitive behavioral therapy
CCM: Collaborative care model
CONSORT: Consolidated Standards of Reporting Trials
DAA: Direct-acting antivirals
DNA: Deoxyribonucleic acid
DOT: Direct observatory therapy
HBeAg: Hepatitis Be antigen
HBsAg: Hepatitis B surface antigen
HBV: Hepatitis B virus
HCV: Hepatitis C virus
HIV: Human immunodeficiency virus
MEMS®: Micro-Electro-Mechanical-System
MI: Motivational interviewing
NOS: Newcastle Ottawa score
OAT: Opioid agonist treatment
OR: Odds ratio
PRISMA: Preferred Reporting Items for Systematic reviews and Meta-Analysis
PWID: People who inject drug
RNA: Ribonucleic acid
RR: Relative risk
SUD: Substance use disorder
SVR: Sustained virological response
TB: *Mycobacterium tuberculosis*
WHO: World Health Organization
