# Supplementary material for: Integrated care of severe infectious diseases to people with substance use disorders; a systematic review
Source: BMC Infect Dis. 2019 Apr 4;19:306. doi: 10.1186/s12879-019-3918-2 (PMC6449980; doi:10.1186/s12879-019-3918-2)
Supplement: Supplementary file 1 — Comprehensive details of the search strategy. (PDF 274 kb) [file 12879_2019_3918_MOESM1_ESM.pdf]

Main search
Search engine: EMBASE
Search date: July 26, 2018

1. exp hepatitis B/ or exp hepatitis C/
2. ("hepatitis C" or "hepatitis B" or HCV or HBV or PT-NANBH or (Non adj2 hepatitis)).ti,ab,kw.
3. exp Human immunodeficiency virus/
4. (HIV or "human immunodeficiency virus" or AIDS or "aquired immune deficiency syndrome" or "Acquired Immunodeficiency Syndrome").ti,ab,kw.
5. exp tuberculosis/
6. (tuberculosis or tuberculoses or "koch* disease" or tbc).ti,ab,kw.
7. 1 or 2 or 3 or 4 or 5 or 6
8. exp narcotic agent/ or exp amphetamine derivative/ or exp anabolic agent/ or cocaine/
9. exp drug dependence/ or exp withdrawal syndrome/
10. exp drug abuse/ or exp substance abuse/
11. ((drug* or substance* or opioid or opiate or cocaine or heroin or morphine or methadone or buprenorphine or "anabolic steroid*") adj3 (abuse or use* or dependence or addict* or disorder)).ti,ab,kw.
12. 8 or 9 or 10 or 11
13. integrated health care system/
14. home care/ or home health agency/ or home monitoring/ or visiting nursing service/ or exp nursing care delivery system/ or ambulatory care/
15. (((collaborative or integrat*) adj2 (care or model* or system*)) or "shared care" or PCBH or "primary care behavio?ral health" or "mobile health units" or "ambulatory care").ti,ab,kw.
16. 13 or 14 or 15
17. 7 and 12 and 16

Search engine: MEDLINE/PubMed
Search date: July 26, 2018

1. exp Hepatitis B/
2. exp Hepatitis C/
3. ("hepatitis C" or "hepatitis B" or HCV or HBV or PT-NANBH or (Non adj2 hepatitis)).ti,ab,kw.
4. exp HIV/
5. (HIV or "human immunodeficiency virus" or AIDS or "aquired immune deficiency syndrome" or "Acquired Immunodeficiency Syndrome").ti,ab,kw.
6. exp Tuberculosis/
7. (tuberculosis or tuberculoses or "koch* disease" or tbc).ti,ab,kw.
8. 1 or 2 or 3 or 4 or 5 or 6 or 7
9. exp Narcotics/ or exp Amphetamines/ or exp Anabolic Agents/ or exp Cocaine/
10. exp Substance-Related Disorders/
11. ((drug* or substance* or opioid or opiate or cocaine or heroin or morphine or methadone or buprenorphine or "anabolic steroid*") adj3 (abuse or use* or dependence or addict* or disorder)).ti,ab,kw.
12. 9 or 10 or 11
13. exp "Delivery of Health Care, Integrated"/
14. Mobile Health Units/ or exp Patient Care Team/ or Ambulatory Care/
15. home care services/ or home care services, hospital-based/ or home health nursing/ or exp home nursing/ or homemaker services/
16. (((collaborative or integrat*) adj2 (care or model* or system*)) or "shared care" or PCBH or "primary care behavio?ral health" or "mobile health units").ti,ab,kw.
17. 13 or 14 or 15 or 16
18. 8 and 12 and 17

Additional file 1

Specific search strategy
Search engine: EMBASE
Search date: July 26, 2018

1. (“opioid" or "substance use").ti,ab,kw.
2. exp hepatitis C/
3. ("integrated" or "integrative").ti,ab,kw.
4. 1 and 2 and 3
5. exp Human immunodeficiency virus/
6. 1 and 3 and 5
7. exp tuberculosis/
8. 1 and 3 and 7
9. exp hepatitis B/
10. 1 and 3 and 9

Search engine: MEDLINE
Search date: July 26, 2018

1. ("Opioid" or "substance use").ti,ab,kw.
2. exp Hepatitis C/
3. ("integrated" or "integrative").ti,ab,kw.
4. 1 and 2 and 3
5. exp HIV/
6. 1 and 3 and 5
7. exp TUBERCULOSIS/
8. 1 and 3 and 7
9. exp Hepatitis B/
10. 1 and 3 and 9

Search to screen for references of relevant reviews and RCTs
Search engine: MEDLINE
"Substance-Related Disorders"[Mesh] AND ("HIV"[Mesh] OR "Hepatitis C"[Mesh] OR "Tuberculosis"[Mesh] OR "Hepatitis B"[Mesh]) AND (systematic[sb] OR Review[ptyp] OR Meta-Analysis[ptyp] OR Controlled Clinical Trial[ptyp])
