# Supplementary material for: Integrated care of severe infectious diseases to people with substance use disorders; a systematic review
Source: BMC Infect Dis. 2019 Apr 4;19:306. doi: 10.1186/s12879-019-3918-2 (PMC6449980; doi:10.1186/s12879-019-3918-2)
Supplement: Supplementary file 2 — Assessed articles, references and cross-references, excluded duplicates. (PDF 1228 kb) [file 12879_2019_3918_MOESM2_ESM.pdf]

Additional file 2

Search strategy:

1. Integrated prevention services for HIV infection, viral hepatitis, sexually transmitted diseases, and tuberculosis for persons who use drugs illicitly: summary guidance from CDC and the U.S. Department of Health and Human Services. MMWR Recomm Rep 2012, 61(Rr-5):1-40.

2. Aaron E, Alvare T, Gracely EJ, Riviello R, Althoff A: Predictors of Linkage to Care for Newly Diagnosed HIV-Positive Adults. West J Emerg Med 2015, 16(4):535-542.

3. Abbaticola MM: A team approach to the treatment of AIDS wasting. Journal of the Association of Nurses in AIDS Care 2000, 11(1):45-56.

4. Abd Rashid RA, Merican MN, Sulaiman AH, Habil H: Assissted medication therapy integrated with therapeutic community: The way forward. International Journal of Neuropsychopharmacology 2010, 1):47.

5. Abeles H: Treatment of alcoholic, tuberculous patient. New York State Journal of Medicine 1974, 74(9):1593-1596.

6. Abidjanova N: Palliative Care Development in Tajikistan. Journal of Pain and Symptom Management 2018, 55(2 Supplement):S81-S84.

7. Achmad YM, Istiqomah AN, Iskandar S, Wisaksana R, van Crevel R, Hidayat T: Integration of methadone maintenance treatment and HIV care for injecting drug users: a cohort study in Bandung, Indonesia. Acta medica Indonesiana 2009, 41 Suppl 1:23-27.

8. Adams JL, Gaynes BN, McGuinness T, Modi R, Willig J, Pence BW: Treating depression within the HIV medical home: A guided algorithm for antidepressant management by HIV clinicians. AIDS Patient Care and STDs 2012, 26(11):647-654.

9. Adelsheim S: From school health to integrated health: expanding our children's public mental health system. Academic psychiatry : the journal of the American Association of Directors of Psychiatric Residency Training and the Association for Academic Psychiatry 2014, 38(4):405-408.

10. Ahlberg-Hulten G, Theorell T: Combined somatic and psychiatric care--effects on personnel treating drug addicts with infectious diseases. J Occup Health Psychol 1997, 2(3):263-272.

11. Alavi M, Grebely J, Micallef M, Dunlop AJ, Balcomb AC, Day CA, Treloar C, Bath N, Haber PS, Dore GJ: Assessment and treatment of hepatitis c virus infection among people who inject drugs in the opioid substitution setting: ETHOS study. Clinical Infectious Diseases 2013, 57(SUPPL.2):S62-S69.

12. Al-Darraji HA, Wong KC, Yeow DG, Fu JJ, Loeliger K, Paiji C, Kamarulzaman A, Altice FL: Tuberculosis screening in a novel substance abuse treatment center in Malaysia: implications for a comprehensive approach for integrated care. Journal of Substance Abuse Treatment 2014, 46(2):144-149.

13. Al-Darraji HAA, Wong KC, Yeow DGE, Fu JJ, Loeliger K, Paiji C, Kamarulzaman A, Altice FL: Tuberculosis screening in a novel substance abuse treatment center in Malaysia: Implications for a comprehensive approach for integrated care. Journal of Substance Abuse Treatment 2014, 46(2):144-149.

14. Alessi SS, Sanches JA, de Oliveira WR, Messina MC, Pimentel RA, Neto CF: Treatment of cutaneous tumors with topical 5% imiquimod cream. Clinics 2009, 64(10):961-966.

15. Aletraris L, Roman PM, Pruett J: Integration of Care in the Implementation of the Affordable Care Act: Changes in Treatment Services in a National Sample of Centers Treating Substance Use Disorders. Journal of Psychoactive Drugs 2017, 49(2):132-140.

16. Ali Z: Kenya Hospices and Palliative Care Association: Integrating palliative care in public hospitals in Kenya. ecancermedicalscience 2016, 10 (no pagination)(655).

17. Alim T, Richardson F, Settles-Reaves B, Kumari S, Akinfiresoye E, Chapman E, Bland W, Johnson M: Buprenorphine integrated care delivery project: Correlates of mental health screening and primary care outcomes. Neuropsychopharmacology 2017, 43 (Supplement 1):S256-S257.

18. Allemann SS, Bornand D, Hug B, Hersberger KE, Arnet I: Issues around the Prescription of Half Tablets in Northern Switzerland: The Irrational Case of Quetiapine. BioMed Research International 2015, 2015 (no pagination)(602021).

19. Allen SM, Fleishman J: Problems encountered in home health service delivery to persons with AIDS. Home health care services quarterly 1992, 13(1-2):129-159.

20. Altice FL, Azbel L, Stone J, Brooks-Pollock E, Smyrnov P, Dvoriak S, Taxman FS, El-Bassel N, Martin NK, Booth R et al: The perfect storm: incarceration and the high-risk environment perpetuating transmission of HIV, hepatitis C virus, and tuberculosis in Eastern Europe and Central Asia. Lancet 2016, 388(10050):1228-1248.

21. Altice FL, Azbel L, Stone J, Brooks-Pollock E, Smyrnov P, Dvoriak S, Taxman FS, El-Bassel N, Martin NK, Booth R et al: The perfect storm: incarceration and the high-risk environment perpetuating transmission of HIV, hepatitis C virus, and tuberculosis in Eastern Europe and Central Asia. Lancet 2016, 388(10050):1228-1248.

22. Altice FL, Bruce RD, Walton MR, Buitrago MI: Adherence to hepatitis B virus vaccination at syringe exchange sites. Journal of Urban Health 2005, 82(1):151-161.

23. Altice FL, Kamarulzaman A, Soriano VV, Schechter M, Friedland GH: Treatment of medical, psychiatric, and substance-use comorbidities in people infected with HIV who use drugs. Lancet 2010, 376(9738):367-387.

24. Altice FL, Kamarulzaman A, Soriano VV, Schechter M, Friedland GH: Treatment of medical, psychiatric, and substance-use comorbidities in people infected with HIV who use drugs. Lancet 2010, 376(9738):367-387.

25. Altice FL, Sullivan LE, Smith-Rohrberg D, Basu S, Stancliff S, Eldred L: The potential role of buprenorphine in the treatment of opioid dependence in HIV-infected individuals and in HIV infection prevention. Clinical Infectious Diseases 2006, 43(SUPPL. 4):S178-S183.

26. Ambia J, Renju J, Wringe A, Todd J, Geubbels E, Nakiyingi-Miiro J, Urassa M, Lutalo T, Crampin AC, Kwaro D et al: From policy to practice: exploring the implementation of antiretroviral therapy access and retention policies between 2013 and 2016 in six sub-Saharan African countries. BMC health services research 2017, 17(1):758.

27. Anand T, Nitpolprasert C, Phanuphak N: Online-to-offline models in HIV service delivery. Current Opinion in HIV and AIDS 2017, 12(5):447-457.

28. Anassi EO, Egbunike IG, Akpaffiong MJ, Ike EN, Cate TR: Developing and implementing guidelines to promote appropriate use of fluconazole therapy in an AIDS clinic. Hospital Pharmacy 1994, 29(6):576-578, 581-572, 585-576.

29. Andersen M, Paliwoda J, Kaczynski R, Schoener E, Harris C, Madeja C, Reid H, Weber C, Trent C: Integrating Medical and Substance Abuse Treatment for Addicts Living with HIV/AIDS: Evidence-Based Nursing Practice Model. American Journal of Drug and Alcohol Abuse 2003, 29(4):847-859.

30. Andersen M, Smereck GA, Hockman E, Tinsley J, Milfort D, Shekoski C, Connelly C, Faber-Bermudez I, Schuman P, Emrich K et al: Integrating health care for women diagnosed with HIV infection, substance abuse, and mental illness in Detroit, Michigan. The Journal of the Association of Nurses in AIDS Care : JANAC 2003, 14(5):49-58.

31. Andersen MD, Smereck GA, Hockman EM, Ross DJ, Ground KJ: Nurses decrease barriers to health care by "hyperlinking" multiple-diagnosed women living with HIV/AIDS into care. The Journal of the Association of Nurses in AIDS Care : JANAC 1999, 10(2):55-65.

32. Anderson JE, Cheney R, Clatts M, Faruque S, Kipke M, Long A, Mills S, Toomey K, Wiebel W: HIV risk behavior, street outreach, and condom use in eight high-risk populations. AIDS Educ Prev 1996, 8(3):191-204.

33. Andreu A, Sala F, Nieto X, Gloria C, Vilaro L, Delicado S, Segui C, Molto J, Bonafont X: Knowledge of polypharmacy in HIV-Infected patients in a Spanish university hospital. International Journal of Clinical Pharmacy 2015, 37 (1):193.

34. Andrews CM, Shin HC, Marsh JC, Cao D: Client and program characteristics associated with wait time to substance abuse treatment entry. Am J Drug Alcohol Abuse 2013, 39(1):61-68.

35. Ang DC, Calabrese LH: A common-sense approach to chronic fatigue in primary care. Cleveland Clinic journal of medicine 1999, 66(6):343-350, 352.

36. Anonymous: A survey on distribution of intractable tuberculosis patients and an analysis. [Korean]. Tuberculosis and Respiratory Diseases 1980, 27(1):1-8.

37. Anonymous: Drug injectors in Glasgow: a community at risk? A report from a multidisciplinary group. The Possilpark Group. Health Bull (Edinb) 1993, 51(6):418-429.

38. Anonymous: [Substance dependent patients are rarely in treatment. Step-children in HIV medicine]. MMW Fortschr Med 2001, 143 Suppl 1:102.

39. Anonymous: Breastfeeding and the use of human milk. Pediatrics 2005, 115(2):496-506.

40. Anonymous: Society for Adolescent Medicine Annual Meeting - Adolescent Clinical Care: Integrating Art and Science. Journal of Adolescent Health Conference: Society for Adolescent Medicine Annual Meeting Adolescent Clinical Care: Integrating Art and Science Toronto, ON Canada Conference Publication: 2010, 46(2 SUPPL. 1).

41. Anonymous: HIV clinic gets ART to toughest cases. Patients with 10-plus meds targeted. Aids Alert 2010, 25(5):51-53.

42. Anonymous: 2013 CAEP/ACMU Scientific Abstracts, CAEP 2013. Canadian Journal of Emergency Medicine 2013, 15:S1.

43. Anonymous: ESCP International Workshop Extended Responsibilities for Pharmacists in the Treatment of Acute and Chronic Conditions. International Journal of Clinical Pharmacy Conference 2017, 39(4).

44. Anonymous: National Kidney Foundation 2018 Spring Clinical Meeting Abstracts April 10-14, 2018. American Journal of Kidney Diseases 2018, 71(4):502-515.

45. Appenheimer AB, Bokhour B, McInnes DK, Richardson KK, Thurman AL, Beck BF, Vaughan-Sarrazin M, Asch SM, Midboe AM, Taylor T et al: Should human immunodeficiency virus specialty clinics treat patients with hypertension or refer to primary care? An analysis of treatment outcomes. Open Forum Infectious Diseases 2017, 4 (1) (no pagination)(ofx005).

46. Arain A, Robaeys G: Eligibility of persons who inject drugs for treatment of hepatitis C virus infection. World J Gastroenterol 2014, 20(36):12722-12733.

47. Arain A, Robaeys G: Eligibility of persons who inject drugs for treatment of hepatitis C virus infection. World J Gastroenterol 2014, 20(36):12722-12733.

48. Artenie AA, Bruneau J, Zang G, Bamvita JM, Levesque A, Jutras-Aswad D, Daniel M, Roy E: Visiting a primary care physician among persons who inject drugs attending syringe exchange programs: Coupling strategies to optimize HCV prevention. Journal of Hepatology 2014, 1):S515.

49. Artenie AA, Roy E, Zang G, Jutras-Aswad D, Bamvita JM, Puzhko S, Daniel M, Bruneau J: Hepatitis C Virus seroconversion among persons who inject drugs in relation to primary care physician visiting: The potential role of primary healthcare in a combined approach to Hepatitis C prevention. International Journal of Drug Policy 2015, 26(10):970-975.

50. Ashman JJ, Conviser R, Pounds MB: Associations between HIV-positive individuals' receipt of ancillary services and medical care receipt and retention. AIDS Care 2002, 14 Suppl 1:S109-118.

51. Aspinall EJ, Corson S, Doyle JS, Grebely J, Hutchinson SJ, Dore GJ, Goldberg DJ, Hellard ME: Treatment of hepatitis C virus infection among people who are actively injecting drugs: a systematic review and meta-analysis. Clin Infect Dis 2013, 57 Suppl 2:S80-89.

52. Aspinall EJ, Corson S, Doyle JS, Grebely J, Hutchinson SJ, Dore GJ, Goldberg DJ, Hellard ME: Treatment of hepatitis C virus infection among people who are actively injecting drugs: a systematic review and meta-analysis. Clin Infect Dis 2013, 57 Suppl 2:S80-89.

53. Astone JM, Strauss SM, Hagan H, Des Jarlais DC: Outpatient drug treatment program directors' hepatitis C-related beliefs and their relationship to the provision of HCV services. Am J Drug Alcohol Abuse 2004, 30(4):783-797.

54. Austin MP, Colton J, Priest S, Reilly N, Hadzi-Pavlovic D: The antenatal risk questionnaire (ANRQ): Acceptability and use for psychosocial risk assessment in the maternity setting. Women and Birth 2013, 26(1):17-25.

55. Avins AL, Lindan CP, Woods WJ, Hudes ES, Boscarino JA, Kay J, Clark W, Hulley SB: Changes in HIV-related behaviors among heterosexual alcoholics following addiction treatment. Drug Alcohol Depend 1997, 44(1):47-55.

56. Azad N, Rojanasakul Y: Nanobiotechnology in drug delivery. American Journal of Drug Delivery 2006, 4(2):79-88.

57. Azar MM, Springer SA, Meyer JP, Altice FL: A systematic review of the impact of alcohol use disorders on HIV treatment outcomes, adherence to antiretroviral therapy and health care utilization. Drug Alcohol Depend 2010, 112(3):178-193.

58. Azar MM, Springer SA, Meyer JP, Altice FL: A systematic review of the impact of alcohol use disorders on HIV treatment outcomes, adherence to antiretroviral therapy and health care utilization. Drug Alcohol Depend 2010, 112(3):178-193.

59. Azzopardi C, Salter R, Wade M, MacDougall GM, Read S, Bitnun A: Children with HIV: Intersection with the child welfare system for medical non-adherence. Canadian Journal of Infectious Diseases and Medical Microbiology 2013, 24:132A-133A.

60. Bachireddy C, Soule MC, Izenberg JM, Dvoryak S, Dumchev K, Altice FL: Integration of health services improves multiple healthcare outcomes among HIV-infected people who inject drugs in Ukraine. Drug and Alcohol Dependence 2014, 134(1):106-114.

61. Backmund M, Eichenlaub D, Soyka M: [The "Qualified Detoxification Treatment of Drug Dependent Patients" Federal Model Project in a general hospital: concept, investments and clinical results]. Gesundheitswesen 1998, 60(10):552-557.

62. Backmund M, Reimer J, Meyer K, Gerlach JT, Zachoval R: Hepatitis C virus infection and injection drug users: prevention, risk factors, and treatment. Clin Infect Dis 2005, 40 Suppl 5:S330-335.

63. Backmund M, Reimer J, Meyer K, Gerlach JT, Zachoval R: Hepatitis C virus infection and injection drug users: prevention, risk factors, and treatment. Clin Infect Dis 2005, 40 Suppl 5:S330-335.

64. Backus LI, Boothroyd D, Deyton LR: HIV, hepatitis C and HIV/hepatitis C virus co-infection in vulnerable populations. Aids 2005, 19(SUPPL. 3):S13-S19.

65. Bahorik AL, Satre DD, Kline-Simon AH, Weisner CM, Campbell CI: Alcohol, Cannabis, and Opioid Use Disorders, and Disease Burden in an Integrated Health Care System. J Addict Med 2017, 11(1):3-9.

66. Bajis S, Dore GJ, Hajarizadeh B, Cunningham EB, Maher L, Grebely J: Interventions to enhance testing, linkage to care and treatment uptake for hepatitis C virus infection among people who inject drugs: A systematic review. International Journal of Drug Policy 2017, 47:34-46.

67. Bajis S, Dore GJ, Hajarizadeh B, Cunningham EB, Maher L, Grebely J: Interventions to enhance testing, linkage to care and treatment uptake for hepatitis C virus infection among people who inject drugs: A systematic review. The International journal on drug policy 2017, 47:34-46.

68. Bajis S, Dore GJ, Hajarizadeh B, Cunningham EB, Maher L, Grebely J: Interventions to enhance testing, linkage to care and treatment uptake for hepatitis C virus infection among people who inject drugs: A systematic review. The International journal on drug policy 2017, 47:34-46.

69. Balinsky W: Pediatric home care: reimbursement and cost benefit analysis. Journal of pediatric health care : official publication of National Association of Pediatric Nurse Associates & Practitioners 1999, 13(6 Pt 1):288-294.

70. Ball K, Parrish R, Thornton A: An assessment of the effects of ancillary services on CD4 counts and viral loads of people living with HIV/AIDS. Sexually Transmitted Infections 2011, 1):A289.

71. Baltzer Turje R, Payne HM, McDougall P, Davis M: Developing best practices for clinical policies and procedures for supervised injection services within integrated health care settings in Canada. Canadian Journal of Infectious Diseases and Medical Microbiology 2015, 26:21B.

72. Barcellos NT, Fuchs SC, Fuchs FD: Prevalence of and risk factors for HIV infection in individuals testing for HIV at counseling centers in Brazil. Sex Transm Dis 2003, 30(2):166-173.

73. Bari K, Augustin S, Eggers CA, Hashem HJ, Desai M, Shea M, Wongcharatrawee S, Lim JK, Garcia-Tsao G: Hepatitis c (HCV) antiviral therapy is feasible in patients with active substance use (SU) and mental illness (MI) and is more likely to occur in those who receive education class. Gastroenterology 2012, 1):S967.

74. Barry M, Fleck E, Lentz S, Bell C, O'Connor P, Horwitz R: "Medicine on wheels": an opportunity for outreach and housestaff education. Connecticut medicine 1994, 58(9):535-539.

75. Bass M, Linsk N: Stigma's relationship to substance abuse counselors' HIV medication adherence counseling intention. Journal of the International Association of Physicians in AIDS Care 2010, 9 (1):61-62.

76. Basu S, Smith-Rohrberg D, Bruce RD, Altice FL: Models for integrating buprenorphine therapy into the primary HIV care setting. Clinical Infectious Diseases 2006, 42(5):716-721.

77. Bataller R, Stein E, Altamirano JT, Cruz-Lemini M, Cheong J: A worldwide study reveals that the amount of daily alcohol intake is a better predictor of the weight of alcohol in the cirrhosis burden than the total per capita comsumption. Journal of Hepatology 2015, 62:S256-S257.

78. Batchelder AW, Peyser D, Nahvi S, Arnsten JH, Litwin AH: "Hepatitis C treatment turned me around:" Psychological and behavioral transformation related to hepatitis C treatment. Drug and Alcohol Dependence 2015, 153:66-71.

79. Batisse A, Batel P, Chevallier C, Marillier M, Djezzar S: "Sex and drugs" in substance-using men who have sex with men (SUMSM). European Psychiatry 2015, 1):491.

80. Batki SL: Drug abuse, psychiatric disorders, and AIDS. Dual and triple diagnosis. West J Med 1990, 152(5):547-552.

81. Batki SL, Ferrando SJ: Diagnosis and treatment of substance use disorders in patients with HIV infection. International Review of Psychiatry 1996, 8(2-3):245-252.

82. Batki SL, Gruber VA, Bradley JM, Bradley M, Delucchi K: A controlled trial of methadone treatment combined with directly observed isoniazid for tuberculosis prevention in injection drug users. Drug Alcohol Depend 2002, 66(3):283-293.

83. Batki SL, Gruber VA, Bradley JM, Bradley M, Delucchi K: A controlled trial of methadone treatment combined with directly observed isoniazid for tuberculosis prevention in injection drug users. Drug Alcohol Depend 2002, 66(3):283-293.

84. Batkis MF, Treisman GJ, Angelino AF: Integrated opioid use disorder and HIV treatment: Rationale, clinical guidelines for addiction treatment, and review of interactions of antiretroviral agents and opioid agonist therapies. AIDS Patient Care and STDs 2010, 24(1):15-22.

85. Baudry A: Comparative study of the results of 3 different regimens of antitubercular drug therapy used in rural Zaire during the last 15 years. [French]. Annales de la Societe belge de medecine tropicale 1973, 53(2):67-75.

86. Baudry A: [Comparative study of the results of 3 different regimens of antitubercular drug therapy used in rural Zaire during the last 15 years]. Annales de la Societe Belge de Medecine Tropicale 1973, 53(2):67-75.

87. Baumann S, Neff C, Fetzick S, Stangl G, Basler L, Vereneck R, Schneider W: A virtual reality system for neurobehavioral and functional MRI studies. Cyberpsychology and Behavior 2003, 6(3):259-266.

88. Bayer R: AIDS and the ethics of public health: challenges posed by a maturing epidemic. AIDS 1988, 2 Suppl 1:S217-221.

89. Behrends K: [Integration of the public health service in community management of substance dependent patients]. Offentl Gesundheitswes 1991, 53(8-9):543-545.

90. Bekker LG, Beyrer C, Quinn TC: Behavioral and biomedical combination strategies for HIV prevention. Cold Spring Harbor Perspectives in Medicine 2012, 2 (8) (no pagination)(a007435).

91. Belfiori B, Ciliegi P, Chiodera A, Bacosi D, Tosti A, Baldelli F, Francisci D: Peginterferon plus Ribavirin for chronic hepatitis C in opiate addicts on methadone/buprenorphine maintenance therapy. Digestive and Liver Disease 2009, 41(4):303-307.

92. Bellingham C: Enhanced services: What is on offer? Pharmaceutical Journal 2005, 275(7367):341-342.

93. Belperio PS, Chartier M, Ross DB, Alaigh P, Shulkin D: Curing Hepatitis C virus infection: Best practices from the U.S. Department of Veterans Affairs. Annals of Internal Medicine 2017, 167(7):499-504.

94. Benjamin-Johnson R, Nakazono T, Moore A, Zingmond D, Cunningham W: Impact of behavioral health treatment on aids outcomes among californiamedicaid beneficiaries. Journal of General Internal Medicine 2010, 25:S302.

95. Bennett CL, Pascal A, Cvitanic M, Graham V, Kitchens A, DeHovitz JA: Medical care costs of intravenous drug users with AIDS in Brooklyn. Journal of Acquired Immune Deficiency Syndromes 1992, 5(1):1-6.

96. Berg KM, Litwin AH, Li X, Heo M, Arnsten JH: Lack of sustained improvement in adherence or viral load following a directly observed antiretroviral therapy intervention. Clin Infect Dis 2011, 53(9):936-943.

97. Berg KM, Litwin AH, Li X, Heo M, Arnsten JH: Lack of sustained improvement in adherence or viral load following a directly observed antiretroviral therapy intervention. Clin Infect Dis 2011, 53(9):936-943.

98. Beyrer C, Baral S, Kerrigan D, El-Bassel N, Bekker LG, Celentano DD: Expanding the space: Inclusion of most-at-risk populations in HIV prevention, treatment, and care services. Journal of Acquired Immune Deficiency Syndromes 2011, 57(SUPPL. 2):S96-S99.

99. Bien MB: Art therapy as emotional and spiritual medicine for Native Americans living with HIV/AIDS. Journal of Psychoactive Drugs 2005, 37(3):281-292.

100. Bilal U, Lau B, Hutton HE, Mathews WC, Mugavero M, Cropsey K, McCaul ME, Crane H, Chander G: Alcohol use trajectories among persons living with HIV in care in the United States. Alcoholism: Clinical and Experimental Research 2016, 40:48A.

101. Bilal U, McCaul ME, Crane HM, Mathews WC, Mayer KH, Geng E, Napravnik S, Cropsey KL, Mugavero MJ, Saag MS et al: Predictors of Longitudinal Trajectories of Alcohol Consumption in People with HIV. Alcoholism: Clinical and Experimental Research 2018, 42(3):561-570.

102. Bird K, Socias ME, Ti L: Integrating hepatitis C and addiction care for people who inject drugs in the era of direct-acting antiviral therapy. International Journal of Drug Policy 2018, 59:1-2.

103. Birkhead GS, Klein SJ, Candelas AR, O'Connell DA, Rothman JR, Feldman IS, Tsui DS, Cotroneo RA, Flanigan CA: Integrating multiple programme and policy approaches to hepatitis C prevention and care for injection drug users: A comprehensive approach. International Journal of Drug Policy 2007, 18(5):417-425.

104. Birkhead GS, Klein SJ, Candelas AR, O'Connell DA, Rothman JR, Feldman IS, Tsui DS, Cotroneo RA, Flanigan CA: Integrating multiple programme and policy approaches to hepatitis C prevention and care for injection drug users: a comprehensive approach. The International journal on drug policy 2007, 18(5):417-425.

105. Birkhead GS, Klein SJ, Candelas AR, O'Connell DA, Rothman JR, Feldman IS, Tsui DS, Cotroneo RA, Flanigan CA: Integrating multiple programme and policy approaches to hepatitis C prevention and care for injection drug users: a comprehensive approach. The International journal on drug policy 2007, 18(5):417-425.

106. Black MM, Nair P, Harrington D: Maternal HIV infection: Parenting and early child development. Journal of Pediatric Psychology 1994, 19(5):595-616.

107. Black MM, Nair P, Kight C, Wachtel R, Roby P, Schuler M: Parenting and early development among children of drug-abusing women: effects of home intervention. Pediatrics 1994, 94(4 Pt 1):440-448.

108. Blackstock OJ, King JR, Mason RD, Lee CC, Mannheimer SB: Evaluation of a rapid HIV testing initiative in an urban, hospital-based dental clinic. AIDS Patient Care STDS 2010, 24(12):781-785.

109. Blair JM, Fagan JL, Frazier EL, Do A, Bradley H, Valverde EE, McNaghten A, Beer L, Zhang S, Huang P et al: Behavioral and clinical characteristics of persons receiving medical care for HIV infection - Medical Monitoring Project, United States, 2009. Morbidity and mortality weekly report 2014, Surveillance summaries (Washington, D.C. : 2002). 63 Suppl 5:1-22.

110. Blair JM, Fagan JL, Frazier EL, Do A, Bradley H, Valverde EE, McNaghten A, Beer L, Zhang S, Huang P et al: Behavioral and clinical characteristics of persons receiving medical care for HIV infection - Medical Monitoring Project, United States, 2009. MMWR (Suppl) 2014, 63(5):1-22.

111. Boadas A, Mi Jares M, Hernandez L, Ramirez G, Ruiz-Saez A: Causes of death in patients with hemophilia attended at the national hemophilia center of venezuela (1989-2011). Haemophilia 2012, 18:15.

112. Bobchenok AP: Improving ambulatory services for children in the foci of tuberculosis with special reference to the factors of increased risk of infection. [Russian]. Problemy tuberkuleza 1991(8):23-25.

113. Bobchenok AP: [Improving ambulatory services for children in the foci of tuberculosis with special reference to the factors of increased risk of infection]. Problemy Tuberkuleza 1991(8):23-25.

114. Bodrozic JN, Miljic PS, Lekovic DR, Petronijevic MA, Antic DA, Mitrovic MM, Petronijevic-Vrzic SM, Djunic IS: Pregnancy and delivery in a woman with severe haemophilia A. Blood Coagulation and Fibrinolysis 2017, 28(6):496-499.

115. Boissonnas A: [How to treat a patient with AIDS and drug dependence unwilling and/or unable to withdraw? Give substitution drugs at the hospital? Should substitution be continued after

discharge? Is substitution a useful adjuvant to programs designed to prevent opportunistic diseases?] opportunisticinfections?]. Annales de Medecine Interne 1994, 145 Suppl 3:54-55.

116. Bokhari SM, Yao H, Bethel-Brown C, Fuwang P, Williams R, Dhillon NK, Hegde R, Kumar A, Buch SJ: Morphine enhances Tat-induced activation in murine microglia. Journal of neurovirology 2009, 15(3):219-228.

117. Bonafede M, Cappell K, Sapra S, Shah N, Desai P, Tepper S: Direct costs associated with migraine in the us. Neurology Conference: 69th American Academy of Neurology Annual Meeting, AAN 2017, 88(16 Supplement 1).

118. Bonkovsky HL, Tice AD, Yapp RG, Bodenheimer HC, Jr., Monto A, Rossi SJ, Sulkowski MS: Efficacy and safety of peginterferon alfa-2a/ribavirin in methadone maintenance patients: randomized comparison of direct observed therapy and self-administration. The American journal of gastroenterology 2008, 103(11):2757-2765.

119. Bonkovsky HL, Tice AD, Yapp RG, Bodenheimer HC, Jr., Monto A, Rossi SJ, Sulkowski MS: Efficacy and safety of peginterferon alfa-2a/ribavirin in methadone maintenance patients: randomized comparison of direct observed therapy and self-administration. The American journal of gastroenterology 2008, 103(11):2757-2765.

120. Bonner JE, Barritt ASt, Fried MW, Evon DM: Tangible resources for preparing patients for antiviral therapy for chronic hepatitis C. Dig Dis Sci 2012, 57(6):1439-1444.

121. Bonner JE, Barritt ASt, Fried MW, Evon DM: Time to rethink antiviral treatment for hepatitis C in patients with coexisting mental health/substance abuse issues. Dig Dis Sci 2012, 57(6):1469-1474.

122. Bonner JE, Barritt ASt, Fried MW, Evon DM: Tangible resources for preparing patients for antiviral therapy for chronic hepatitis C. Dig Dis Sci 2012, 57(6):1439-1444.

123. Bonner JE, Barritt ASt, Fried MW, Evon DM: Time to rethink antiviral treatment for hepatitis C in patients with coexisting mental health/substance abuse issues. Dig Dis Sci 2012, 57(6):1469-1474.

124. Bonner JE, Barritt ASt, Fried MW, Evon DM: Tangible resources for preparing patients for antiviral therapy for chronic hepatitis C. Dig Dis Sci 2012, 57(6):1439-1444.

125. Bonner JE, Barritt IAS, Fried MW, Evon DM: Tangible resources for preparing patients for antiviral therapy for chronic hepatitis C. Digestive Diseases and Sciences 2012, 57(6):1439-1444.

126. Booth RE, Crowley TJ, Zhang Y: Substance abuse treatment entry, retention and effectiveness: out-of-treatment opiate injection drug users. Drug Alcohol Depend 1996, 42(1):11-20.

127. Boryc K, Anastario MP, Dann G, Chi B, Cicatelli B, Steilen M, Gordon-Boyle K, Singh S, Morris M: A Needs Assessment of Clients With HIV in a Home-Based Care Program in Guyana. Public Health Nursing 2010, 27(6):482-491.

128. Boscarino JA, Kirchner HL, Pitcavage JM, Nadipelli VR, Ronquest NA, Fitzpatrick MH, Han JJ: Factors associated with opioid overdose: a 10-year retrospective study of patients in a large integrated health care system. Subst Abuse Rehabil 2016, 7:131-141.

129. Bouis S, Reif S, Whetten K, Scovil J, Murray A, Swartz M: An integrated, multidimensional treatment model for individuals living with HIV, mental illness, and substance abuse. Health & social work 2007, 32(4):268-278.

130. Bowker K, Orton S, Cooper S, Naughton F, Whitemore R, Lewis S, Bauld L, Sinclair L, Coleman T, Dickinson A et al: Views on and experiences of electronic cigarettes: A qualitative study of women who are pregnant or have recently given birth. BMC Pregnancy and Childbirth 2018, 18 (1) (no pagination)(233).

131. Bowman MA, Neale AV, Seehusen DA: In this issue: Opiates, tobacco, social determinants of health, social accountability for non-profit hospitals, more on PCMH, and clinical topics. Journal of the American Board of Family Medicine 2017, 30(4):399-401.

132. Boyadjieva N: Teaching pharmacology in the medical faculty of medical university in sofia. Autonomic and Autacoid Pharmacology 2010, 30 (1):68-70.

133. Boyer A, Indyk D: Shaping garments of care: Tools for maximizing adherence potential. Social Work in Health Care 2006, 42(3-4):151-166.

134. Branson BM: Home sample collection tests for HIV infection. Journal of the American Medical Association 1998, 280(19):1699-1701.

135. Breitbart W: Pain management and psychosocial issues in HIV and AIDS. Am J Hosp Palliat Care 1996, 13(1):20-29.

136. Breitbart W, McDonald MV, Rosenfeld B, Monkman ND, Passik S: Fatigue in ambulatory AIDS patients. J Pain Symptom Manage 1998, 15(3):159-167.

137. Breitbart W, Rosenfeld B, Passik S, Kaim M, Funesti-Esch J, Stein K: A comparison of pain report and adequacy of analgesic therapy in ambulatory AIDS patients with and without a history of substance abuse. Pain 1997, 72(1-2):235-243.

138. Breitbart W, Rosenfeld BD, Passik SD, McDonald MV, Thaler H, Portenoy RK: The undertreatment of pain in ambulatory AIDS patients. Pain 1996, 65(2-3):243-249.

139. Brener L, Gray R, Cama EJ, Treloar C: 'Makes you wanna do treatment': Benefits of a hepatitis C specialist clinic to clients in Christchurch, New Zealand. Health and Social Care in the Community 2013, 21(2):216-223.

140. Brettle RP, Willocks L, Hamilton BA, Shaw L, Leen CL, Richardson A, Gore SM: Out-patient medical care in Edinburgh for IDU-related HIV. AIDS Care 1994, 6(1):49-58.

141. Brew B, Cysique LA, Jones S, Johnson T, Nath A: Detectable CSF tat despite dual compartment HIV viral suppression with cART. Topics in Antiviral Medicine 2015, 23 (E-1):209.

142. Brief DJ, Bollinger AR, Vielhauer MJ, Berger-Greenstein JA, Morgan EE, Brady SM, Buondonno LM, Keane TM: Understanding the interface of HIV, trauma, post-traumatic stress disorder, and substance use and its implications for health outcomes. AIDS Care - Psychological and Socio-Medical Aspects of AIDS/HIV 2004, 16(SUPPL. 1):S97-S120.

143. Brief DJ, Bollinger AR, Vielhauer MJ, Berger-Greenstein JA, Morgan EE, Brady SM, Buondonno LM, Keane TM, Hiv/Aids Treatment Adherence HO, Cost Study G: Understanding the interface of HIV, trauma, post-traumatic stress disorder, and substance use and its implications for health outcomes. AIDS Care 2004, 16 Suppl 1:S97-120.

144. Brodszky V, Becsi R, Karpati K, Pentek M, Sebestyen A, Boncz I, Gulacsi L: Costs of psoriatic arthritis in Hungary; Results from a cross-sectional survey. Value in Health 2009, 12 (3):A68.

145. Brooner R, Kidorf M, King V, Beilenson P, Svikis D, Vlahov D: Drug abuse treatment success among needle exchange participants. Public Health Reports 1998, 113 Suppl 1:129-139.

146. Brooner RK, Herbst JH, Schmidt CW, Bigelow GE, Costa PT, Jr.: Antisocial personality disorder among drug abusers. Relations to other personality diagnoses and the five-factor model of personality. J Nerv Ment Dis 1993, 181(5):313-319.

147. Brooner RK, Schmidt CW, Felch LJ, Bigelow GE: Antisocial behavior of intravenous drug abusers: implications for diagnosis of antisocial personality disorder. American Journal of Psychiatry 1992, 149(4):482-487.

148. Brosnan S: Our first home care A.I.D.S. patient: Maria. Nursing 1986, 16(9):37-39.

149. Brown BS: AIDS and the provision of drug user treatment. Int J Addict 1990, 25(12A):1503-1514.

150. Brown BS: Observations on the recent history of drug user counseling. Int J Addict 1993, 28(12):1243-1255.

151. Brown K, Crofts N: Health care costs of a continuing epidemic of hepatitis C virus infection among injecting drug users. Aust N Z J Public Health 1998, 22(3 Suppl):384-388.

152. Brown L, Kritz S, Lin M, Louie B, Zavala R, Madray C: Evaluation of an electronic information system to enhance practice at a medication-assisted opioid treatment program. Neuropsychopharmacology 2013, 38:S308-S309.

153. Brown LS, Talal A, Andrews P, McLeod A, Zeremski M, Chen Y, Sylvester C: Integrated telemedicine-based treatment for hepatitis C at an opioid agonist treatment program. Drug and Alcohol Dependence 2017, 171:e27-e28.

154. Bruce RD, Eiserman J, Acosta A, Gote C, Lim JK, Altice FL: Developing a modified directly observed therapy intervention for hepatitis C treatment in a methadone maintenance program: Implications for program replication. American Journal of Drug and Alcohol Abuse 2012, 38(3):206-212.

155. Bruce RD, Lambdin B, Chang O, Masao F, Mbwambo J, Mteza I, Nyandindi C, Zamudio-Haas S, Buma D, Dunbar MS et al: Lessons from Tanzania on the integration of HIV and tuberculosis treatments into methadone assisted treatment. International Journal of Drug Policy 2014, 25(1):22-25.

156. Bruggmann P: Gaps in hepatitis C care of people who use drugs. [German]. Praxis 2017, 106(7):359-363.

157. Bruggmann P: Gaps in Hepatitis C Care of People Who Use Drugs. Praxis 2017, 106(7):359-363.

158. Bruggmann P, Litwin AH: Models of care for the management of hepatitis C virus among people who inject drugs: One size does not fit all. Clinical Infectious Diseases 2013, 57(SUPPL.2):S56-S61.

159. Bruggmann P, Litwin AH: Models of care for the management of hepatitis C virus among people who inject drugs: one size does not fit all. Clin Infect Dis 2013, 57 Suppl 2:S56-61.

160. Bruggmann P, Litwin AH: Models of care for the management of hepatitis C virus among people who inject drugs: one size does not fit all. Clin Infect Dis 2013, 57 Suppl 2:S56-61.

161. Bruneau J, Aswad DJ, Zang G, Roy E: Effect of combined harm reduction strategies on HCV incidence among people who inject drugs in Montreal, Canada. Journal of Hepatology 2016, 1):S462.

162. Brunner N, Senn O, Rosemann T, Falcato L, Bruggmann P: Hepatitis C treatment for multimorbid patients with substance use disorder in a primary care-based integrated treatment centre: A retrospective analysis. European Journal of Gastroenterology and Hepatology 2013, 25(11):1300-1307.

163. Brust JC, Litwin AH, Berg KM, Li X, Heo M, Arnsten JH: Directly observed antiretroviral therapy in substance abusers receiving methadone maintenance therapy does not cause increased drug resistance. AIDS research and human retroviruses 2011, 27(5):535-541.

164. Brust JC, Litwin AH, Berg KM, Li X, Heo M, Arnsten JH: Directly observed antiretroviral therapy in substance abusers receiving methadone maintenance therapy does not cause increased drug resistance. AIDS research and human retroviruses 2011, 27(5):535-541.

165. Buchanan D, Shaw S, Teng W, Hiser P, Singer M: Neighborhood differences in patterns of syringe access, use, and discard among injection drug users: implications for HIV outreach and prevention education. Journal of Urban Health 2003, 80(3):438-454.

166. Burholt R, Phillips C, Verma S, Williams D, Gilleece Y: Developing collaborative nursing models for people living with HIV and hepatitis C co-infection in the advent of increased access to direct acting antivirals: A service development in a large UK urban clinic. HIV Medicine 2018, 19 (Supplement 2):S100.

167. Burman WJ, Cohn DL, Rietmeijer CA, Judson FN, Sbarbaro JA, Reves RR: Short-term incarceration for the management of noncompliance with tuberculosis treatment. Chest 1997, 112(1):57-62.

168. Burman WJ, Cohn DL, Rietmeijer CA, Judson FN, Sbarbaro JA, Reves RR: Noncompliance with directly observed therapy for tuberculosis. Epidemiology and effect on the outcome of treatment. Chest 1997, 111(5):1168-1173.

169. Burton MJ, Voluse A, Anthony VA: Screening and treating hepatitis C virus infection in a residential substance use disorder program. Hepatology 2017, 66 (Supplement 1):315A.

170. Burtscher D, Van Den Bergh R, Toktosunov U, Angmo N, Samieva N, Arechaga EPR: "My favourite day is sunday": Community perceptions of (drug-resistant) tuberculosis and ambulatory tuberculosis care in Kara Suu District, Osh Province, Kyrgyzstan. PLoS ONE 2016, 11 (3) (no pagination)(e0152283).

171. Bury G, O'Kelly F, Pomeroy L: The use of primary care services by drug users attending a HIV prevention unit. Irish medical journal 1993, 86(2):53-55.

172. Busen NH, Beech B: A collaborative model for community-based health care screening of homeless adolescents. Journal of professional nursing : official journal of the American Association of Colleges of Nursing 1997, 13(5):316-324.

173. Butz AM, Stephenson H, Hutton N, Joyner M, Vogelhut J: Care of HIV-risk infants: nursing outreach by PNPs. Journal of pediatric health care : official publication of National Association of Pediatric Nurse Associates & Practitioners 1992, 6(3):138-145.

174. Caekelbergh K, Lamotte M, Nevens F, Colle I, Michielsen P, Robaeys G, Moreno C, Wyffels V: Resource use and cost of hepatitis C related care in Belgium. Value in Health 2011, 14 (7):A394.

175. Cahn P, Perez H, Casiro A, Scaglione C, Muchinik G: [Analysis of the spontaneous demand on an outpatient AIDS clinic in the city of Buenos Aires]. Medicina (B Aires) 1988, 48(2):125-131.

176. Calsyn DA, Campbell AN, Crits-Christoph P, Doyle SR, Tross S, Hatch-Maillette MA, Mandler R: Men in methadone maintenance versus psychosocial outpatient treatment: differences in sexual risk behaviors and intervention effectiveness from a multisite HIV prevention intervention trial. Journal of Addictive Diseases 2010, 29(3):370-382.

177. Calsyn DA, Saxon AJ, Blaes P, Lee-Meyer S: Staffing patterns of American methadone maintenance programs. Journal of Substance Abuse Treatment 1990, 7(4):255-259.

178. Calsyn RJ, Klinkenberg WD, Morse GA, Miller J, Cruthis R, Hiv/Aids Treatment Adherence HO, Cost Study G: Recruitment, engagement, and retention of people living with HIV and co-occurring mental health and substance use disorders. AIDS Care 2004, 16 Suppl 1:S56-70.

179. Campbell AN, Tross S, Hu MC, Pavlicova M, Nunes EV: Predictors of relationship power among drug-involved women. AIDS and behavior 2012, 16(6):1532-1541.

180. Caniglia EC, Cain LE, Sabin CA, Robins JM, Logan R, Abgrall S, Mugavero MJ, Hernandez-Diaz S, Meyer L, Seng R et al: Comparison of dynamic monitoring strategies based on CD4 cell counts in virally suppressed, HIV-positive individuals on combination antiretroviral therapy in high-income countries: a prospective, observational study. The Lancet HIV 2017, 4(6):e251-e259.

181. Cano S, Fuentes M, Ballesteros J, Clavo P, Menendez B, Del Romero J: [Gonorrhea diagnoses in a center for sexually transmitted disease (STD) and their relationship with HIV and other STD, Madrid, 2005]. Enfermedades Infecciosas y Microbiologia Clinica 2009, 27(6):338-341.

182. Capone CT, Melchionda R, DeRovira N, Katowsky K, Condren R, Peterson M: A cognitive-existential analysis of counselor responses to HIV-positive substance misusers in an outpatient methadone program and a residential therapeutic community. Int J Addict 1992, 27(5):587-611.

183. Cardenas Rodriguez M, Perez Gomez P, Lozano Mera L: [A health system that does not live up to expectations. A primary health care team attempt at an integrated approach to a case]. Aten Primaria 1996, 17(3):232-233.

184. Carey MP, Carey KB, Maisto SA, Gordon CM, Schroder KE, Vanable PA: Reducing HIV-risk behavior among adults receiving outpatient psychiatric treatment: results from a randomized controlled trial. J Consult Clin Psychol 2004, 72(2):252-268.

185. Carey MP, Carey KB, Maisto SA, Gordon CM, Vanable PA: Prevalence and correlates of sexual activity and HIV-related risk behavior among psychiatric outpatients. J Consult Clin Psychol 2001, 69(5):846-850.

186. Carey MP, Carey KB, Maisto SA, Schroder KE, Vanable PA, Gordon CM: HIV risk behavior among psychiatric outpatients: association with psychiatric disorder, substance use disorder, and gender. J Nerv Ment Dis 2004, 192(4):289-296.

187. Carmichael JM, Meier J, Robinson A, Taylor J, Higgins DT, Patel S: Leveraging electronic medical record data for population health management in the Veterans Health Administration: Successes and lessons learned. American Journal of Health-System Pharmacy 2017, 74(18):1447-1459.

188. Casado JL, Navas E, Garcia A, Antela A, Redondo E, Fortun J, Guerrero A: Central venous catheter infections in AIDS patients receiving daily home therapy for cytomegalovirus disease. QJM - Monthly Journal of the Association of Physicians 1996, 89(9):695-699.

189. Cavaleri MA, Kalogerogiannis K, McKay MM, Vitale L, Levi E, Jones S, Wallach F, Flynn E: Barriers to HIV care: An exploration of the complexities that influence engagement in and utilization of treatment. Social Work in Health Care 2010, 49(10):934-945.

190. Cavanaugh S, Milne J: Recent changes in consultation-liaison psychiatry. A blueprint for the future. Psychosomatics 1995, 36(2):95-102.

191. Centers for Disease C, Prevention: Integrated prevention services for HIV infection, viral hepatitis, sexually transmitted diseases, and tuberculosis for persons who use drugs illicitly: summary guidance from CDC and the U.S. Department of Health and Human Services. Mmwr 2012, Recommendations and reports : Morbidity and mortality weekly report. Recommendations and reports / Centers for Disease Control. 61(RR-5):1-40.

192. Chadborn TR, McGarrigle CA, Waight PA, Fenton KA, Group HIVTSC: Trends in, and determinants of, HIV testing at genitourinary medicine clinics and general practice in England, 1990-2000. Sexually Transmitted Infections 2004, 80(2):145-150.

193. Chaisson RE, Barnes GL, Hackman J, Watkinson L, Kimbrough L, Metha S, Cavalcante S, Moore RD: A randomized, controlled trial of interventions to improve adherence to isoniazid therapy to prevent tuberculosis in injection drug users. The American journal of medicine 2001, 110(8):610-615.

194. Chaisson RE, Barnes GL, Hackman J, Watkinson L, Kimbrough L, Metha S, Cavalcante S, Moore RD: A randomized, controlled trial of interventions to improve adherence to isoniazid therapy to prevent tuberculosis in injection drug users. The American journal of medicine 2001, 110(8):610-615.

195. Chan SS, Yuen EH, Kew J, Cheung WL, Cocks RA: Community-acquired pneumonia--implementation of a prediction rule to guide selection of patients for outpatient treatment. European journal of emergency medicine : official journal of the European Society for Emergency Medicine 2001, 8(4):279-286.

196. Chasser Y, Kim AY, Freudenreich O: Hepatitis C Treatment: Clinical Issues for Psychiatrists in the Post-Interferon Era. Psychosomatics 2017, 58(1):1-10.

197. Chawarski MC, Mazlan M, Schottenfeld RS: Behavioral drug and HIV risk reduction counseling (BDRC) with abstinence-contingent take-home buprenorphine: A pilot randomized clinical trial. Drug and Alcohol Dependence 2008, 94(1-3):281-284.

198. Cheever LW, Kresina TF, Cajina A, Lubran R: A model federal collaborative to increase patient access to buprenorphine treatment in HIV primary Care. Journal of Acquired Immune Deficiency Syndromes 2011, 56(SUPPL. 1):S3-S6.

199. Chemtob D, Levy A: Rationale and staff evaluation of using a "therapeutic milieu" for substance users within a tuberculosis ward. Substance Use & Misuse 2009, 44(5):672-683.

200. Chen IM, Huang CL, Yeh BJ, Chien YL: Health service utilization of heroin abusers: a retrospective cohort study. Addict Behav 2015, 45:281-286.

201. Chen YC, Chen CK, Lin SK, Chiang SC, Su LW, Wang LJ: Health care service utilization and associated factors among heroin users in northern Taiwan. Addict Behav 2013, 38(11):2635-2638.

202. Cheung R, Mannalithara A, Singh G: Utilization and antiviral therapy in patients with chronic hepatitis C: Analysis of ambulatory care visits in the US. Digestive Diseases and Sciences 2010, 55(6):1744-1751.

203. Cheung RC, Hanson AK, Maganti K, Keeffe EB, Matsui SM: Viral hepatitis and other infectious diseases in a homeless population. Journal of Clinical Gastroenterology 2002, 34(4):476-480.

204. Chiampas T, Badowski ME, Burgos R, Michienzi S, Smith R: Description of collaboration between an interdisciplinary human immunodeficiency virus (HIV) clinic. Pharmacotherapy 2016, 36 (12):e288-e289.

205. Choi BY, Dinitto DM, Marti CN, Choi NG: Impact of mental health and substance use disorders on emergency department visit outcomes for HIV patients. Western Journal of Emergency Medicine 2016, 17(2):153-164.

206. Choi SKY, Boyle E, Cairney J, Grootendorst P, Gardner S, Collins EJ, Kendall C, Rourke SB: Impact of depression and recreational drug use on emergency department encounters and hospital admissions among people living with HIV in Ontario: A secondary analysis using the OHTN cohort study. PLoS ONE 2018, 13 (4) (no pagination)(e0195185).

207. Choi SKY, Boyle E, Cairney J, Grootendorst P, Gardner S, Collins EJ, Kendall C, Rourke SB, Study OC: Impact of depression and recreational drug use on emergency department encounters and hospital admissions among people living with HIV in Ontario: A secondary analysis using the OHTN cohort study. PLoS ONE [Electronic Resource] 2018, 13(4):e0195185.

208. Chonabayashi N, Aoshima M, Yokota N, Kisu T, Yambe Y, Taneda K, Tada H, Nagano H: Mechanical ventilation and long-term respiratory care in the intensive care unit of a general hospital. [Japanese]. Nihon Kyobu Shikkan Gakkai zasshi 1995, 33 Suppl:159-167.

209. Chonabayashi N, Aoshima M, Yokota N, Kisu T, Yambe Y, Taneda K, Tada H, Nagano H: [Mechanical ventilation and long-term respiratory care in the intensive care unit of a general hospital]. Nihon Kyobu Shikkan Gakkai Zasshi Japanese Journal of Thoracic Diseases 1995, 33 Suppl:159-167.

210. Chossegros P, Chevallier P, Trepo C, Sepetjan M: Epidemiology of acute hepatitis among outpatients in the urban community of Lyon, 1983. [French]. Revue d'epidemiologie et de sante publique 1986, 34(3):174-180.

211. Chossegros P, Chevallier P, Trepo C, Sepetjan M: [Epidemiology of acute hepatitis among outpatients in the urban community of Lyon, 1983]. Rev Epidemiol Sante Publique 1986, 34(3):174-180.

212. Chow CH, Sasaki L, Mai T, Hui W, Gensler A, Howard T: Specialty pharmaceuticals: Delivering comprehensive integrated care. Drug Benefit Trends 2004, 16(5):252-256.

213. Chriqui JF, Terry-McElrath Y, McBride DC, Eidson SS: State policies matter: the case of outpatient drug treatment program practices. Journal of Substance Abuse Treatment 2008, 35(1):13-21.

214. Christensen S, Schober A, Mauss S, Busch HW, Gunther R, Teuber G, Buggisch P, Pfeiffer-Vornkahl H, Weber B, Reimer J et al: DAA-treatment of HCV-infected patients on Opioid Substitution Therapy (OST): Does the clinical setting matter? Data from the German Hepatitis C-Registry (DHC-R). Hepatology 2016, 64 (1 Supplement 1):982A-983A.

215. Chrystie IL, Zander L, Tilzey A, Wolfe CD, Kenney A, Banatvala JE: Is HIV testing in antenatal clinics worthwhile? Can we afford it? AIDS Care 1995, 7(2):135-142; discussion 143-135.

216. Chrystie IL, Zander L, Tilzey A, Wolfe CDA, Kenney A, Banatvala JE, Meadows J, Catalan J: Is HIV testing in antenatal clinics worthwhile? Can we afford it? AIDS Care - Psychological and Socio-Medical Aspects of AIDS/HIV 1995, 7(2):135-145.

217. Chu PL, Santos GM, Vu A, Nieves-Rivera I, Colfax GN, Grinsdale J, Huang S, Philip S, Scheer S, Aragon T: Impact of syndemics on people living with HIV in San Francisco. Journal of the International AIDS Society 2012, 15:102-103.

218. Chum HJ, Ilmolelian G, Rieder HL, Msangi J, Mwinyi N, Zwahlen M, Enarson DA, Ipuge YA: Impact of the change from an injectable to a fully oral regimen on patient adherence to ambulatory tuberculosis treatment in Dar es Salaam, Tanzania. Tuber Lung Dis 1995, 76(4):286-289.

219. Cikach F, Hanouneh IA, Zein NN, Alkhouri N, Lopez R, Dweik RA: Breath test analysis in patients with liver disease. American Journal of Respiratory and Critical Care Medicine Conference: American Thoracic Society International Conference, ATS 2013, 187(no pagination).

220. Claborn K, Becker S, Ramsey S, Rich J, Friedmann PD: Mobile technology intervention to improve care coordination between HIV and substance use treatment providers: development, training, and evaluation protocol. Addiction science & clinical practice 2017, 12(1):8.

221. Clapp JD, Burke AC: Supervisor ideology and organizational response: HIV/AIDS prevention in outpatient substance abuse treatment units. Administration in social work 1997, 21(1):49-64.

222. Clarke SM, Mulcahy FM: Antiretroviral therapy for drug users. Int J STD AIDS 2000, 11(10):627-631.

223. Clarke SM, Mulcahy FM: Antiretroviral therapy for drug users. Int J STD AIDS 2000, 11(10):627-631.

224. Closson K, McNeil R, McDougall P, Fernando S, Collins AB, Baltzer Turje R, Howard T, Parashar S: Meaningful engagement of people living with HIV who use drugs: Methodology for the design of a Peer Research Associate (PRA) hiring model. Harm Reduction Journal 2016, 13 (1) (no pagination)(26).

225. Coffin PO, Sullivan SD: Cost-effectiveness of distributing naloxone to heroin users for lay overdose reversal in Russian cities. Journal of Medical Economics 2013, 16(8):1051-1060.

226. Cohen ED: An exploratory attempt to distinguish subgroups among crack-abusing African-American women. Journal of Addictive Diseases 1999, 18(3):41-54.

227. Cohen JF, Vogenthaler N, DelRio C, Armstrong W: The Transition Center: Evaluation of an integrated care model for those with aids, serious mental illness (SMI) and substance use disorders (SUD). Journal of Investigative Medicine 2011, 59 (2):521-522.

228. Cohen MA, Alfonso CA, Haque MM: Lilliputian hallucinations and medical illness. General Hospital Psychiatry 1994, 16(2):141-143.

229. Cohn A, Stanton C, Elmasry H, Ehlke S, Niaura R: Characteristics of U.S. Substance abuse treatment facilities offering HIV services: Results from a national survey. Psychiatric Services 2016, 67(6):692-695.

230. Collins AB, Parashar S, Closson K, Turje RB, Strike C, McNeil R: Navigating identity, territorial stigma, and HIV care services in Vancouver, Canada: A qualitative study. Health Place 2016, 40:169-177.

231. Collins AB, Parashar S, Hogg RS, Fernando S, Worthington C, McDougall P, Turje RB, McNeil R: Integrated HIV care and service engagement among people living with HIV who use drugs in a setting with a community-wide treatment as prevention initiative: A qualitative study in Vancouver, Canada. Journal of the International AIDS Society 2017, 20 (1) (no pagination)(21407).

232. Committee On A: Office-based care for lesbian, gay, bisexual, transgender, and questioning youth. Pediatrics 2013, 132(1):198-203.

233. Compton M, Thumath M, Boyd S, McKinney M: Supported housing as a component of a treatment as prevention (TASP) pilot initiative. Canadian Journal of Infectious Diseases and Medical Microbiology 2012, SA):92A.

234. Connery H, Greenfield S, Livchits V, McGrady L, Patrick N, Lastimoso CS, Heney JH, Nelson AK, Shields A, Stepanova YP et al: Training and fidelity monitoring of alcohol treatment interventions integrated into routine tuberculosis care in Tomsk, Russia: the IMPACT Effectiveness Trial. Subst Use Misuse 2013, 48(9):784-792.

235. Connery H, Greenfield S, Livchits V, McGrady L, Patrick N, Lastimoso CS, Heney JH, Nelson AK, Shields A, Stepanova YP et al: Training and fidelity monitoring of alcohol treatment interventions integrated into routine tuberculosis care in Tomsk, Russia: the IMPACT Effectiveness Trial. Subst Use Misuse 2013, 48(9):784-792.

236. Conniff J, Evensen A: Preexposure prophylaxis (PrEP) for HIV prevention: The primary care perspective. Journal of the American Board of Family Medicine 2016, 29(1):143-151.

237. Connors MM, Lewis BF, Russo JP, Baker LA: Drug treatment staff attitudes toward AIDS and workplace transmission of HIV: a survey and follow-up--Spectrum House, Inc. Journal of Substance Abuse Treatment 1991, 8(4):297-302.

238. Conviser R, Pounds MB: Background for the studies on ancillary services and primary care use. AIDS Care 2002, 14 Suppl 1:S7-14.

239. Conway B, Grebely J, Tossonian H, Lefebvre D, de Vlaming S: A systematic approach to the treatment of HIV and hepatitis C virus infection in the inner city: a Canadian perspective. Clin Infect Dis 2005, 41 Suppl 1:S73-78.

240. Conway B, Grebely J, Tossonian H, Lefebvre D, de Vlaming S: A systematic approach to the treatment of HIV and hepatitis C virus infection in the inner city: a Canadian perspective. Clin Infect Dis 2005, 41 Suppl 1:S73-78.

241. Conway B, Raycraft T, Alimohammadi A, Bhutani Y, Kiani G, Hakobyan S: Efficacy of all-oral HCV therapy in people who inject drugs (PWID). Hepatology 2016, 64 (1 Supplement 1):990A.

242. Cooke A, Saleem H, Mushi D, Mbwambo J, Hassan S, Lambdin BH: Convenience without disclosure: a formative research study of a proposed integrated methadone and antiretroviral therapy service delivery model in Dar es Salaam, Tanzania. Addiction science & clinical practice 2017, 12(1):23.

243. Cooke M, Gourlay L, Collette L, Boccellari A, Chesney MA, Folkman S: Informal caregivers and the intention to hasten AIDS-related death. Archives of Internal Medicine 1998, 158(1):69-75.

244. Cooper CL: Obstacles to successful HCV treatment in substance addicted patients. J Addict Dis 2008, 27(2):61-68.

245. Cooper CL: Obstacles to successful HCV treatment in substance addicted patients. J Addict Dis 2008, 27(2):61-68.

246. Corace K, Garber G, Prematunga C, Laframboise SC, Daley J, Kaluzienski M, Lee C, Shaw-Stiffel T, Balfour L, Cooper C: Enhancing hepatitis c treatment access, Uptake, And outcomes through multidisciplinary care. Hepatology 2011, 1):1181A.

247. Corsi KF, Kwiatkowski CF, Booth RE: Predictors of methamphetamine injection in out-of-treatment IDUs. Substance Use & Misuse 2009, 44(3):332-342.

248. Craig GM, Booth H, Hall J, Story A, Hayward A, Goodburn A, Zumla A: Establishing a new service role in tuberculosis care: the tuberculosis link worker. Journal of Advanced Nursing 2008, 61(4):413-424.

249. Craig GM, Zumla A: The social context of tuberculosis treatment in urban risk groups in the United Kingdom: A qualitative interview study. International Journal of Infectious Diseases 2015, 32:105-110.

250. Crane HM, Delaney JAC, Brown E, Willig JW, Mugavero M, Hunt PW, Rodriguez B, Matthews WC, Saag M, Kitahat MM: Lipid levels among antiretroviral (ARV) naive hiv-infected individuals in routine care. Pharmacoepidemiology and Drug Safety 2010, 19:S196-S197.

251. Crane HM, Fredericksen RJ, Church A, Harrington A, Ciechanowski P, Magnani J, Nasby K, Brown T, Dhanireddy S, Harrington RD et al: A Randomized Controlled Trial Protocol to Evaluate the Effectiveness of an Integrated Care Management Approach to Improve Adherence Among HIV-Infected Patients in Routine Clinical Care: Rationale and Design. JMIR Res Protoc 2016, 5(4):e156.

252. Crane HM, Heckbert SR, Drozd DR, Budoff MJ, Delaney JA, Rodriguez C, Paramsothy P, Lober WB, Burkholder G, Willig JH et al: Lessons learned from the design and implementation of myocardial infarction adjudication tailored for HIV clinical cohorts. American Journal of Epidemiology 2014, 179(8):996-1005.

253. Crane HM, Heckbert SR, Drozd DR, Budoff MJ, Delaney JAC, Rodriguez C, Paramsothy P, Lober WB, Burkholder G, Willig JH et al: Lessons learned from the design and implementation of myocardial infarction adjudication tailored for HIV clinical cohorts. American Journal of Epidemiology 2014, 179(8):996-1005.

254. Crane HM, Nance RM, Hahn A, Tsui J, Chander G, McCaul ME, Lau B, Cropsey K, Fredericksen R, Pence B et al: Decreased alcohol use (even without abstinence) is associatedwith improved depressive symptoms over time. Alcoholism: Clinical and Experimental Research 2018, 42 (Supplement 1):186A.

255. Crane HM, Nance RM, Hahn A, Tsui JI, Mugavero MJ, Lau B, Chander G, Napravnik S, Cropsey K, Christopoulos KA et al: Decreased alcohol use (even without abstinence) is associated with better viral load. Topics in Antiviral Medicine 2018, 26 (Supplement 1):535s.

256. Cranfield S, Feinmann C, Ferlie E, Walter C: Managing service change in post HIV drugs services--a case study from Inner London. British Journal of Addiction 1992, 87(8):1127-1132.

257. Craw JA, Bradley H, Gremel G, West BT, Duke CC, Beer L, Weiser J: Retention in Care Services Reported by HIV Care Providers in the United States, 2013 to 2014. Journal of the International Association of Providers of AIDS Care 2017, 16(5):460-466.

258. Crawford S, Bath N: Peer support models for people with a history of injecting drug use undertaking assessment and treatment for hepatitis C virus infection. Clin Infect Dis 2013, 57 Suppl 2:S75-79.

259. Crawford S, Bath N: Peer support models for people with a history of injecting drug use undertaking assessment and treatment for hepatitis C virus infection. Clin Infect Dis 2013, 57 Suppl 2:S75-79.

260. Crepaz N, Baack BN, Higa DH, Mullins MM: Effects of integrated interventions on transmission risk and care continuum outcomes in persons living with HIV: Meta-analysis, 1996-2014. Aids 2015, 29(18):2371-2383.

261. Crocco JA, Rooney JJ, Lyons HA: Outpatient treatment of tuberculosis in unreliable alcoholic patients. New York State Journal of Medicine 1976, 76(1):58-61.

262. Cropsey KL, Willig JH, Mugavero MJ, Crane HM, McCullumsmith C, Lawrence S, Raper JL, Christopher Mathews W, Boswell S, Kitahata MM et al: Cigarette Smokers are Less Likely to Have Undetectable Viral Loads: Results from Four HIV Clinics. Journal of Addiction Medicine 2016, 10(1):13-19.

263. Cropsey KL, Willig JH, Mugavero MJ, Crane HM, McCullumsmith C, Lawrence S, Raper JL, Mathews WC, Boswell S, Kitahata MM et al: Cigarette Smokers are Less Likely to Have Undetectable Viral Loads: Results From Four HIV Clinics. Journal of Addiction Medicine 2016, 10(1):13-19.

264. Cullen W, Stanley J, Langton D, Kelly Y, Staines A, Bury G: Hepatitis C infection among injecting drug users in general practice: a cluster randomised controlled trial of clinical guidelines' implementation. Br J Gen Pract 2006, 56(532):848-856.

265. Cullen W, Stanley J, Langton D, Kelly Y, Staines A, Bury G: Hepatitis C infection among injecting drug users in general practice: a cluster randomised controlled trial of clinical guidelines' implementation. Br J Gen Pract 2006, 56(532):848-856.

266. Culpepper L: Does screening for depression in primary care improve outcome? Current Psychiatry Reports 2012, 14(4):345-352.

267. Cunningham CO, Sohler NL, McCoy K, Heller D, Selwyn PA: Health care access and utilization patterns in unstably housed HIV-infected individuals in New York City. AIDS Patient Care and STDs 2005, 19(10):690-695.

268. Cunningham CO, Sohler NL, Wong MD, Relf M, Cunningham WE, Drainoni ML, Bradford J, Pounds MB, Cabral HD: Utilization of health care services in hard-to-reach marginalized HIV-infected individuals. AIDS Patient Care and STDs 2007, 21(3):177-186.

269. Cunningham PB, Naar-King S, Ellis DA, Pejuan S, Secord E: Achieving adherence to antiretroviral medications for pediatric HIV disease using an empirically supported treatment: A case report. Journal of Developmental and Behavioral Pediatrics 2006, 27(1):44-50.

270. Cunningham WE, Sohler NL, Tobias C, Drainoni ML, Bradford J, Davis C, Cabral HJ, Cunningham CO, Eldred L, Wong MD: Health services utilization for people with HIV infection:

Comparison of a population targeted for outreach with the U.S. population in care. Medical Care 2006, 44(11):1038-1047.

271. Cuthbert-Allman C, Chausse M: Crossing cultural barriers to care for people with AIDS. Caring : National Association for Home Care magazine 1996, 15(8):14-18.

272. Dahlman D, Palm A, Sunesdotter C, Troberg K, Wallin C: [Low-threshold primary care for patients in opiate maintenance therapy. A pilot project in Malmo, Sweden, integrates primary care and OMT]. Lakartidningen 2016, 113(10):14.

273. Dahlman D, Palm A, Sunesdotter C, Troberg K, Wallin C: Lakartidningen 2016, 113:14.

274. Dakin CL, O'Connor CA, Patsdaughter CA: HAART to heart: HIV-related cardiomyopathy and other cardiovascular complications. AACN clinical issues 2006, 17(1):18-29; quiz 88-2990.

275. Daley D: Reproductive health and AIDS-related services for women: how well are they integrated? Fam Plann Perspect 1994, 26(6):264-269.

276. Daoust R, Saindon Larose D, Sarton Debert FA: [Severe personality disorders associated with addictive behavior]. Can Nurse 1992, 88(5):42-46.

277. Daudon M, Estepa L, Kebede M, Viard JP, Montagnac R, Deray G, Bricaire F: [Urinary calculi and crystalluria in HIV+ patients treated with indinavir sulfate]. Presse Med 1997, 26(34):1612-1615.

278. Davey MP, Foster J, Milton K, Duncan TM: Collaborative Approaches to Increasing Family Support for HIV Positive Youth. Families, Systems and Health 2009, 27(1):39-52.

279. Dawson Rose C, Cuca YP, Kamitani E, Eng S, Zepf R, Draughon J, Lum P: Using Interactive Web-Based Screening, Brief Intervention and Referral to Treatment in an Urban, Safety-Net HIV Clinic. AIDS and behavior 2015, 19:186-193.

280. Dawson-Rose C, Draughon JE, Zepf R, Cuca YP, Huang E, Freeborn K, Lum PJ: Prevalence of Substance Use in an HIV Primary Care Safety Net Clinic: A Call for Screening. The Journal of the Association of Nurses in AIDS Care : JANAC 2017, 28(2):238-249.

281. De la Fuente L, Barrio G, Vicente J, Bravo MJ, Lardelli P: Intravenous administration among heroin users having treatment in Spain. International Journal of Epidemiology 1994, 23(4):805-811.

282. De Leon G: Therapeutic communities: AIDS/HIV risk and harm reduction. Journal of Substance Abuse Treatment 1996, 13(5):411-420; discussion 439.

283. De Luca SM, Franklin C, Yueqi Y, Johnson S, Brownson C: The Relationship Between Suicide Ideation, Behavioral Health, and College Academic Performance. Community mental health journal 2016, 52(5):534-540.

284. de Maat MM, de Boer A, Koks CH, Mulder JW, Meenhorst PL, van Gorp EC, Mairuhu AT, Huitema AD, Beijnen JH: Evaluation of clinical pharmacist interventions on drug interactions in outpatient pharmaceutical HIV-care. J Clin Pharm Ther 2004, 29(2):121-130.

285. de Maat MM, ter Heine R, Mulder JW, Meenhorst PL, Mairuhu AT, van Gorp EC, Huitema AD, Beijnen JH: Incidence and risk factors for nevirapine-associated rash. Eur J Clin Pharmacol 2003, 59(5-6):457-462.

286. De Valle MB, Av Klinteberg V, Alem N, Olsson R, Bjornsson E: Drug-induced liver injury in a Swedish University hospital out-patient hepatology clinic. Aliment Pharmacol Ther 2006, 24(8):1187-1195.

287. de Vries G, van Hest RA: From contact investigation to tuberculosis screening of drug addicts and homeless persons in Rotterdam. European Journal of Public Health 2006, 16(2):133-136.

288. de Vries G, van Hest RA, Richardus JH: Impact of mobile radiographic screening on tuberculosis among drug users and homeless persons. Am J Respir Crit Care Med 2007, 176(2):201-207.

289. DeLorenze GN, Weisner C, Tsai AL, Satre DD, Quesenberry CP, Jr.: Excess mortality among HIV-infected patients diagnosed with substance use dependence or abuse receiving care in a fully integrated medical care program. Alcohol Clin Exp Res 2011, 35(2):203-210.

290. DeLorenze GN, Weisner C, Tsai AL, Satre DD, Quesenberry Jr CP: Excess Mortality Among HIV-Infected Patients Diagnosed With Substance Use Dependence or Abuse Receiving Care in a Fully Integrated Medical Care Program. Alcoholism: Clinical and Experimental Research 2011, 35(2):203-210.

291. Denholm JT, Yong MK, Elliott JH: Long term management of people with HIV. Australian Family Physician 2009, 38(8):574-577.

292. Denis CM, Metzger D, Huang L, Trias V, Auriacombe M, Raguin G, Hoai SMT, Truong GL, Daulouede JP, O'Brien CP: A 6-month follow-up of the integrated treatment for opiate addiction and HIV in Vietnam. Drug and Alcohol Dependence 2015, 156:e56-e57.

293. Desai MM, Rosenheck RA: HIV testing and receipt of test results among homeless persons with serious mental illness. American Journal of Psychiatry 2004, 161(12):2287-2294.

294. Desai MM, Rosenheck RA, Desai RA: Prevalence and correlates of human immunodeficiency virus testing and posttest counseling among outpatients with serious mental illness. J Nerv Ment Dis 2007, 195(9):776-780.

295. Deterding K, Wiegand J, Gruner N, Wedemeyer H: Medical procedures as a risk factor for HCV infection in developed countries: Do we neglect a significant problem in medical care? Journal of Hepatology 2008, 48(6):1019-1020.

296. Deuffic-Burban S, Delarocque-Astagneau E, Abiteboul D, Bouvet E, Yazdanpanah Y: Blood-borne viruses in health care workers: prevention and management. J Clin Virol 2011, 52(1):4-10.

297. Di Sorbo PG, Chifamba DD, Mastrojohn IJ, Sisimayi CN, Williams SH: The zimbabwe rural palliative care initiative: PCI-Z. Journal of Pain and Symptom Management 2010, 40(1):19-22.

298. Diaz A, Ten A, Marcos H, Gutierrez G, Gonzalez-Garcia J, Moreno S, Barrios AM, Arponen S, Portillo A, Serrano R et al: Factors determining irregular attendance to follow-up visits among human immunodeficiency virus patients: Results of the hospital survey of patients infected with human immunodeficiency virus. [Spanish]. Enfermedades Infecciosas y Microbiologia Clinica 2015, 33(5):324-330.

299. Dieperink E, Fuller B, Isenhart C, McMaken K, Lenox R, Pocha C, Thuras P, Hauser P: Efficacy of motivational enhancement therapy on alcohol use disorders in patients with chronic hepatitis C: a randomized controlled trial. Addiction 2014, 109(11):1869-1877.

300. Dieperink E, Fuller B, Isenhart C, McMaken K, Lenox R, Pocha C, Thuras P, Hauser P: Efficacy of motivational enhancement therapy on alcohol use disorders in patients with chronic hepatitis C: a randomized controlled trial. Addiction 2014, 109(11):1869-1877.

301. Dieperink E, Fuller B, Thuras P, McMaken K, Lenox R, Isenhart C, Pocha C, Hauser P: Randomized Controlled trial of Motivational Enhancement Therapy to Reduce Alcohol in Patients with Chronic Hepatitis C. Hepatology 2012, 56:579A.

302. Dieperink E, Ho SB, Heit S, Durfee JM, Thuras P, Willenbring ML: Significant reductions in drinking following brief alcohol treatment provided in a hepatitis C clinic. Psychosomatics 2010, 51(2):149-156.

303. Dieperink E, Ho SB, Thuras P, Colton S, Knott A, Pocha C: Impact of SVR on all-cause and liver-related mortality in a cohort of Veterans with chronic hepatitis C. Hepatology 2012, 56:680A.

304. Dieperink E, Knott A, Thuras P, Pocha C: The effect of stimulant use on antiviral treatment in an integrated hepatitis clinic. General Hospital Psychiatry 2013, 35(4):387-392.

305. Dierst-Davies R, Wohl AR, Pinney G, Johnson CH, Vincent-Jones C, Perez MJ: Methods to Obtain a Representative Sample of Ryan White-Funded Patients for a Needs Assessment in Los Angeles County: Results from a Replicable Approach. Journal of the International Association of Providers of AIDS Care 2017, 16(4):383-395.

306. Diez M, Diaz A, Garriga C, Pons M, Ten A, Marcos H, Gutierrez G, Moreno S, Gonzalez-Garcia J, Arponen S et al: A low-cost, sustainable, second generation system for surveillance of people living with HIV in Spain: 10-year trends in behavioural and clinical indicators, 2002 to 2011. Eurosurveillance 2014, 19(20).

307. Dijkgraaf MG, Luijben AH, Jager JC, Schrijvers AJ, Borleffs JC: In-patient care for symptomatic, HIV-infected persons: a longitudinal study of hospitalizations, in-patient drug use, and related costs. AIDS Care 1995, 7(3):321-336.

308. Dillard D, Bincsik AK, Zebley C, Mongare K, Harrison J, Gerardi KE, Parcher DW: Integrated nested services: Delaware's experience treating minority substance abusers at risk for HIV or HIV positive. Journal of Evidence-Based Social Work 2010, 7(1-2):130-143.

309. Dimova RB, Zeremski M, Jacobson IM, Hagan H, Des Jarlais DC, Talal AH: Determinants of hepatitis C virus treatment completion and efficacy in drug users assessed by meta-analysis. Clin Infect Dis 2013, 56(6):806-816.

310. Dimova RB, Zeremski M, Jacobson IM, Hagan H, Des Jarlais DC, Talal AH: Determinants of hepatitis C virus treatment completion and efficacy in drug users assessed by meta-analysis. Clin Infect Dis 2013, 56(6):806-816.

311. Dinwiddie SH, Cottler L, Compton W, Abdallah AB: Psychopathology and HIV risk behaviors among injection drug users in and out of treatment. Drug Alcohol Depend 1996, 43(1-2):1-11.

312. DiPietro BY, Kindermann D, Schenkel SM: Ill, itinerant, and insured: the top 20 users of emergency departments in Baltimore city. ScientificWorldJournal 2012, 2012:726568.

313. DiPietro Mager NA: Fulfilling an Unmet Need: Roles for Clinical Pharmacists in Preconception Care. Pharmacotherapy 2016, 36(2):141-151.

314. Dixon P, Higginson I: AIDS and cancer pain treated with slow release morphine. Postgraduate Medical Journal 1991, 67(SUPPL. 2):S92+S93+S94+.

315. Dlamini S, Taylor M, Mkhize N, Huver R, Sathiparsad R, de Vries H, Naidoo K, Jinabhai C: Gender factors associated with sexual abstinent behaviour of rural South African high school going youth in KwaZulu-Natal, South Africa. Health Educ Res 2009, 24(3):450-460.

316. Dobson AS, Clarke N: Pediatric AIDS program addresses whole families. Caring 1996, 15(8):42-44.

317. Dolan KA, Shearer J, White B, Zhou J, Kaldor J, Wodak AD: Four-year follow-up of imprisoned male heroin users and methadone treatment: mortality, re-incarceration and hepatitis C infection. Addiction 2005, 100(6):820-828.

318. Dolan KA, Shearer J, White B, Zhou J, Kaldor J, Wodak AD: Four-year follow-up of imprisoned male heroin users and methadone treatment: mortality, re-incarceration and hepatitis C infection. Addiction 2005, 100(6):820-828.

319. Dolce P, Brin-Clement S, Bernatchez H, Jutras P, Tourangeau F: Multidisciplinary clinic for the treatment of hepatitis C: An useful tool for the management of the patients; review of the experience of a regional hospital. Sexually Transmitted Infections 2011, 87:A295.

320. Doll M, Ward L, Bettiker R, Samuel R: HIV screening practices in an Urban outpatient resident clinic. Journal of General Internal Medicine 2011, 26:S200.

321. Doloresco F, Fominaya C, Schumock GT, Vermeulen LC, Matusiak L, Hunkler RJ, Shah ND, Hoffman JM: Projecting future drug expenditures - 2011. American Journal of Health-System Pharmacy 2011, 68(10):921-932.

322. Domb M: Connecting the dots: integrating HIV and substance abuse. Focus (San Francisco, Calif) 2003, 18(7):1-4.

323. Dombrowski JC, Kitahata MM, Van Rompaey SE, Crane HM, Mugavero MJ, Eron JJ, Boswell SL, Rodriguez B, Mathews WC, Martin JN et al: High levels of antiretroviral use and viral suppression among persons in HIV care in the United States, 2010. Journal of Acquired Immune Deficiency Syndromes 2013, 63(3):299-306.

324. Dombrowski JC, Simoni JM, Katz DA, Golden MR: Barriers to HIV Care and Treatment Among Participants in a Public Health HIV Care Relinkage Program. AIDS Patient Care STDS 2015, 29(5):279-287.

325. Donastorg Y, Barrington C, Perez M, Kerrigan D: Abriendo Puertas: Baseline findings from an integrated intervention to promote prevention, treatment and care among FSW living with HIV in the Dominican Republic. PLoS ONE 2014, 9 (2) (no pagination)(e88157).

326. Donastorg Y, Barrington C, Perez M, Kerrigan D: Abriendo Puertas: baseline findings from an integrated intervention to promote prevention, treatment and care among FSW living with HIV in the Dominican Republic.[Erratum appears in PLoS One. 2014;9(3):e92480]. PLoS ONE [Electronic Resource] 2014, 9(2):e88157.

327. Donenberg GR, Emerson E, Bryant FB, Wilson H, Weber-Shifrin E: Understanding AIDS-risk behavior among adolescents in psychiatric care: links to psychopathology and peer relationships. J Am Acad Child Adolesc Psychiatry 2001, 40(6):642-653.

328. Douaihy AB, Jou RJ, Gorske T, Salloum IM: Triple diagnosis: dual diagnosis and HIV disease, part 2. AIDS Reader 2003, 13(8):375-382.

329. Douaihy AB, Stowell KR, Bui T, Daley D, Salloum I: HIV/AIDS and homelessness, Part 1: background and barriers to care. The AIDS reader 2005, 15(10):516-520, 527.

330. Douaihy AB, Stowell KR, Bui T, Daley D, Salloum I: HIV/AIDS and homelessness, Part 2: treatment issues. The AIDS reader 2005, 15(11):604-606, 611-613, 618.

331. Doucette KE, Robson V, Shafran S, Kunimoto D: Improving access to care by allowing self-referral to a hepatitis C clinic. Canadian Journal of Gastroenterology 2009, 23(6):421-424.

332. Drainoni ML, Farrell C, Sorensen-Alawad A, Palmisano JN, Chaisson C, Walley AY: Patient perspectives of an integrated program of medical care and substance use treatment. AIDS Patient Care and STDs 2014, 28(2):71-81.

333. Du WJ, Xiang YT, Wang ZM, Chi Y, Zheng Y, Luo XN, Cai ZJ, Ungvari GS, Gerevich J: Socio-demographic and clinical characteristics of 3129 heroin users in the first methadone maintenance treatment clinic in China. Drug Alcohol Depend 2008, 94(1-3):158-164.

334. Duff K: You can make a difference in the administration of intravenous immunoglobulin therapy. Journal of Infusion Nursing 2006, 29(3 SUPPL.):S5-S14.

335. Duff P, Muldoon KA, Akello M, Simo A, Isiko S, Muzaaya G, Shannon K: Intersecting reproductive health and HIV risks: Correlates of unintended pregnancies among a cohort of young women sex workers working in bars, truck-stops and lodges in postconflict Northern Uganda. Canadian Journal of Infectious Diseases and Medical Microbiology 2013, SA):107A.

336. Duffy M, Sharer M, Cornman H, Pearson J, Pitorak H, Fullem A: Integrating Mental Health and HIV Services in Zimbabwean Communities: A Nurse and Community-led Approach to Reach the Most Vulnerable. The Journal of the Association of Nurses in AIDS Care : JANAC 2017, 28(2):186-198.

337. Dumchev K, Dvoryak S, Chernova O, Morozova O, Altice FL: Retention in medication-assisted treatment programs in Ukraine-Identifying factors contributing to a continuing HIV epidemic. International Journal of Drug Policy 2017, 48:44-53.

338. Dumchev K, Dvoryak S, Chernova O, Morozova O, Altice FL: Retention in medication-assisted treatment programs in Ukraine-Identifying factors contributing to a continuing HIV epidemic. International Journal of Drug Policy 2017, 48:44-53.

339. Dumont T, Balaan M, Barker BJ: Just another office visit for shortness of breath. Chest Conference: CHEST 2013, 144(4 MEETING ABSTRACT).

340. Dux MC, Lee-Wilk T: Integration of Neuropsychological Services in a VA HIV Primary Care Clinic. Arch Clin Neuropsychol 2018, 33(3):290-300.

341. Eaton EF, Tamhane A, Davy-Mendez T, Mathews WC, Moore RD, Saag MS, Mugavero MJ: Trends in ART Prescription, Durability and Modification: New drugs, more changes, but less failure. Aids 2017, 30:30.

342. Eaton EF, Tamhane A, Davy-Mendez T, Mathews WC, Moore RD, Saag MS, Mugavero MJ: Trends in antiretroviral therapy prescription, durability and modification: New drugs, more changes, but less failure. Aids 2018, 32(3):347-355.

343. Eaton EF, Tamhane A, Davy-Mendez T, Mathews WC, Moore RD, Saag MS, Mugavero MJ: Trends in antiretroviral therapy prescription, durability and modification: new drugs, more changes, but less failure. AIDS 2018, 32(3):347-355.

344. Ebner N, Wanner C, Winklbaur B, Matzenauer C, Jachmann CA, Thau K, Fischer G: Retention rate and side effects in a prospective trial on hepatitis C treatment with pegylated interferon alpha-2a and ribavirin in opioid-dependent patients. Addiction biology 2009, 14(2):227-237.

345. Ebner N, Wanner C, Winklbaur B, Matzenauer C, Jachmann CA, Thau K, Fischer G: Retention rate and side effects in a prospective trial on hepatitis C treatment with pegylated interferon alpha-2a and ribavirin in opioid-dependent patients. Addiction biology 2009, 14(2):227-237.

346. Edelman E, Hansen N, Cutter C, Danton C, Fiellin L, O'Connor P, Williams E, Maisto S, Bryant K, Fiellin D: Implementing integrated stepped care for unhealthy alcohol use in HIV treatment settings: A qualitative study using the CFIR constructs. Alcoholism: Clinical and Experimental Research 2015, 39:204A.

347. Edelman EJ, Maisto SA, Hansen NB, Cutter CJ, Dziura J, Fiellin LE, O'Connor PG, Bedimo R, Gibert C, Marconi VC et al: Protocol for three parallel multi-site stepped care effectiveness studies for unhealthy alcohol use in HIV-positive patients. Contemporary Clinical Trials 2017, 52:80-90.

348. Edelman EJ, Maisto SA, Hansen NB, Cutter CJ, Dziura J, Fiellin LE, O'Connor PG, Bedimo R, Gibert C, Marconi VC et al: The Starting Treatment for Ethanol in Primary care Trials (STEP Trials): Protocol for Three Parallel Multi-Site Stepped Care Effectiveness Studies for Unhealthy Alcohol Use in HIV-Positive Patients. Contemporary Clinical Trials 2017, 52:80-90.

349. Edlin BR, Kresina TF, Raymond DB, Carden MR, Gourevitch MN, Rich JD, Cheever LW, Cargill VA: Overcoming barriers to prevention, care, and treatment of hepatitis C in illicit drug users. Clin Infect Dis 2005, 40 Suppl 5:S276-285.

350. Edlin BR, Kresina TF, Raymond DB, Carden MR, Gourevitch MN, Rich JD, Cheever LW, Cargill VA: Overcoming barriers to prevention, care, and treatment of hepatitis C in illicit drug users. Clin Infect Dis 2005, 40 Suppl 5:S276-285.

351. Egan JE, Netherland J, Gass J, Finkelstein R, Weiss L: Patient perspectives on buprenorphine/naloxone treatment in the context of HIV care. Journal of Acquired Immune Deficiency Syndromes 2011, 56(SUPPL. 1):S46-S53.

352. Egan JE, Netherland J, Gass J, Finkelstein R, Weiss L, Collaborative B: Patient perspectives on buprenorphine/naloxone treatment in the context of HIV care. J Acquir Immune Defic Syndr 2011, 56 Suppl 1:S46-53.

353. Elhassan MMA: Africa. Journal of Thoracic Oncology 2015, 2):S103.

354. Ellen JM, Bonu S, Arruda JS, Ward MA, Vogel R: Comparison of clients of a mobile health van and a traditional STD clinic. J Acquir Immune Defic Syndr 2003, 32(4):388-393.

355. Ellen JM, Liang TS, Jacob CA, Erbelding E, Christmyer C: Post-HIV test counselling of clients of a mobile STD/HIV clinic. International Journal of STD and AIDS 2004, 15(11):728-731.

356. Ellis D, Collis I, King M: A controlled comparison of HIV and general medical referrals to a liaison psychiatry service. AIDS Care 1994, 6(1):69-76.

357. Ellis D, Collis I, King M: Personality disorder and sexual risk taking among homosexually active and heterosexually active men attending a genito-urinary medicine clinic. J Psychosom Res 1995, 39(7):901-910.

358. Emery C, George B, Gomas JM, Delfieu D, Mimaud V, Guillon P, Fagnani F: Pain management in general practice: The EPIDOL study. [French]. Douleurs 2005, 6(6):366-373.

359. Emlet CA, Berghuis JP: Service priorities, use, and needs: Views of older and younger consumers living with HIV/AIDS. Journal of Mental Health and Aging 2002, 8(4):307-318.

360. Emlet CA, Gusz SS: Service use patterns in HIV/AIDS case management: a five-year study. Journal of Case Management 1998, 7(1):3-9.

361. Emlet CA, Scott Gusz S: Service use patterns in HIV/AIDS case management: A five-year study. Journal of Case Management 1998, 7(1):3-9.

362. Emmanuel F, Adrien A, Athar U, Imran M, Reza T, Blanchard J: Using surveillance data for action: Lessons learnt from the second generation HIV/AIDS surveillance project in Pakistan. Eastern Mediterranean Health Journal 2011, 17(8):712-718.

363. Etgen T, Eberl B, Freudenberger T: Anti-retroviral therapy-induced status epilepticus in "pseudo-HIV serodeconversion". General Hospital Psychiatry 2010, 32(5):559.e515-557.

364. Ettner SL, Conover CJ, Proescholdbell RJ, Weaver MR, Ang A, Arno PS, Hiv/Aids Treatment Adherence HO, Cost Study G: Triply-diagnosed patients in the HIV/AIDS Treatment Adherence, Health Outcomes and Cost Study: patterns of home care use. AIDS Care 2008, 20(10):1177-1189.

365. Ettner SL, Conover CJ, Proescholdbell RJ, Weaver MR, Ang A, Arno PS, Kalichman S, Knipmeyer MC, Meyer P, Bell J et al: Triply-diagnosed patients in the HIV/AIDS treatment adherence, health outcomes and cost study: Patterns of home care use. AIDS Care - Psychological and Socio-Medical Aspects of AIDS/HIV 2008, 20(10):1177-1189.

366. Ettner SL, Weissman J: Utilization of formal and informal home care by AIDS patients in Boston: a comparison of intravenous drug users and homosexual males. Medical care 1994, 32(5):459-470.

367. Ettner SL, Weissman J: Utilization of formal and informal home care by AIDS patients in Boston: a comparison of intravenous drug users and homosexual males. Medical Care 1994, 32(5):459-470.

368. Evon DM, Golin CE, Fried MW, Keefe FJ: Chronic hepatitis C and antiviral treatment regimens: where can psychology contribute? Journal of consulting and clinical psychology 2013, 81(2):361-374.

369. Evon DM, Golin CE, Fried MW, Keefe FJ: Chronic hepatitis C and antiviral treatment regimens: where can psychology contribute? J Consult Clin Psychol 2013, 81(2):361-374.

370. Evon DM, Simpson K, Kixmiller S, Galanko J, Dougherty K, Golin C, Fried MW: A randomized controlled trial of an integrated care intervention to increase eligibility for chronic hepatitis C treatment. American Journal of Gastroenterology 2011, 106(10):1777-1786.

371. Evon DM, Simpson K, Kixmiller S, Galanko J, Dougherty K, Golin C, Fried MW: A randomized controlled trial of an integrated care intervention to increase eligibility for chronic hepatitis C treatment. American Journal of Gastroenterology 2011, 106(10):1777-1786.

372. Evon DM, Simpson K, Kixmiller S, Galanko JA, Dougherty KA, Golin CE, Fried MW: A randomized clinical trial to evaluate the impact of an integrated care intervention on eligibility rates for hepatitis C treatment. Hepatology 2010, 52:408A-409A.

373. Fahey S: Developing a nursing service for patients with hepatitis C. Nursing standard (Royal College of Nursing (Great Britain) : 1987) 2007, 21(43):35-40.

374. Fahey S: Developing a nursing service for patients with hepatitis C. Nurs Stand 2007, 21(43):35-40.

375. Failde Garrido JM, Lopez Castro J, Fernandez Rodriguez V, Fernandez Rodriguez R: [HIV-1 infection, use of drugs and neuropsychologic efficiency]. Anales de medicina interna (Madrid, Spain : 1984) 2005, 22(12):569-574.

376. Falzon D, Jaramillo E, Schunemann HJ, Arentz M, Bauer M, Bayona J, Blanc L, Caminero JA, Daley CL, Duncombe C et al: WHO guidelines for the programmatic management of drug-resistant tuberculosis: 2011 update. European Respiratory Journal 2011, 38(3):516-528.

377. Falzon D, Jaramillo E, Schunemann HJ, Arentz M, Bauer M, Bayona J, Blanc L, Caminero JA, Daley CL, Duncombe C et al: WHO guidelines for the programmatic management of drug-resistant tuberculosis: 2011 update. European Respiratory Journal 2011, 38(3):516-528.

378. Fassati A: Multiple roles of the capsid protein in the early steps of HIV-1 infection. Virus research 2012, 170(1-2):15-24.

379. Fatukasi TV, Cole SR, Moore RD, Mathews WC, Edwards JK, Eron JJ: Risk factors for delayed antiretroviral therapy initiation among HIV-seropositive patients. PLoS ONE 2017, 12 (7) (no pagination)(e0180843).

380. Fatukasi TV, Cole SR, Moore RD, Mathews WC, Edwards JK, Eron JJ, investigators C: Risk factors for delayed antiretroviral therapy initiation among HIV-seropositive patients. PLoS ONE [Electronic Resource] 2017, 12(7):e0180843.

381. Federman AD, Arnsten JH: Primary care affiliations of adults in a methadone program with onsite care. Journal of Addictive Diseases 2007, 26(1):27-34.

382. Feingold A, Slammon WR: A model integrating mental health and primary care services for families with HIV. General Hospital Psychiatry 1993, 15(5):290-300.

383. Felippe MJDB, Meira DA: Comparison of risk factors among blood donors, volunteers and replacement individuals, infected or not by hepatitis c virus. Journal of Venomous Animals and Toxins Including Tropical Diseases 2009, 15(1):103-124.

384. Felix-Ortiz de la Garza M, Sorensen JL: A self-help group for drug-addicted clients. Assisted implementation in outpatient treatment. Journal of Substance Abuse Treatment 1995, 12(4):259-268.

385. Fenech M, D'Arcy D: Specialist hepatology nurses leading the way. Qld Nurse 2004, 23(5):15.

386. Fernando S, McNeil R, Closson K, Samji H, Kirkland S, Strike C, Turje RB, Zhang W, Hogg RS, Parashar S: An integrated approach to care attracts people living with HIV who use illicit drugs in an urban centre with a concentrated HIV epidemic. Harm Reduction Journal 2016, 13 (1) (no pagination)(31).

387. Fernando S, McNeil R, Closson K, Samji H, Kirkland S, Strike C, Turje RB, Zhang W, Hogg RS, Parashar S: An integrated approach to care attracts people living with HIV who use illicit drugs in an urban centre with a concentrated HIV epidemic. Harm Reduction Journal 2016, 13(1):31.

388. Fernando SM, Parashar S, McNeil R, McDougall P, Lamoureux R, Ranville F, Baltzer-Turje R, Hogg R: Practicing gipa through pra-led survey administration. Canadian Journal of Infectious Diseases and Medical Microbiology 2015, 26:110B.

389. Fernando SM, Parashar S, Pauly B, Worthington C, McNeil R, Strike C, Kirkland S, Samji H, Wong J, Oliveira N et al: Meeting needs of plHIV with complex health issues: The Dr. Peter centre, an integrative healthcare service. Canadian Journal of Infectious Diseases and Medical Microbiology 2015, SB):86B.

390. Ferrando SJ, Batki SL: Substance abuse and HIV infection. New Directions for Mental Health Services 2000(87):57-67.

391. Ferrando SJ, Rabkin JG, de Moore GM, Rabkin R: Antidepressant treatment of depression in HIV-seropositive women. J Clin Psychiatry 1999, 60(11):741-746.

392. Feurdean M, Yousaf A, Kothari N, Natale-Pereira A: Team based learning approach to resident education in out patient internal medicine. Journal of General Internal Medicine 2016, 1):S847.

393. Fhima A, Henrion R, Lowenstein W, Charpak Y: Two-year follow-up of an opioid-user cohort treated with high-dose buprenorphine (Subutex). [French]. Annales de medecine interne 2001, 152 Suppl 3:IS26-36.

394. Fhima A, Henrion R, Lowenstein W, Charpak Y: [Two-year follow-up of an opioid-user cohort treated with high-dose buprenorphine (Subutex)]. Annales de Medecine Interne 2001, 152 Suppl 3:IS26-36.

395. Fiellin D, Fiellin L: Trials of integrated stepped care and injectable naltrexone for unhealthy alcohol use in HIV-infected patients. Alcoholism: Clinical and Experimental Research 2014, 38:359A.

396. Fiellin DA, Justice AC, Braithwaite RS: Consortium to improve outcomes in HIV/AIDS, Alcohol, aging & multisubstance use (compaaas). Alcoholism: Clinical and Experimental Research 2012, 36:145A.

397. Filiniuk OV, Ianova GV, Strelis AK, Urazova OI, Zemlianaia NA, Buinova LN, Voronkova OV, Ianov SA, Shchegertsov D: [Multidrug pulmonary tuberculosis: sociomedical features and the efficiency of inpatient treatment]. Probl 2008(8):23-28; discussion 28.

398. Filiniuk OV, Ianova GV, Strelis AK, Urazova OI, Zemlianaia NA, Buinova LN, Voronkova OV, Ianov SA, Shchegertsov DI: Multidrug pulmonary tuberculosis: sociomedical features and the efficiency of inpatient treatment. [Russian]. Problemy tuberkuleza i boleznei legkikh 2008(8):23-28; discussion 28.

399. Filippovych S, Burgay O: Development of treatment model for HIV/HCV co-infected OST patients as the basis for scaling up access to HCV treatment for key populations in Ukraine. Journal of Viral Hepatitis 2015, 22:89.

400. Filippovych S, Burgay O, Pavlyiuk I: Results of hepatitis C treatment program among people who inject drugs. Journal of Hepatology 2017, 66 (1 Supplement 1):S737.

401. Finkelstein R, Netherland J, Sylla L, Gourevitch MN, Cajina A, Cheever L: Policy implications of integrating buprenorphine/naloxone treatment and HIV care. Journal of Acquired Immune Deficiency Syndromes 2011, 56(SUPPL. 1):S98-S104.

402. Finkelstein R, Netherland J, Sylla L, Gourevitch MN, Cajina A, Cheever L, Collaborative B: Policy implications of integrating buprenorphine/naloxone treatment and HIV care. J Acquir Immune Defic Syndr 2011, 56 Suppl 1:S98-S104.

403. Fisch HU, Lauterburg BH: [Treatment of alcoholic liver diseases and psychiatric and psychosocial problems]. Ther Umsch 1998, 55(2):80-83.

404. Fischer B, Cruz MF, Rehm J: Illicit opioid use and its key characteristics: A select overview and evidence from a Canadian multisite cohort of illicit opioid users (OPICAN). Canadian Journal of Psychiatry 2006, 51(10):624-634.

405. Flanzer J: Health Services Research: Drug Use and Human Immunodeficiency Virus in the United States. Clinical Infectious Diseases 2003, 37(12 SUPPL. 5):S439-S444.

406. Flanzer J: Health services research: drug use and human immunodeficiency virus in the United States. Clinical Infectious Diseases 2003, 37 Suppl 5:S439-444.

407. Fleishman JA, Gebo KA, Reilly ED, Conviser R, Christopher Mathews W, Todd Korthuis P, Hellinger J, Rutstein R, Keiser P, Rubin H et al: Hospital and outpatient health services utilization among HIV-infected adults in care 2000-2002. Medical Care 2005, 43(9 Suppl):III40-52.

408. Fleishman JA, Gebo KA, Reilly ED, Conviser R, Mathews WC, Korthuis PT, Hellinger J, Rutstein R, Keiser P, Rubin H et al: Hospital and outpatient health services utilization among HIV-infected adults in care 2000-2002. Medical Care 2005, 43(9 SUPPL.):III40-III52.

409. Fletcher JB, Reback CJ: Depression mediates and moderates effects of methamphetamine use on sexual risk taking among treatment-seeking gay and bisexual men. Health Psychology 2015, 34(8):865-869.

410. Foley ME, Ehr AP, Raza B, Devlin CJ: Tuberculosis surveillance in a therapeutic community. Journal of Addictive Diseases 1995, 14(1):55-65.

411. Ford N, Wiktor S, Kaplan K, Andrieux-Meyer I, Hill A, Radhakrishnan P, Londeix P, Forette C, Momenghalibaf A, Verster A et al: Ten priorities for expanding access to HCV treatment for people who inject drugs in low- and middle-income countries. International Journal of Drug Policy 2015, 26(11):1088-1093.

412. Fortuna LR, Alvarez K, Ramos Ortiz Z, Wang Y, Mozo Alegria X, Cook BL, Alegria M: Mental health, migration stressors and suicidal ideation among Latino immigrants in Spain and the United States. European Psychiatry 2016, 36:15-22.

413. Fortuna LR, Alvarez K, Ramos Ortiz Z, Wang Y, Mozo Alegria X, Cook BL, Alegria M: Mental health, migration stressors and suicidal ideation among Latino immigrants in Spain and the United States. Eur Psychiatry 2016, 36:15-22.

414. Fox AD, Hawks LC, Norton BL, Litwin AH, Cunningham C: Integrated care increases evaluation but not treatment for chronic hepatitis C virus infection in primary care. Journal of General Internal Medicine 2015, 30:S194-S195.

415. Fox AD, Masyukova M, Cunningham CO: Optimizing psychosocial support during office-based buprenorphine treatment in primary care: Patients' experiences and preferences. Substance abuse 2016, 37(1):70-75.

416. Fox AD, Starrels JL, Cunningham C: Prior incarceration is associated with distrust in the health care system but not with trust in health care providers. Journal of General Internal Medicine 2013, 28:S157-S158.

417. Fragomeli V, Weltman M: Addressing viral hepatitis in the opiate substitution setting: An integrated nursing model of care. Journal of Gastroenterology and Hepatology (Australia) 2015, 30(S2):6-11.

418. Fragomeli V, Weltman M: Addressing viral hepatitis in the opiate substitution setting: an integrated nursing model of care. J Gastroenterol Hepatol 2015, 30 Suppl 2:6-11.

419. Franco RA, Tamhane A, Overton ET: Impact of Poor Retention in HIV Medical Care on Hepatitis B Vaccination. Journal of the International Association of Providers of AIDS Care 2015, 14(2):185-190.

420. Fraser C, Milne R, Drost A: The benefits of onsite transient elastography use in an inner city community health centre. Canadian Journal of Gastroenterology and Hepatology Conference 2016(pagination).

421. Fredericksen R, Gibbons L, Fitzsimmons E, Mayer K, Mathews W, Mugavero M, Christopoulos K, Chander G, Hutton H, McCaul M et al: Illicit drug use among alcohol-misusing gay and bisexual men in HIV care. Alcoholism: Clinical and Experimental Research 2016, 40:72A.

422. Freedman JB, O'Dowd MA, McKegney FP, Kaplan IJ, Bernstein G, Biderman DJ, Gomez MF: Managing diazepam abuse in an AIDS-related psychiatric clinic with a high percentage of substance abusers. Psychosomatics 1996, 37(1):43-47.

423. Freedman K, Nathanson J: Interferon-based hepatitis C treatment in patients with pre-existing severe mental illness and substance use disorders. Expert review of anti-infective therapy 2009, 7(3):363-376.

424. Friedmann PD, Alexander JA, Jin L, D'Aunno TA: On-site primary care and mental health services in outpatient drug abuse treatment units. Journal of Behavioral Health Services and Research 1999, 26(1):80-94.

425. Friedmann PD, Alexander JA, Jin L, D'Aunno TA: On-site primary care and mental health services in outpatient drug abuse treatment units. J Behav Health Serv Res 1999, 26(1):80-94.

426. Friedmann PD, D'Aunno TA, Jin L, Alexander JA: Medical and psychosocial services in drug abuse treatment: do stronger linkages promote client utilization? Health Services Research 2000, 35(2):443-465.

427. Frimpong JA, D'Aunno T, Helleringer S, Metsch LR: Spillover effects of HIV testing policies: changes in HIV testing guidelines and HCV testing practices in drug treatment programs in the United States. BMC public health 2016, 16:666.

428. Frimpong JA, D'Aunno T, Helleringer S, Metsch LR: Spillover effects of HIV testing policies: changes in HIV testing guidelines and HCV testing practices in drug treatment programs in the United States. BMC Public Health 2016, 16:666.

429. Frimpong JA, D'Aunno T, Perlman DC, Strauss SM, Mallow A, Hernandez D, Schackman BR, Feaster DJ, Metsch LR: On-site bundled rapid HIV/HCV testing in substance use disorder treatment programs: study protocol for a hybrid design randomized controlled trial. Trials 2016, 17(1):117.

430. Frimpong JA, D'Aunno T, Perlman DC, Strauss SM, Mallow A, Hernandez D, Schackman BR, Feaster DJ, Metsch LR: On-site bundled rapid HIV/HCV testing in substance use disorder treatment programs: study protocol for a hybrid design randomized controlled trial. Trials 2016, 17(1):117.

431. Fu J, Gavaghan A, Millett G, Walsh T: Replicating PEPFAR's success: How interventions shown to be effective abroad can be applied to the AIDS epidemic in the US. Health Affairs 2012, 31(7):1585-1592.

432. Fuller BE, Loftis JM, Rodriguez VL, McQuesten MJ, Hauser P: Psychiatric and substance use disorders comorbidities in veterans with hepatitis C virus and HIV coinfection. Current Opinion in Psychiatry 2009, 22(4):401-408.

433. Fuller BE, Loftis JM, Rodriguez VL, McQuesten MJ, Hauser P: Psychiatric and substance use disorders comorbidities in veterans with hepatitis C virus and HIV coinfection. Current Opinion in Psychiatry 2009, 22(4):401-408.

434. Fuller BE, Rodriguez VL, Linke A, Sikirica M, Dirani R, Hauser P: Prevalence of liver disease in veterans with bipolar disorder or schizophrenia. General Hospital Psychiatry 2011, 33(3):232-237.

435. Gallucci G, Smolinski J: Treatment contracts for patients with hepatitis C, psychiatric illness, and substance abuse. Psychosomatics 2001, 42(4):353-355.

436. Gardenier D, Andrews CM, Thomas DC, Bookhardt-Murray LJ, Fitzpatrick JJ: Social Support and Adherence: Differences Among Clients in an AIDS Day Health Care Program. Journal of the Association of Nurses in AIDS Care 2010, 21(1):75-85.

437. Gardenier D, Andrews CM, Thomas DC, Bookhardt-Murray LJ, Fitzpatrick JJ: Social support and adherence: differences among clients in an AIDS day health care program. Journal of the Association of Nurses in AIDS Care 2010, 21(1):75-85.

438. Gardenier D, Neushotz LA, O'Connor-Moore N: Medical/psychiatric comanagement by nurse practitioners in chronic hepatitis C treatment: a case study. Arch Psychiatr Nurs 2007, 21(2):87-90.

439. Gebo KA, Fleishman JA, Conviser R, Reilly ED, Korthuis PT, Moore RD, Hellinger J, Keiser P, Rubin HR, Crane L et al: Racial and gender disparities in receipt of highly active antiretroviral therapy persist in a multistate sample of HIV patients in 2001. J Acquir Immune Defic Syndr 2005, 38(1):96-103.

440. Gelberg L, Robertson MJ, Leake B, Wenzel SL, Bakhtiar L, Hardie EA, Sadler N, Getzug T: Hepatitis B among homeless and other impoverished US military veterans in residential care in Los Angeles. Public Health 2001, 115(4):286-291.

441. Gelkopf M, Weizman T, Melamed Y, Adelson M, Bleich A: Does psychiatric comorbidity affect drug abuse treatment outcome? A prospective assessment of drug abuse, treatment tenure and infectious diseases in an Israeli methadone maintenance clinic. Israel Journal of Psychiatry and Related Sciences 2006, 43(2):126-136.

442. Gelkopf M, Weizman T, Melamed Y, Adelson M, Bleich A: Does psychiatric comorbidity affect drug abuse treatment outcome? A prospective assessment of drug abuse, treatment tenure and

infectious diseases in an Israeli methadone maintenance clinic. Isr J Psychiatry Relat Sci 2006, 43(2):126-136.

443. Geppert CM, Arora S: Ethical issues in the treatment of hepatitis C. Clin Gastroenterol Hepatol 2005, 3(10):937-944.

444. Geppert CMA, Arora S: Ethical issues in the treatment of hepatitis C. Clinical Gastroenterology and Hepatology 2005, 3(10):937-944.

445. Getahun H, Baddeley A, Raviglione M: Managing tuberculosis in people who use and inject illicit drugs. Bulletin of the World Health Organization 2013, 91(2):154-156.

446. Getahun H, Gunneberg C, Sculier D, Verster A, Raviglione M: Tuberculosis and HIV in people who inject drugs: Evidence for action for tuberculosis, HIV, prison and harm reduction services. Current Opinion in HIV and AIDS 2012, 7(4):345-353.

447. Getahun H, Gunneberg C, Sculier D, Verster A, Raviglione M: Tuberculosis and HIV in people who inject drugs: evidence for action for tuberculosis, HIV, prison and harm reduction services. Curr Opin HIV AIDS 2012, 7(4):345-353.

448. Ghodse AH, McCartney J: Systems analysis of a drug dependency service in London: St George's Hospital. British Journal of Addiction 1992, 87(10):1377-1385.

449. Giang LM, Li L, Metzger D, Korthuis PT, Bart G: The cascade of care in Vietnam: From communities to Codons. Journal of Neuroimmune Pharmacology 2017, 12 (2 Supplement 1):S93-S94.

450. Gilbert L, Hunt T, Primbetova S, Terlikbayeva A, Chang M, Wu E, McCrimmon T, El-Bassel N: Reducing opioid overdose in Kazakhstan: A randomized controlled trial of a couple-based integrated HIV/HCV and overdose prevention intervention "Renaissance". International Journal of Drug Policy 2018, 54:105-113.

451. Gilbert L, Primbetova S, Nikitin D, Hunt T, Terlikbayeva A, Momenghalibaf A, Ruziev M, El-Bassel N: Redressing the epidemics of opioid overdose and HIV among people who inject drugs in Central Asia: The need for a syndemic approach. Drug and Alcohol Dependence 2013, 132(SUPPL1):S56-S60.

452. Gimeno-Gracia M, Crusells-Canales MJ, Rabanaque-Hernandez MJ: Clinical characteristics and antiretroviral treatment of older HIV-infected patients. International Journal of Clinical Pharmacy 2014, 36(6):1190-1195.

453. Ginsberg GM, Shinar E, Kopel E, Chemtob D: Should Men who have sex with Men be allowed to donate blood in Israel. Israel Journal of Health Policy Research 2016, 5 (1) (no pagination)(60).

454. Giordano TP, Visnegarwala F, White AC, Jr., Troisi CL, Frankowski RF, Hartman CM, Grimes RM: Patients referred to an urban HIV clinic frequently fail to establish care: factors predicting failure. AIDS Care 2005, 17(6):773-783.

455. Gish RG, Bui TD, Nguyen CT, Nguyen DT, Tran HV, Tran DM, Trinh HN, International Group for Liver Health in Viet N: Liver disease in Viet Nam: screening, surveillance, management and education: a 5-year plan and call to action. J Gastroenterol Hepatol 2012, 27(2):238-247.

456. Glasner S, Moore A, Lake J, Reid M, Gomez L, Jenkins J, Dominguez B, Candelario J: Changes in alcohol use among older HIV+ adults in response to a technology-assisted integrated CBT

intervention combined with tai chi. Alcoholism: Clinical and Experimental Research 2017, 41 (Supplement 1):91A.

457. Gleason O, Fucci J, Yates W: Gastroenterologists' perceptions of need and availability of psychiatric services for patients with hepatitis C. Psychosomatics 2008, 49(2):132-136.

458. Goforth HW, Lupash DP, Brown ME, Tan J, Fernandez F: Role of alcohol and substances of abuse in the immunomodulation of human immunodeficiency virus disease: A review. Addictive Disorders and their Treatment 2004, 3(4):174-182.

459. Gold J, High HA, Li Y, Michelmore H, Bodsworth NJ, Finlayson R, Furner VL, Allen BJ, Oliver CJ: Safety and efficacy of nandrolone decanoate for treatment of wasting in patients with HIV infection. Aids 1996, 10(7):745-752.

460. Golz J: Ambulatory medical care of drug addicts. [German]. Zeitschrift fur Arztliche Fortbildung 1996, 90(4):271-278.

461. Golz J: [Ambulatory medical treatment of drug dependent patients]. Z Arztl Fortbild (Jena) 1996, 90(4):271-278.

462. Gomez MF, Klein DA, Sand S, Marconi M, O'Dowd MA: Delivering mental health care to HIV-positive individuals. A comparison of two models. Psychosomatics 1999, 40(4):321-324.

463. Gomez MP, Kimball AM, Orlander H, Bain RM, Fisher LD, Holmes KK: Epidemic crack cocaine use linked with epidemics of genital ulcer disease and heterosexual HIV infection in the Bahamas: evidence of impact of prevention and control measures. Sex Transm Dis 2002, 29(5):259-264.

464. Gomez-Batiste X, Porta-Sales J, Pascual A, Nabal M, Espinosa J, Paz S, Minguell C, Rodriguez D, Esperalba J, Stjernsward J et al: Catalonia WHO palliative care demonstration project at 15 Years (2005). J Pain Symptom Manage 2007, 33(5):584-590.

465. Gonzales R, Marinelli-Casey P, Shoptaw S, Ang A, Rawson RA: Hepatitis C virus infection among methamphetamine-dependent individuals in outpatient treatment. Journal of Substance Abuse Treatment 2006, 31(2):195-202.

466. Gonzales R, Perlman DC: Response: a case for collaborative care. Addiction science & clinical practice 2007, 4(1):43-44.

467. Gonzales R, Perlman DC: Response: a case for collaborative care. Addiction Science & Clinical Practice 2007, 4(1):43-44.

468. Gonzalez HV, Casillas-Rodriguez J, Allain NG: HIV treatment cascade in HIV incarcerated patients in Mexico City 2014. Journal of the International AIDS Society 2015, 18:11-12.

469. Gonzalez SA, Fierer DS, Talal AH: Medical and behavioral approaches to engage people who inject drugs into care for Hepatitis C Virus infection. Addictive Disorders and their Treatment 2017, 16(2 Supplement 1):S1-S23.

470. Gordon D: CDC advises on integrating management of HIV, other STDs, and illicit drug use. AIDS Reader 2012, 14.

471. Gordon MS, Vocci FJ, Fitzgerald TT, O'Grady KE, O'Brien CP: Extended-release naltrexone for pre-release prisoners: A randomized trial of medical mobile treatment. Contemporary Clinical Trials 2017, 53:130-136.

472. Gottheil E, Lundy A, Weinstein SP, Sterling RC: Does intensive outpatient cocaine treatment reduce AIDS risky behaviors? Journal of Addictive Diseases 1998, 17(4):61-69.

473. Grady BP, Vanhommerig JW, Schinkel J, Weegink CJ, Bruisten SM, Lindenburg CE, Prins M: Low incidence of reinfection with the hepatitis C virus following treatment in active drug users in Amsterdam. Eur J Gastroenterol Hepatol 2012, 24(11):1302-1307.

474. Grando-Lemaire V, Goisset P, Sorge F, Trinchet JC, Castera L, Roulot D, Sitruk V, Beaugrand M: [Hepatitis C virus screening in drug users in an addiction out-patient unit]. Gastroenterol Clin Biol 2002, 26(12):1091-1096.

475. Grange F, Levin B, Pellenq E, Haegy JM, Guillaume JC: Dermatological consultation behind bars: An analysis on a three-year period in a French prison. [French]. Annales de Dermatologie et de Venereologie 2001, 128(4):513-516.

476. Grant L, Leng M, Murray SA: Palliative care making a difference in Africa: A rapid evaluation methods study. Palliative Medicine 2010, 1):S226-S227.

477. Grassi A, Ballardini G: Hepatitis C in injection drug users: It is time to treat. World Journal of Gastroenterology 2017, 23(20):3569-3571.

478. Grau L, Colombotos J, Gorman S: Psychological morale and job satisfaction among homecare workers who care for persons with AIDS. Women and Health 1992, 18(1):1-21.

479. Grau L, Colombotos J, Gorman S: Psychological morale and job satisfaction among homecare workers who care for persons with AIDS. Women Health 1992, 18(1):1-21.

480. Grebely J, Alavi M, Micallef M, Dunlop AJ, Balcomb AC, Phung N, Weltman MD, Day CA, Treloar C, Bath N et al: Treatment for hepatitis C virus infection among people who inject drugs attending opioid substitution treatment and community health clinics: the ETHOS Study. Addiction (Abingdon, England) 2016, 111(2):311-319.

481. Grebely J, Alavi M, Micallef M, Dunlop AJ, Balcomb AC, Phung N, Weltman MD, Day CA, Treloar C, Bath N et al: Treatment for hepatitis C virus infection among people who inject drugs attending opioid substitution treatment and community health clinics: the ETHOS Study. Addiction 2016, 111(2):311-319.

482. Grebely J, Bruggmann P, Treloar C, Byrne J, Rhodes T, Dore GJ: Expanding access to prevention, care and treatment for hepatitis C virus infection among people who inject drugs. International Journal of Drug Policy 2015, 26(10):893-898.

483. Grebely J, deVlaming S, Duncan F, Viljoen M, Conway B: Current approaches to HCV infection in current and former injection drug users. Journal of Addictive Diseases 2008, 27(2):25-35.

484. Grebely J, Genoway K, Khara M, Duncan F, Viljoen M, Elliott D, Raffa JD, DeVlaming S, Conway B: Treatment uptake and outcomes among current and former injection drug users receiving directly observed therapy within a multidisciplinary group model for the treatment of hepatitis C virus infection. International Journal of Drug Policy 2007, 18(5):437-443.

485. Grebely J, Knight E, Genoway KA, Viljoen M, Khara M, Elliott D, Gallagher L, Storms M, Raffa JD, DeVlaming S et al: Optimizing assessment and treatment for hepatitis C virus infection in illicit drug users: a novel model incorporating multidisciplinary care and peer support. Eur J Gastroenterol Hepatol 2010, 22(3):270-277.

486. Grebely J, Tyndall MW: Management of HCV and HIV infections among people who inject drugs. Current Opinion in HIV and AIDS 2011, 6(6):501-507.

487. Grebely J, Tyndall MW: Management of HCV and HIV infections among people who inject drugs. Curr Opin HIV AIDS 2011, 6(6):501-507.

488. Grebely J, Tyndall MW: Management of HCV and HIV infections among people who inject drugs. Curr Opin HIV AIDS 2011, 6(6):501-507.

489. Grebely J, Tyndall MW: Management of HCV and HIV infections among people who inject drugs. Curr Opin HIV AIDS 2011, 6(6):501-507.

490. Greco R, Lavack L, Rovers J: Assessment of the pharmacy service needs of HIV-positive outpatients receiving zidovudine. Ann Pharmacother 1992, 26(5):621-626.

491. Green K, Tuan T, Hoang TV, Thi Trang NN, Thanh Ha NT, Hung ND: Integrating palliative care into HIV outpatient clinical settings: Preliminary findings from an intervention study in vietnam. Journal of Pain and Symptom Management 2010, 40(1):31-34.

492. Greenfield SF, Shields A, Connery HS, Livchits V, Yanov SA, Lastimoso CS, Strelis AK, Mishustin SP, Fitzmaurice G, Mathew TA et al: Integrated management of physician-delivered alcohol care for tuberculosis patients: Design and implementation. Alcoholism: Clinical and Experimental Research 2010, 34(2):317-330.

493. Greenfield SF, Shields A, Connery HS, Livchits V, Yanov SA, Lastimoso CS, Strelis AK, Mishustin SP, Fitzmaurice G, Mathew TA et al: Integrated Management of Physician-delivered Alcohol Care for Tuberculosis Patients: Design and Implementation. Alcohol Clin Exp Res 2010, 34(2):317-330.

494. Greenwald JL, Rich CA, Bessega S, Posner MA, Maeda JL, Skolnik PR: Evaluation of the Centers for Disease Control and Prevention's recommendations regarding routine testing for human immunodeficiency virus by an inpatient service: Who are we missing? Mayo Clinic Proceedings 2006, 81(4):452-458.

495. Greenwood J: Persuading general practitioners to prescribe - Good husbandry or a recipe for chaos? British Journal of Addiction 1992, 87(4):567-575.

496. Greenwood J: Persuading general practitioners to prescribe--good husbandry or a recipe for chaos? British Journal of Addiction 1992, 87(4):567-575.

497. Greenwood J: Six years' experience of sharing the care of Edinburgh's drug users. Psychiatric Bulletin 1996, 20(1):8-11.

498. Grenfell P, Baptista Leite R, Garfein R, de Lussigny S, Platt L, Rhodes T: Tuberculosis, injecting drug use and integrated HIV-TB care: A review of the literature. Drug and Alcohol Dependence 2013, 129(3):180-209.

499. Grenfell P, Baptista Leite R, Garfein R, de Lussigny S, Platt L, Rhodes T: Tuberculosis, injecting drug use and integrated HIV-TB care: a review of the literature. Drug Alcohol Depend 2013, 129(3):180-209.

500. Grenfell P, Baptista Leite R, Garfein R, de Lussigny S, Platt L, Rhodes T: Tuberculosis, injecting drug use and integrated HIV-TB care: a review of the literature. Drug Alcohol Depend 2013, 129(3):180-209.

501. Grenfell P, Baptista Leite R, Garfein R, de Lussigny S, Platt L, Rhodes T: Tuberculosis, injecting drug use and integrated HIV-TB care: a review of the literature. Drug Alcohol Depend 2013, 129(3):180-209.

502. Groessl EJ, Liu L, Sklar M, Ho SB: HCV Integrated Care: A Randomized Trial to Increase Treatment Initiation and SVR with Direct Acting Antivirals. International Journal of Hepatology 2017, 2017 (no pagination)(5834182).

503. Groessl EJ, Liu L, Sklar M, Ho SB: HCV Integrated Care: A Randomized Trial to Increase Treatment Initiation and SVR with Direct Acting Antivirals. International Journal of Hepatology 2017, 2017:5834182.

504. Groessl EJ, Sklar M, Cheung RC, Brau N, Ho SB: Increasing antiviral treatment through integrated hepatitis C care: A randomized multicenter trial. Contemporary Clinical Trials 2013, 35(2):97-107.

505. Groessl EJ, Sklar M, Cheung RC, Brau N, Ho SB: Increasing antiviral treatment through integrated hepatitis C care: a randomized multicenter trial. Contemporary Clinical Trials 2013, 35(2):97-107.

506. Guerrero EG, Aarons GA, Palinkas LA: Organizational capacity for service integration in community-based addiction health services. American Journal of Public Health 2014, 104(4):e40-47.

507. Gugelmann RJ, Freed GL, Desgrandchamps D, Diebold P: [Hepatitis B vaccination: knowledge and acceptance by Swiss physicians]. Soz Praventivmed 1998, 43 Suppl 1:S57-60, S130-133.

508. Guindalini C, O'Gara C, Laranjeira R, Collier D, Castelo A, Vallada H, Breen G: A GSTP1 functional variant associated with cocaine dependence in a Brazilian population. Pharmacogenet Genomics 2005, 15(12):891-893.

509. Guise A, Rhodes T, Ndimbii J, Ayon S, Nnaji O: Access to HIV treatment and care for people who inject drugs in Kenya: a short report. AIDS Care 2016, 28(12):1595-1599.

510. Guise A, Seguin M, Mburu G, McLean S, Grenfell P, Islam Z, Filippovych S, Assan H, Low A, Vickerman P et al: Integrated opioid substitution therapy and HIV care: a qualitative systematic review and synthesis of client and provider experiences. AIDS Care - Psychological and Socio-Medical Aspects of AIDS/HIV 2017, 29(9):1119-1128.

511. Guise A, Seguin M, Mburu G, McLean S, Grenfell P, Islam Z, Filippovych S, Assan H, Low A, Vickerman P et al: Integrated opioid substitution therapy and HIV care: a qualitative systematic review and synthesis of client and provider experiences. AIDS Care 2017, 29(9):1119-1128.

512. Gunn RA, Lee MA, Callahan DB, Gonzales P, Murray PJ, Margolis HS: Integrating hepatitis, STD, and HIV services into a drug rehabilitation program. Am J Prev Med 2005, 29(1):27-33.

513. Habrat B, Chmielewska K, Baran-Furga H, Keszycka B, Taracha E: Subjective Quality of Life in opiate-dependent patients before admission after six months and one-year participation in methadone program. [Polish]. Przeglad lekarski 2002, 59(4-5):351-354.

514. Habrat B, Chmielewska K, Baran-Furga H, Keszycka B, Taracha E: [Subjective Quality of Life in opiate-dependent patients before admission after six months and one-year participation in methadone program]. Przeglad Lekarski 2002, 59(4-5):351-354.

515. Hafkin J, Modongo C, Newcomb C, Lowenthal E, MacGregor RR, Steenhoff A, Friedman H, Bisson G: Impact of HIV on early MDR-TB treatment outcomes in Botswana. Journal of the International AIDS Society 2012, 15:43-44.

516. Hagan LM, Schinazi RF: Best strategies for global HCV eradication. Liver Int 2013, 33 Suppl 1:68-79.

517. Hagman G: Methadone maintenance counseling. Definition, principles, components. Journal of Substance Abuse Treatment 1994, 11(5):405-413.

518. Hahm HC, Chang ST, Lee GY, Tagerman MD, Lee CS, Trentadue MP, Hien DA: Asian Women's Action for Resilience and Empowerment Intervention: Stage I Pilot Study. J Cross Cult Psychol 2017, 48(10):1537-1553.

519. Haines CF, Fleishman JA, Yehia BR, Lau B, Berry SA, Agwu AL, Moore RD, Gebo KA: Closing the Gap in Antiretroviral Initiation and Viral Suppression: Time Trends and Racial Disparities. J Acquir Immune Defic Syndr 2016, 73(3):340-347.

520. Hajarizadeh B, Grebely J, Dore GJ: Epidemiology and natural history of HCV infection. Nature Reviews Gastroenterology and Hepatology 2013, 10(9):553-562.

521. Haldane V, Cervero-Liceras F, Chuah FL, Ong SE, Murphy G, Sigfrid L, Watt N, Balabanova D, Hogarth S, Maimaris W et al: Integrating HIV and substance use services: A systematic review: A. Journal of the International AIDS Society 2017, 20 (1) (no pagination)(21585).

522. Haldane V, Cervero-Liceras F, Chuah FL, Ong SE, Murphy G, Sigfrid L, Watt N, Balabanova D, Hogarth S, Maimaris W et al: Integrating HIV and substance use services: a systematic review. Journal of the International AIDS Society 2017, 20(1):21585.

523. Haley SJ, Kreek MJ: A window of opportunity: maximizing the effectiveness of new HCV regimens in the United States with the expansion of the Affordable Care Act. American journal of public health 2015, 105(3):457-463.

524. Haley SJ, Kreek MJ: A window of opportunity: maximizing the effectiveness of new HCV regimens in the United States with the expansion of the Affordable Care Act. American Journal of Public Health 2015, 105(3):457-463.

525. Hall YN, Choi AI, Himmelfarb J, Chertow GM, Bindman AB: Homelessness and CKD: A cohort study. Clinical Journal of the American Society of Nephrology 2012, 7(7):1094-1102.

526. Hallinan R, Byrne A, Dore GJ: Harm reduction, hepatitis C and opioid pharmacotherapy: An opportunity for integrated hepatitis C virus-specific harm reduction. Drug and Alcohol Review 2007, 26(4):437-443.

527. Hallinan R, Byrne A, Dore GJ: Harm reduction, hepatitis C and opioid pharmacotherapy: an opportunity for integrated hepatitis C virus-specific harm reduction. Drug Alcohol Rev 2007, 26(4):437-443.

528. Halota W, Opoka J, Topczewska E, Trzcinski J, Inczyk M, Apanasiewicz M, Gizinski W: Organization of health care for persons from the groups of high risk of HIV infection in Bydgoszcz. [Polish]. Przeglad epidemiologiczny 1990, 44(4):351-356.

529. Halota W, Opoka J, Topczewska E, Trzcinski J, Inczyk M, Apanasiewicz M, Gizinski W: [Organization of health care for persons from the groups of high risk of HIV infection in Bydgoszcz]. Przeglad Epidemiologiczny 1990, 44(4):351-356.

530. Hammig R: Draft guiding principles for regulations governing the use of agonist medication in the treatment of opioid dependence. Heroin Addiction and Related Clinical Problems 2016, 18 (3 Supplement 1):34.

531. Hampton T: HIV study shines spotlight on women. JAMA - Journal of the American Medical Association 2010, 304(3):257-258.

532. Hanouneh IA, Zein NN, Cikach F, Alkhouri N, Lopez R, Dweik RA: A novel non-invasive breath test in patients with acute alcoholic hepatitis. Gastroenterology 2013, 1):S446-S447.

533. Hanselmann H, Hanselmann S: [Tuberculous meningitis]. Psychother Psychosom Med Psychol 1996, 46(8):318.

534. Haque L, Jakab S: Substance use disorders in recently hospitalized patients with cirrhosis: Insights from a transitional care clinic. American Journal of Gastroenterology 2016, 111 (Supplement 1):S391.

535. Harford JB: Palliative care in low-income and middle-income countries: The need is great, but the services are few. Journal of Pediatric Hematology/Oncology 2010, 32 (5):419-420.

536. Harries AD: Management of HIV in resource-poor countries, with a focus on sub-Saharan Africa. Leprosy Review 2002, 73(3):268-275.

537. Harris HW, Young DM: Care of injection drug users with soft tissue infections in San Francisco, California. Arch Surg 2002, 137(11):1217-1222.

538. Harris KA, Jr., Arnsten JH, Litwin AH: Successful integration of hepatitis C evaluation and treatment services with methadone maintenance. Journal of Addiction Medicine 2010, 4(1):20-26.

539. Harris SN, Mowbray CT, Solarz A: Physical health, mental health, and substance abuse problems of shelter users. Health & Social Work 1994, 19(1):37-45.

540. Hart TA, Tulloch TG, O'Cleirigh C: Integrated HIV prevention and mental health counselling for social anxiety for HIV-negative gay and bisexual men. Canadian Journal of Infectious Diseases and Medical Microbiology 2013, SA):134A.

541. Hartzler B, Carlini BH, Newville H, Crane HM, Eron JJ, Geng EH, Mathews WC, Mayer KH, Moore RD, Mugavero MJ et al: Identifying HIV care enrollees at-risk for cannabis use disorder. AIDS Care - Psychological and Socio-Medical Aspects of AIDS/HIV 2017, 29(7):846-850.

542. Hartzler B, Carlini BH, Newville H, Crane HM, Eron JJ, Geng EH, Mathews WC, Mayer KH, Moore RD, Mugavero MJ et al: Identifying HIV care enrollees at-risk for cannabis use disorder. AIDS Care 2017, 29(7):846-850.

543. Hartzler B, Donovan D, Beadnell B, Crane HM, Eron JJ, Geng EH, Matthews WC, Mayer KH, Moore RD, Mugavero M et al: Domestic prevalence of substance use disorders in HIV care settings. Drug and Alcohol Dependence 2017, 171:e84.

544. Hasker E, Khodjikhanov M, Usarova S, Asamidinov U, Yuldashova U, van der Werf MJ, Uzakova G, Veen J: Default from tuberculosis treatment in Tashkent, Uzbekistan; Who are these defaulters and why do they default? BMC Infectious Diseases 2008, 8 (no pagination)(97).

545. Haug NA, Padula CB, Sottile JE, Vandrey R, Heinz AJ, Bonn-Miller MO: Cannabis use patterns and motives: A comparison of younger, middle-aged, and older medical cannabis dispensary patients. Addict Behav 2017, 72:14-20.

546. Hawks LC, Norton BL, Cunningham C, Fox AD: The hepatitis c virus cascade at an urban postin carceration transitions clinic. Journal of General Internal Medicine 2015, 30:S273.

547. Hayward P: First reduce harm: tackling HIV in Ukraine. Lancet 2010, 376(9749):1287.

548. Hazeghazam MH, Gesmundo CK, Ramos GM, James WS: Identification of behavioral and sleep problems in dementia patients admitted to maricopa integrated health system (mihs) from jan 2013 to june 2014. American Journal of Geriatric Psychiatry 2015, 1):S145.

549. Hearst A, Heffner J: Public responsibility and home tests for HIV. AIDS policy & law 1996, 11(5):8-9.

550. Hearst A, Heffner J: Public responsibility and home tests for HIV. AIDS Policy & Law 1996, 11(5):8-9.

551. Heinz AJ, Fogler KA, Newcomb ME, Trafton JA, Bonn-Miller MO: Problematic alcohol use among individuals with HIV: relations with everyday memory functioning and HIV symptom severity. Aids Behav 2014, 18(7):1302-1314.

552. Heller D, McCoy K, Cunningham C: An invisible barrier to integrating HIV primary care with harm reduction services: philosophical clashes between the harm reduction and medical models. Public Health Reports 2004, 119(1):32-39.

553. Hellinger FJ: The use of health services by women with HIV infection. Health Services Research 1993, 28(5):543-561.

554. Henrickson M: A mobile HIV education, counseling, and testing unit: A pilot initiative. AIDS Education and Prevention 1990, 2(2):137-144.

555. Henry K: Management of HIV infection. A 1995-96 overview for the clinician. Minn Med 1995, 78(11):17-24.

556. Henry NK, Hoecker JL, Rhodes KH: Antimicrobial therapy for infants and children: guidelines for the inpatient and outpatient practice of pediatriac infectious diseases. Mayo Clinic Proceedings 2000, 75(1):86-97.

557. Henry PC, Rich JD: Editorial comment: the complexities of homelessness and HIV. AIDS Reader 2005, 15(11):613.

558. Herce ME, Elmore SN, Kalanga N, Keck JW, Wroe EB, Phiri A, Mayfield A, Chingoli F, Beste JA, Tengatenga L et al: Assessing and responding to palliative care needs in rural sub-Saharan Africa: Results from a model intervention and situation analysis in Malawi. PLoS ONE 2014, 9 (10) (no pagination)(e110457).

559. Herman M, Gourevitch MN: Integrating primary care and methadone maintenance treatment: Implementation issues. Journal of Addictive Diseases 1997, 16(1):91-102.

560. Hermos JA, Young MM, Gagnon DR, Fiore LD: Characterizations of long-term oxycodone/acetaminophen prescriptions in veteran patients. Archives of Internal Medicine 2004, 164(21):2361-2366.

561. Herrmann ES, Matusiewicz AK, Stitzer ML, Higgins ST, Sigmon SC, Heil SH: Contingency Management Interventions for HIV, Tuberculosis, and Hepatitis Control Among Individuals With Substance Use Disorders: A Systematized Review. J Subst Abuse Treat 2017, 72:117-125.

562. Herrmann ES, Matusiewicz AK, Stitzer ML, Higgins ST, Sigmon SC, Heil SH: Contingency Management Interventions for HIV, Tuberculosis, and Hepatitis Control Among Individuals With Substance Use Disorders: A Systematized Review. J Subst Abuse Treat 2017, 72:117-125.

563. Hetrick G, Humarau H: [Treatment modalities applied to tuberculosis patients in a French department. Hautes-Pyrenees, 1973 to 1980]. Rev Fr Mal Respir 1983, 11(6):883-887.

564. Hiatt RA, Quesenberry CP, Jr., Selby JV, Fireman BH, Knight A: The cost of acquired immunodeficiency syndrome in northern California. The experience of a large prepaid health plan. Archives of Internal Medicine 1990, 150(4):833-838.

565. Higgins NH, Gauthier J, Wong L, Potter M, Lalonde RG, Klein MB: Description of factors associated with medication errors in an hiv ambulatory care setting: a pilot study (defeat study). Canadian Journal of Infectious Diseases and Medical Microbiology 2010, SB):61B-62B.

566. Hilsden RJ, Macphail G, Grebely J, Conway B, Lee SS: Directly observed pegylated interferon plus self-administered ribavirin for the treatment of hepatitis C virus infection in people actively using drugs: a randomized controlled trial. Clin Infect Dis 2013, 57 Suppl 2:S90-96.

567. Hilsden RJ, Macphail G, Grebely J, Conway B, Lee SS: Directly observed pegylated interferon plus self-administered ribavirin for the treatment of hepatitis C virus infection in people actively using drugs: a randomized controlled trial. Clin Infect Dis 2013, 57 Suppl 2:S90-96.

568. Hirnschall G, Harries AD, Easterbrook PJ, Doherty MC, Ball A: The next generation of the World Health Organization's global antiretroviral guidance. Journal of the International AIDS Society 2013, 16 (no pagination)(18757).

569. Hirschel B: New antiviral drugs: Progress and problems. [French]. Medecine et Hygiene 2001, 59(2341):776-778.

570. Hiv/Aids Treatment Adherence HO, Cost Study G: The HIV/AIDS Treatment Adherence, Health Outcomes and Cost Study: conceptual foundations and overview. AIDS Care 2004, 16 Suppl 1:S6-21.

571. Ho SB, Brau N, Cheung R, Liu L, Sanchez C, Sklar M, Phelps TE, Marcus SG, Wasil MM, Tisi A et al: Integrated Care Increases Treatment and Improves Outcomes of Patients With Chronic Hepatitis C Virus Infection and Psychiatric Illness or Substance Abuse. Clinical Gastroenterology and Hepatology 2015, 13(11):2005-2014.e2003.

572. Ho SB, Groessl E, Dollarhide A, Robinson S, Kravetz D, Dieperink E: Management of chronic hepatitis c in veterans: The potential of integrated care models. American Journal of Gastroenterology 2008, 103(7):1810-1823.

573. Ho SB, Groessl EJ, Brau N, Cheung R, Sanchez CM, Campbell N, Sklar M, Liu L, Phelps TE, Marcus S et al: Integrated care for high risk psychiatric and substance use disorder patients with hepatitis C increases overall SVR rates: Final results of a prospective multisite randomized trial. Hepatology 2013, 1):1295A.

574. Ho SB, Groessl EJ, Brau N, Cheung R, Weingart KR, Ward M, Sklar M, Phelps TE, Marcus S, Wasil MM et al: Multisite randomized trial of an Integrated Care (IC) model for HCV patients with

psychiatric and substance use co-morbidities: Final results of impact on treatment initiation. Hepatology 2012, 56:1000A-1001A.

575. Ho SB, Groessl EJ, Brau N, Cheung RC, Weingart KR, Ward MA, Sklar M, Phelps TE, Marcus SG, Wasil MM et al: Prospective multisite randomized trial of Integrated Care (IC) vs. Usual Care (UC) for improving access to antiviral therapy for high risk patients with chronic HCV. Journal of Hepatology 2012, 56:S386.

576. Ho SB, Groessl EJ, Liu L, Sanchez CM, Wasil MM, Robinson SK: Randomized trial of integrated care to promote direct acting antiviral treatment among high risk hepatitis c patients with psychiatric and substance use disorders. Journal of Hepatology 2014, 1):S333.

577. Ho SB, Groessl EJ, Weingart KR, Ward M, Sklar M, Robinson S, Johnson N, Phelps TE, Marcus S, Cheung R et al: Prospective multicenter evaluation of hepatitis C patients for psychiatric/substance use risk factors that reduce likelihood of antiviral treatment. Hepatology 2011, 54:1194A-1195A.

578. Hoffman HL, Castro-Donlan CA, Johnson VM, Church DR: The Massachusetts HIV, hepatitis, addiction services integration (HHASI) experience: responding to the comprehensive needs of individuals with co-occurring risks and conditions. Public Health Reports 2004, 119(1):25-31.

579. Holmberg SD, Moorman AC, Rupp LB, Lu M, Spradling PR, Teshale EH, Nerenz DR, Boscarino JA, Nakasato C, Naleway A et al: The chronic hepatitis cohort study ('checs'): Methods: and population characteristics. Hepatology 2010, 52:694A-695A.

580. Honarvar B, Lankarani KB, Odoomi N, Roudgari A, Moghadami M, Kazerooni PA, Abadi AH: Pulmonary and latent tuberculosis screening in opiate drug users: an essential and neglected approach for harm-reduction facilities. J Addict Med 2013, 7(4):230-235.

581. Hong BA, Berger SG: Characteristics of individuals using different HIV/AIDS counseling and testing programs. AIDS 1994, 8(2):259-262.

582. Hope V, Kimber J, Vickerman P, Hickman M, Ncube F: Frequency, factors and costs associated with injection site infections: findings from a national multi-site survey of injecting drug users in England. BMC Infectious Diseases 2008, 8:120.

583. Horberg MA, Hurley LB, Silverberg MJ, Klein DB, Quesenberry CP, Mugavero MJ: Missed office visits and risk of mortality among hiv-infected subjects in a large healthcare system in the United States. AIDS Patient Care and STDs 2013, 27(8):442-449.

584. Horcajada JP, Garcia L, Benito N, Cervera C, Sala M, Olivera A, Soriano A, Robau M, Gatell JM, Miro JM: Specialized home care for infectious disease. Experience from 1995 to 2002. [Spanish]. Enfermedades Infecciosas y Microbiologia Clinica 2007, 25(7):429-436.

585. Horcajada JP, Garcia L, Benito N, Cervera C, Sala M, Olivera A, Soriano A, Robau M, Gatell JM, Miro JM: [Specialized home care for infectious disease. Experience from 1995 to 2002]. Enfermedades Infecciosas y Microbiologia Clinica 2007, 25(7):429-436.

586. Horsfall L, MacDonald G, Scott I, Skoien R, Khatun M, Moss C, Seligman C, Kardash C, Poxon V, Powell EE: Use of standardised assessment forms in referrals to hepatology outpatient services: Implications for accurate triaging of patients with chronic hepatitis C. Australian Health Review 2013, 37(2):218-222.

587. Houtrow A, Carle A, Stein R, Perrin J: Health service needs for children with disabilities on supplemental security income. Archives of Physical Medicine and Rehabilitation 2017, 98 (10):e15.

588. Howard BN, Van Dorn R, Myers BJ, Zule WA, Browne FA, Carney T, Wechsberg WM: Barriers and facilitators to implementing an evidence-based woman-focused intervention in South African health services. BMC health services research 2017, 17(1):746.

589. Hoyos J, de la Fuente L, Fernandez S, Gutierrez J, Rosales ME, Garcia de Olalla P, Ruiz M, Belza MJ, Grupo de Pruebas Rapidas de VIHM: [Street outreach rapid HIV testing in university settings: a priority strategy?]. Gac Sanit 2012, 26(2):131-137.

590. Hsieh J, Banker S, Khokhar A: Why are we not treating enough hepatitis C? Gastroenterology 2013, 1):S219.

591. Huber M, Weber R, Oppliger R, Vernazza P, Schmid P, Schonbucher P, Bertisch B, Meili D, Renner EL: Interferon alpha-2a plus ribavirin 1,000/1,200 mg versus interferon alpha-2a plus ribavirin 600 mg for chronic hepatitis C infection in patients on opiate maintenance treatment: an open-label randomized multicenter trial. Infection 2005, 33(1):25-29.

592. Huber M, Weber R, Oppliger R, Vernazza P, Schmid P, Schonbucher P, Bertisch B, Meili D, Renner EL: Interferon alpha-2a plus ribavirin 1,000/1,200 mg versus interferon alpha-2a plus ribavirin 600 mg for chronic hepatitis C infection in patients on opiate maintenance treatment: an open-label randomized multicenter trial. Infection 2005, 33(1):25-29.

593. Huckans MS, Blackwell AD, Harms TA, Indest DW, Hauser P: Integrated hepatitis C virus treatment: Addressing comorbid substance use disorders and HIV infection. Aids 2005, 19(SUPPL. 3):S106-S115.

594. Huckans MS, Loftis JM, Blackwell AD, Linke A, Hauser P: Interferon alpha therapy for hepatitis C: treatment completion and response rates among patients with substance use disorders. Substance abuse treatment, prevention, and policy 2007, 2:4.

595. Hung V, Nguyen ST, Tieu VTT, Nguyen TTT, Duong TH, Lyss S, Oeltmann JE: Evaluation of the integrated clinic model for HIV/AIDS services in Ho Chi Minh City, Viet Nam, 2013-2014. Public Health Action 2016, 6(4):255-260.

596. Hurley PM, Ungvarski PJ: Home healthcare needs of adults living with HIV disease/AIDS in New York City. The Journal of the Association of Nurses in AIDS Care : JANAC 1994, 5(2):33-40.

597. Ignazio G, Ubaldi E, Portincasa P, Palasciano G: Liver disease: Early signs you may be missing. Journal of Family Practice 2009, 58(10):514-521.

598. Ingersoll KS, Farrell-Carnahan L, Cohen-Filipic J, Heckman CJ, Ceperich SD, Hettema J, Marzani-Nissen G: A pilot randomized clinical trial of two medication adherence and drug use interventions for HIV+ crack cocaine users. Drug Alcohol Depend 2011, 116(1-3):177-187.

599. Ingersoll KS, Farrell-Carnahan L, Cohen-Filipic J, Heckman CJ, Ceperich SD, Hettema J, Marzani-Nissen G: A pilot randomized clinical trial of two medication adherence and drug use interventions for HIV+ crack cocaine users. Drug Alcohol Depend 2011, 116(1-3):177-187.

600. Irvin R, McAdams-Mahmoud A, Hickman D, Wilson J, Fenwick W, Chen I, Irvin N, Falade-Nwulia O, Sulkowski M, Chaisson R et al: Building a Community - Academic Partnership to Enhance Hepatitis C Virus Screening. J Community Med Health Educ 2016, 6(3).

601. Islam MM, Grummett S, White A, Reid SE, Day CA, Haber PS: A primary healthcare clinic in a needle syringe program may contribute to HIV prevention by early detection of incident HIV in an injecting drug user. Australian and New Zealand journal of public health 2011, 35(3):294-295.

602. Islam MM, Topp L, Conigrave KM, White A, Reid SE, Grummett S, Haber PS, Day CA: Linkage into specialist hepatitis C treatment services of injecting drug users attending a needle syringe program-based primary healthcare centre. Journal of Substance Abuse Treatment 2012, 43(4):440-445.

603. Jack K, Varnam M, Thomson B: Injecting drug users. Br J Gen Pract 2005, 55(515):473.

604. Jacob D, Rihet P, Siouffi P, Ravaux I, Bongrand MC, Timond-David P, Sambuc R, Durand JM: Home care and AIDS: 4 years of experience in public Hospital of Marseille. [French]. Pharmacie Hospitaliere Francaise 1997(122):133-141.

605. Jacobs JL, Damson LC, Rogers DE: One approach to care for patients infected with human immunodeficiency virus in an academic medical center. Bulletin of the New York Academy of Medicine 1996, 73(2):301-313.

606. Jaffe MP, O'Neill J, Vandergoot D, Gordon WA, Small B: The unveiling of traumatic brain injury in an HIV/AIDS population. Brain Inj 2000, 14(1):35-44.

607. James H, Imhof R, Sahoo A, Montenegro M, Imhof N, Gonzalez C, Chang AY, Lteif AN, Davidge-Pitts CJ, Nippoldt TB: A community-based study of demographics and associated medical and psychiatric conditions of transgender individuals from the rochester epidemiology project. Endocrine Reviews Conference: 100th Annual Meeting of the Endocrine Society, ENDO 2018, 39(2 Supplement 1).

608. Janjua NZ, Darvishian M, Chong MY, Ramji A, Yoshida EM, Islam N, Butt Z, Cook D, Chapinal N, Alvarez M et al: Real-world effectiveness of sofosbuvir-based regimens for hepatitis C treatment among people who inject drugs and those in opioid substitution therapy in canada. Hepatology 2017, 66 (Supplement 1):591A.

609. Jenkins RA: Getting to Zero: We Can't Do It Without Addressing Substance Use. AIDS Educ Prev 2018, 30(3):225-231.

610. Johnson RL, Botwinick G, Sell RL, Martinez J, Siciliano C, Friedman LB, Dodds S, Shaw K, Walker LE, Sotheran JL et al: The utilization of treatment and case management services by HIV-infected youth. J Adolesc Health 2003, 33(2 Suppl):31-38.

611. Jork K: [Tobacco smoking cessation. Methods and aids]. Med Monatsschr Pharm 1996, 19(6):158-163.

612. Juday TR, Wu A, Celentano DD, Frick KD, Wang MC, Vlahov D: The role of medicaid HMO enrollment in the longitudinal utilization of medical care services in a cohort of injecting drug users in Baltimore, Maryland. Substance Abuse 2003, 24(1):27-41.

613. Kaan IA, Jones T, McCaughan GW: Have we significantly underestimated the capacity in the Australian health system to treat chronic hepatitis C infection in an interferon-free era? Internal Medicine Journal 2017, 47(3):269-274.

614. Kachko AV, Ershov AE, Gavrilova IV, Shustov AV, Kochneva GV, Sivolobova GF, Grazhdantseva AA, Bukin VN, Komissarova MA, Netesov SV: The occurrence rate of HGV/GBV-C RNA and risk factors in patients of narcological dispensary in Novosibirsk. [Russian]. Zhurnal mikrobiologii, epidemiologii, i immunobiologii 2005(2):25-30.

615. Kachko AV, Ershov AE, Gavrilova IV, Shustov AV, Kochneva GV, Sivolobova GF, Grazhdantseva AA, Bukin VN, Komissarova MA, Netesov SV: [The occurrence rate of HGV/GBV-C RNA and risk factors

in patients of narcological dispensary in Novosibirsk]. Zh Mikrobiol Epidemiol Immunobiol 2005(2):25-30.

616. Kalichman SC: The HIV/AIDS treatment adherence, health outcomes and cost study: Conceptual foundations and overview. AIDS Care - Psychological and Socio-Medical Aspects of AIDS/HIV 2004, 16(SUPPL. 1):S6-S21.

617. Kalichman SC: Co-occurrence of treatment nonadherence and continued HIV transmission risk behaviors: implications for positive prevention interventions. Psychosomatic medicine 2008, 70(5):593-597.

618. Kalichman SC, Kelly JA, Johnson JR, Bulto M: Factors associated with risk for HIV infection among chronic mentally ill adults. American Journal of Psychiatry 1994, 151(2):221-227.

619. Kamarulzaman A, Altice FL: Challenges in managing HIV in people who use drugs. Current opinion in infectious diseases 2015, 28(1):10-16.

620. Kamarulzaman A, Altice FL: Challenges in managing HIV in people who use drugs. Current opinion in infectious diseases 2015, 28(1):10-16.

621. Kang D, Tao X, Liao M, Li J, Zhang N, Zhu X, Sun X, Lin B, Su S, Hao L et al: An integrated individual, community, and structural intervention to reduce HIV/STI risks among female sex workers in China. BMC Public Health 2013, 13:717.

622. Kaptan F, Ormen B, Turker N, El S, Ural S, Vardar I, Coskun NA, Er H, Unal Z: Retrospective evaluation of 128 cases infected with human immunodeficiency virus. Turkiye Klinikleri Journal of Medical Sciences 2011, 31(3):525-533.

623. Karumbi J, Garner P: Directly observed therapy for treating tuberculosis. Cochrane Database of Systematic Reviews 2015, 2015 (5) (no pagination)(CD003343).

624. Katz MH, Cunningham WE, Mor V, Andersen RM, Kellogg T, Zierler S, Crystal SC, Stein MD, Cylar K, Bozzette SA et al: Prevalence and predictors of unmet need for supportive services among HIV-infected persons: impact of case management. Medical care 2000, 38(1):58-69.

625. Keats J, Micallef M, Grebely J, Hazelwood S, Everingham H, Shrestha N, Jones T, Bath N, Treloar C, Dore GJ et al: Assessment and delivery of treatment for hepatitis C virus infection in an opioid substitution treatment clinic with integrated peer-based support in Newcastle, Australia. The International journal on drug policy 2015, 26(10):999-1006.

626. Keats J, Micallef M, Grebely J, Hazelwood S, Everingham H, Shrestha N, Jones T, Bath N, Treloar C, Dore GJ et al: Assessment and delivery of treatment for hepatitis C virus infection in an opioid substitution treatment clinic with integrated peer-based support in Newcastle, Australia. International Journal of Drug Policy 2015, 26(10):999-1006.

627. Keats J, Micallef M, Grebely J, Hazelwood S, Everingham H, Shrestha N, Jones T, Bath N, Treloar C, Dore GJ et al: Assessment and delivery of treatment for hepatitis C virus infection in an opioid substitution treatment clinic with integrated peer-based support in Newcastle, Australia. International Journal of Drug Policy 2015, 26(10):999-1006.

628. Keene JM, Stimson GV: Professional ideologies and the development of syringe exchange: Wales as a case study. Medical anthropology 1997, 18(1):85-105.

629. Keith Branham D, Borders TF, Stewart KE, Curran GM, Booth BM: Acceptability of HIV Testing Sites Among Rural and Urban African Americans Who Use Cocaine. Aids Behav 2017, 21(2):576-586.

630. Kelen GD, Fleetwood D: HIV infection and intravenous drug users. Implications for emergency services. Emerg Med Clin North Am 1990, 8(3):653-664.

631. Kelly JA, Kalichman SC: Increased attention to human sexuality can improve HIV-AIDS prevention efforts: key research issues and directions. J Consult Clin Psychol 1995, 63(6):907-918.

632. Kelly JA, Murphy DA, Bahr GR, Brasfield TL, Davis DR, Hauth AC, Morgan MG, Stevenson LY, Eilers MK: AIDS/HIV risk behavior among the chronic mentally ill. American Journal of Psychiatry 1992, 149(7):886-889.

633. Kelly JF, Stout RL, Magill M, Tonigan JS, Pagano ME: Spirituality in recovery: a lagged mediational analysis of alcoholics anonymous' principal theoretical mechanism of behavior change. Alcohol Clin Exp Res 2011, 35(3):454-463.

634. Kelly JJ, Chu SY, Buehler JW: AIDS deaths shift from hospital to home. American Journal of Public Health 1993, 83(10):1433-1437.

635. Kendall CE, Boucher LM, Mark AE, Martin A, Marshall Z, Boyd R, Oickle P, Diliso N, Pineau D, Renaud B et al: A cohort study examining emergency department visits and hospital admissions among people who use drugs in Ottawa, Canada. Harm Reduction Journal 2017, 14 (1) (no pagination)(16).

636. Kendall CE, Boucher LM, Mark AE, Martin A, Marshall Z, Boyd R, Oickle P, Diliso N, Pineau D, Renaud B et al: A cohort study examining emergency department visits and hospital admissions among people who use drugs in Ottawa, Canada.[Erratum appears in Harm Reduct J. 2017 Jul 3;14 (1):42; PMID: 28673291]. Harm Reduction Journal 2017, 14(1):16.

637. Kendall EA, Theron D, Franke MF, van Helden P, Victor TC, Murray MB, Warren RM, Jacobson KR: Alcohol, hospital discharge, and socioeconomic risk factors for default from multidrug resistant tuberculosis treatment in rural South Africa: a retrospective cohort study. PLoS ONE [Electronic Resource] 2013, 8(12):e83480.

638. Kern AM, Akerman SC, Nordstrom BR: Opiate dependence in schizophrenia: Case presentation and literature review. Journal of Dual Diagnosis 2014, 10(1):52-57.

639. Kerouac JD: The regulation of home diagnostic tests for genetic disorders: Can the FDA deny a premarket application on the basis of the device's social impacts? Journal of Biolaw and Business 2002, 5(1):34-43.

640. Keshavjee S, Gelmanova IY, Pasechnikov AD, Mishustin SP, Andreev YG, Yedilbayev A, Furin JJ, Mukherjee JS, Rich ML, Nardell EA et al: Treating multidrug-resistant tuberculosis in Tomsk, Russia: Developing programs that address the linkage between poverty and disease. Reducing the Impact of Poverty on Health and Human Development: Scientific Approaches 2008, Annals of the New York Academy of Sciences. 1136:1-11.

641. Khalsa J, Vocci F, Altice F, Fiellin D, Miller V: Buprenorphine and HIV primary care: New opportunities for integrated treatment. Clinical Infectious Diseases 2006, 43(SUPPL. 4):S169-S170.

642. Khalsa JH, Elkashef A: Interventions for HIV and hepatitis C virus infections in recreational drug users. Clin Infect Dis 2010, 50(11):1505-1511.

643. Khalsa JH, Elkashef A: Interventions for HIV and hepatitis C virus infections in recreational drug users. Clin Infect Dis 2010, 50(11):1505-1511.

644. Khatana GH, Masoodi M, Amin M: Factors affecting applicability of "home-based interventional model" for active case finding among household contacts of index cases of pulmonary tuberculosis in Kashmir. Indian Journal of Tuberculosis 2017, 64(3):189-195.

645. Kielly J, Kelly DV, Asghari S, Burt K, Biggin J: Patient satisfaction with chronic HIV care provided through an innovative pharmacist/nurse-managed clinic and a multidisciplinary clinic. Canadian Pharmacists Journal 2017, 150(6):397-406.

646. Kikumbih SN, Isingo R, Boerma JT: Consequences of adult HIV infection for outpatient morbidity and treatment costs: a prospective study in a factory clinic in Tanzania. Health Policy Plan 1997, 12(3):234-239.

647. Kilbourne AM, Greenwald DE, Hermann RC, Charns MP, McCarthy JF, Yano EM: Financial incentives and accountability for integrated medical care in department of veterans affairs mental health programs. Psychiatric Services 2010, 61(1):38-44.

648. Kim HF, Fisch MJ: Antidepressant use in ambulatory cancer patients. Current Oncology Reports 2006, 8(4):275-281.

649. Kim JH, Psevdos G, Suh J, Sharp VL: Co-infection of hepatitis B and hepatitis C virus in human immunodeficiency virus-infected patients in New York City, United States. World Journal of Gastroenterology 2008, 14(43):6689-6693.

650. Kim JH, Psevdos Jr G, Suh J, Sharp VL: Co-infection of hepatitis B and hepatitis C virus in human immunodeficiency virus-infected patients in New York City, United States. World Journal of Gastroenterology 2008, 14(43):6689-6693.

651. Kim SY, Solomon DH, Liu J, Chang G, Daniel GW, Schneeweiss S: Accuracy of identifying outpatient neutropenia diagnoses in claims data. Pharmacoepidemiology and Drug Safety 2010, 19:S74-S75.

652. Kim TW, Kertesz SG, Horton NJ, Tibbetts N, Samet JH: Episodic homelessness and health care utilization in a prospective cohort of HIV-infected persons with alcohol problems. BMC Health Services Research 2006, 6 (no pagination)(19).

653. Kinahan JC, Surah S, Keating S, Bergin C, Mulcahy F, Lyons F, Keenan E: Effect of integrating HIV and addiction care for non-engaging HIV-infected opiate-dependent patients. Irish Journal of Medical Science 2016, 185(3):623-628.

654. King LJ, Key MD, Towe S, Meade CS: Relationship of alcohol and cocaine on mood episodes among HIV-infected adults with bipolar disorder and major depressive disorder. Drug and Alcohol Dependence 2015, 146:e158-e159.

655. King VL, Jr., Brooner RK, Bigelow GE, Schmidt CW, Felch LJ, Gazaway PM: Condom use rates for specific sexual behaviors among opioid abusers entering treatment. Drug Alcohol Depend 1994, 35(3):231-238.

656. Kinsman H, Korthuis T, Saha S, Sharp V, Cohn J, Moore R, Beach MC: "We're not going there": An analysis of patient-provider communication about narcotic analgesia for chronic non-malignant pain. Journal of General Internal Medicine 2011, 26:S185-S186.

657. Kipp AM, Rebeiro PF, Shepherd BE, Brinkley-Rubinstein L, Turner M, Bebawy S, Sterling TR, Hulgan T: Daily Marijuana Use is Associated with Missed Clinic Appointments Among HIV-Infected Persons Engaged in HIV Care. AIDS and behavior 2017, 21(7):1996-2004.

658. Kipp W, Tindyebwa D, Karamagi E, Rubaale T: How much should we expect? Family caregiving of AIDS patients in rural Uganda. Journal of Transcultural Nursing 2007, 18(4):358-365.

659. Kissinger P, Clark R, Rice J, Kutzen H, Morse A, Brandon W: Evaluation of a program to remove barriers to public health care for women with HIV infection. South Med J 1995, 88(11):1121-1125.

660. Klinkenberg WD, Caslyn RJ, Morse GA, Yonker RD, McCudden S, Ketema F, Constantine NT: Prevalence of human immunodeficiency virus, hepatitis B, and hepatitis C among homeless persons with co-occurring severe mental illness and substance use disorders. Comprehensive Psychiatry 2003, 44(4):293-302.

661. Klinkenberg WD, Sacks S: Mental disorders and drug abuse in persons living with HIV/AIDS. AIDS Care - Psychological and Socio-Medical Aspects of AIDS/HIV 2004, 16(SUPPL. 1):S22-S42.

662. Klinkenberg WD, Sacks S, Hiv/Aids Treatment Adherence HO, Cost Study G: Mental disorders and drug abuse in persons living with HIV/AIDS. AIDS Care 2004, 16 Suppl 1:S22-42.

663. Knobel H, Escobar I, Polo R, Ortega L, Martin-Conde MT, Casado JL, Codina C, Fernandez J, Galindo MJ, Ibarra O et al: [Recommendations from GESIDA/SEFH/PNS to improve adherence to antiviral treatment (2004)]. Enfermedades Infecciosas y Microbiologia Clinica 2005, 23(4):221-231.

664. Knott A, Dieperink E, Willenbring ML, Heit S, Durfee JM, Wingert M, Johnson JR, Thuras P, Ho SB: Integrated psychiatric/medical care in a chronic hepatitis C clinic: Affect on antiviral treatment evaluation and outcomes. American Journal of Gastroenterology 2006, 101(10):2254-2262.

665. Knott A, Dieperink E, Willenbring ML, Heit S, Durfee JM, Wingert M, Johnson JR, Thuras P, Ho SB: Integrated psychiatric/medical care in a chronic hepatitis C clinic: effect on antiviral treatment evaluation and outcomes. American Journal of Gastroenterology 2006, 101(10):2254-2262.

666. Knowlton AR: Informal HIV caregiving in a vulnerable population: toward a network resource framework. Soc Sci Med 2003, 56(6):1307-1320.

667. Knowlton AR, Hoover DR, Chung SE, Celentano DD, Vlahov D, Latkin CA: Access to medical care and service utilization among injection drug users with HIV/AIDS. Drug and Alcohol Dependence 2001, 64(1):55-62.

668. Kobayashi JS, Standridge WL: An integrated program for comprehensive HIV care. New directions for mental health services 2000(87):111-118.

669. Komiti A, Judd F, Grech P, Mijch A, Hoy J, Williams B, Street A, Lloyd JH: Depression in people living with HIV/AIDS attending primary care and outpatient clinics. Aust N Z J Psychiatry 2003, 37(1):70-77.

670. Korthuis PT, McCarty D, Weimer M, Bougatsos C, Blazina I, Zakher B, Grusing S, Devine B, Chou R: Primary care-based models for the treatment of opioid use disorder: A scoping review. Annals of Internal Medicine 2017, 166(4):268-278.

671. Korthuis PT, Saha S, Chander G, McCarty D, Moore RD, Cohn JA, Sharp VL, Beach MC: Substance use and the quality of patient-provider communication in HIV clinics. AIDS and behavior 2011, 15(4):832-841.

672. Krach P, DeVaney S, DeTurk C, Zink MH: Functional status of the oldest-old in a home setting. Journal of advanced nursing 1996, 24(3):456-464.

673. Krakauer EL, Ngoc NT, Green K, Van Kham L, Khue LN: Vietnam: integrating palliative care into HIV/AIDS and cancer care. J Pain Symptom Manage 2007, 33(5):578-583.

674. Kresina TF, Bruce RD, Cargill VA, Cheever LW: Integrating care for hepatitis C virus (HCV) and primary care for HIV for injection drug users coinfected with HIV and HCV. Clinical Infectious Diseases 2005, 41(1 SUPPL.):S83-S88.

675. Kresina TF, Bruce RD, Cargill VA, Cheever LW: Integrating care for hepatitis C virus (HCV) and primary care for HIV for injection drug users coinfected with HIV and HCV. Clin Infect Dis 2005, 41 Suppl 1:S83-88.

676. Kresina TF, Bruce RD, Cargill VA, Cheever LW: Integrating care for hepatitis C virus (HCV) and primary care for HIV for injection drug users coinfected with HIV and HCV. Clin Infect Dis 2005, 41 Suppl 1:S83-88.

677. Kresina TF, Bruce RD, Lubran R, Clark HW: Integration of viral hepatitis services into opioid treatment programs. Journal of Opioid Management 2008, 4(6):369-381.

678. Kresina TF, Eldred L, Bruce RD, Francis H: Integration of pharmacotherapy for opioid addiction into HIV primary care for HIV/hepatitis C virus-co-infected patients. Aids 2005, 19 Suppl 3:S221-226.

679. Kresina TF, Eldred L, Bruce RD, Francis H: Integration of pharmacotherapy for opioid addiction into HIV primary care for HIV/hepatitis C virus-co-infected patients. Aids 2005, 19 Suppl 3:S221-226.

680. Kresina TF, Eldred L, Bruce RD, Francis H: Integration of pharmacotherapy for opioid addiction into HIV primary care for HIV/hepatitis C virus-co-infected patients. Aids 2005, 19 Suppl 3:S221-226.

681. Kresina TF, Khalsa J, Cesari H, Francis H: Hepatitis C virus infection and substance abuse: medical management and developing models of integrated care--an introduction. Clinical infectious diseases : an official publication of the Infectious Diseases Society of America 2005, 40 Suppl 5:S259-262.

682. Kresina TF, Khalsa J, Cesari H, Francis H: Hepatitis C virus infection and substance abuse: Medical management and developing models of integrated care - An introduction. Clinical Infectious Diseases 2005, 40(SUPPL. 5):S259-S262.

683. Kresina TF, Lubran R: Improving public health through access to and utilization of medication assisted treatment. International Journal of Environmental Research and Public Health 2011, 8(10):4102-4117.

684. Kresina TF, Seeff LB, Francis H: Hepatitis C infection and injection drug use: The role of hepatologists in evolving treatment efforts. Hepatology 2004, 40(3):516-519.

685. Kresina TF, Sylvestre D, Seeff L, Litwin AH, Hoffman K, Lubran R, Clark HW: Hepatitis infection in the treatment of opioid dependence and abuse. Substance Abuse 2008, 1:15-61.

686. Kristensen O, Sundoy A, Skeie K, Vederhus JK, Oye I, Opsal A, Rysstad O, Gallefoss F: [Short-term treatment of Hepatitis C in heroin-dependent patients]. Tidsskr Nor Laegeforen 2009, 129(16):1639-1642.

687. Kritz S: Implementation of an electronic information system to enhance practice at an opioid treatment program. American Journal on Addictions 2011, 20 (4):383.

688. Kritz S, Brown LS, Chu M, Zavala R: Implementation of an electronic information system to enhance practice at an opioid treatment program. American Journal on Addictions 2010, 19 (4):370-371.

689. Krook A, Kastrup A, Lidman K, Mann C, Stymne A: Somatic care wanted by HIV-infected intravenous drug abusers: the patients' opinions and experiences. AIDS Care 1995, 7(3):375-379.

690. Krupitsky EM, Zvartau EE, Lioznov DA, Tsoy MV, Egorova VY, Belyaeva TV, Antonova TV, Brazhenko NA, Zagdyn ZM, Verbitskaya EV et al: Co-morbidity of infectious and addictive diseases in St. Petersburg and the Leningrad Region, Russia. Eur Addict Res 2006, 12(1):12-19.

691. Kudryashova Hernandez L: One-stop shop service delivery model: Integrating prevention interventions with HIV Care/Treatment services in a community-based medical home setting. Sexually Transmitted Infections Conference: STI and AIDS World Congress 2013, 89(no pagination).

692. Kuehn BM: Integrated care needed for patients with HIV, drug abuse, and mental illness. JAMA - Journal of the American Medical Association 2008, 300(5):494-495.

693. Kupprat SA, Dayton A, Guschlbauer A, Halkitis PN: Case manager-reported utilization of support group, substance use and mental health services among HIV-positive women in New York City. AIDS Care - Psychological and Socio-Medical Aspects of AIDS/HIV 2009, 21(7):874-880.

694. Kutzen HS: Integration of palliative care into primary care for human immunodeficiency virus-infected patients. Am J Med Sci 2004, 328(1):37-47.

695. Kyaw NTT, Kumar AMV, Oo MM, Oo HN, Kyaw KWY, Thiha S, Aung TK, Win T, Mon YY, Harries AD: Long-term outcomes of second-line antiretroviral treatment in an adult and adolescent cohort in Myanmar. Glob Health Action 2017, 10(1):1290916.

696. Labbe AK, Wilner JG, Kosiba JD, Gonzalez A, Smits JA, Zvolensky MJ, Norton PJ, O'Cleirigh C: Demonstration of an Integrated Treatment for Smoking Cessation and Anxiety Symptoms in People With HIV: A Clinical Case Study. Cognitive and Behavioral Practice 2017, 24(2):200-214.

697. Lafitte C, Bonnal F, Daulouede JP, Tignol J: [Effect of treatment with methadone on utilization of medical care by addicts seropositive for the AIDS virus. Results of a descriptive study, Bayonne, 1996]. Encephale 1998, 24(3):205-214.

698. Lagisetty PA, Bohnert A, Maust D, Heisler M: Physical health comorbidity associated with primary care visits for substance use disorders. Journal of General Internal Medicine 2016, 1):S338-S339.

699. Lahaie C: [Towards an integrated network for substance abusers with HIV/AIDS]. Infirm Que 2002, 9(6):27-31, 37-28.

700. Laine C, Hauck WW, Gourevitch MN, Rothman J, Cohen A, Turner BJ: Regular outpatient medical and drug abuse care and subsequent hospitalization of persons who use illicit drugs. Jama 2001, 285(18):2355-2362.

701. Laine C, Hauck WW, Turner BJ: Outpatient patterns of care and longitudinal intensity of antiretroviral therapy for HIV-infected drug users. Medical care 2002, 40(10):976-995.

702. Laine C, Lin YT, Hauck WW, Turner BJ: Availability of medical care services in drug treatment clinics associated with lower repeated emergency department use. Medical Care 2005, 43(10):985-995.

703. Laine C, Newschaffer C, Zhang D, Rothman J, Hauck WW, Turner BJ: Models of care in New York State Medicaid substance abuse clinics. Range of services and linkages to medical care. Journal of Substance Abuse 2000, 12(3):271-285.

704. Laine C, Zhang D, Hauck WW, Turner BJ: HIV-1 RNA viral load monitoring in HIV-infected drug users on antiretroviral therapy: relationship with outpatient care patterns. J Acquir Immune Defic Syndr 2002, 29(3):270-274.

705. Lambdin BH, Kral AH, Comfort M, Lopez AM, Lorvick J: Associations of criminal justice and substance use treatment involvement with HIV/HCV testing and the HIV treatment cascade among people who use drugs in Oakland, California. Addiction science & clinical practice 2017, 12(1):13.

706. Lambdin BH, Lorvick J, Mbwambo JK, Rwegasha J, Hassan S, Lum P, Kral AH: Prevalence and predictors of HCV among a cohort of opioid treatment patients in Dar es Salaam, Tanzania. International Journal of Drug Policy 2017, 45:64-69.

707. Lambers FA, Stolte IG, van den Berg CH, Coutinho RA, Prins M: Harm reduction intensity-Its role in HAART adherence amongst drug users in Amsterdam. International Journal of Drug Policy 2011, 22(3):210-218.

708. Lambers FAE, Stolte IG, van den Berg CHSB, Coutinho RA, Prins M: Harm reduction intensity-Its role in HAART adherence amongst drug users in Amsterdam. International Journal of Drug Policy 2011, 22(3):210-218.

709. Lambert A, Kirk G, Astemborski J, Wise RA, Drummond MB: Mhealth monitoring informs respiratory symptom assessments among HIV-infected individuals with COPD. American Journal of Respiratory and Critical Care Medicine Conference: American Thoracic Society International Conference, ATS 2017, 195(no pagination).

710. Lambert J, Cullen W, Oprea C, Story A, Sanchez JM, Surey J: Hepcare Europe: HepCheck; reaching vulnerable populations. Journal of Hepatology 2018, 68 (Supplement 1):S153.

711. Lambert JS, Murphy C, O'Carroll A, Farrell J, Patel A, Avramovic G, Cullen W, Ni Cheallaigh C: The Dublin hepcheck study: Community based testing of HCV by point of care oraquick HCV saliva test in homeless populations. Journal of Hepatology 2016, 1):S726.

712. Lambert JS, Murphy C, Patel A, Crowley D, Stewart S, Farrell J, Cullen W, McKenna-Barry M: Opportunistic fibroscan testing in a dublin general practice (GP) managing opiate substitution therapy: The hepcare study. Journal of Hepatology 2016, 1):S725-S726.

713. Lang JP, Bonnewitz ML, Kusterer M, Lalanne-Tongio L: Alcohol consumption in patients with psychiatric disorders: Assessment and treatment. [French]. Encephale 2014, 40(4):301-307.

714. Lang JP, Bonnewitz ML, Kusterer M, Lalanne-Tongio L: [Alcohol consumption in patients with psychiatric disorders: assessment and treatment]. Encephale 2014, 40(4):301-307.

715. Lang JP, Michel L, Melin P, Schoeffler M, Gauchet A, Rousseaux C, Cartier V, Henry C: Management of psychiatric disorders and addictive behaviors in patients with viral hepatitis C in France. Gastroenterol Clin Biol 2009, 33(1 Pt 1):1-7.

716. Langendam MW, van Haastrecht HJ, van Brussel GH, Reurs H, van den Hoek AA, Coutinho RA, van Ameijden EJ: Differentiation in the Amsterdam methadone dispensing circuit: determinants of methadone dosage and site of methadone prescription. Addiction 1998, 93(1):61-72.

717. Laraque F, Ford M, Desai P, Bresnahan MP, Litwin AH, Schwartz JM, Shukla SJ, Weiss JJ, Wyatt B, Perumalswami PV: Comprehensive care coordination reduces disparities in access to hepatitis C care and treatment in highly complex patients. Hepatology 2016, 64 (1 Supplement 1):95A.

718. Lardelli P, de la Fuente L, Alonso JM, Lopez R, Bravo MJ, Delgado-Rodriguez M: Geographical variations in the prevalence of HIV infection among drug users receiving ambulatory treatment in Spain. International Journal of Epidemiology 1993, 22(2):306-314.

719. Larios SE, Masson CL, Shopshire MS, Hettema J, Jordan AE, McKnight C, Young C, Khalili M, Seewald RM, Min A et al: Education and counseling in the methadone treatment setting improves knowledge of viral hepatitis. Journal of Substance Abuse Treatment 2014, 46(4):528-531.

720. Larson AM, Curtis JR: Integrating palliative care for liver transplant candidates: "Too well for transplant, too sick for life". Journal of the American Medical Association 2006, 295(18):2168-2176.

721. Larue F, Fontaine A, Colleau SM: Underestimation and undertreatment of pain in HIV disease: multicentre study. Bmj 1997, 314(7073):23-28.

722. Latypov A, Otiashvili D, Zule W: Drug scene, drug use and drug-related health consequences and responses in Kulob and Khorog, Tajikistan. International Journal of Drug Policy 2014, 25(6):1204-1214.

723. Lawoyin D, McCoy-Collins R, Obayomi T, Reid E, Sam F, Bonnick A, Jones R, Lawoyin J: Validity of self completed health questionnaire among oral surgery patients in capitol dental. African Journal Biomedical Research 2008, 11(3):275-279.

724. Lazarus JV, Sperle I, Maticic M, Wiessing L: A systematic review of Hepatitis C virus treatment uptake among people who inject drugs in the European Region. BMC Infectious Diseases 2014, 14 (no pagination)(S16).

725. Le GM, Nguyen H, Bart G, Rieckmann TR, Tran H, Darfler K, Rawson R: Assist in HIV/AIDS service settings: Comparison of self-screening and peer-support screening method. Drug and Alcohol Dependence 2015, 156:e123.

726. Lea T, Kolstee J, Lambert S, Ness R, Hannan S, Holt M: Methamphetamine treatment outcomes among gay men attending a LGBTI-specific treatment service in Sydney, Australia. PLoS ONE [Electronic Resource] 2017, 12(2):e0172560.

727. Leclerc S, Brunschwig O, Berki-Benhaddad Z, Soyris D, Grataud C, Breton G, Leport C, Vilde JL: [Antiretroviral therapy for HIV-infected patients with schizophrenia. Coordinated multidisciplinary management (7 cases)]. Presse Med 2005, 34(6):431-437.

728. Leff JA, Hernandez D, Teixeira PA, Castellon PC, Feaster DJ, Rodriguez AE, Santana-Bagur JL, De Leon SM, Vidot JV, Metsch LR et al: The structural and health policy environment for delivering integrated HIV and substance use disorder treatments in Puerto Rico. BMC health services research 2017, 17(1):232.

729. Legoupil C, Peltier A, Henry Kagan V, Segouin C, Alberti C, de Masse L, Shelly M, Krastinova E: Out-of-Hospital screening for HIV, HBV, HCV and Syphilis in a vulnerable population, a public health challenge. AIDS Care 2017, 29(6):686-688.

730. Leonhardt KK, Gentile F, Gilbert BP, Aiken M: A cluster of tuberculosis among crack house contacts in San Mateo County, California. American Journal of Public Health 1994, 84(11):1834-1836.

731. Lepretre A, Ba I, Lacombe K, Maynart M, Toufik A, Ndiaye O, Kane CT, Gozlan J, Tine J, Ndoye I et al: Prevalence and behavioural risks for HIV and HCV infections in a population of drug users of Dakar, Senegal: The ANRS 12243 UDSEN study. Journal of the International AIDS Society 2015, 18 (1) (no pagination)(19888).

732. Lesko CR, Cole SR, Hall HI, Mugavero MJ: Effect of ART on mortality generalized to newly HIV-diagnosed persons in the USA. Topics in Antiviral Medicine 2015, 23:247.

733. Levin JM, Huckins E: From diagnosis to cure: An integrated health system baby boomer HCV screening and therapy management program. Hepatology 2017, 66 (Supplement 1):821A.

734. Levy MH, Lerwitworapong J: Issues facing TB control (3.1) - Tuberculosis in prisons. Scottish Medical Journal 2000, 45(5 SUPPL.):30-32.

735. Lewis D, Bellis M: General practice or drug clinic for methadone maintenance? A controlled comparison of treatment outcomes. International Journal of Drug Policy 2001, 12(1):81-89.

736. Li X, He G, Wang H, Williams AB: Consequences of drug abuse and HIV/AIDS in China: Recommendations for integrated care of HIV-infected drug users. AIDS Patient Care and STDs 2009, 23(10):877-884.

737. Liang TS, Erbelding E, Jacob CA, Wicker H, Christmyer C, Brunson S, Richardson D, Ellen JM: Rapid HIV testing of clients of a mobile STD/HIV clinic. AIDS Patient Care STDS 2005, 19(4):253-257.

738. Liebman J, Pat Lamberti M, Altice F: Effectiveness of a mobile medical van in providing screening services for STDs and HIV. Public Health Nursing 2002, 19(5):345-353.

739. Liebschutz JM, Geier JL, Horton NJ, Chuang CH, Samet JH: Physical and sexual violence and health care utilization in HIV-infected persons with alcohol problems. AIDS Care - Psychological and Socio-Medical Aspects of AIDS/HIV 2005, 17(5):566-578.

740. Lima M, Costa JA, Figueiredo Wdos S, Schraiber LB: [Invisibility of drug use and support for professionals of the AIDS services]. Rev Saude Publica 2007, 41 Suppl 2:6-13.

741. Linas BP, Hu H, Barter DM, Horberg M: Hepatitis C screening trends in a large integrated health system. American Journal of Medicine 2014, 127(5):398-405.

742. Lipsitz MC, Segura ER, Castro JL, Smith E, Medrano C, Clark JL, Lake JE, Cabello R: Bringing testing to the people - benefits of mobile unit HIV/syphilis testing in Lima, Peru, 2007-2009. Int J STD AIDS 2014, 25(5):325-331.

743. Liss G, Stine J, Ezmerli N, Lewis JH: Safety and efficacy of same day endoscopy (SDE) combined with percutaneous liver biopsy (PLB). Gastroenterology 2010, 1):S328.

744. Litwin AH: Strategies to enhance HCV assessment and adherence to therapy among people who use drugs. Suchtmedizin in Forschung und Praxis 2013, 15 (4):220.

745. Litwin AH, Soloway I, Gourevitch MN: Integrating services for injection drug users infected with hepatitis C virus with methadone maintenance treatment: Challenges and opportunities. Clinical Infectious Diseases 2005, 40(SUPPL. 5):S339-S345.

746. Lohmann KM, Talbot J: A case of questionable medical capacity and immutable individual autonomy. Journal of General Internal Medicine 2015, 30:S332.

747. Loinaz Segurola C, JL LIN, Burgos Rde L, Martin Rios D, Ochando Cerdan F, Alonso Lopez S, Martel Villagran J, Gutierrez Garcia ML, Fernandez Cebrian JM, Fernandez Rodriguez C: Multidisciplinary Approach in Hepatocellular Carcinoma in a Level II Hospital: The First Decade of Hospital Universitario Fundacion Alcorcon. Hepatogastroenterology 2015, 62(140):971-977.

748. Lombard F, Proescholdbell RJ, Cooper K, Musselwhite L, Quinlivan EB: Adaptations across clinical sites of an integrated treatment model for persons with HIV and substance abuse. AIDS Patient Care and STDs 2009, 23(8):631-638.

749. Longshore D, Hsieh S: Drug abuse treatment and risky sex: evidence for a cumulative treatment effect? Am J Drug Alcohol Abuse 1998, 24(3):439-451.

750. Loo VS, Diaz T, Gadelha AMJ, Pereira Campos D, Pilloto JH, Starling Brandao Jr P, Grinsztejin B, Veloso Dos Santos VG: Managing HIV-infected patients on antiretroviral therapy in Rio de Janeiro, Brazil: Do providers follow national guidelines? AIDS Care - Psychological and Socio-Medical Aspects of AIDS/HIV 2004, 16(7):834-840.

751. Lopez R, Bravo MJ, Barrio G, Lardelli P, Bueno A, de la Fuente L: HIV-transmission knowledge in drug users from outpatient facilities in Spain. A national survey. Drug Alcohol Depend 1994, 34(3):181-190.

752. Lucas GM: Substance abuse, adherence with antiretroviral therapy, and clinical outcomes among HIV-infected individuals. Life Sciences 2011, 88(21-22):948-952.

753. Lucas GM, Weidle PJ, Hader S, Moore RD: Directly administered antiretroviral therapy in an urban methadone maintenance clinic: a nonrandomized comparative study. Clin Infect Dis 2004, 38 Suppl 5:S409-413.

754. Lucas GM, Weidle PJ, Hader S, Moore RD: Directly administered antiretroviral therapy in an urban methadone maintenance clinic: a nonrandomized comparative study. Clin Infect Dis 2004, 38 Suppl 5:S409-413.

755. Lunsky Y, Durbin A, Brown HK, Bansal S, Heifetz M, Antoniou T: Health profiles and associated service use among adults with HIV and intellectual and developmental disabilities. Aids 2017, 31(5):697-705.

756. Lyketsos CG, Fishman M, Hutton H, Cox T, Hobbs S, Spoler C, Hunt W, Driscoll J, Treisman G: The effectiveness of psychiatric treatment for HIV-infected patients. Psychosomatics 1997, 38(5):423-432.

757. Lynch FL, McCarty D, Mertens J, Perrin NA, Green CA, Parthasarathy S, Dickerson JF, Anderson BM, Pating D: Costs of care for persons with opioid dependence in commercial integrated health systems. Addict Sci Clin Pract 2014, 9:16.

758. Macalino GE, Hogan JW, Mitty JA, Bazerman LB, Delong AK, Loewenthal H, Caliendo AM, Flanigan TP: A randomized clinical trial of community-based directly observed therapy as an adherence intervention for HAART among substance users. Aids 2007, 21(11):1473-1477.

759. Macalino GE, Hogan JW, Mitty JA, Bazerman LB, Delong AK, Loewenthal H, Caliendo AM, Flanigan TP: A randomized clinical trial of community-based directly observed therapy as an adherence intervention for HAART among substance users. Aids 2007, 21(11):1473-1477.

760. MacHattie A, Payne HM, McDougall P, Baltzer Turje R: The role of the nurse practitioner in an integrated HIV/AIDS health care setting at the DR peter centre. Canadian Journal of Infectious Diseases and Medical Microbiology 2015, SB):116B.

761. Mackey TK, Strathdee SA: Responding to the public health consequences of the Ukraine crisis: An opportunity for global health diplomacy. Journal of the International AIDS Society 2015, 18 (1) (no pagination)(19410).

762. MacMaster S, Wodarski J: Initial results from an HIV/AIDS primary prevention program with Caribbean young adults in The United States virgin Islands. Journal of Sexual Medicine 2011, 3):242.

763. Maddocks I: Palliative Care Education in the Developing Countries. Journal of Pain and Palliative Care Pharmacotherapy 2003, 17(3-4):211-221.

764. Magnus M, Schmidt N, Kirkhart K, Schieffelin C, Fuchs N, Brown B, Kissinger PJ: Association between ancillary services and clinical and behavioral outcomes among HIV-infected women. AIDS Patient Care STDS 2001, 15(3):137-145.

765. Magura S, Lee SJ, Salsitz EA, Kolodny A, Whitley SD, Taubes T, Seewald R, Joseph H, Kayman DJ, Fong C et al: Outcomes of buprenorphine maintenance in office-based practice. Journal of Addictive Diseases 2007, 26(2):13-23.

766. Malotte CK, Hollingshead JR, Larro M: Incentives vs outreach workers for latent tuberculosis treatment in drug users. Am J Prev Med 2001, 20(2):103-107.

767. Malotte CK, Hollingshead JR, Larro M: Incentives vs outreach workers for latent tuberculosis treatment in drug users. Am J Prev Med 2001, 20(2):103-107.

768. Malow R, Devieux JG, Stein JA, Rosenberg R, Jean-Gilles M, Attonito J, Koenig SP, Raviola G, Severe P, Pape JW: Depression, substance abuse and other contextual predictors of adherence to antiretroviral therapy (ART) among Haitians. AIDS and behavior 2013, 17(4):1221-1230.

769. Malow RM, Devieux JG, Rosenberg R, Dyer JG, St Lawrence JS: Integrated HIV care: HIV risk outcomes of pregnant substance abusers. Substance Use & Misuse 2006, 41(13):1745-1767.

770. Malow RM, Devieux JG, Rosenberg R, Dyer JG, St. Lawrence JS: Integrated HIV care: HIV risk outcomes of pregnant substance abusers. Substance Use and Misuse 2006, 41(13):1745-1767.

771. Malow RM, Ireland SJ, Halpert ES, Szapocznik J, McMahon RC, Haber L: A description of the Maternal Addiction Program of the University of Miami/Jackson Memorial Medical Center. Journal of Substance Abuse Treatment 1994, 11(1):55-60.

772. Mangla N, Mamun R, Weisberg I: Hepatitis screening in transgender patients with gender identity hormonal therapy. Gastroenterology 2017, 152 (5 Supplement 1):S1193-S1194.

773. Manos MM, Shvachko V, Pat Pauly M, Arduino JM: Characteristics and clinical management of a large cohort of HepatitisC patients from a northern california integrated health plan. Hepatology 2012, 1):660A.

774. Mansfeld M, Skrahina A, Shepherd L, Schultze A, Miller R, Miro J, Post F, Lundgren J, Mocroft A, Efsen A et al: Major differences in organization and availability of health care and medicines for HIV/TB coinfected patients across Europe. HIV Medicine 2015, 16(9):544-552.

775. Mansfeld M, Skrahina A, Shepherd L, Schultze A, Panteleev AM, Miller RF, Miro JM, Zeltina I, Tetradov S, Furrer H et al: Major differences in organization and availability of health care and medicines for HIV/TB coinfected patients across Europe. HIV Med 2015, 16(9):544-552.

776. Mansfeld M, Skrahina A, Shepherd L, Schultze A, Panteleev AM, Miller RF, Miro JM, Zeltina I, Tetradov S, Furrer H et al: Major differences in organization and availability of health care and medicines for HIV/TB coinfected patients across Europe. HIV Medicine 2015, 16(9):544-552.

777. Mansoor E, Parikh KP, Kim CH, Glessing B, Chak A, Cooper GS: Risk factors for pancreatic cancer in new-onset diabetes mellitus: A population based study. Gastroenterology 2016, 1):S233.

778. Mansoor E, Saleh MA, Ho EY, Dave M: Epidemiology of histoplasmosis in crohn's disease and ulcerative colitis in the united states between 1999 and 2016: A national study. Gastroenterology 2017, 152 (5 Supplement 1):S977.

779. Mao Y, Wu Z, Poundstone K, Wang C, Qin Q, Ma Y, Ma W: Development of a unified web-based national HIV/AIDS information system in China. International Journal of Epidemiology 2010, 39(SUPPL. 2):ii79-ii89.

780. Marais BJ, Lonnroth K, Lawn SD, Migliori GB, Mwaba P, Glaziou P, Bates M, Colagiuri R, Zijenah L, Swaminathan S et al: Tuberculosis comorbidity with communicable and non-communicable diseases: Integrating health services and control efforts. The Lancet Infectious Diseases 2013, 13(5):436-448.

781. Marazzi MC, Palombi L, Mancinelli S, Liotta G, Pana A: Care requirements of people with ARC/AIDS in Rome: Non-hospital services. AIDS Care - Psychological and Socio-Medical Aspects of AIDS/HIV 1994, 6(1):95-104.

782. Marcus JL, Hurley LB, Hare CB, Nguyen DP, Phengrasamy T, Silverberg MJ, Stoltey JE, Volk JE: Preexposure prophylaxis for HIV prevention in a large integrated health care system: adherence, renal safety, and discontinuation. Journal of Acquired Immune Deficiency Syndromes 2016, 73(5):540-546.

783. Marcus JL, Leyden WA, Chao CR, Horberg MA, Klein DB, Quesenberry CP, Towner WJ, Silverberg MJ: Immunodeficiency, AIDS-related pneumonia, and risk of lung cancer among HIV-infected individuals. Aids 2017, 31(7):989-993.

784. Marcus P: Integrating behavioral health into a transplantation service-one institution's approach. Current Psychiatry Reviews 2017, 13(1):20-22.

785. Markson LE, Cosler LE, Turner BJ: Implications of generalists' slow adoption of zidovudine in clinical practice. Archives of Internal Medicine 1994, 154(13):1497-1504.

786. Marshall BD, Green TC, Yedinak JL, Hadland SE: Harm reduction for young people who use prescription opioids extra-medically: Obstacles and opportunities. International Journal of Drug Policy 2016, 31:25-31.

787. Martin EG, Wang KH: Integrating substance abuse treatment into HIV care: Missed opportunities in the AIDS drug assistance program. Journal of Acquired Immune Deficiency Syndromes 2012, 29.

788. Martinez AD, Dimova R, Marks KM, Beeder AB, Zeremski M, Kreek MJ, Talal AH: Integrated internist - Addiction medicine - Hepatology model for hepatitis C management for individuals on methadone maintenance. Journal of Viral Hepatitis 2012, 19(1):47-54.

789. Maru DS, Bruce RD, Walton M, Springer SA, Altice FL: Persistence of virological benefits following directly administered antiretroviral therapy among drug users: results from a randomized controlled trial. J Acquir Immune Defic Syndr 2009, 50(2):176-181.

790. Maru DS, Bruce RD, Walton M, Springer SA, Altice FL: Persistence of virological benefits following directly administered antiretroviral therapy among drug users: results from a randomized controlled trial. J Acquir Immune Defic Syndr 2009, 50(2):176-181.

791. Masip M, Tuneu L, Pages N, Torras X, Gallego A, Guardiola JM, Faus MJ, Mangues MA: Prevalence and detection of neuropsychiatric adverse effects during hepatitis C treatment. International Journal of Clinical Pharmacy 2015, 37(6):1143-1151.

792. Mason J, Preisinger J, Sperling R, Walther V, Berrier J, Evans V: Incorporating HIV education and counseling into routine prenatal care: a program model. AIDS Educ Prev 1991, 3(2):118-123.

793. Mason K, Dodd Z, Sockalingam S, Altenberg J, Meaney C, Millson P, Powis J: Beyond viral response: A prospective evaluation of a community-based, multi-disciplinary, peer-driven model of HCV treatment and support. International Journal of Drug Policy 2015, 26(10):1007-1013.

794. Masson CL, Sorensen JL, Perlman DC, Shopshire MS, Delucchi KL, Chen T, Sporer K, Des Jarlais D, Hall SM: Hospital- versus community-based syringe exchange: A randomized controlled trial. AIDS Education and Prevention 2007, 19(2):97-110.

795. Masson CL, Sorensen JL, Phibbs CS, Okin RL: Predictors of medical service utilization among individuals with co-occurring HIV infection and substance abuse disorders. AIDS Care - Psychological and Socio-Medical Aspects of AIDS/HIV 2004, 16(6):744-755.

796. Masson S, Hewett M, Jones D, Docherty B, Saksena S: The "One-Stop" Clinic for prisoners with hepatitis C: An innovative approach. Gut 2009, 58:A153.

797. Master RJ: Pneumocystis pneumonia in a prepaid care system caring for a Medicaid-covered population with AIDS. Journal of Ambulatory Care Management 1996, 19(1):38-42; discussion 42-35.

798. Master RJ, Cohen C, Cotton DJ: Pneumocystis pneumonia in a prepaid care system caring for a Medicaid- covered population with AIDS. Journal of Ambulatory Care Management 1996, 19(1):38-45.

799. Mathew TA, Yanov SA, Mazitov R, Mishustin SP, Strelis AK, Yanova GV, Golubchikova VT, Taran DV, Golubkov A, Shields AL et al: Integration of alcohol use disorders identification and management in the tuberculosis programme in Tomsk Oblast, Russia. European Journal of Public Health 2009, 19(1):16-18.

800. Matlosz B, Firlag-Burkacka E, Horban A, Kowalska JD: Nephrology consultations incorporated into HIV care-non-compliance is an important issue. AIDS Care - Psychological and Socio-Medical Aspects of AIDS/HIV 2017, 29(2):226-230.

801. Matthews AM, Huckans MS, Blackwell AD, Hauser P: Hepatitis C testing and infection rates in bipolar patients with and without comorbid substance use disorders. Bipolar Disorders 2008, 10(2):266-270.

802. Maurer MA, Gilson AM, Ryan KM, Cleary JF: Consumption of strong opioids for palliative care in europe: The influence of country-level characteristics. Palliative Medicine 2012, 26 (4):557.

803. Mauskopf J, Turner BJ, Markson LE, Houchens RL, Fanning TR, McKee L: Patterns of ambulatory care for AIDS patients, and association with emergency room use. Health Services Research 1994, 29(4):489-510.

804. Mauss S, Berger F, Goelz J, Jacob B, Schmutz G: A prospective controlled study of interferon-based therapy of chronic hepatitis C in patients on methadone maintenance. Hepatology 2004, 40(1):120-124.

805. Mauss S, Berger F, Goelz J, Jacob B, Schmutz G: A prospective controlled study of interferon-based therapy of chronic hepatitis C in patients on methadone maintenance. Hepatology 2004, 40(1):120-124.

806. Mayaud C: [Synthesis of recommendations for the management of HIV-infected patients]. Rev Pneumol Clin 1993, 49(2):61-64.

807. Mayer K, Appelbaum J, Rogers T, Lo W, Bradford J, Boswell S: The evolution of the Fenway Community Health model. American Journal of Public Health 2001, 91(6):892-894.

808. Mayer KH, Grinsztejn B, El-Sadr WM: Transgender People and HIV Prevention: What We Know and What We Need to Know, a Call to Action. Journal of acquired immune deficiency syndromes (1999) 2016, 72:S207-S209.

809. Mayer KH, Meyer JP, Althoff AL, Altice FL: Optimizing care for HIV-Infected people who use drugs: Evidence-based approaches to overcoming healthcare disparities. Clinical Infectious Diseases 2013, 57(9):1309-1317.

810. Mayer KH, Skeer MR, O'Cleirigh C, Goshe BM, Safren SA: Factors associated with amplified HIV transmission behavior among American men who have sex with men engaged in care: implications for clinical providers. Annals of behavioral medicine : a publication of the Society of Behavioral Medicine 2014, 47(2):165-171.

811. Mays M, Leiner S: Pharmacologic management of common lower respiratory tract disorders in women. Journal of nurse-midwifery 1997, 42(3):163-175.

812. McCarty D, LaPrade J, Botticelli M: Substance abuse treatment and HIV services: Massachusetts' policies and programs. Journal of Substance Abuse Treatment 1996, 13(5):429-438; discussion 439.

813. McColloster P, Neff NE: Outpatient management of tuberculosis. Am Fam Physician 1996, 53(5):1579-1594.

814. McCombe G, Cullen W, Almaazmi B, Lambert JS, Avramovic G, Murphy C, O'Connor M, Perry N, Ianache I, Kosa A et al: Integrating primary and secondary care to optimise hepatitis C treatment: Development and evaluation of a Multidisciplinary Educational 'Masterclass' Series. Journal of Viral Hepatitis 2018, 25 (Supplement 2):170-171.

815. McCombe G, Swan D, O'Connor E, Avramovic G, Vickerman P, Ward Z, Surey J, Macias J, Lambert JS, Cullen W: Integrated Hepatitis C Care for People Who Inject Drugs (Heplink): Protocol for a Feasibility Study in Primary Care. JMIR Res Protoc 2018, 7(6):e149.

816. McCombs JS, Yuan Y, Shin J, Saab S: Economic burden associated with patients diagnosed with hepatitis c. Clinical Therapeutics 2011, 33(9):1268-1280.

817. McCormick PA, Keavney M, O'Toole S, Moloney J: Methadone and HCV treatment. Irish medical journal 2008, 101(10):316-317.

818. McDonald SA, Hutchinson SJ, Bird SM, Robertson C, Mills PR, Dillon JF, Goldberg DJ: Hospitalisation for an alcohol-related cause among injecting drug users in Scotland: Increased risk following diagnosis with hepatitis C infection. International Journal of Drug Policy 2011, 22(1):63-69.

819. McDonell MG, Leickly E, McPherson S, Skalisky J, Srebnik D, Angelo F, Vilardaga R, Nepom JR, Roll JM, Ries RK: A Randomized Controlled Trial of Ethyl Glucuronide-Based Contingency Management for Outpatients With Co-Occurring Alcohol Use Disorders and Serious Mental Illness.[Erratum appears in Am J Psychiatry. 2017 Jun 1;174(6):604; PMID: 28565953]. American Journal of Psychiatry 2017, 174(4):370-377.

820. McDonell MG, Leickly E, McPherson S, Skalisky J, Srebnik D, Angelo F, Vilardaga R, Nepom JR, Roll JM, Ries RK: A Randomized Controlled Trial of Ethyl Glucuronide-Based Contingency Management for Outpatients With Co-Occurring Alcohol Use Disorders and Serious Mental Illness. American Journal of Psychiatry 2017, 174(4):370-377.

821. McGarrigle CA, Nicoll A: Prevalence of HIV-1 among attenders at sexually transmitted disease clinics: analyses according to country of birth. Sexually Transmitted Infections 1998, 74(6):415-420.

822. McGovern JJ, Guida F, Corey P: Improved health and self-esteem among patients with AIDS in a therapeutic community nursing program. Journal of Substance Abuse Treatment 2002, 23(4):437-440.

823. McKay JR, McLellan AT, Alterman AI, Cacciola JS, Rutherford MJ, O'Brien CP: Predictors of participation in aftercare sessions and self-help groups following completion of intensive outpatient treatment for substance abuse. J 1998, 59(2):152-162.

824. McKetin R, Dunlop AJ, Holland RM, Sutherland RA, Baker AL, Salmon AM, Hudson SL: Treatment outcomes for methamphetamine users receiving outpatient counselling from the Stimulant Treatment Program in Australia. Drug and Alcohol Review 2013, 32(1):80-87.

825. McKinnon K, Cournos F, Herman R, Satriano J, Silver BJ, Puello I: AIDS-related services and training in outpatient mental health care agencies in New York. Psychiatric Services 1999, 50(9):1225-1228.

826. McKinnon K, Rosner J: Severe mental illness and HIV-AIDS. New Directions for Mental Health Services 2000(87):69-76.

827. McKinnon K, Wainberg ML, Cournos F: HIV/AIDS preparedness in mental health care agencies with high and low substance use disorder caseloads. Journal of Substance Abuse 2001, 13(1-2):127-135.

828. McLaughlin TJ, Soumerai SB, Weinrib D, Aupont O, Cotton D: The association between primary source of ambulatory care and access to and outcomes of treatment among AIDS patients. International Journal for Quality in Health Care 1999, 11(4):293-300.

829. Meade CS, Hansen NB, Kochman A, Sikkema KJ: Utilization of medical treatments and adherence to antiretroviral therapy among HIV-positive adults with histories of childhood sexual abuse. AIDS Patient Care STDS 2009, 23(4):259-266.

830. Meade CS, Sikkema KJ: Psychiatric and psychosocial correlates of sexual risk behavior among adults with severe mental illness. Community Mental Health Journal 2007, 43(2):153-169.

831. Medaglia JD, VanKirk KK, Oswald CB, Church LW: Interdisciplinary Differential Diagnosis and Care of a Patient with Atypical Delusional Parasitosis due to early HIV-related Dementia. Clin Neuropsychol 2015, 29(4):559-569.

832. Mehta SH, Genberg BL, Astemborski J, Kavasery R, Kirk GD, Vlahov D, Strathdee SA, Thomas DL: Limited uptake of hepatitis C treatment among injection drug users. Journal of Community Health 2008, 33(3):126-133.

833. Mellins CA, Havens JF, McDonnell C, Lichtenstein C, Uldall K, Chesney M, Santamaria EK, Bell J: Adherence to antiretroviral medications and medical care in HIV-infected adults diagnosed with mental and substance abuse disorders. AIDS Care 2009, 21(2):168-177.

834. Mendive Arbeloa JM: [Treatment of drug dependence at the health center: 10 years' experience]. Aten Primaria 1997, 20(4):159-160.

835. Merlin J, Long DM, Becker W, Cachay ER, Christopoulos KA, Crane HM, Edelman EJ, Harding R, Liebschutz J, Mathews WC et al: The association of pain and long-term opioid therapy with HIV treatment outcomes. Topics in Antiviral Medicine 2018, 26 (Supplement 1):332s.

836. Merlin JS, Cen L, Praestgaard A, Turner M, Obando A, Alpert C, Woolston S, Casarett D, Kostman J, Gross R et al: Pain and physical and psychological symptoms in ambulatory HIV patients in the current treatment era. J Pain Symptom Manage 2012, 43(3):638-645.

837. Merlin JS, Westfall AO, Heath SL, Goodin BR, Stewart JC, Sorge RE, Younger J: Brief Report: IL-1beta Levels Are Associated with Chronic Multisite Pain in People Living with HIV. Journal of Acquired Immune Deficiency Syndromes 2017, 75(4):e99-e103.

838. Merlin JS, Westfall AO, Heath SL, Goodin BR, Stewart JC, Sorge RE, Younger J: IL-1beta levels are associated with chronic multisite pain in people living with HIV. Journal of Acquired Immune Deficiency Syndromes 2017.

839. Merlin JS, Westfall AO, Raper JL, Zinski A, Norton WE, Willig JH, Gross R, Ritchie CS, Saag MS, Mugavero MJ: Pain, mood, and substance abuse in HIV: implications for clinic visit utilization, antiretroviral therapy adherence, and virologic failure.[Erratum appears in J Acquir Immune Defic Syndr. 2013 May 1;63(1):e38-9]. J Acquir Immune Defic Syndr 2012, 61(2):164-170.

840. Merrill J, Nance R, Hutton H, McCaul M, Chander G, Mayer K, Mugavero M, Mimiaga M, Willig J, Burkholder G et al: Alcohol use predicts antiretroviral adherence independent of illicit drug use in a national HIV clinical cohort. Alcoholism: Clinical and Experimental Research 2015, 39:200A.

841. Metsch L, Feaster D, Gooden L, Root C, Castellon PC, Colasanti J, Farber EW, Cardenas G, Liguori T, Armstrong WS et al: Integrated cocaine & mental health treatment with navigation rct for HIV+ outpatients. Topics in Antiviral Medicine 2018, 26 (Supplement 1):494s.

842. Metzger DS, Navaline H: HIV Prevention among Injection Drug Users: The Need for Integrated Models. Journal of Urban Health 2003, 80(4 SUPPL. 3):iii59-iii66.

843. Meyer I, Empfield M, Engel D, Cournos F: Characteristics of HIV-positive chronically mentally ill inpatients. Psychiatr Q 1995, 66(3):201-207.

844. Mgbemena O, Westfall A, Hicks J, Saag M, Raper J, Overton E, Norton W, Merlin J: Evaluation of a pilot physical therapy program for HIV-infected patients with chronic pain. Journal of Pain 2014, 1):S100.

845. Michels, II, Stover H: Harm reduction--from a conceptual framework to practical experience: the example of Germany. Substance Use & Misuse 2012, 47(8-9):910-922.

846. Michels II, Stover H: Harm reduction--from a conceptual framework to practical experience: the example of Germany. Substance use & misuse 2012, 47(8-9):910-922.

847. Michlig GJ, Westergaard RP, Lam Y, Ahmadi A, Kirk GD, Genz A, Keruly J, Hutton H, Surkan PJ: Avoidance, meaning and grief: psychosocial factors influencing engagement in HIV care. AIDS Care - Psychological and Socio-Medical Aspects of AIDS/HIV 2018, 30(4):511-517.

848. Midde NM, Yuan Y, Quizon PM, Sun WL, Huang X, Zhan CG, Zhu J: Mutations at Tyrosine 88, Lysine 92 and Tyrosine 470 of Human Dopamine Transporter Result in an Attenuation of HIV-1 Tat-Induced Inhibition of Dopamine Transport. Journal of Neuroimmune Pharmacology 2015, 10(1):122-135.

849. Miller LJ, Finnerty M: Sexuality, pregnancy, and childrearing among women with schizophrenia-spectrum disorders. Psychiatric Services 1996, 47(5):502-506.

850. Miller M, Hagan E: Integrated biological-behavioural surveillance in pandemic-threat warning systems. Bulletin of the World Health Organization 2017, 95(1):62-68.

851. Miller V: Buprenorphine and HIV primary care: Report of a Forum for Collaborative HIV Research Workshop. Clinical Infectious Diseases 2006, 43(SUPPL. 4):S254-S257.

852. Miller V, Forum for Collaborative HIVRWP, Steering Committee M: Buprenorphine and HIV primary care: report of a forum for collaborative HIV research workshop. Clinical Infectious Diseases 2006, 43 Suppl 4:S254-257.

853. Miller WC, Hoffman I, Hanscom B, Ha TV, Dumchev K, Djoerban Z, Rose SM, Lancaster KE, Go V, Reifeis S et al: Impact of systems navigation and counseling on ART, SUT and death in PWID: HPTN 074. Topics in Antiviral Medicine 2018, 26 (Supplement 1):506s.

854. Millner L, Widerman E: Women's health issues: a review of the current literature in the social work journals, 1985-1992. Social Work in Health Care 1994, 19(3-4):145-172.

855. Mills S, Saidel T, Magnani R, Brown T: Surveillance and modelling of HIV, STI, and risk behaviours in concentrated HIV epidemics. Sexually Transmitted Infections 2004, 80(SUPPL. 2):ii57-ii62.

856. Mimiaga MJ, Biello K, Reisner SL, Crane HM, Wilson J, Grasso C, Kitahata MM, Mathews WC, Mayer KH, Safren SA: Latent class profiles of internalizing and externalizing psychosocial health indicators are differentially associated with sexual transmission risk: Findings from the cfar network of integrated clinical systems (CNICS) cohort study of HIV-infected men engaged in primary care in the United States. Health Psychology 2015, 34(9):951-959.

857. Mimiaga MJ, Reisner SL, Grasso C, Crane HM, Safren SA, Kitahata MM, Schumacher JE, Mathews WC, Mayer KH: Substance use among HIV-infected patients engaged in primary care in the

United States: findings from the Centers for AIDS Research Network of Integrated Clinical Systems cohort. American journal of public health 2013, 103(8):1457-1467.

858. Mimiaga MJ, Reisner SL, Vanderwarker R, Gaucher MJ, O'Connor CA, Medeiros MS, Safren SA: Polysubstance use and HIV/STD risk behavior among Massachusetts men who have sex with men accessing Department of Public Health mobile van services: implications for intervention development. AIDS Patient Care STDS 2008, 22(9):745-751.

859. Misko AN, Nelson DL, Duggan JM: Three case studies of community occupational therapy for individuals with human immunodeficiency virus. Occupational Therapy in Health Care 2015, 29(1):11-26.

860. Mistler LA, Brunette MF, Rosenberg SD, Vidaver RM, Luckoor R, Iber M: Case report of 3 patients with severe mental illness and chronic hepatitis C virus infection treated with interferon-alpha. Int J Psychiatry Med 2006, 36(4):449-455.

861. Mitchell SG, Gryczynski J, Schwartz RP, O'Grady KE, Olsen YK, Jaffe JH: A randomized trial of intensive outpatient (IOP) vs. standard outpatient (OP) buprenorphine treatment for African Americans.[Erratum appears in Drug Alcohol Depend. 2016 Nov 1;168:340; PMID: 27665675]. Drug Alcohol Depend 2013, 128(3):222-229.

862. Mitchell SG, Gryczynski J, Schwartz RP, O'Grady KE, Olsen YK, Jaffe JH: A randomized trial of intensive outpatient (IOP) vs. standard outpatient (OP) buprenorphine treatment for African Americans. Drug Alcohol Depend 2013, 128(3):222-229.

863. Mitchell SG, Schwartz RP, Kirk AS, Dusek K, Oros M, Hosler C, Gryczynski J, Barbosa C, Dunlap L, Lounsbury D et al: SBIRT Implementation for Adolescents in Urban Federally Qualified Health Centers. Journal of Substance Abuse Treatment 2016, 60:81-90.

864. Mitchison DA: The diagnosis and therapy of tuberculosis during the past 100 years. American Journal of Respiratory and Critical Care Medicine 2005, 171(7):699-706.

865. Mizuno Y, Wilkinson JD, Santibanez S, Dawson Rose C, Knowlton A, Handley K, Gourevitch MN, Inspire T: Correlates of health care utilization among HIV-seropositive injection drug users. AIDS Care 2006, 18(5):417-425.

866. Moatti JP, Souville M, Escaffre N, Obadia Y: French general practitioners' attitudes toward maintenance drug abuse treatment with buprenorphine. Addiction 1998, 93(10):1567-1575.

867. Mokri A, Chawarski MC, Taherinakhost H, Schottenfeld RS: Medical treatments for opioid use disorder in Iran: a randomized, double-blind placebo-controlled comparison of buprenorphine/naloxone and naltrexone maintenance treatment. Addiction (Abingdon, England) 2016, 111(5):874-882.

868. Momplaisir F, Long JA, Badolato G, Brady KA: The role of primary care physicians in improving colorectal cancer screening in patients with HIV. Journal of General Internal Medicine 2012, 27(8):940-944.

869. Monroe AK, Lau B, Mugavero MJ, Mathews WC, Mayer KH, Napravnik S, Hutton HE, Kim HS, Jabour S, Moore RD et al: Heavy alcohol use is associated with worse retention in HIV care. Journal of Acquired Immune Deficiency Syndromes 2016, 73(4):419-425.

870. Moody AL, Morgello S, Gerits P, Byrd D: Vulnerabilities and caregiving in an ethnically diverse HIV-infected population. Aids Behav 2009, 13(2):337-347.

871. Morano JP, Walton MR, Zelenev A, Bruce RD, Altice FL: Latent tuberculosis infection: screening and treatment in an urban setting. Journal of Community Health 2013, 38(5):941-950.

872. Morano JP, Zelenev A, Lombard A, Marcus R, Gibson BA, Altice FL: Strategies for hepatitis C testing and linkage to care for vulnerable populations: point-of-care and standard HCV testing in a mobile medical clinic. Journal of Community Health 2014, 39(5):922-934.

873. Morano JP, Zelenev A, Walton MR, Bruce RD, Altice FL: Latent tuberculosis infection screening in foreign-born populations: a successful mobile clinic outreach model. American Journal of Public Health 2014, 104(8):1508-1515.

874. Morasco BJ, Greaves DW, Lovejoy TI, Turk DC, Dobscha SK, Hauser P: Development and preliminary evaluation of an integrated cognitive-behavior treatment for chronic pain and substance use disorder in patients with the hepatitis C virus. Pain Medicine (United States) 2016, 17(12):2280-2290.

875. Morgan TR, Elnahal S, Gonzalez R, Park AM, Lukesh W, Yakovchenko V, Belperio PS, Chartier M, Rogal S, Ross D: Hepatitis C care in the department of veterans affairs: A system-wide process improvement project. Hepatology 2017, 66 (Supplement 1):7A.

876. Morin M, Obadia Y, Moatti JP, Souville M: Commitment, value conflicts and role strains among French GPs in care for HIV positive patients. AIDS Care - Psychological and Socio-Medical Aspects of AIDS/HIV 1995, 7(SUPPL. 1):S79-S84.

877. Morozova O, Dvoriak S, Pykalo I, Altice FL: Primary healthcare-based integrated care with opioid agonist treatment: First experience from Ukraine. Drug and Alcohol Dependence 2017, 173:132-138.

878. Morozova O, Dvoryak S, Altice FL: Methadone treatment improves tuberculosis treatment among hospitalized opioid dependent patients in Ukraine. International Journal of Drug Policy 2013, 24(6):e91-e98.

879. Morris L, Smirnov A, Kvassay A, Leslie E, Kavanagh R, Alexander N, Davey G, Williams O, Gilks C, Najman J: Initial outcomes of integrated community-based hepatitis C treatment for people who inject drugs: Findings from the Queensland Injectors' Health Network. International Journal of Drug Policy 2017, 47:216-220.

880. Morrison RE, Brint JM, Smith WR, Arheart KL, Wray D, Palte SB, Ackerman TF: Appropriate and inappropriate prescribing of narcotics for ambulatory HIV-positive patients. Journal of General Internal Medicine 1994, 9(6):301-305.

881. Moser KM, Sowell RL, Phillips KD: Issues of women dually diagnosed with HIV infection and substance use problems in the Carolinas. Issues Ment Health Nurs 2001, 22(1):23-49.

882. Mostardt S, Hanhoff N, Wasem J, Goetzenich A, Schewe K, Wolf E, Mayr C, Jaeger H, Pfaff H, Dupke S et al: Cost of HIV and determinants of health care costs in HIV-positive patients in Germany: Results of the DAGNA K3A Study. European Journal of Health Economics 2013, 14(5):799-808.

883. Moussalli J, Melin P, Wartelle-Bladou C, Lang JP: [Management of hepatitis C among drug user patients]. Gastroenterol Clin Biol 2007, 31(8-9 Pt 3):4S51-55.

884. Mukambetov A, Sabyrbekova T, Asanalieva L, Sadykov I, Connor SR: Palliative Care Development in Kyrgyzstan. Journal of Pain and Symptom Management 2018, 55(2 Supplement):S41-S45.

885. Mukelabai M: Gender, HIV/AIDS and palliative care in sub-Sahara Africa. Pain Practice 2009, 9:59.

886. Muller MC, Pichler M, Martin G, Plorer D, Winter C, Pogarell O, Bogner JR: [Burden of disease and level of patient's medical care in substitution treatment for opiates]. Med Klin 2009, 104(12):913-917.

887. Munoz-Plaza CE, Strauss SM, Astone-Twerwll JM, Des Jarlais DC, Hagan H: Staff perspectives on facilitating the implementation of hepatitis C services at drug treatment programs. Journal of Psychoactive Drugs 2006, 38(3):233-241.

888. Murphy BS, Branson CE, Francis J, Vaughn GC, Greene A, Kingwood N, Adjei GA: Integrating adolescent substance abuse treatment with HIV services: evidence-based models and baseline descriptions. Journal of evidence-based social work 2014, 11(5):445-459.

889. Murphy K, Edelstein H, Smith L, Clanon K, Schweitzer B, Reynolds L, Wheeler P: Treatment of HIV in outpatients with schizophrenia, schizoaffective disorder and bipolar disease at two county clinics. Community mental health journal 2011, 47(6):668-671.

890. Mustanski B: The Gene, Environment, and Neighborhood Initiative (GENI) to understand a cluster of health outcomes in African American youth. Behavior Genetics 2011, 41 (6):926.

891. Naar-King S, Lam P, Wang B, Wright K, Parsons JT, Frey MA: Brief report: maintenance of effects of motivational enhancement therapy to improve risk behaviors and HIV-related Health in a randomized controlled trial of youth living with HIV. Journal of Pediatric Psychology 2008, 33(4):441-445.

892. Nachega JB, Uthman OA, Del Rio C, Mugavero MJ, Rees H, Mills EJ: Addressing the achilles' heel in the HIV care continuum for the success of a test-and-treat strategy to achieve an AIDS-free generation. Clinical Infectious Diseases 2014, 59(SUPPL.1):S21-S27.

893. Nanney E, Smith S, Hartwig K, Mmbando P: Scaling Up palliative care services in rural Tanzania. Journal of Pain and Symptom Management 2010, 40(1):15-18.

894. Nazzal Z, Sobuh I: Risk factors of hepatitis B transmission in northern Palestine: a case - control study. BMC research notes 2014, 7:190.

895. Nebelkopf E, King J: A holistic system of care for Native Americans in an urban environment. Journal of Psychoactive Drugs 2003, 35(1):43-52.

896. Nebelkopf E, Penagos M: Holistic Native Network: Integrated HIV/AIDS, substance abuse, and mental health services for Native Americans in San Francisco. Journal of Psychoactive Drugs 2005, 37(3):257-264.

897. Nechaeva OB, Skachkova EI: Causes and factors of development of drug resistance in pulmonary tuberculosis. [Russian]. Problemy tuberkuleza i boleznei legkikh 2003(9):6-9.

898. Nechaeva OB, Skachkova EI: [Causes and factors of development of drug resistance in pulmonary tuberculosis]. Probl 2003(9):6-9.

899. Nelson FL, Kabacy M: Evaluation of an AIDS prevention education program for health service providers working with i.v. drug users. Journal of Substance Abuse 1989, 1(4):467-470.

900. Network HIVR: Hospital and outpatient health services utilization among HIV-infected patients in care in 1999. J Acquir Immune Defic Syndr 2002, 30(1):21-26.

901. Nevens F, Colle I, Michielsen P, Robaeys G, Moreno C, Caekelbergh K, Lamotte M, Wyffels V: Resource use and cost of hepatitis C-related care. European Journal of Gastroenterology and Hepatology 2012, 24(10):1191-1198.

902. Neves L, Reis R, Gir E, Ribeiro P: Adherence to the treatment of individuals with the HIV/tuberculosis co-infection: Integrative review. Retrovirology 2010, 1):P188.

903. Newman A, Beckstead S, Finch S, Knorr T, Lynch C, MacKenzie M, Robertson R, Shore R: Hepatitis C treatment of a marginalized population: A multi-disciplinary primary practitioner-led treatment model in practice. Canadian Journal of Gastroenterology Conference: Canadian Digestive Diseases Week 2009, 23(no pagination).

904. Newman AI, Beckstead S, Beking D, Finch S, Knorr T, Lynch C, MacKenzie M, Mayer D, Melles B, Shore R: Treatment of chronic hepatitis c infection among current and former injection drug users within a multidisciplinary treatment model at a community health centre. Canadian Journal of Gastroenterology 2013, 27(4):217-223.

905. Newman CE, Kippax SC, Mao L, Rogers GD, Saltman DC, Kidd MR: Features of the management of depression in gay men and men with HIV from the perspective of Australian GPs. Fam Pract 2009, 26(1):27-33.

906. Newschaffer CJ, Laine C, Hauck WW, Fanning T, Turner BJ: Clinic characteristics associated with reduced hospitalization of drug users with aids. Journal of Urban Health 1998, 75(1):153-169.

907. Newschaffer CJ, Zhang D, Hauck WW, Fanning T, Turner BJ: Effect of enhanced prenatal and HIV-focused services for pregnant women who are infected by human immunodeficiency virus on emergency department use. Medical care 1999, 37(12):1308-1319.

908. Nguyen Bich D, Korthuis PT, Nguyen Thu T, Van Dinh H, Le Minh G: HIV patients' preference for integrated models of addiction and HIV treatment in Vietnam. Journal of Substance Abuse Treatment 2016, 69:57-63.

909. Nguyen OK, Dore GJ, Kaldor JM, Hellard ME, Committee APS: Recruitment and follow-up of injecting drug users in the setting of early hepatitis C treatment: insights from the ATAHC study. International Journal of Drug Policy 2007, 18(5):447-451.

910. Nikolaev VP, Baisheva NN, Luginova LD: Outpatient observation and treatment of patients with tuberculosis and massive bacterial excretion aggravated by negative social factors. [Russian]. Problemy tuberkuleza 1992(9-10):16-19.

911. Nikolaev VP, Baisheva NN, Luginova LD: [Outpatient observation and treatment of patients with tuberculosis and massive bacterial excretion aggravated by negative social factors]. Problemy Tuberkuleza 1992(9-10):16-19.

912. Noble K, Holden M, Warner G: A solution to improving uptake of hepatitis B immunisation in at risk groups, through collaboration and adopting an integrated approach with pharmacists as service providers. International Journal of Pharmacy Practice 2010, 18:43-44.

913. Nogueira SA: Prevention of vertical transmission of HIV. [Portuguese]. Arquivos Brasileiros de Pediatria 1997, 4(6):157-160.

914. Norman J, Walsh NM, Mugavin J, Stoove MA, Kelsall J, Austin K, Lintzeris N: The acceptability and feasibility of peer worker support role in community based HCV treatment for injecting drug users. Harm Reduction Journal 2008, 5 (no pagination)(8).

915. Norton BL, Beitin A, Glenn M, DeLuca J, Litwin AH, Cunningham CO: Retention in buprenorphine treatment is associated with improved HCV care outcomes. Journal of Substance Abuse Treatment 2017, 75:38-42.

916. Norton BL, Fleming J, Bachhuber MA, Steinman M, DeLuca J, Cunningham CO, Johnson N, Laraque F, Litwin AH: High HCV cure rates for people who use drugs treated with direct acting antiviral therapy at an urban primary care clinic. The International journal on drug policy 2017, 47:196-201.

917. Norton BL, Fleming J, Bachhuber MA, Steinman M, DeLuca J, Cunningham CO, Johnson N, Laraque F, Litwin AH: High HCV cure rates for people who use drugs treated with direct acting antiviral therapy at an urban primary care clinic. The International journal on drug policy 2017, 47:196-201.

918. Noska AJ, Belperio PS, Loomis TP, O'Toole TP, Backus LI: Prevalence of Human Immunodeficiency Virus, Hepatitis C Virus, and Hepatitis B Virus among Homeless and Nonhomeless United States Veterans. Clinical Infectious Diseases 2017, 65(2):252-258.

919. Novak RM, Hart RL, Chmiel JS, Brooks JT, Buchacz K, Investigators HIVOS: Disparities in Initiation of Combination Antiretroviral Treatment and in Virologic Suppression Among Patients in the HIV Outpatient Study, 2000-2013. J Acquir Immune Defic Syndr 2015, 70(1):23-32.

920. Novick DM, Kreek MJ: Critical issues in the treatment of hepatitis C virus infection in methadone maintenance patients. Addiction 2008, 103(6):905-918.

921. Novick DM, Kreek MJ: Critical issues in the treatment of hepatitis C virus infection in methadone maintenance patients. Addiction 2008, 103(6):905-918.

922. Novick DM, Kreek MJ: Critical issues in the treatment of hepatitis C virus infection in methadone maintenance patients. Addiction 2008, 103(6):905-918.

923. Ntizimira CR, Ngizwenayo S, Krakauer EL, Dunne ML, Esmaili E: Addressing End-of-Life Care in Cancer Patients through "Ubuntu": Lessons Learned from Rwanda in Global Health Perspective of Humanity. Current Obstetrics and Gynecology Reports 2016, 5(4):273-278.

924. Nyamathi AM, Christiani A, Windokun F, Jones T, Strehlow A, Shoptaw S: Hepatitis C virus infection, substance use and mental illness among homeless youth: A review. Aids 2005, 19(SUPPL. 3):S34-S40.

925. Obadia Y, Perrin V, Feroni I, Vlahov D, Moatti JP: Injecting misuse of buprenorphine among French drug users. Addiction 2001, 96(2):267-272.

926. O'Connor CA, Patsdaughter CA, Grindel CG, Taveira PF, Steinberg JL: A mobile HIV education and testing program: bringing services to hard-to-reach populations. AIDS Patient Care STDS 1998, 12(12):931-937.

927. O'Dowd MA, Biderman DJ, McKegney FP: Incidence of suicidality in AIDS and HIV-positive patients attending a psychiatry outpatient program. Psychosomatics 1993, 34(1):33-40.

928. Odum AL, Madden GJ, Badger GJ, Bickel WK: Needle sharing in opioid-dependent outpatients: psychological processes underlying risk. Drug Alcohol Depend 2000, 60(3):259-266.

929. Oertle D, Edelmann R, Ostewalder J, Vernazza PL, Galeazzi RL: [HIV prevention in HIV-positive drug addicts. A methadone-supported model]. Schweiz Med Wochenschr 1993, 123(48):2284-2292.

930. Ogbuagu O, Bruce RD: Reaching the Unreached: Treatment as Prevention as a Workable Strategy to Mitigate HIV and Its Consequences in High-Risk Groups. Current HIV/AIDS Reports 2014, 11(4):505-512.

931. Oji VU, Hung LC, Abbasgholizadeh R, Hamilton FT, Essien EJ, Nwulia E: Spiritual care may impact mental health and medication adherence in HIV+ populations. HIV/AIDS - Research and Palliative Care 2017, 9:101-109.

932. Oji VU, Hung LC, Abbasgholizadeh R, Terrell Hamilton F, Essien EJ, Nwulia E: Spiritual care may impact mental health and medication adherence in HIV+ populations. HIV AIDS (Auckl) 2017, 9:101-109.

933. Oldfield B, Munoz N, Boshnack N, Leavitt R, McGovern M, Villanueva M, Tetrault JM, Edelman EJ: Consumer, provider, and organizational leadership perspectives on integrated care for opioid use disorder and hiv: A community-engaged, qualitative study. Journal of General Internal Medicine 2018, 33 (2 Supplement 1):149.

934. Oliveira PR, Felix CS, Carvalho VC, Giovani AM, Reis RS, Beraldo M, Albuquerque EP, Ferreira WC, Silva JS, Lima ALL: Outpatient parenteral antimicrobial therapy for orthopedic infections - a successful public healthcare experience in Brazil. Brazilian Journal of Infectious Diseases 2016, 20(3):272-275.

935. O'Neill JF, Romaguera R, Parham D, Marconi K: Practicing palliative care in resource-poor settings. Journal of Pain and Symptom Management 2002, 24(2):148-151.

936. Onstad GD, Sbarbaro JA, Rotenberg L: Posthospital chemotherapy of the unreliable patient. The American review of respiratory disease 1970, 101(2):258-264.

937. Oosterhoff P, Hardon AP, Nguyen TA, Pham NY, Wright P: Dealing with a positive result: routine HIV testing of pregnant women in Vietnam. AIDS Care 2008, 20(6):654-659.

938. Orchard T, Salters K, Michelow W, Lepik K, Palmer A, Hogg R: "My job is to deal with what I can": HIV care providers' perspectives on adherence to HAART, addictions, and comprehensive care delivery in Vancouver, British Columbia. Critical Public Health 2016, 26(5):542-553.

939. Ordean A, Kahan M, Graves L, Abrahams R, Boyajian T: Integrated care for pregnant women on methadone maintenance treatment: Canadian primary care cohort study. Canadian Family Physician 2013, 59(10):e462-e469.

940. O'Rourke A, Ruiz MS, Allen ST: Client-Identified needs and agency-provided services at a harm reduction community based organization in the District of Columbia. Harm Reduction Journal 2015, 12 (1) (no pagination)(17).

941. Orr DA, Pinto PF: The clinical management of HIV-related dementia and other memory disorders in the residential drug treatment environment. Journal of Substance Abuse Treatment 1993, 10(6):505-511.

942. Orwat J, Saitz R, Tompkins CP, Cheng DM, Dentato MP, Samet JH: Substance abuse treatment utilization among adults living with HIV/AIDS and alcohol or drug problems. Journal of Substance Abuse Treatment 2011, 41(3):233-242.

943. Oser CB, Tindall MS, Leukefeld CG: HIV testing in correctional agencies and community treatment programs: the impact of internal organizational structure. Journal of Substance Abuse Treatment 2007, 32(3):301-310.

944. Oshri A, Tubman JG, Wagner EF, Leon-Morris S, Snyders J: Psychiatric symptom patterns, proximal risk factors, and sexual risk behaviors among youth in outpatient substance abuse treatment. The American journal of orthopsychiatry 2008, 78(4):430-441.

945. O'Toole TP, Pollini R, Gray P, Jones T, Bigelow G, Ford DE: Factors identifying high-frequency and low-frequency health service utilization among substance-using adults. Journal of Substance Abuse Treatment 2007, 33(1):51-59.

946. Pace CA, Tsui JI, Nader C, Bergeron LP, LaBelle CT, Samet JH: Integrating care for hepatitis C virus infection with office-based therapy for opioid dependence. Journal of General Internal Medicine 2014, 29:S488.

947. Painter RG, Lanson Jr NA, Jin Z, Park F, Wang G: Conditional expression of a suicide gene by the telomere reverse transcriptase promoter for potential post-therapeutic deletion of tumorigenesis. Cancer Science 2005, 96(9):607-613.

948. Painter RG, Lanson NA, Jr., Jin Z, Park F, Wang G: Conditional expression of a suicide gene by the telomere reverse transcriptase promoter for potential post-therapeutic deletion of tumorigenesis. Cancer Science 2005, 96(9):607-613.

949. Palacio H, Shiboski CH, Yelin EH, Hessol NA, Greenblatt RM: Access to and utilization of primary care services among HIV-infected women. Journal of Acquired Immune Deficiency Syndromes and Human Retrovirology 1999, 21(4):293-300.

950. Palepu A, Cheng DM, Kim T, Nunes D, Vidaver J, Alperen J, Saitz R, Samet JH: Substance abuse treatment and receipt of liver specialty care among persons coinfected with HIV/HCV who have alcohol problems. Journal of Substance Abuse Treatment 2006, 31(4):411-417.

951. Pantel ES: The health-care needs of AIDS patients: parallels with the elderly. AIDS Public Policy J 1991, 6(2):83-87.

952. Park H, Klausner J, Konda KA, Caceres CF, Brown B, Moreno GMC, Leon S, Rivera SKV: Recent syphilis infection among high-risk men who have sex with men (MSM) in Lima, Peru. Sex Transm Dis 2014, 1):S85.

953. Parker KA, Koumans EH, Hawkins RV, Massanga M, Somse P, Barker K, Moran J: Providing low-cost sexually transmitted diseases services in two semi-urban health centers in Central African Republic (CAR): characteristics of patients and patterns of health care-seeking behavior. Sex Transm Dis 1999, 26(9):508-516.

954. Patel MJ, Salahuddin N, Kashif W, Riaz M, Tariq M, Samdani AJ, Khan MS, Ayaz SI, Sorathia A, Furqan M: Preventive medicine practices by primary care providers in Karachi. Journal of the College of Physicians and Surgeons Pakistan 2008, 18(3):193-194.

955. Patel V, Araya R, Chatterjee S, Chisholm D, Cohen A, De Silva M, Hosman C, McGuire H, Rojas G, van Ommeren M: Treatment and prevention of mental disorders in low-income and middle-income countries. Lancet 2007, 370(9591):991-1005.

956. Patterson S, Salters KA, Zhang W, Chen Y, Money D, Hogg R, Kaida A: Contraceptive use and method preference among Harder-to-reach women on antiretroviral therapy (ART) living with HIV in British Columbia (BC). Canadian Journal of Infectious Diseases and Medical Microbiology 2013, 24:34A.

957. Paul JP, Barrett DC, Crosby GM, Stall RD: Longitudinal changes in alcohol and drug use among men seen at a gay-specific substance abuse treatment agency. J 1996, 57(5):475-485.

958. Pauly MP, Russell M, Moore C, Chia C, Edwards JF, Cunanan R, Menenberg SR, Witt GL: The impact of lifetime drug use on hepatitis c treatment outcomes in privately insured members of an integrated health care plan. Hepatology 2012, 56:655A.

959. Pauly MP, Russell M, Moore C, Chia C, Menenberg S, Witt GL, Martin SE, Ruebner BH: The impact of chemical dependency diagnosis and treatment on treatment of chronic hepatitis C (HCV) in patients in a stable insured population. Hepatology 2009, 50:726A.

960. Pelet A, Doll S, Huissoud T, Resplendino J, Besson J, Favrat B: Methadone maintenance treatment in the Swiss Canton of Vaud: demographic and clinical data on 1,782 ambulatory patients. Eur Addict Res 2005, 11(2):99-106.

961. Pellecchia K, Roeschlein A, Lewis J, Zuniga M: Conjoint Treatment: A Novel Approach to Target the Syndemic Conditions of Trauma, Substance Abuse, and HIV in Women Living with HIV. South Med J 2017, 110(11):705-708.

962. Perez-Gomez A: The ambulatory treatment of noncompulsive users of psychoactive substances. Journal of Substance Abuse Treatment 1993, 10(3):317-321.

963. Perry BA, Westfall AO, Molony E, Tucker R, Ritchie C, Saag MS, Mugavero MJ, Merlin JS: Characteristics of an ambulatory palliative care clinic for HIV-infected patients. Journal of Palliative Medicine 2013, 16(8):934-937.

964. Perz JF, Grytdal S, Beck S, Fireteanu AM, Poissant T, Rizzo E, Bornschlegel K, Thomas A, Balter S, Miller J et al: Case-control study of hepatitis B and hepatitis C in older adults: Do healthcare exposures contribute to burden of new infections? Hepatology 2013, 57(3):917-924.

965. Petrenko VM, Egorova OB, Iukhno NI, Klochko LT: Incidence of concomitant diseases in patients with pulmonary tuberculosis living in a rural area and characteristics of their outpatient treatment. [Russian]. Vrachebnoe delo 1987(11):84-85.

966. Petrenko VM, Egorova OB, Iukhno NI, Klochko LT: [Incidence of concomitant diseases in patients with pulmonary tuberculosis living in a rural area and characteristics of their outpatient treatment]. Vrachebnoe Delo 1987(11):84-85.

967. Petry NM, Tawfik Z: Comparison of problem-gambling and non-problem-gambling youths seeking treatment for marijuana abuse. J Am Acad Child Adolesc Psychiatry 2001, 40(11):1324-1331.

968. Piana C, Danhof M, Della Pasqua OE: Impact of non-adherence to antiretroviral therapy in HIV-infected children. Basic and Clinical Pharmacology and Toxicology 2011, 109:71.

969. Piccirillo M, Sardo M, Gnarini R: The diagnostic importance of the bone marrow aspirate in the diagnosis of Visceral Leishmaniasis in patients with HIV infection. Infection 2010, 38:71.

970. Piette J, Fleishman JA, Mor V, Dill A: A comparison of hospital and community case management programs for persons with AIDS. Medical care 1990, 28(8):746-755.

971. Piette JD, Fleishman JA, Stein MD, Mor V, Mayer K: Perceived needs and unmet needs for formal services among people with HIV disease. Journal of Community Health 1993, 18(1):11-23.

972. Piette JD, Mor V, Mayer K, Zierler S, Wachtel T: The effects of immune status and race on health service use among people with HIV disease. American Journal of Public Health 1993, 83(4):510-514.

973. Pizzo PA, Eddy J, Faloon J: Acquired immune deficiency syndrome in children. Current problems and therapeutic considerations. American Journal of Medicine 1988, 85(2A):195-202.

974. Poeta J, Linden R, Antunes MV, Real L, Menezes AM, Ribeiro JP, Sprinz E: Plasma concentrations of efavirenz are associated with body weight in HIV-positive individuals. Journal of Antimicrobial Chemotherapy 2011, 66(11):2601-2604.

975. Poisson-Salomon AS, Mayaud C, Blum-Boisgard C: [Medical and social factors in the orientation of patients with AIDS after discharge]. Rev Epidemiol Sante Publique 1995, 43(1):37-47.

976. Poisson-Salomon AS, Mayaud C, Blum-Boisgard C, Rozenbaum W, Delfraissy JF, Katlama C, Bary M, Boue F, Veber F, Joyeux A et al: Post-discharge care patterns for AIDS patients: Medical and social factors influencing the choice of home care or health-care institutions. [French]. Revue d'Epidemiologie et de Sante Publique 1995, 43(1):37-47.

977. Pokhrel KN, Sharma VD, Pokhrel KG, Neupane SR, Mlunde LB, Poudel KC, Jimba M: Investigating the impact of a community home-based care on mental health and anti-retroviral therapy adherence in people living with HIV in Nepal: A community intervention study. BMC Infectious Diseases 2018, 18 (1) (no pagination)(263).

978. Pollack HA, D'Aunno T: HIV testing and counseling in the nation's outpatient substance abuse treatment system, 1995-2005. Journal of Substance Abuse Treatment 2010, 38(4):307-316.

979. Pollack HA, D'Aunno T, Lamar B: Outpatient substance abuse treatment and HIV prevention: an update. Journal of Substance Abuse Treatment 2006, 30(1):39-47.

980. Pollack HA, Khoshnood K, Blankenship KM, Altice FL: The impact of needle exchange-based health services on emergency department use. Journal of General Internal Medicine 2002, 17(5):341-348.

981. Powell-Cope GM, Brown MA: Going public as an AIDS family caregiver. Social Science and Medicine 1992, 34(5):571-580.

982. Power A, Duncan N, Goodlad C: Management of the dialysis patient for the hospital physician. Postgraduate Medical Journal 2009, 85(1005):376-381.

983. Pozzi G, Del Borgo C, Del Forno A, Genualdo A, Mannelli P, Fantoni M: Psychological discomfort and mental illness in patients with AIDS: implications for home care. AIDS Patient Care STDS 1999, 13(9):555-564.

984. Prince M, Patel V, Saxena S, Maj M, Maselko J, Phillips MR, Rahman A: No health without mental health. Lancet 2007, 370(9590):859-877.

985. Proeschold-Bell RJ, Heine A, Pence BW, McAdam K, Quinlivan EB: A cross-site, comparative effectiveness study of an integrated HIV and substance use treatment program. AIDS Patient Care and STDs 2010, 24(10):651-658.

986. Proeschold-Bell RJ, Patkar AA, Naggie S, Coward L, Mannelli P, Yao J, Bixby P, Muir AJ: An Integrated alcohol abuse and medical treatment model for patients with hepatitis c. Digestive Diseases and Sciences 2012, 57(4):1083-1091.

987. Pyne JM: Expanding the Scope of Integrated Behavioral Health Care for Patients With Hepatitis C Virus. Clin Gastroenterol Hepatol 2015, 13(11):2015-2016.

988. Qiu S, Pan H, Zhang S, Peng X, Zheng X, Xu G, Wang M, Wang J, Lu H: Is tuberculosis treatment really free in China? A study comparing two areas with different management models. PloS one 2015, 10(5):e0126770.

989. Quanbeck A, Gustafson DH, Marsch LA, Chih MY, Kornfield R, McTavish F, Johnson R, Brown RT, Mares ML, Shah DV: Implementing a Mobile Health System to Integrate the Treatment of Addiction Into Primary Care: A Hybrid Implementation-Effectiveness Study. J Med Internet Res 2018, 20(1):e37.

990. Quizon PM, Yuan Y, Sun WL, Strauss MJ, Zhu Y, Zhou Y, Midde NM, Zhan CG, Zhu J: Mutation of human dopamine transporter aspartic acid206 displays a neutral effect on basal dopamine transport and attenuates Tat-induced inhibition of transport function. Journal of Neuroimmune Pharmacology 2017, 12 (1 Supplement 1):S39-S40.

991. RachBeisel J, Scott J, Dixon L: Co-occurring severe mental illness and substance use disorders: a review of recent research. Psychiatric Services 1999, 50(11):1427-1434.

992. Raehl CL, Bond CA, Pitterle ME: Clinical pharmacy services in hospitals educating pharmacy students. Pharmacotherapy 1998, 18(5):1093-1102.

993. Ralston JD, Silverberg MJ, Grothaus L, Leyden WA, Ross T, Stewart C, Carzasty S, Horberg M, Catz SL: Use of web-based shared medical records among patients with HIV. American Journal of Managed Care 2013, 19(4):e114-e124.

994. Rance J, Newland J, Hopwood M, Treloar C: The politics of place(ment): Problematising the provision of hepatitis C treatment within opiate substitution clinics. Social Science and Medicine.

995. Rance J, Newland J, Hopwood M, Treloar C: The politics of place(ment): problematising the provision of hepatitis C treatment within opiate substitution clinics. Soc Sci Med 2012, 74(2):245-253.

996. Randall HB, Alhamad T, Schnitzler MA, Zhang Z, Ford-Glanton S, Axelrod DA, Segev DL, Kasiske BL, Hess GP, Yuan H et al: Survival implications of opioid use before and after liver transplantation. Liver Transplantation 2017, 23(3):305-314.

997. Rasbach DA, Desruisseau AJ, Kipp AM, Stinnette S, Kheshti A, Shepherd BE, Sterling TR, Hulgan T, McGowan CC, Qian HZ: Active cocaine use is associated with lack of HIV-1 virologic suppression independent of nonadherence to antiretroviral therapy: use of a rapid screening tool during routine clinic visits. AIDS Care 2013, 25(1):109-117.

998. Rasch RF, Davidson D, Seiters J, MacMaster SA, Adams S, Darby K, Cooper RL: Integrated recovery management model for ex-offenders with co-occurring mental health and substance use disorders and high rates of HIV risk behaviors. Journal of the Association of Nurses in AIDS Care 2013, 24(5):438-448.

999. Rasch RFR, Davidson D, Seiters J, MacMaster SA, Adams S, Darby K, Cooper RL: Integrated recovery management model for ex-offenders with co-occurring mental health and substance use disorders and high rates of hiv risk behaviors. Journal of the Association of Nurses in AIDS Care 2013, 24(5):438-448.

1000. Rash CJ, Burki M, Montezuma-Rusca JM, Petry NM: A retrospective and prospective analysis of trading sex for drugs or money in women substance abuse treatment patients. Drug Alcohol Depend 2016, 162:182-189.

1001. Rastegar DA, Fingerhood MI, Jasinski DR: A resident clerkship that combines inpatient and outpatient training in substance abuse and HIV care. Substance Abuse 2004, 25(4):11-15.

1002. Rayner L, Matcham F, Hutton J, Stringer C, Dobson J, Steer S, Hotopf M: Embedding integrated mental health assessment and management in general hospital settings: Feasibility, acceptability and the prevalence of common mental disorder. General Hospital Psychiatry 2014, 36(3):318-324.

1003. Read P, Lothian R, Chronister K, Gilliver R, Kearley J, Dore GJ, van Beek I: Delivering direct acting antiviral therapy for hepatitis C to highly marginalised and current drug injecting populations in a targeted primary health care setting. International Journal of Drug Policy 2017, 47:209-215.

1004. Reichman LB: Report of the task force assigned to consider the future of the New Jersey Hospital for chest diseases at Glen Gardner. Journal of the Medical Society of New Jersey 1975, 72(10):845-850.

1005. Reif SS, Pence BW, LeGrand S, Wilson ES, Swartz M, Ellington T, Whetten K: In-home mental health treatment for individuals with HIV. AIDS Patient Care STDS 2012, 26(11):655-661.

1006. Reimer J, Schmidt CS, Schulte B, Gansefort D, Golz J, Gerken G, Scherbaum N, Verthein U, Backmund M: Psychoeducation improves hepatitis C virus treatment during opioid substitution therapy: a controlled, prospective multicenter trial. Clin Infect Dis 2013, 57 Suppl 2:S97-104.

1007. Reimer J, Schmidt CS, Schulte B, Gansefort D, Golz J, Gerken G, Scherbaum N, Verthein U, Backmund M: Psychoeducation improves hepatitis C virus treatment during opioid substitution therapy: a controlled, prospective multicenter trial. Clin Infect Dis 2013, 57 Suppl 2:S97-104.

1008. Reisner SL, Poteat T, Keatley J, Cabral M, Mothopeng T, Dunham E, Holland CE, Max R, Baral SD: Global health burden and needs of transgender populations: a review. The Lancet 2016, 388(10042):412-436.

1009. Remy AJ, Wenger H, Bouchkira H: [Treatment of chronic hepatitis C in drug users: ethic, successful and useful]. Presse Med 2014, 43(12 Pt 1):1314-1316.

1010. Reynolds EK, Magidson JF, Bornovalova MA, Gwadz M, Ewart CK, Daughters SB, Lejuez CW: Application of the social action theory to understand factors associated with risky sexual behavior among individuals in residential substance abuse treatment. Psychology of Addictive Behaviors 2010, 24(2):311-321.

1011. Rich JD, Holmes L, Salas C, Macalino G, Davis D, Ryczek J, Flanigan T: Successful linkage of medical care and community services for HIV-positive offenders being released from prison. Journal of Urban Health 2001, 78(2):279-289.

1012. Rich KM, Bia J, Altice FL, Feinberg J: Integrated Models of Care for Individuals with Opioid Use Disorder: How Do We Prevent HIV and HCV? Current HIV/AIDS Reports 2018, 15(3):266-275.

1013. Rich KM, Valencia Huamani J, Kiani SN, Cabello R, Elish P, Florez Arce J, Pizzicato LN, Soria J, Wickersham JA, Sanchez J et al: Correlates of viral suppression among HIV-infected men who have sex with men and transgender women in Lima, Peru. AIDS Care - Psychological and Socio-Medical Aspects of AIDS/HIV 2018:1-10.

1014. Ricks PM, Hershow RC, Rahimian A, Huo D, Johnson W, Prachand N, Jimenez A, Wiebel W, Paul W: A randomized trial comparing standard outcomes in two treatment models for substance users with tuberculosis. Int J Tuberc Lung Dis 2015, 19(3):326-332.

1015. Ricks PM, Hershow RC, Rahimian A, Huo D, Johnson W, Prachand N, Jimenez A, Wiebel W, Paul W: A randomized trial comparing standard outcomes in two treatment models for substance users with tuberculosis. Int J Tuberc Lung Dis 2015, 19(3):326-332.

1016. Rieckmann T, Muench J, McBurnie MA, Leo MC, Crawford P, Ford D, Stubbs J, O'Cleirigh C, Mayer KH, Fiscella K et al: Medication-assisted treatment for substance use disorders within a national community health center research network. Substance abuse 2016, 37(4):625-634.

1017. Ries R, Krupski A, West, II, Maynard C, Bumgardner K, Donovan D, Dunn C, Roy-Byrne P: Correlates of Opioid Use in Adults With Self-Reported Drug Use Recruited From Public Safety-Net Primary Care Clinics. Journal of Addiction Medicine 2015, 9(5):417-426.

1018. Ries R, Krupski A, West II, Maynard C, Bumgardner K, Donovan D, Dunn C, Roy-Byrne P: Correlates of opioid use in adults with self-reported drug use recruited from public safety-net primary care clinics. Journal of Addiction Medicine 2015, 9(5):417-426.

1019. Rifai MA: Hepatitis C treatment of patients with bipolar disorder: A case series. Primary Care Companion to the Journal of Clinical Psychiatry 2006, 8(6):361-366.

1020. Robaeys G: Management of substance use induced hepatitis C viral infection. An Update. Suchtmedizin in Forschung und Praxis 2009, 11(5):219-223.

1021. Robaeys G: The Management of Chronic Hepatitis C in Patients Infected after Substance Abuse - Practical Considerations. European Gastroenterology and Hepatology Review 2011, 7(3):204-208.

1022. Robaeys G: The management of chronic hepatitis C in patients infected after substance abuse - practical considerations. European Gastroenterology and Hepatology Review 2012, 8(2):116-120.

1023. Rocha M, Pereira S, Ferreira L, Barros H: The role of adherence in tuberculosis HIV-positive patients treated in ambulatory regimen. European Respiratory Journal 2003, 21(5):785-788.

1024. Rodilla Calvelo F, Orovitg Moreno F, Escriva Moscardo S, Ferriols Lisart F, Magraner Gil J: At-home hospitalization of patients with HIV+. Repercussions on a pharmacy service. [Spanish]. Farmacia Hospitalaria 1993, 17(5):257-261.

1025. Rodriguez CV, Rubenstein K, Hu H, Horberg MA: Times they are a changing: HCV treatment trends in a large integrated health system. Hepatology 2015, 62:1122A-1123A.

1026. Rodriguez CV, Rubenstein K, Hu H, Linas BP, Horberg MA: Increasing hepatitis C Virus (HCV) screening and linkage to care in a large integrated health system. Hepatology 2015, 62:1091A-1092A.

1027. Rogers SJ, Tross S, Doino-Ingersol J, Weisfuse I: Partner notification with HIV-infected drug users: results of formative research. AIDS Care 1998, 10(4):415-429.

1028. Rojas-Feria M, Castro M, Suarez E, Ampuero J, Romero-Gomez M: Hepatobiliary manifestations in inflammatory bowel disease: the gut, the drugs and the liver. World Journal of Gastroenterology 2013, 19(42):7327-7340.

1029. Rolf J, Nanda J, Baldwin J, Chandra A, Thompson L: Substance misuse and HIV/AIDS risks among delinquents: a prevention challenge. Int J Addict 1990, 25(4A):533-559.

1030. Romac D, Gracin B, Cavar Z, Orban M: Travelling through recovery. European Psychiatry 2018, 48 (Supplement 1):S450.

1031. Roncero C, Barral C, Grau-Lopez L, Bachiller D, Szerman N, Casas M, Ruiz P: Protocols of dual diagnosis intervention in schizophrenia. Addictive Disorders and their Treatment 2011, 10(3):131-154.

1032. Rongey C, Asch S, Knight SJ: Access to care for vulnerable veterans with hepatitis C: A hybrid conceptual framework and a case study to guide translation. Translational Behavioral Medicine 2011, 1(4):644-651.

1033. Roose RJ, Cockerham-Colas L, Soloway I, Batchelder A, Litwin AH: Reducing barriers to hepatitis C treatment among drug users: an integrated hepatitis C peer education and support program. Journal of Health Care for the Poor & Underserved 2014, 25(2):652-662.

1034. Rosedale MT, Strauss SM: How persons with chronic hepatitis C in residential substance abuse treatment programs think about depression and interferon therapy. J Am Psychiatr Nurses Assoc 2010, 16(6):350-356.

1035. Rosenberg SD, Goldberg RW, Dixon LB, Wolford GL, Slade EP, Himelhoch S, Gallucci G, Potts W, Tapscott S, Welsh CJ: Assessing the STIRR model of best practices for blood-borne infections of clients with severe mental illness. Psychiatric Services 2010, 61(9):885-891.

1036. Rosendahl C, Neumann-Mager J, Schroll E, Fahrenheim E, Autenrieth R, Wintergerst U, Belohradsky BH: [Psychosocial problems in HIV infected families--possibilities for coping]. Monatsschr Kinderheilkd 1990, 138(12):789-790.

1037. Rothe TB, Karrer W: Short-course therapy of pulmonary tuberculosis: doctor's compliance. Tuber Lung Dis 1996, 77(1):93-97.

1038. Rudoi NM, Starikov AI, Zhumabaev SN, Modelevskii BS: Improving the diagnosis and treatment of pulmonary tuberculosis among the inmates of reformatory institutions of the Ministry of Home Affairs. [Russian]. Problemy tuberkuleza 1990(7):14-17.

1039. Rudoi NM, Starikov AI, Zhumabaev SN, Modelevskii BS: [Improving the diagnosis and treatment of pulmonary tuberculosis among the inmates of reformatory institutions of the Ministry of Home Affairs]. Problemy Tuberkuleza 1990(7):14-17.

1040. Ruiz M, Cefalu C: Palliative care program for human immunodeficiency virus-infected patients: rebuilding of an academic urban program. Am J Hosp Palliat Care 2011, 28(1):16-21.

1041. Ruiz P: Issues in the psychiatric care of Hispanics. Psychiatric Services 1997, 48(4):539-540.

1042. Russell M, Pauly MP, Moore CD, Chia C, Dorrell JM, Cunanan RJ, Witt G: The impact of lifetime drug use on hepatitis C treatment outcomes in insured members of an integrated health care plan. Drug and Alcohol Dependence 2014, 134(1):222-227.

1043. Ruter O, Haardt B, Demelius S: Experiences with levomethadone substitution in AIDS patients with intravenous drug dependence. [German]. Das Offentliche Gesundheitswesen 1991, 53(8-9):447-450.

1044. Ruter O, Haardt B, Demelius S: [Experiences with levomethadone substitution in AIDS patients with intravenous drug dependence]. Offentl Gesundheitswes 1991, 53(8-9):447-450.

1045. Rytik PG, Kutcherov, II, Muller WE, Poleschuk NN, Duboiskaya GP, Kruzo M, Podolskaya IA: Small animal model of HIV-1 infection. J Clin Virol 2004, 31 Suppl 1:S83-87.

1046. Saberi P, Ranatunga DK, Quesenberry CP, Silverberg MJ: Clinical implications of the nelfinavir-proton pump inhibitor drug interaction in patients with human immunodeficiency virus. Pharmacotherapy:The Journal of Human Pharmacology & Drug Therapy 2011, 31(3):253-261.

1047. Saberi P, Ranatunga DK, Quesenberry Jr CP, Silverberg MJ: Clinical implications of the nelfinavir-proton pump inhibitor drug interaction in patients with human immunodeficiency virus. Pharmacotherapy 2011, 31(3):253-261.

1048. Safo SA, Blank AE, Cunningham CO, Quinlivan EB, Lincoln T, Blackstock OJ: Pain is Associated with Missed Clinic Visits Among HIV-Positive Women. AIDS and behavior 2017, 21(6):1782-1790.

1049. Sajid A, Whiteman A, Bell RL, Greene MS, Engleman EA, Chambers RA: Prescription drug monitoring program data tracking of opioid addiction treatment outcomes in integrated dual diagnosis care involving injectable naltrexone. Am J Addict 2016, 25(7):557-564.

1050. Saldana L, Chamberlain P, Sheidow A: Integrated treatment for mothers involved in child welfare for substance abuse. Drug and Alcohol Dependence 2015, 146:e87.

1051. Salloum IM, Brown ES: Management of comorbid bipolar disorder and substance use disorders. American Journal of Drug and Alcohol Abuse 2017, 43(4):366-376.

1052. Salomon N, Perlman DC, Rubenstein A, Mandelman D, McKinley FW, Yancovitz SR: Implementation of universal directly observed therapy at a New York City hospital and evaluation of an out-patient directly observed therapy program. The international journal of tuberculosis and lung disease : the official journal of the International Union against Tuberculosis and Lung Disease 1997, 1(5):397-404.

1053. Sambamoorthi U, Collins SR, Crystal S, Walkup J: Home-care use and expenditures among Medicaid beneficiaries with AIDS. Health Care Financing Review 1999, 20(4):161-177.

1054. Samet JH, Larson MJ, Horton NJ, Doyle K, Winter M, Saitz R: Linking alcohol- and drug-dependent adults to primary medical care: a randomized controlled trial of a multi-disciplinary health intervention in a detoxification unit. Addiction 2003, 98(4):509-516.

1055. Samuels A, Reinert S, Friedmann P, Trinh T, Frank J: Health care utilization of the recently released incarcerated population in rhode island - A critical need for linkage to primary care. Journal of General Internal Medicine 2010, 25:S291.

1056. Sanchez GV, Llibre JM, Torrens M, Sanvisens A, Mateu G, Knobel H, Langohr K, Santos JR, Muga R: Effectiveness of antiretroviral therapy in HIV-1-infected active drug users attended in a drug abuse outpatient treatment facility providing a multidisciplinary care strategy. Current HIV Research 2012, 10(4):356-363.

1057. Sangsari S, Milloy MJ, Ibrahim A, Kerr T, Zhang R, Montaner J, Wood E: Physician experience and rates of plasma HIV-1 RNA suppression among illicit drug users: an observational study. BMC Infect Dis 2012, 12:22.

1058. Sansovich D: HIV and substance abuse: the biggest challenge for many clinicians. HIV Clin 2002, 14(4):7.

1059. Saphonn V, Fujita M, Samreth S, Chan S, Rouet F, Khol V, Mam S, Mom C, Tuot S, Le LV et al: Cluster of HIV Infections Associated With Unsafe Injection Practices in a Rural Village in Cambodia. J Acquir Immune Defic Syndr 2017, 75(3):e82-e86.

1060. Satre DD, Altschuler A, Parthasarathy S, Silverberg MJ, Volberding P, Campbell CI: Implementation and operational research: Affordable care act implementation in a California health care system leads to growth in HIV-positive patient enrollment and changes in patient characteristics. Journal of Acquired Immune Deficiency Syndromes 2016, 73(5):e76-e82.

1061. Satre DD, DeLorenze GN, Quesenberry CP, Tsai A, Weisner C: Factors associated with treatment initiation for psychiatric and substance use disorders among persons with HIV. Psychiatric Services 2013, 64(8):745-753.

1062. Satre DD, DeLorenze GN, Quesenberry Jr CP, Tsai A, Weisner C: Factors associated with treatment initiation for psychiatric and substance use disorders among persons with HIV. Psychiatric Services 2013, 64(8):745-753.

1063. Satre DD, Leibowitz A, Hare CB, Horberg M, Lu Y, Weisner C, Silverberg M: Alcohol use among HIV-positive patients in primary care: Psychiatric symptoms and other substance use associated with hazardous drinking. Alcoholism: Clinical and Experimental Research 2016, 1):72A.

1064. Satre DD, Leibowitz A, Silverberg MJ: Behavioral interventions to reduce hazardous drinking among primary care HIV patients: Methods and preliminary results. Alcoholism: Clinical and Experimental Research 2015, 39:21A.

1065. Saunderson PR: An economic evaluation of alternative programme designs for tuberculosis control in rural Uganda. Social Science and Medicine 1995, 40(9):1203-1212.

1066. Saxinger L, Taylor G: Two cases of significant adverse patient events due to contact isolation precautions in ambulatory care programs - Time to reassess evidence and policies. Canadian Journal of Infectious Diseases and Medical Microbiology 2011, 22:31A.

1067. Sayer C, Fisher M, Nixon E, Nambiar K, Richardson D, Perry N, Llewellyn C: Will I? Won't I? Why do men who have sex with men present for post-exposure prophylaxis for sexual exposures? Sexually Transmitted Infections 2009, 85(3):206-211.

1068. Schackman BR, Gutkind S, Morgan JR, Leff JA, Behrends CN, Delucchi KL, McKnight C, Perlman DC, Masson CL, Linas BP: Cost-effectiveness of hepatitis C screening and treatment linkage intervention in US methadone maintenance treatment programs. Drug and Alcohol Dependence 2018, 185:411-420.

1069. Schackman BR, Leff JA, Botsko M, Fiellin DA, Altice FL, Korthuis PT, Sohler N, Weiss L, Egan JE, Netherland J et al: The cost of integrated HIV care and buprenorphine/naloxone treatment: Results of a cross-site evaluation. Journal of Acquired Immune Deficiency Syndromes 2011, 56(SUPPL. 1):S76-S82.

1070. Schackman BR, Leff JA, Botsko M, Fiellin DA, Altice FL, Korthuis PT, Sohler N, Weiss L, Egan JE, Netherland J et al: The cost of integrated HIV care and buprenorphine/naloxone treatment: results of a cross-site evaluation. J Acquir Immune Defic Syndr 2011, 56 Suppl 1:S76-82.

1071. Schackman BR, Merrill JO, McCarty D, Levi J, Lubinski C: Overcoming policy and financing barriers to integrated buprenorphine and HIV primary care. Clinical Infectious Diseases 2006, 43(SUPPL. 4):S247-S253.

1072. Schaefer M, Heinz A, Backmund M: Treatment of chronic hepatitis C in patients with drug dependence: time to change the rules? Addiction 2004, 99(9):1167-1175.

1073. Schaefer M, Heinz A, Backmund M: Treatment of chronic hepatitis C in patients with drug dependence: time to change the rules? Addiction 2004, 99(9):1167-1175.

1074. Schafer A, Eck M, Bell U, Heckmann W, Schwartlander B: [Use of methadone in obstetric and gynecologic management of drug-dependent females with and without HIV infection]. Geburtshilfe Frauenheilkd 1991, 51(8):595-601.

1075. Schenker S, Montalvo R: Alcohol and the pancreas. Recent Dev Alcohol 1998, 14:41-65.

1076. Scherz N, Brunner N, Bruggmann P: Direct-acting antivirals for hepatitis C in patient in opioid substitution treatment and heroin assisted treatment: Real-life data. Journal of Hepatology 2017, 66 (1 Supplement 1):S726.

1077. Schietinger H: Housing for people with AIDS. Death Studies 1988, 12(5-6):481-499.

1078. Schlaff AL: Boston's Codman Square Community Partnership for Health Promotion. Public Health Reports 1991, 106(2):186-191.

1079. Schleifer SJ, Keller SE, LaFarge S, Dhaibar Y, Shiflett SC, Eckholdt HM: Human immunodeficiency virus-type 1 infection in an inner-city alcohol treatment program. Alcohol Clin Exp Res 1996, 20(1):75-80.

1080. Schluger NW, El-Bassel N, Hermosilla S, Terlikbayeva A, Darisheva M, Aifah A, Galea S: Tuberculosis, drug use and HIV infection in Central Asia: An urgent need for attention. Drug and Alcohol Dependence 2013, 132(SUPPL1):S32-S36.

1081. Schwappach DL, Bruggmann P: An integrated model of care to counter high incidence of HIV and sexually transmitted diseases in men who have sex with men - initial analysis of service utilizers in Zurich. BMC Public Health 2008, 8:180.

1082. Schwappach DLB, Bruggmann P: An integrated model of care to counter high incidence of HIV and sexually transmitted diseases in men who have sex with men - Initial analysis of service utilizers in Zurich. BMC Public Health 2008, 8 (no pagination)(180).

1083. Scott K, Ngayen TQ, Whetten K: If there's a will, is there a way? Integrating HIV and mental health services in rural areas. AIDS and Public Policy Journal 2002, 17(4):127-134.

1084. Selwyn PA: The impact of HIV infection on medical services in drug abuse treatment programs. Journal of Substance Abuse Treatment 1996, 13(5):397-410; discussion 439.

1085. Selwyn PA, Forstein M: Overcoming the False Dichotomy of Curative vs Palliative Care for Late-Stage HIV/AIDS: "Let Me Live the Way I Want to Live, until I Can't". Journal of the American Medical Association 2003, 290(6):806-814.

1086. Senak M: Minorities dissatisfied with outpatient HIV care. Journal of the International Association of Physicians in AIDS Care 1996, 2(1):42.

1087. Senay EC, Barthwell A, Marks R, Bokos PJ: Medical maintenance: an interim report. Journal of Addictive Diseases 1994, 13(3):65-69.

1088. Sendagire I, Cobelens F, Kambugu A, Konde-Lule J, van der Loeff MS: Frequency and predictors for late start of antiretroviral therapy in primary care clinics, Kampala, Uganda. Journal of Acquired Immune Deficiency Syndromes 2012, 19.

1089. Serre A, Schutz-Samson M, Cabral C, Martin F, Hardy R, DeAquino O, Vinsonneau P, Arnaudies M, Fierro F, Mathieu L et al: [Living conditions of prostitutes: consequences for the prevention of HIV infection]. Rev Epidemiol Sante Publique 1996, 44(5):407-416.

1090. Setzer JR, Scott AA, Balli J, Rodriguez-Aragon G, Mangos JA, Flynn C, Castillo JE, Sherman JO: An integrated model for medical care, substance abuse treatment and AIDS prevention services to minority youth in a short-term detention facility. Journal of Prison and Jail Health 1991, 10(2):91-115.

1091. Shah ND, Hoffman JM, Vermeulen LC, Hunkler RJ, Hontz KM: Projecting future drug expenditures - 2003. American Journal of Health-System Pharmacy 2003, 60(2):137-149.

1092. Shannon K, Kerr T, Milloy MJ, Anema A, Zhang R, Montaner JS, Wood E: Severe food insecurity is associated with elevated unprotected sex among HIV-seropositive injection drug users independent of HAART use. AIDS 2011, 25(16):2037-2042.

1093. Shannon K, Kerr T, Milloy MJ, Anema A, Zhang R, Montaner JSG, Wood E: Severe food insecurity is associated with elevated unprotected sex among HIV-seropositive injection drug users independent of HAART use. Aids 2011, 25(16):2037-2042.

1094. Shaw SY, McClarty LM, Kasper K, Bergen C, Ireland L, Carnochan T, Wylie J, Yu N, Keynan Y, Caetano P et al: Factors associated with length of time to care program entry from initial HIV diagnosis in Manitoba. Canadian Journal of Infectious Diseases and Medical Microbiology 2014, 25:79A.

1095. Shidhaye R, Lund C, Chisholm D: Closing the treatment gap for mental, neurological and substance use disorders by strengthening existing health care platforms: Strategies for delivery and integration of evidence-based interventions. International Journal of Mental Health Systems 2015, 9 (1) (no pagination)(40).

1096. Shin S, Livchits V, Connery HS, Shields A, Yanov S, Yanova G, Fitzmaurice GM, Nelson AK, Greenfield SF: Effectiveness of alcohol treatment interventions integrated into routine tuberculosis care in Tomsk, Russia. Addiction 2013, 108(8):1387-1396.

1097. Shin S, Livchits V, Connery HS, Shields A, Yanov S, Yanova G, Fitzmaurice GM, Nelson AK, Greenfield SF: Effectiveness of alcohol treatment interventions integrated into routine tuberculosis care in Tomsk, Russia. Addiction 2013, 108(8):1387-1396.

1098. Shiraishi A, Hiraoka A, Sogabe I, Furuya K, Tatsukawa H, Imai Y, Yamago H, Shimizu Y, Tanihira T, Miyata H et al: Real-time tissue elastography: Non-invasive evaluation for liver fibrosis in chronic liver disease due to HCV. Gastroenterology 2013, 1):S449.

1099. Shuster J: ISMP Adverse Drug Reactions - Agranulocytosis Induced by Proton Pump Inhibitors; Two Cases of Fatal Antimalarial-Induced Cardiomyopathy; Oxaliplatin-Induced Thrombotic Thrombocytopenic Purpura; Unique Skin Eruption Caused by Telaprevir; Acute-Onset Opioid-Induced Hyperalgesia in a Child; Risk of Sharing a Pill Cutter; Dementia and Risk of Adverse Warfarin-Related Events in Nursing Homes. Hospital Pharmacy 2013, 48(1):10-13.

1100. Sibthorpe B, Fleming D, Tesselaar H, Gould J, Nichols L: The response of injection drug users to free treatment on demand: implications for HIV control. Am J Drug Alcohol Abuse 1996, 22(2):203-213.

1101. Siccardi L, Vautel-Pons D, dos Santos MT, Camus N, Louchart de la Chapelle S: [Secondary benefits of cognitive rehabilitation for a chronic ethylic patient: effects on disorder consciousness, motivation, and global therapeutic cooperation]. Encephale 2014, 40(3):263-270.

1102. Silberstein CH, O'Dowd MA, Chartock P, Schoenbaum EE, Friedland G, Hartel D, McKegney FP: A prospective four-year follow-up of neuropsychological function in HIV seropositive and seronegative methadone-maintained patients. General Hospital Psychiatry 1993, 15(6):351-359.

1103. Silva MJ, Pereira C, Loureiro R, Balsa C, Lopes P, Agua-Doce I, Belo E, Martins HC, Coutinho R, Padua E: Hepatitis C in a Mobile Low-Threshold Methadone Program. Eur J Gastroenterol Hepatol 2017, 29(6):657-662.

1104. Simeone C, Shapiro B, Lum PJ: Integrated HIV care is associated with improved engagement in treatment in an urban methadone clinic. Addiction science & clinical practice 2017, 12(1):19.

1105. Simoni J, Nance R, Delaney JA, Wilson IB, Aunon F, Safren SA, Mugavero MJ, Mayer KH, Kitahata MM, Crane HM: Changes in viral load across us clinics over time. Topics in Antiviral Medicine 2016, 24 (E-1):447.

1106. Simoni JM, Pantalone DW, Plummer MD, Huang B: A randomized controlled trial of a peer support intervention targeting antiretroviral medication adherence and depressive symptomatology in HIV-positive men and women. Health Psychology 2007, 26(4):488-495.

1107. Simonova M, Vasilev G, Mateva L, Kantchelov, Katzarov K: Consensus for the diagnosis, treatment and monitoring of patients with chronic viral hepatitis, who are diagnosed with opioid dependence and undergo treatment with opioid agonists and agonists-antagonists. Suchtmedizin in Forschung und Praxis 2013, 15 (4):244-245.

1108. Singal AG, Tiro J, Li X, Adams-Huet B, Chubak J: Hepatocellular Carcinoma Surveillance among Patients with Cirrhosis in a Population-based Integrated Health Care Delivery System. Journal of Clinical Gastroenterology 2017, 51(7):650-655.

1109. Singer M: Why do Puerto Rican injection drug users inject so often? Anthropology and Medicine 1999, 6(1):31-58.

1110. Slade EP, Rosenberg S, Dixon LB, Goldberg RW, Wolford GL, Himelhoch S, Tapscott S: Costs of a public health model to increase receipt of hepatitis-related services for persons with mental illness. Psychiatric Services 2013, 64(2):127-133.

1111. Smirnoff M, Goldberg R, Indyk L, Adler JJ: Directly observed therapy in an inner city hospital. Int J Tuberc Lung Dis 1998, 2(2):134-139.

1112. Smith-Rohrberg D, Mezger J, Walton M, Bruce RD, Altice FL: Impact of enhanced services on virologic outcomes in a directly administered antiretroviral therapy trial for HIV-infected drug users. J Acquir Immune Defic Syndr 2006, 43 Suppl 1:S48-53.

1113. Smith-Rohrberg D, Mezger J, Walton M, Bruce RD, Altice FL: Impact of enhanced services on virologic outcomes in a directly administered antiretroviral therapy trial for HIV-infected drug users. J Acquir Immune Defic Syndr 2006, 43 Suppl 1:S48-53.

1114. Smyrnov P, Broadhead RS, Datsenko O, Matiyash O: Rejuvenating harm reduction projects for injection drug users: Ukraine's nationwide introduction of peer-driven interventions. International Journal of Drug Policy 2012, 23(2):141-147.

1115. Snyder CM, Kaempfer SH, Ries K: An interdisciplinary, interagency, primary care approach to case management of the dually diagnosed patient with HIV disease. Journal of the Association of Nurses in AIDS Care 1996, 7(5):72-82.

1116. Socias ME, Ti L, Dong H, Shoveller J, Kerr T, Montaner J, Milloy MJ: High prevalence of willingness to use direct-acting antiviral-based regimens for hepatitis C virus (HCV) infection among HIV/HCV coinfected people who use drugs. HIV Medicine 2017, 18(9):647-654.

1117. Sohler NL, Wong MD, Cunningham WE, Cabral H, Drainoni ML, Cunningham CO: Type and pattern of illicit drug use and access to health care services for HIV-infected people. AIDS Patient Care and STDs 2007, 21(SUPPL. 1):S-68-S-76.

1118. Solomon R: Some concerns in providing home care for i.v. drug users. Caring : National Association for Home Care magazine 1991, 10(8):42-43.

1119. Solomon SS, Lucas GM, Celentano DD, McFall AM, Ogburn E, Moulton LH, Srikrishnan AK, Kumar MS, Anand S, Solomon S et al: Design of the Indian NCA study (Indian national collaboration on AIDS): a cluster randomized trial to evaluate the effectiveness of integrated care centers to improve HIV outcomes among men who have sex with men and persons who inject drugs in India. BMC health services research 2016, 16(1):652.

1120. Solomon SS, Solomon S, McFall AM, Srikrishnan AK, Anand S, Balakrishnan P, Ogburn E, Moulton L, Kumar MS, Celentano DD et al: Impact of service integration on HIV testing uptake among key populations in India. Topics in Antiviral Medicine 2018, 26 (Supplement 1):508s.

1121. Somlai AM, Kelly JA, Otto-Salaj L, Nelson D: 'Lifepoint': A case study in using social science community identification data to guide the implementation of a needle exchange program. AIDS Education and Prevention 1999, 11(3):187-202.

1122. Song JH, Jung KS, Kang MW, Kim DJ, Pai H, Suh GY, Shim TS, Ahn JH, Ahn CM, Woo JH et al: Treatment guidelines for community-acquired pneumonia in Korea: An evidence-based approach to appropriate antimicrobial therapy. [Korean]. Tuberculosis and Respiratory Diseases 2009, 67(4):281-302.

1123. Sorensen JL, Costantini MF, Wall TL, Gibson DR: Coupons attract high-risk untreated heroin users into detoxification. Drug Alcohol Depend 1993, 31(3):247-252.

1124. Sorensen JL, Haug NA, Larios S, Gruber VA, Tulsky J, Powelson E, Logan DP, Shapiro B: Directly administered antiretroviral therapy: pilot study of a structural intervention in methadone maintenance. Journal of Substance Abuse Treatment 2012, 43(4):418-423.

1125. Sorensen JL, London J, Heitzmann C, Gibson DR, Morales ES, Dumontet R, Acree M: Psychoeducational group approach: HIV risk reduction in drug users. AIDS Educ Prev 1994, 6(2):95-112.

1126. Sorensen JL, Miller MS: Impact of HIV risk and infection on delivery of psychosocial treatment services in outpatient programs. Journal of Substance Abuse Treatment 1996, 13(5):387-395; discussion 439.

1127. Sosin I, Burmaka N, Chuev Y, Dvoryak S: Integrative narcology: New strategy for the improvement of therapeutic programs. European Psychiatry Conference: 21st European Congress of Psychiatry, EPA 2013, 28(SUPPL. 1).

1128. Soto TA, Bell J, Pillen MB: Literature on integrated HIV care: A review. AIDS Care - Psychological and Socio-Medical Aspects of AIDS/HIV 2004, 16(SUPPL. 1):S43-S55.

1129. Soto TA, Bell J, Pillen MB, Hiv/Aids Treatment Adherence HO, Cost Study G: Literature on integrated HIV care: a review. AIDS Care 2004, 16 Suppl 1:S43-55.

1130. Southern WN, Drainoni ML, Smith BD, Christiansen CL, McKee D, Gifford AL, Weinbaum CM, Thompson D, Koppelman E, Maher S et al: Hepatitis C testing practices and prevalence in a high-risk urban ambulatory care setting. Journal of Viral Hepatitis 2011, 18(7):474-481.

1131. Souza PN, Miranda EJ, Cruz R, Forte DN: Palliative care for patients with HIV/AIDS admitted to intensive care units. Rev 2016, 28(3):301-309.

1132. Spencer M: Pain relief in Thailand. J Pain Pall Care Pharmacother 2003, 17(3-4):53-61; discussion 63-54.

1133. Spire B, Lucas GM, Carrieri MP: Adherence to HIV treatment among IDUs and the role of opioid substitution treatment (OST). International Journal of Drug Policy 2007, 18(4):262-270.

1134. Spizzichino L, Casella P, Zaccarelli M, Rezza G, Venezia S, Gattari P: HIV infection among foreign people involved in HIV-related risk activities and attending an HIV reference centre in Rome: the possible role of counselling in reducing risk behaviour. AIDS Care 1998, 10(4):473-480.

1135. Spradling PR, Richardson JT, Buchacz K, Moorman AC, Finelli L, Bell BP, Brooks JT, Investigators HIVOS: Trends in hepatitis C virus infection among patients in the HIV Outpatient Study, 1996-2007. J Acquir Immune Defic Syndr 2010, 53(3):388-396.

1136. Sprah L, Dernovsek MZ, Wahlbeck K, Haaramo P: Psychiatric readmissions and their association with physical comorbidity: A systematic literature review. BMC Psychiatry 2017, 17 (1) (no pagination)(2).

1137. Stahmeyer JT, Rossol S, Bert F, Abdelfattah AM, Mauss S, Heyne R, John C, Pape S, Schober A, Teuber G et al: Epidemiology, treatment outcomes and costs of treating hepatitisc in routine care - Results from a large multicenter trial. Value in Health 2013, 16 (7):A341-A342.

1138. Stahmeyer JT, Rossol S, Bert F, Abdelfattah M, Wiebner B, Wedemeyer H, Krauth C: Cost of illness of hepatitis c in germany: A retrospective multicenter analysis. Value in Health 2013, 16 (3):A86.

1139. Stahmeyer JT, Rossol S, Bert F, Antoni C, Demir M, Hinrichsen H, Huppe D, Teuber G, Wiebner B, Wedemeyer H et al: Cost of treating hepatitis C in Germany: A retrospective multicenter analysis. European Journal of Gastroenterology and Hepatology 2014, 26(11):1278-1285.

1140. Steffen T, Kaufmann B, Blattler R, Dobler-Mikola A, Gutzwiller F, Uchtenhagen A: [Heroin-assisted treatment of opiate addicts--former and current research emphasis]. Gesundheitswesen 1999, 61(8-9):407-412.

1141. Stein E, Cruz-Lemini M, Altamirano J, Ndugga N, Couper D, Abraldes JG, Bataller R: Heavy daily alcohol intake at the population level predicts the weight of alcohol in cirrhosis burden worldwide. Journal of Hepatology 2016, 65(5):998-1005.

1142. Stein GL, Fleischman AR: Addiction treatment: promoting a medical approach to substance use. Journal of Urban Health 1998, 75(3):558-583.

1143. Stein JA, Andersen RM, Robertson M, Gelberg L: Impact of hepatitis B and C infection on health services utilization in homeless adults: A test of the Gelberg-Andersen behavioral model for vulnerable populations. Health Psychology 2012, 31(1):20-30.

1144. Stein MD, O'Sullivan PS, Ellis P, Perrin H, Wartenberg A: Utilization of medical services by drug abusers in detoxification. Journal of substance abuse 1993, 5(2):187-193.

1145. Stein MD, Urdaneta ME, Clarke J, Maksad J, Sobota M, Hanna L, Markson LE: Use of antiretroviral therapies by HIV-infected persons receiving methadone maintenance. Journal of Addictive Diseases 2000, 19(1):85-94.

1146. Stephens DB, Young AM, Havens JR: Healthcare contact and treatment uptake following hepatitis C virus screening and counseling among rural Appalachian people who use drugs. International Journal of Drug Policy 2017, 47:86-94.

1147. Sterling RC, Gottheil E, Weinstein SP, Serota R: The effect of therapist/patient race- and sex-matching in individual treatment. Addiction 2001, 96(7):1015-1022.

1148. Stevens SJ, Murphy BS, McKnight K: Traumatic stress and gender differences in relationship to substance abuse, mental health, physical health, and HIV risk behavior in a sample of adolescents enrolled in drug treatment. Child maltreatment 2003, 8(1):46-57.

1149. Stiel S, Joppich R, Korb K, Hahnen M, Elsner F, Rossaint R, Radbruch L: [Problems and deficits in the transition from inpatient and outpatient care of cancer patients. A qualitative analysis]. Schmerz 2009, 23(5):510-517.

1150. Stjernsward J: Palliative care: the public health strategy. J Public Health Policy 2007, 28(1):42-55.

1151. Stojanovic D, Schelfhout J, Houtchens A, Crane HM, Brown E, Kim N, Kitahata MM, Kauf TL, Delaney JAC: Changes in platelet counts over time in HIV and hepatitis c virus (HCV) co-infected patients. Pharmacoepidemiology and Drug Safety 2012, 21:109.

1152. Stone KA: Reviewing harm reduction for people who inject drugs in Asia: The necessity for growth. Harm Reduction Journal 2015, 12 (1) (no pagination)(32).

1153. Stone VE, Weissman JS, Cleary PD: Satisfaction with ambulatory care of persons with AIDS: Predictors of patient ratings of quality. Journal of General Internal Medicine 1995, 10(5):239-245.

1154. Stopka TJ, Hutcheson M, Donahue A: Access to healthcare insurance and healthcare services among syringe exchange program clients in Massachusetts: Qualitative findings from health navigators with the iDU ("I do") Care Collaborative. Harm Reduction Journal 2017, 14 (1) (no pagination)(26).

1155. Storholm ED, Silverberg M, Satre D: Racial and ethnic differences in substance use diagnoses, comorbid psychiatric disorders and treatment initiation among HIV-positive and HIV-negative women. Drug and Alcohol Dependence 2017, 171:e198.

1156. Storholm ED, Silverberg MJ, Satre DD: Racial and Ethnic Differences in Substance Use Diagnoses, Comorbid Psychiatric Disorders, and Treatment Initiation among HIV-Positive and HIV-Negative Women in an Integrated Health Plan. Journal of Psychoactive Drugs 2016, 48(5):377-383.

1157. Storholm ED, Volk JE, Marcus JL, Silverberg MJ, Satre DD: Risk Perception, Sexual Behaviors, and PrEP Adherence Among Substance-Using Men Who Have Sex with Men: a Qualitative Study. Prevention science : the official journal of the Society for Prevention Research 2017, 18(6):737-747.

1158. Stowe A, Ross MW, Wodak A, Thomas GV, Larson SA: Significant relationships and social supports of injecting drug users and their implications for HIV/AIDS services. AIDS Care 1993, 5(1):23-33.

1159. Straub H, Adams M, Silver RK: Can an electronic health record system be used for preconception health optimization? Maternal and child health journal 2014, 18(9):2134-2140.

1160. Strauss AL, Fagerhaugh S, Suczek B, Wiener C: AIDS and health care deficiencies. Society 1991, 28(5):63-73.

1161. Strauss D: A community-based organization's integration of HIV and substance abuse treatment services for ex-offenders. Social Work in Health Care 2006, 42(3-4):61-76.

1162. Strauss JL, Marx CE, Weitlauf JC, Stechuchak KM, Straits-Troster K, Worjoloh AW, Sherrod CB, Olsen MK, Butterfield MI, Calhoun PS: Is military sexual trauma associated with trading sex among women veterans seeking outpatient mental health care? J Trauma Dissociation 2011, 12(3):290-304.

1163. Strauss SM, Falkin GP, Vassilev Z, Des Jarlais DC, Astone J: A nationwide survey of hepatitis C services provided by drug treatment programs. Journal of Substance Abuse Treatment 2002, 22(2):55-62.

1164. Stringari-Murray S, Clayton A, Chang J: A model for integrating hepatitis C services into an HIV/AIDS program. Journal of the Association of Nurses in AIDS Care 2003, 14(5 Suppl):95S-107S.

1165. Stuber M, Spence Gress C, Rodger D, Wong A: Clinic to community: Inter-professional collaboration in enhanced adherence to antiretroviral therapy. Canadian Journal of Infectious Diseases and Medical Microbiology 2014, 25:54A-55A.

1166. Stuck AE, Tamai IY: Medication management in the home. Clinics in Geriatric Medicine 1991, 7(4):733-748.

1167. Subramaniam GA, Stitzer MA: Clinical characteristics of treatment-seeking prescription opioid vs. heroin-using adolescents with opioid use disorder. Drug and Alcohol Dependence 2009, 101(1-2):13-19.

1168. Sue K, Simon RE, Sneh G, Chaudhary MJ, Zeidman J, Cohen MJ: Development of a student-faculty collaborative clinic with a special focus on serving post-incarcerated patients. Journal of General Internal Medicine 2013, 28:S434.

1169. Sulistio M, Jackson K: Three weeks from diagnosis to death: The chaotic journey of a long-term methadone maintenance patient with terminal cancer. Journal of Pain and Symptom Management 2013, 46(4):598-602.

1170. Sullivan LE, Bruce RD, Haltiwanger D, Lucas GM, Eldred L, Finkelstein R, Fiellin DA: Initial strategies for integrating buprenorphine into HIV care settings in the United States. Clinical Infectious Diseases 2006, 43(SUPPL. 4):S191-S196.

1171. Sullivan LE, Fiellin DA: Buprenorphine: Its role in preventing HIV transmission and improving the care of HIV-infected patients with opioid dependence. Clinical Infectious Diseases 2005, 41(6):891-896.

1172. Sun Q, Yin J, Yin X, Zou G, Liang M, Zhong J, Walley J, Wei X: Does the integration of TB medical services in the general hospital improve the quality of TB care? Evidence from a case study in China. Journal of Public Health (United Kingdom) 2013, 35(2):322-328.

1173. Sun WL, Ananthan S, Quizon PM, Huang X, Zhan CG, Zhu J: Mutation of tyrosine 470 of human dopamine transporter is critical for tat-mediated allosteric modulation on human dopamine transporter. Journal of Neuroimmune Pharmacology 2015, 1):S103.

1174. Sun WL, Quizon PM, Yuan Y, Strauss M, McCain R, Zhan CG, Zhu J: Effects of human dopamine transporter histidine547, tyrosine88 and lysine92 intermolecular interactions on basal and Tat-inhibited dopamine transport. Journal of Neuroimmune Pharmacology 2017, 12 (1 Supplement 1):S72-S73.

1175. Surah S, O'Connor A, Hunter K, Delamere S, Kennan E, Barry M, Mulcahy F, Walsh C, Lyons F: Health-related quality of life (HRQOL) and clinical outcomes of HIV-infected intravenous drug users post integration of HIV and addiction services. HIV Medicine 2013, 14:14.

1176. Surratt HL, Kurtz SP, Chen M, Mooss A: HIV risk among female sex workers in Miami: The impact of violent victimization and untreated mental illness. AIDS Care - Psychological and Socio-Medical Aspects of AIDS/HIV 2012, 24(5):553-561.

1177. Sutton P, Huskins B: Against silos : A good practices guide on undertaking an integrated approach to HIV and hepatitis C. Canadian Journal of Infectious Diseases and Medical Microbiology 2014, 25:98A-99A.

1178. Svikis DS, Gorenstein S, Paluzzi P, Fingerhood M: Personality characteristics of treatment-seeking HIV+ pregnant drug dependent women. Journal of Addictive Diseases 1998, 17(3):91-111.

1179. Swartzendruber A, Haardorfer R, Sales JM, Windle M, Brown JL: Changes in recent marijuana use during ad-olescence and emerging adulthood among African American females in the South. J Adolesc Health 2018, 62 (2 Supplement 1):S133-S134.

1180. Sylla L, Bruce RD, Kamarulzaman A, Altice FL: Integration and co-location of HIV/AIDS, tuberculosis and drug treatment services. International Journal of Drug Policy 2007, 18(4):306-312.

1181. Sylvestre DL, Loftis JM, Hauser P, Genser S, Cesari H, Borek N, Kresina TF, Seeff L, Francis H: Co-occurring hepatitis C, substance use, and psychiatric illness: Treatment issues and developing integrated models of care. Journal of Urban Health 2004, 81(4):719-734.

1182. Tadros MB, Chauhan K: Lower extremity edema, Could it be cancer? Journal of General Internal Medicine 2011, 26:S548.

1183. Tait J, Stephens BP, Evans M, Cleary S, Dillon JF: People who inject drugs (PWID): Difficult to get into services, easy to cure. Hepatology 2013, 1):1102A-1103A.

1184. Taj R, Keenan E, O'Connor JJ: A review of patients on methadone maintenance. Irish medical journal 1995, 88(6):218-219.

1185. Takano T, Nakamura K, Takeuchi S, Watanabe M: Disease patterns of the homeless in Tokyo. Journal of Urban Health 1999, 76(1):73-84.

1186. Talal A: Interactive case study: Treatment of active drug users. Journal of Viral Hepatitis 2013, 3):9.

1187. Tamblyn R, Perreault R: Prescription drug use and seniors. Canadian Journal on Aging 2000, 19(SUPPL. 1):143-175.

1188. Tang AM, Bhatnagar T, Ramachandran R, Dong K, Skinner S, Kumar MS, Wanke CA: Malnutrition in a population of HIV-positive and HIV-negative drug users living in Chennai, South India. Drug Alcohol Depend 2011, 118(1):73-77.

1189. Tang YL, Hao W: Improving drug addiction treatment in China. Addiction 2007, 102(7):1057-1063.

1190. Tato J, Nicolas I, Saborido T, Lacasta S, Rodriguez N, Tomas X, Moreno Gomez M: Home care intervention program for AIDS and drugdependency. [Spanish]. Adicciones 2000, 12(4):461-466.

1191. Taylor LE: Delivering care to injection drug users coinfected with HIV and hepatitis C virus. Clinical Infectious Diseases 2005, 40 Suppl 5:S355-361.

1192. Taylor LE, Bowman SE, Chapman S, Zaller N, Stein MD, Cioe PA, Maynard MA, McGovern BH: Treatment for hepatitis C virus genotype 1 infection in HIV-infected individuals on methadone maintenance therapy. Drug and Alcohol Dependence 2011, 116(1-3):233-237.

1193. Tetrault JM, Moore BA, Barry DT, O'Connor PG, Schottenfeld R, Fiellin DA, Fiellin LE: Brief versus extended counseling along with buprenorphine/naloxone for HIV-infected opioid dependent patients. J Subst Abuse Treat 2012, 43(4):433-439.

1194. Thakarar K, Weinstein ZM, Walley AY: Optimising health and safety of people who inject drugs during transition from acute to outpatient care: narrative review with clinical checklist. Postgraduate Medical Journal 2016, 92(1088):356-363.

1195. Thomas BE, Shanmugam P, Malaisamy M, Ovung S, Suresh C, Subbaraman R, Adinarayanan S, Nagarajan K: Psycho-Socio-Economic Issues Challenging Multidrug Resistant Tuberculosis Patients: A Systematic Review. PloS one 2016, 11(1):e0147397.

1196. Thorens G, Pavlicova M, Hu MC, Nunes EV, Campbell AN, Tross S: Comparison of 2 Measures of HIV Sexual Risk Behavior in Women in Outpatient Drug Treatment Programs. Journal of Addiction Medicine 2015, 9(6):454-456.

1197. Thurstone C, Riggs PD, Klein C, Mikulich-Gilbertson SK: A one-session human immunodeficiency virus risk-reduction intervention in adolescents with psychiatric and substance use disorders. J Am Acad Child Adolesc Psychiatry 2007, 46(9):1179-1186.

1198. Ti L, Dong H, Kerr T, Turje RB, Parashar S, Min JE, Montaner J, Wood E, Milloy MJ: The effect of engagement in an HIV/AIDS integrated health programme on plasma HIV-1 RNA suppression among HIV-positive people who use illicit drugs: a marginal structural modelling analysis. HIV Medicine 2017, 18(8):580-586.

1199. Ti L, Parent S, Socias ME: Integrated Models of Care for People Living with Hepatitis C Virus and a Substance Use Disorder: Protocol for a Systematic Review. JMIR Res Protoc 2018, 7(5):e122.

1200. Timko C: Physical characteristics of residential psychiatric and substance abuse programs: organizational determinants and patients outcomes. Am J Community Psychol 1996, 24(1):173-192.

1201. Timko C, Dixon K, Moos RH: Treatment for dual diagnosis patients in the psychiatric and substance abuse systems. Mental Health Services Research 2005, 7(4):229-242.

1202. Tims FM, Dennis ML, Hamilton N, B JB, Diamond G, Funk R, Brantley LB: Characteristics and problems of 600 adolescent cannabis abusers in outpatient treatment. Addiction 2002, 97 Suppl 1:46-57.

1203. Tirschwell DL, Chow F, Becker KJ, Marra CM, Zunt JR, Ho E, Kalani R, Huffer A, Nance R, Delaney JAC et al: Stroke cases in the US centers for aids research (CFAR) network of integrated clinical systems (CNICS) cohort. International Journal of Stroke 2015, 10:215.

1204. Tobias C, Drainoni ML, Wood S: Analysis of human resources and services administration-funded services for HIV-positive substance users: A study of Ryan White CARE act-title III, title IV, and special projects of national significance providers. AIDS Patient Care and STDs 2004, 18(10):604-613.

1205. Tobias C, Drainoni ML, Wood S: Analysis of Human Resources and Services Administration-funded services for HIV-positive substance users: a study of Ryan White CARE Act Title III, Title IV, and Special Projects of National Significance providers. AIDS Patient Care STDS 2004, 18(10):604-613.

1206. Tolou-Shams M, Conrad SM, Houck C, Brown LK: Alcohol use and HIV risk among juvenile drug court offenders. Alcoholism: Clinical and Experimental Research 2009, 1):108A.

1207. Tomljenovic I, Pavlovic M, Grozdek D: [Importance and role of a team work with tuberculous alcoholic patients after 1 year of experience at the department for alcoholic patients with lung diseases in the hospital of Klenovnik]. Plucne Bolesti Tuberk 1973, 25(3):304-309.

1208. Tommasello AC, Gillis LM, Lawler JT, Bujak GJ: Characteristics of homeless HIV-positive outreach responders in urban US and their success in primary care treatment. AIDS Care 2006, 18(8):911-917.

1209. Toresen KH, Salte IM, Skrede S, Nilsen RM, Leiva RA: Clinical outcomes in a cohort of anti-hepatitis C virus-positive patients with significant barriers to treatment referred to a Norwegian outpatient clinic. Scand J Gastroenterol 2014, 49(4):465-472.

1210. Torian LV, Wiewel EW: Continuity of HIV-related medical care, New York City, 2005-2009: Do patients who initiate care stay in care? AIDS Patient Care STDS 2011, 25(2):79-88.

1211. Torrens M, Fonseca F, Castillo C, Domingo-Salvany A: Methadone maintenance treatment in Spain: The success of a harm reduction approach. Bulletin of the World Health Organization 2013, 91(2):136-141.

1212. Torrens M, Fonseca F, Castillo C, Salvany AD: Opioid substitution treatment in Spain: 20 years of experience in harm reduction programs. Drug and Alcohol Dependence 2015, 156:e223.

1213. Tovo CV, Dos Santos DE, De Mattos AZ, De Almeida PRL, De Mattos AA, Santos BR: Ambulatorial prevalence of hepatitis B and C markers in patients with human immunodeficiency virus infection in a general hospital. [Portuguese]. Arquivos de Gastroenterologia 2006, 43(2):73-76.

1214. Trabut JB, Barrault C, Charlot H, Carmona D, Bourdel A, Benslimane M, Francois M, Kini-Matondo W, Causse R, Roudot-Thoraval F et al: Integrated Care for the Use of Direct-acting Antivirals in Patients With Chronic Hepatitis C and Substance Use Disorder. Journal of Addiction Medicine 2018, 26:26.

1215. Trager E, Khalili M, Masson CL, Vittinghoff E, Creasman J, Mangurian C: Hepatitis C Screening Rate Among Underserved Adults With Serious Mental Illness Receiving Care in California Community Mental Health Centers. American journal of public health 2016, 106(4):740-742.

1216. Tran BX, Nguyen LH, Nong VM, Nguyen CT, Phan HT, Latkin CA: Behavioral and quality-of-life outcomes in different service models for methadone maintenance treatment in Vietnam. Harm Reduction Journal 2016, 13:4.

1217. Tran BX, Nguyen LH, Nong VM, Nguyen CT, Phan HTT, Latkin CA: Behavioral and quality-of-life outcomes in different service models for methadone maintenance treatment in Vietnam. Harm Reduction Journal 2016, 13 (1) (no pagination)(4).

1218. Tran BX, Nguyen LH, Phan HT, Latkin CA: Patient Satisfaction with Methadone Maintenance Treatment in Vietnam: A Comparison of Different Integrative-Service Delivery Models. PLoS ONE [Electronic Resource] 2015, 10(11):e0142644.

1219. Tran BX, Nguyen LH, Phan HT, Nguyen LK, Latkin CA: Preference of methadone maintenance patients for the integrative and decentralized service delivery models in Vietnam. Harm Reduction Journal 2015, 12:29.

1220. Tran BX, Nguyen LH, Phan HTT, Nguyen LK, Latkin CA: Preference of methadone maintenance patients for the integrative and decentralized service delivery models in Vietnam. Harm Reduction Journal 2015, 12 (1) (no pagination)(29).

1221. Tran BX, Nguyen QL, Nguyen LH, Phan HTT, Le HT, Tran TD, Vu TTM, Latkin CA: Expanding co-payment for methadone maintenance services in Vietnam: the importance of addressing health and socioeconomic inequalities. BMC health services research 2017, 17(1):480.

1222. Tran OC, Bruce RD, Masao F, Ubuguyu O, Sabuni N, Mbwambo J, Lambdin BH: Implementation and operational research: Linkage to care among methadone clients living with HIV in Dar es Salaam, Tanzania. Journal of Acquired Immune Deficiency Syndromes 2015, 69(2):e43-e48.

1223. Treloar C, Gray R, Brener L: A piece of the jigsaw of primary care: health professional perceptions of an integrated care model of hepatitis C management in the community. Journal of primary health care 2014, 6(2):129-134.

1224. Treloar C, Rance J, Dore GJ, Grebely J: Client and staff experiences of a co-located service for the assessment and treatment of hepatitis C virus infection in the opioid substitution treatment setting: The ETHOS study. Suchtmedizin in Forschung und Praxis 2013, 15 (4):226.

1225. Treloar C, Rance J, Dore GJ, Grebely J: Barriers and facilitators for assessment and treatment of hepatitis C virus infection in the opioid substitution treatment setting: Insights from the ETHOS study. Journal of Viral Hepatitis 2014, 21(8):560-567.

1226. Treloar C, Rance J, Dore GJ, Grebely J, Group ES: Barriers and facilitators for assessment and treatment of hepatitis C virus infection in the opioid substitution treatment setting: insights from the ETHOS study. Journal of Viral Hepatitis 2014, 21(8):560-567.

1227. Treloar C, Rance J, Grebely J, Dore GJ: Client and staff experiences of a co-located service for hepatitis C care in opioid substitution treatment settings in New South Wales, Australia. Drug and Alcohol Dependence 2013, 133(2):529-534.

1228. Tross S, Feaster DJ, Thorens G, Duan R, Gomez Z, Pavlicova M, Hu MC, Kyle T, Erickson S, Spector A et al: Substance Use, Depression and Sociodemographic Determinants of HIV Sexual Risk Behavior in Outpatient Substance Abuse Treatment Patients. Journal of Addiction Medicine 2015, 9(6):457-463.

1229. Tso LS, Best J, Beanland R, Doherty M, Lackey M, Ma Q, Hall BJ, Yang B, Tucker JD: Facilitators and barriers in HIV linkage to care interventions: a qualitative evidence review. AIDS 2016, 30(10):1639-1653.

1230. Tsuyuki K, Surratt HL, Levi-Minzi MA, O'Grady CL, Kurtz SP: The Demand for Antiretroviral Drugs in the Illicit Marketplace: Implications for HIV Disease Management Among Vulnerable Populations. Aids Behav 2015, 19(5):857-868.

1231. Tu D, Belda P, Littlejohn D, Pedersen JS, Valle-Rivera J, Tyndall M: Adoption of the chronic care model to improve HIV care: in a marginalized, largely aboriginal population. Canadian Family Physician 2013, 59(6):650-657.

1232. Tubman JG, Oshri A, Taylor HL, Morris SL: Maltreatment clusters among youth in outpatient substance abuse treatment: co-occurring patterns of psychiatric symptoms and sexual risk behaviors. Archives of sexual behavior 2011, 40(2):301-309.

1233. Tun W, Sebastian MP, Sharma V, Madan I, Souidi S, Lewis D, Thior I, Sarna A: Strategies for recruiting injection drug users for HIV prevention services in Delhi, India. Harm Reduction Journal 2013, 10:16.

1234. Turner BJ, Laine C, Cohen A, Hauck WW: Effect of medical, drug abuse, and mental health care on receipt of dental care by drug users. Journal of Substance Abuse Treatment 2002, 23(3):239-246.

1235. Turner BJ, Markson LE, McKee LJ, Houchens R, Fanning T: Health care delivery, zidovudine use, and survival of women and men with AIDS. Journal of Acquired Immune Deficiency Syndromes 1994, 7(12):1250-1262.

1236. Turner BJ, Newschaffer CJ, Zhang D, Cosler L, Hauck WW: Antiretroviral use and pharmacy-based measurement of adherence in postpartum HIV-infected women. Medical care 2000, 38(9):911-925.

1237. Turner BJ, Taylor BS, Hanson JT, Perez ME, Hernandez L: First year experience of HCV screening of 2,765 baby boomers admitted to a safety net hospital in South Texas. Journal of General Internal Medicine 2014, 29:S99.

1238. Tweed E, Brant L, Hurrelle M, Klapper P, Ramsay M, Hepatitis Sentinel Surveillance Study G: Hepatitis C testing in sexual health services in England, 2002-7: results from sentinel surveillance. Sexually Transmitted Infections 2010, 86(2):126-130.

1239. Ulett KB, Willig JH, Lin HY, Routman JS, Abroms S, Allison J, Chatham A, Raper JL, Saag MS, Mugavero MJ: The therapeutic implications of timely linkage and early retention in HIV care. AIDS Patient Care STDS 2009, 23(1):41-49.

1240. Ungvarski PJ: Challenges for the urban home health care provider. The New York City experience. The Nursing clinics of North America 1996, 31(1):81-95.

1241. Upadhyay A, Jaber BL, Madias NE: Epidemiology of Hyponatremia. Seminars in Nephrology 2009, 29(3):227-238.

1242. Uphold CR, Mkanta WN: Use of health care services among persons living with HIV infection: State of the science and future directions. AIDS Patient Care and STDs 2005, 19(8):473-485.

1243. Uwimana J, Struthers P: Met and unmet palliative care needs of people living with HIV/AIDS in Rwanda. Sahara J 2007, 4(1):575-585.

1244. Vaghela JF, Kapoor SK, Kumar A, Dass RT, Khanna A, Bhatnagar AK: Home based care to multi-drug resistant tuberculosis patients: A pilot study. Indian Journal of Tuberculosis 2015, 62(2):91-96.

1245. van Andel E, Been SK, Rokx C, van der Ende ME: Risk factors in an HIV-infected population for refraining from specialist care. AIDS Care 2016, 28(10):1255-1260.

1246. van Beek I: Case study: Accessible primary health care-A foundation to improve health outcomes for people who inject drugs. International Journal of Drug Policy 2007, 18(4):329-332.

1247. van Beek I: Case study: accessible primary health care--a foundation to improve health outcomes for people who inject drugs. International Journal of Drug Policy 2007, 18(4):329-332.

1248. Van den Boom F: Point of view: AIDS in the family: A personal reflection. AIDS Patient Care 1991, 5(6):273-279.

1249. van der Laan LE, Garcia-Prats AJ, Schaaf HS, Tikiso T, Wiesner L, de Kock M, Winckler J, Norman J, McIlleron H, Denti P et al: Pharmacokinetics and drug-drug interactions of lopinavir/ritonavir administered with first and second-line antituberculosis drugs in HIV-infected children treated for multidrug-resistant tuberculosis. Antimicrob Agents Chemother 2017, 13:13.

1250. van der Laan LE, Garcia-Prats AJ, Schaaf HS, Tikiso T, Wiesner L, de Kock M, Winckler J, Norman J, McIlleron H, Denti P et al: Pharmacokinetics and Drug-Drug Interactions of Lopinavir-Ritonavir Administered with First- and Second-Line Antituberculosis Drugs in HIV-Infected Children Treated for Multidrug-Resistant Tuberculosis. Antimicrob Agents Chemother 2018, 62(2).

1251. Van Der Laan LE, Garcia-Prats AJ, Simon Schaaf H, Tikiso T, Wiesner L, De Kock M, Winckler J, Norman J, McIlleron H, Denti P et al: Pharmacokinetics and drug-drug interactions of lopinavir-ritonavir administered with first- and second-line antituberculosis drugs in HIV-infected children treated for multidrug-resistant tuberculosis. Antimicrobial Agents and Chemotherapy 2018, 62 (2) (no pagination)(e00420-17).

1252. Van Dyke RB: Pediatric human immunodeficiency virus infection and the acquired immunodeficiency syndrome. A health care crisis of children and families. Am J Dis Child 1991, 145(5):529-532.

1253. van Hest NA, De Vries G, Smit F, Grant AD, Richardus JH: Estimating the coverage of a targeted mobile tuberculosis screening programme among illicit drug users and homeless persons with truncated models. Epidemiol Infect 2008, 136(5):628-635.

1254. Van Thiel DH: The ABCs of chronic hepatitis: reason out of confusion. J Okla State Med Assoc 1995, 88(8):345-347.

1255. Vaughn A: Substance abuse, medications, HIV, and the community. Nurs Clin North Am 2006, 41(3):355-369, v.

1256. Veitsman E, Shahar E, Hassoun G, Lorber M, Kramskay R, Maor C, Pollack S, Baruch Y: [Outcome of treatment with peg-interferon and ribavirin in HIV-HCV co-infected patients: "real life" single center experience]. Harefuah 2012, 151(12):684-687, 721, 720.

1257. Venkatesan A, Nath A, Ming GL, Song H: Adult hippocampal neurogenesis: regulation by HIV and drugs of abuse. Cellular and molecular life sciences : CMLS 2007, 64(16):2120-2132.

1258. Vergara-Rodriguez PT, Watts J, Tozzi M, Bather-Gardner M, Arenas V: HIV substance treatment and recovery (H-STAR) a comprehensive treatment program for HIV positive persons with dual diagnosis. European Psychiatry Conference: 20th European Congress of Psychiatry, EPA 2012, 27(no pagination).

1259. Vetter C, Robert-Tissot L, Cottagnoud P, Stucki A: [Working under pressure]. Praxis 2008, 97(8):437-442.

1260. Visher CA, Hiller M, Belenko S, Pankow J, Dembo R, Frisman LK, Pearson FS, Swan H, Wiley TR: The effect of a local change team intervention on staff attitudes towards HIV service delivery in correctional settings: a randomized trial. AIDS Educ Prev 2014, 26(5):411-428.

1261. Vladimirsky SN, Soto SS, Altabert NR, Otegui LO, Munne MS, Brajterman LS, Del Monaco R, Gonzalez JE: Birth cohort and risk factors in Hepatitis C patients recorded at the Sentinel Units Network in Argentina 2007-2014. Journal of Viral Hepatitis 2015, 22:60-61.

1262. Volkow ND, Baler RD, Normand JL: The unrealized potential of addiction science in curbing the HIV epidemic. Current HIV Research 2011, 9(6):393-395.

1263. Volkow ND, Montaner J: The urgency of providing comprehensive and integrated treatment for substance abusers with HIV. Health Affairs 2011, 30(8):1411-1419.

1264. Volmink J, Garner P: Directly observed therapy for treating tuberculosis. Cochrane Database of Systematic Reviews 2007, (4) (no pagination)(CD003343).

1265. von Bardeleben U, Petitjean S, Stohler R, Natsch C, Ladewig D: [Substitution as a possibility for the treatment of opiate dependent patients]. Psychiatr Prax 1994, 21(1):7-9.

1266. von Bardeleben U, Stohler R, Petitjean S, Ladewig D: [Substitute drug treatments with methadone]. Ther Umsch 1993, 50(3):139-147.

1267. Wadland WC, Ferenchick GS: Medical comorbidity in addictive disorders. Psychiatric Clinics of North America 2004, 27(4):675-687.

1268. Wall TL, Sorensen JL, Batki SL, Delucchi KL, London JA, Chesney MA: Adherence to zidovudine (AZT) among HIV-infected methadone patients: a pilot study of supervised therapy and dispensing compared to usual care. Drug Alcohol Depend 1995, 37(3):261-269.

1269. Walley AY, Cheng DM, Pierce CE, Chen C, Filippell T, Samet JH, Alford DP: Methadone dose, take home status, and hospital admission among methadone maintenance patients. Journal of Addiction Medicine 2012, 6(3):186-190.

1270. Walley AY, Palmisano J, Sorensen-Alawad A, Chaisson C, Raj A, Samet JH, Drainoni ML: Engagement and Substance Dependence in a Primary Care-Based Addiction Treatment Program for People Infected with HIV and People at High-Risk for HIV Infection. Journal of Substance Abuse Treatment 2015, 59:59-66.

1271. Walley AY, Palmisano J, Sorensen-Alawad A, Duerfeldt K, Raj A, Allensworth-Davies D, Samet JH, Drainoni ML: The fast path program: Engagement and dependence outcomes in a primary care-based addiction treatment program. Journal of General Internal Medicine 2012, 27:S320-S321.

1272. Walley AY, Tetrault JM, Friedmann PD: Integration of substance use treatment and medical care: A special issue of JSAT. Journal of Substance Abuse Treatment 2012, 43(4):377-381.

1273. Ware PJ: The triple diagnosed. Caring 1995, 14(2):28-30, 32-23.

1274. Watson T, Hughes C: Pharmacists and harm reduction: A review of current practices and attitudes. Canadian Pharmacists Journal 2012, 145(3):124-127.e122.

1275. Watters JK, Estilo MJ, Clark GL, Lorvick J: Syringe and needle exchange as HIV/AIDS prevention for injection drug users. Jama 1994, 271(2):115-120.

1276. Weaver MR, Conover CJ, Proescholdbell RJ, Arno PS, Ang A, Uldall KK, Ettner SL: Cost-effectiveness analysis of integrated care for people with HIV, chronic mental illness and substance abuse disorders. Journal of Mental Health Policy and Economics 2009, 12(1):33-46.

1277. Weinberg DS, Murray HW: Coping with AIDS. The special problems of New York City. N Engl J Med 1987, 317(23):1469-1473.

1278. Weinstein LC, LaNoue M, Collins E, Henwood BF, Drake RE: Health care integration for formerly homeless people with serious mental illness. Journal of Dual Diagnosis 2013, 9(1):72-77.

1279. Weinstein ZM, Kim HW, Cheng DM, Quinn E, Hui D, Labelle CT, Drainoni ML, Bachman SS, Samet JH: Long-term retention in Office Based Opioid Treatment with buprenorphine. Journal of Substance Abuse Treatment 2017, 74:65-70.

1280. Weisbord JS, Trepka MJ, Zhang G, Smith IP, Brewer T: Prevalence of and risk factors for hepatitis C virus infection among STD clinic clientele in Miami, Florida. Sexually transmitted infections 2003, 79(1):E1.

1281. Weiser SD, Hatcher A, Frongillo EA, Guzman D, Riley ED, Bangsberg DR, Kushel MB: Food insecurity is associated with greater acute care utilization among hiv-infected homeless and marginally housed individuals in san francisco. Journal of General Internal Medicine 2013, 28(1):91-98.

1282. Weiss L, Egan JE, Botsko M, Netherland J, Fiellin DA, Finkelstein R: The BHIVES collaborative: Organization and evaluation of a multisite demonstration of integrated buprenorphine/naloxone and HIV treatment. Journal of Acquired Immune Deficiency Syndromes 2011, 56(SUPPL. 1):S7-S13.

1283. Weiss L, Netherland J, Egan JE, Flanigan TP, Fiellin DA, Finkelstein R, Altice FL: Integration of buprenorphine/naloxone treatment into HIV clinical care: Lessons from the BHIVES collaborative. Journal of Acquired Immune Deficiency Syndromes 2011, 56(SUPPL. 1):S68-S75.

1284. Weiss L, Netherland J, Egan JE, Flanigan TP, Fiellin DA, Finkelstein R, Altice FL, Collaborative B: Integration of buprenorphine/naloxone treatment into HIV clinical care: lessons from the BHIVES collaborative. J Acquir Immune Defic Syndr 2011, 56 Suppl 1:S68-75.

1285. Weizman T, Gelkopf M, Melamed Y, Adelson M, Bleich A: Cannabis abuse is not a risk factor for treatment outcome in methadone maintenance treatment: a 1-year prospective study in an Israeli clinic. Aust N Z J Psychiatry 2004, 38(1-2):42-46.

1286. Welch KJ: Correlates of alcohol and/or drug use among HIV-infected individuals. AIDS Patient Care and STDs 2000, 14(6):317-323.

1287. Wermuth L: Sexual partners of substance abuse clients: strategies for HIV/AIDS prevention. Journal of Substance Abuse Treatment 1996, 13(6):505-509.

1288. Whetten K, Reif S, Ostermann J, Pence BW, Swartz M, Whetten R, Conover C, Bouis S, Thielman N, Eron J: Improving health outcomes among individuals with HIV, mental illness, and substance use disorders in the Southeast. AIDS Care - Psychological and Socio-Medical Aspects of AIDS/HIV 2006, 18(SUPPL. 1):S18-S26.

1289. Whetten R, Whetten K, Pence BW, Reif S, Conover C, Bouis S: Does distance affect utilization of substance abuse and mental health services in the presence of transportation services? AIDS Care 2006, 18 Suppl 1:S27-34.

1290. Whitteker WB: Needle exchange programs benefit the community. Caring : National Association for Home Care magazine 1996, 15(8):46-47.

1291. Wick JY, Zanni GR: Challenges in caring for aging inmates. Consultant Pharmacist 2009, 24(6):424-436.

1292. Willemsen PA, Senden TF: [Estimation of the possibility of HIV or hepatitis B infection transmission in ambulatory care of substance dependence]. Ned Tijdschr Geneeskd 1993, 137(42):2149-2152.

1293. Willenbring ML: Integrating care for patients with infectious, psychiatric, and substance use disorders: Concepts and approaches. Aids 2005, 19(SUPPL. 3):S227-S237.

1294. Williams B, Murray M, Harris D, Conviser R: Careproviders' adherence to HIV standards of care is uniformly high in an integrated HIV care system. AIDS and Public Policy Journal 2003, 18(1-2):20-34.

1295. Williams-Nguyen J, Nance RM, Lindstrom S, Heckbert S, Peter I, Budoff M, Mathews WC, Eron JJ, Hunt PW, Moore RD et al: Hepatitis C virus and incident type 1 and type 2 myocardial infarction in HIV. Topics in Antiviral Medicine 2018, 26 (Supplement 1):271s-272s.

1296. Wimberly AS, Ivey M, Rennert L, McKay JR: Effect of Continuing Care for Cocaine Dependence on HIV Sex-Risk Behaviors. AIDS and behavior 2017, 21(4):1082-1090.

1297. Winiarski MG, Beckett E, Salcedo J: Outcomes of an inner-city HIV mental health programme integrated with primary care and emphasizing cultural responsiveness. AIDS Care 2005, 17(6):747-756.

1298. Winkelstein ER, Edlin BR, Szott K, Shu MA, McKnight C, DesJarlais DC, Bramson H, Woodin M, Flores De Leon L, Haberlen E: The SWAN Project: Integrating research and service with a cohort of young people who use drugs at risk for hepatitis C in New York City. Suchtmedizin in Forschung und Praxis 2013, 15 (4):238.

1299. Wissow LS, Tegegn T, Asheber K, McNabb M, Weldegebreal T, Jerene D, Ruff A: Collaboratively reframing mental health for integration of HIV care in Ethiopia. Health policy and planning 2015, 30(6):791-803.

1300. Wohl AR, Carlos JA, Tejero J, Dierst-Davies R, Daar ES, Khanlou H, Cadden J, Towner W, Frye D: Barriers and unmet need for supportive services for HIV patients in care in Los Angeles County, California. AIDS Patient Care STDS 2011, 25(9):525-532.

1301. Wolf L: A tuberculosis control plan for ambulatory care centers. The Nurse practitioner 1995, 20(6):34-36, 39-3640.

1302. Wong E, Pedrana A, Draper BL, Gold J, Richmond J, Stoove M, Doyle JS, Thompson AJ, Hellard ME: Eliminate hepatitis C partnership: Clinical site scoping. Journal of Gastroenterology and Hepatology (Australia) 2017, 32 (Supplement 2):84.

1303. Wood E, Milloy MJ, Montaner JS: HIV treatment as prevention among injection drug users. Curr Opin HIV AIDS 2012, 7(2):151-156.

1304. Wood E, Milloy MJ, Montaner JS: HIV treatment as prevention among injection drug users. Curr Opin HIV AIDS 2012, 7(2):151-156.

1305. Woodroffe MA, Hays H: Fentanyl transdermal system. Pain management at home. Canadian Family Physician 1997, 43(FEB.):268-272.

1306. Woolhouse S, Cooper E, Pickard A: "It gives me a sense of belonging": Providing integrated health care and treatment to people with HCV engaged in a psycho-educational support group. International Journal of Drug Policy 2013, 24(6):550-557.

1307. Woolley P: Management of HIV infection. Practitioner 1992, 236(1517):788-790.

1308. Xia YH, Chen W, Tucker JD, Wang C, Ling L: HIV and hepatitis C virus test uptake at methadone clinics in Southern China: opportunities for expanding detection of bloodborne infections. BMC public health 2013, 13:899.

1309. Xu LD: A decision support system for AIDS intervention and prevention. Int J Biomed Comput 1994, 36(4):281-291.

1310. Yaima N, Ningthouja S, Sharma D, Bijaya L, Shyamkanhai K, Narendra P, Lisam K, Agarwal AK: Lessons from home based care for persons affecting with HIV and AIDS. Indian J Public Health 1995, 39(3):113-115.

1311. Yanagawa B, Bahji A, Lamba W, Tan DH, Cheema A, Syed I, Verma S: Endocarditis in the setting of IDU: Multidisciplinary management. Current Opinion in Cardiology 2018, 33(2):140-147.

1312. Yang C, Crane HM, Cropsey K, Hutton H, Chander G, Saag M, McCaul ME: Implementation of Computer-delivered Brief Alcohol Intervention in HIV Clinical Settings: Who Agrees to Participate? J Addict Res Ther 2016, 7(2).

1313. Yellin H, Beckwith C, Kurth A, Liu T, Castonguay B, Patrick R, Trezza C, Bazerman L, Kuo I: Syndemic effect of mental illness and substance use on viral suppression among recently-incarcerated, HIV-infected individuals in the CARE+ Corrections study. AIDS Care - Psychological and Socio-Medical Aspects of AIDS/HIV 2018:1-5.

1314. Yew WW, Borgdorff MW, Enarson DA: New tools for global tuberculosis control: Are we any closer? International Journal of Tuberculosis and Lung Disease 2012, 16(7):855-856.

1315. Yi S, Tuot S, Chhoun P, Pal K, Choub SC, Mburu G: Prevalence and correlates of psychological distress among drug users in Phnom Penh, Cambodia. International Journal of Drug Policy 2016, 36:25-32.

1316. Yokaichiya CM, Figueiredo Wdos S, Schraiber LB: [Injecting drug users and antiretroviral therapy: perceptions of pharmacy teams]. Rev Saude Publica 2007, 41 Suppl 2:14-21.

1317. Young SR, Bishburg E, Boland M, Conviser R, Jackson J: Ambulatory AIDS care for those with special needs. Children, women, and drug users. Journal of Ambulatory Care Management 1988, 11(2):67-80.

1318. Young SR, Bishburg E, Boland M, Conviser R, Jackson J: Ambulatory AIDS care for those with special needs--children, women, and drug users. Journal of Ambulatory Care Management 1988, 11(2):67-80.

1319. Zala C, Rouleau D, Conway B: Is there anything left to learn? A report on the Fifth International Workshop on HIV Drug Resistance. Canadian Journal of Infectious Diseases 1998, 9(3):172-176.

1320. Zaller N, Gillani FS, Rich JD: A model of integrated primary care for HIV-positive patients with underlying substance use and mental illness. AIDS Care - Psychological and Socio-Medical Aspects of AIDS/HIV 2007, 19(9):1128-1133.

1321. Zanini B, Benini F, Pigozzi MG, Furba P, Giaco E, Cinquegrana A, Fasoli M, Lanzini A: Addicts with chronic hepatitis C: difficult to reach, manage or treat? World Journal of Gastroenterology 2013, 19(44):8011-8019.

1322. Zanini B, Covolo L, Donato F, Lanzini A: Effectiveness and tolerability of combination treatment of chronic hepatitis C in illicit drug users: meta-analysis of prospective studies. Clin Ther 2010, 32(13):2139-2159.

1323. Zanini B, Covolo L, Donato F, Lanzini A: Effectiveness and tolerability of combination treatment of chronic hepatitis C in illicit drug users: meta-analysis of prospective studies. Clin Ther 2010, 32(13):2139-2159.

1324. Zaw SK, Tun ST, Thida A, Aung TK, Maung W, Shwe M, Aye MM, Clevenbergh P: Prevalence of hepatitis C and B virus among patients infected with HIV: a cross-sectional analysis of a large HIV care programme in Myanmar. Tropical Doctor 2013, 43(3):113-115.

1325. Zaw SKK, Tun STT, Thida A, Aung TK, Maung W, Shwe M, Aye MM, Clevenbergh P: Prevalence of hepatitis C and B virus among patients infected with HIV: A cross-sectional analysis of a large HIV care programme in Myanmar. Tropical Doctor 2013, 43(3):113-115.

1326. Zeremski M, Zibbell JE, Martinez AD, Kritz S, Smith BD, Talal AH: Hepatitis C virus control among persons who inject drugs requires overcoming barriers to care. World Journal of Gastroenterology 2013, 19(44):7846-7851.

1327. Zhu J: Molecular mechanism of HIV-1 Tat interacting with human dopamine transporter. Journal of Neuroimmune Pharmacology 2017, 12 (1 Supplement 1):S20-S21.

1328. Zhu J, Ananthan S, Zhan CG: The role of human dopamine transporter in NeuroAIDS. Pharmacology and Therapeutics 2017.

1329. Zhu J, Ananthan S, Zhan CG: The role of human dopamine transporter in NeuroAIDS. Pharmacology and Therapeutics 2018, 183:78-89.

1330. Zibbell JE, Iqbal K, Patel RC, Suryaprasad A, Sanders KJ, Moore-Moravian L, Serrecchia J, Blankenship S, Ward JW, Holtzman D: Increases in hepatitis C virus infection related to injection drug use among persons aged <=30 years - Kentucky, Tennessee, Virginia, and West Virginia, 2006-2012. Mmwr 2015, Morbidity and mortality weekly report. 64(17):453-458.

1331. Zibbell JE, Iqbal K, Patel RC, Suryaprasad A, Sanders KJ, Moore-Moravian L, Serrecchia J, Blankenship S, Ward JW, Holtzman D et al: Increases in hepatitis C virus infection related to injection drug use among persons aged <=30 years - Kentucky, Tennessee, Virginia, and West Virginia, 2006-2012. MMWR - Morbidity & Mortality Weekly Report 2015, 64(17):453-458.

1332. Zimmerli W: Current antibiotics for clinical practice. [German]. Schweizerische Rundschau fur Medizin Praxis = Revue suisse de medecine Praxis 1996, 85(40):1240-1244.

1333. Zimmerli W: [Current antibiotics for clinical practice]. Praxis 1996, 85(40):1240-1244.

1334. Zinkernagel C, Taffe P, Rickenbach M, Amiet R, Ledergerber B, Volkart AC, Rauchfleisch U, Kiss A, Werder V, Vernazza P et al: Importance of mental health assessment in HIV-infected outpatients. J Acquir Immune Defic Syndr 2001, 28(3):240-249.

1335. Zucker DM, Choi J, Gallagher ER: Mobile outreach strategies for screening hepatitis and HIV in high-risk populations. Public Health Nursing 2012, 29(1):27-35.

1336. Zule WA, Lam WK, Wechsberg WM: Treatment readiness among out-of-treatment African-American crack users. Journal of Psychoactive Drugs 2003, 35(4):503-510.

1337. Zurovac D, Ndhlovu M, Rowe AK, Hamer DH, Thea DM, Snow RW: Treatment of paediatric malaria during a period of drug transition to artemether-lumefantrine in Zambia: cross sectional study. Bmj 2005, 331(7519):734.

1338. Zweben JE: Hepatitis C: education and counseling issues. Journal of Addictive Diseases 2001, 20(1):33-42.

Additional file 2

References and crosseferences from relevant reviews and randomized controlled trials:

1. Human immunodeficiency virus infection in the United States: a review of current knowledge. MMWR supplements 1987, 36(6):1-48.

2. Management of possible sexual, injecting-drug-use, or other nonoccupational exposure to HIV, including considerations related to antiretroviral therapy. Public Health Service statement. Centers for Disease Control and Prevention. MMWR Recomm Rep 1998, 47(Rr-17):1-14.

3. [Consensus document on tuberculosis prevention and control in Spain. Research Unit on Tuberculosis of Barcelona (UITB). Area of Tuberculosis and Respiratory Infections (TIR) of the Spanish Society of Pneumology and Thoracic Surgery (SEPAR) and Study Group on Aids (GESIDA) of the Spanish Society of Infectious Diseases and Clinical Microbiology (SEIMC)]. Medicina clinica 1999, 113(18):710-715.

4. [International consensus conference on hepatitis C. Conclusions. Organized under the auspices of the European Association for the Study of the Liver (EASL)]. Ann Med Interne (Paris) 1999, 150(5):449-453.

5. Hepatitis C. Shedding light on the shadow epidemic. Harvard health letter 1999, 24(9):1-3.

6. Consensus conference. Treatment of hepatitis C. Gastroenterol Clin Biol 2002, 26 Spec No 2:B303-320.

7. Phosphatidylcholine. Alternative medicine review : a journal of clinical therapeutic 2002, 7(2):150-154.

8. National Institutes of Health Consensus Development Conference statement Management of Hepatitis C: 2002 June 10-12, 2002. HIV clinical trials 2003, 4(1):55-75.

9. [Indications for hepatic transplantation -- January 19 and 20, 2005, Lyon (Palais des congres)]. Journal de chirurgie 2005, 142(3):177-183.

10. Expert Consensus Conference. The screening for hepatitis C virus infection in adults in Italy, May 5-6, 2005. Dig Liver Dis 2006, 38(7):445-451.

11. Abdul-Quader AS, Feelemyer J, Modi S, Stein ES, Briceno A, Semaan S, Horvath T, Kennedy GE, Des Jarlais DC: Effectiveness of structural-level needle/syringe programs to reduce HCV and HIV infection among people who inject drugs: a systematic review. Aids Behav 2013, 17(9):2878-2892.

12. Abougergi MS, Rai R, Cohen CK, Montgomery R, Solga SF: Trends in adult-to-adult living donor liver transplant organ donation: the Johns Hopkins experience. Progress in transplantation (Aliso Viejo, Calif) 2006, 16(1):28-32.

13. Aceijas C, Rhodes T: Global estimates of prevalence of HCV infection among injecting drug users. The International journal on drug policy 2007, 18(5):352-358.

14. Acosta EP, Wu H, Hammer SM, Yu S, Kuritzkes DR, Walawander A, Eron JJ, Fichtenbaum CJ, Pettinelli C, Neath D et al: Comparison of two indinavir/ritonavir regimens in the treatment of HIV-infected individuals. J Acquir Immune Defic Syndr 2004, 37(3):1358-1366.

15. Adams HG, Jordan C: Infections in the alcoholic. The Medical clinics of North America 1984, 68(1):179-200.

16. Adams PC: Iron overload in viral and alcoholic liver disease. J Hepatol 1998, 28 Suppl 1:19-20.

17. Adnams CM: Perspectives of intellectual disability in South Africa: epidemiology, policy, services for children and adults. Curr Opin Psychiatry 2010, 23(5):436-440.

18. Aguirrebengoa L, Montejo M, Urkijo JC, Urra E, Gutierrez A, Mendoza F, Zalacain R, Gonzalez de Zarate P, Aguirre C: [Tuberculosis and AIDS. Study of 54 patients]. Enferm Infecc Microbiol Clin 1991, 9(7):399-404.

19. Ahmad N: Molecular mechanisms of HIV-1 mother-to-child transmission and infection in neonatal target cells. Life Sci 2011, 88(21-22):980-986.

20. Aiuti F, Sirianni MC, Mezzaroma I, D'Offizi GP, Pesce AM, Papetti C, Ensoli F, Luzi G: HIV-1 infection: epidemiological features and immunological alterations during the natural history of the disease. Clinical immunology and immunopathology 1989, 50(1 Pt 2):S157-165.

21. Akinosoglou K, Apostolakis E, Marangos M, Pasvol G: Native valve right sided infective endocarditis. European journal of internal medicine 2013, 24(6):510-519.

22. Albeldawi M, Ruiz-Rodriguez E, Carey WD: Hepatitis C virus: Prevention, screening, and interpretation of assays. Cleve Clin J Med 2010, 77(9):616-626.

23. Alberti A: What are the comorbidities influencing the management of patients and the response to therapy in chronic hepatitis C? Liver Int 2009, 29 Suppl 1:15-18.

24. Alberti A, Vario A, Ferrari A, Pistis R: Review article: chronic hepatitis C--natural history and cofactors. Aliment Pharmacol Ther 2005, 22 Suppl 2:74-78.

25. Aldersley MA, O'Grady JG: Hepatic disorders. Features and appropriate management. Drugs 1995, 49(1):83-102.

26. Ali S, Champagne DL, Spaink HP, Richardson MK: Zebrafish embryos and larvae: a new generation of disease models and drug screens. Birth defects research Part C, Embryo today : reviews 2011, 93(2):115-133.

27. Almeda J, Casabona J, Allepuz A, Garcia-Alcaide F, del Romero J, Tural C, Colm J, Bolao F, Campins M, Dominguez A et al: [Recommendations for non-occupational postexposure HIV prophylaxis. Spanish Working Group on Non-Occupational Postexposure HIV Prophylaxis of the Catalonian Center for Epidemiological Studies on AIDS and the AIDS Study Group]. Enferm Infecc Microbiol Clin 2002, 20(8):391-400.

28. Alonso M, Gutzman A, Mazin R, Pinzon CE, Reveiz L, Ghidinelli M: Hepatitis C in key populations in Latin America and the Caribbean: systematic review and meta-analysis. International journal of public health 2015, 60(7):789-798.

29. Alscher DM, Bode JC: [Liver diseases caused by alcohol consumption and hepatitis C virus infection: association, addition or potentiation?]. Deutsche medizinische Wochenschrift (1946) 2001, 126(5):117-122.

30. Alter MJ: The epidemiology of acute and chronic hepatitis C. Clinics in liver disease 1997, 1(3):559-568, vi-vii.

31. Alter MJ: Epidemiology of hepatitis C virus infection. World J Gastroenterol 2007, 13(17):2436-2441.

32. Alter MJ: HCV routes of transmission: what goes around comes around. Seminars in liver disease 2011, 31(4):340-346.

33. Alter MJ, Hadler SC: Delta hepatitis and infection in North America. Progress in clinical and biological research 1993, 382:243-250.

34. Altice FL, Azbel L, Stone J, Brooks-Pollock E, Smyrnov P, Dvoriak S, Taxman FS, El-Bassel N, Martin NK, Booth R et al: The perfect storm: incarceration and the high-risk environment perpetuating transmission of HIV, hepatitis C virus, and tuberculosis in Eastern Europe and Central Asia. Lancet 2016, 388(10050):1228-1248.

35. Altice FL, Kamarulzaman A, Soriano VV, Schechter M, Friedland GH: Treatment of medical, psychiatric, and substance-use comorbidities in people infected with HIV who use drugs. Lancet 2010, 376(9738):367-387.

36. Altice FL, Maru DS, Bruce RD, Springer SA, Friedland GH: Superiority of directly administered antiretroviral therapy over self-administered therapy among HIV-infected drug users: a prospective, randomized, controlled trial. Clin Infect Dis 2007, 45(6):770-778.

37. Altice FL, Mezger JA, Hodges J, Bruce RD, Marinovich A, Walton M, Springer SA, Friedland GH: Developing a directly administered antiretroviral therapy intervention for HIV-infected drug users: implications for program replication. Clin Infect Dis 2004, 38 Suppl 5:S376-387.

38. Amirkhanian YA, Kelly JA, Takacs J, Kuznetsova AV, DiFranceisco WJ, Mocsonaki L, McAuliffe TL, Khoursine RA, Toth TP: HIV/STD prevalence, risk behavior, and substance use patterns and predictors in Russian and Hungarian sociocentric social networks of men who have sex with men. AIDS Educ Prev 2009, 21(3):266-279.

39. Anand BS, Velez M: Influence of chronic alcohol abuse on hepatitis C virus replication. Digestive diseases (Basel, Switzerland) 2000, 18(3):168-171.

40. Ang DC, Calabrese LH: A common-sense approach to chronic fatigue in primary care. Cleve Clin J Med 1999, 66(6):343-350, 352.

41. Angeli P, Volpin R, Gerunda G, Craighero R, Roner P, Merenda R, Amodio P, Sticca A, Caregaro L, Maffei-Faccioli A et al: Reversal of type 1 hepatorenal syndrome with the administration of midodrine and octreotide. Hepatology 1999, 29(6):1690-1697.

42. Anthony IC, Bell JE: The Neuropathology of HIV/AIDS. International review of psychiatry (Abingdon, England) 2008, 20(1):15-24.

43. Aquinas M: Short-course therapy for tuberculosis. Drugs 1982, 24(2):118-132.

44. Arain A, De Sousa J, Corten K, Verrando R, Thijs H, Mathei C, Buntinx F, Robaeys G: Pilot Study: Combining Formal and Peer Education with FibroScan to Increase HCV Screening and Treatment in Persons who use Drugs. J Subst Abuse Treat 2016, 67:44-49.

45. Arain A, Robaeys G: Eligibility of persons who inject drugs for treatment of hepatitis C virus infection. World J Gastroenterol 2014, 20(36):12722-12733.

46. Arain A, Robaeys G, Stover H: Hepatitis C in European prisons: a call for an evidence-informed response. BMC Infect Dis 2014, 14 Suppl 6:S17.

47. Arase Y: [Acute exacerbation by reactivation of HBV in steroid withdrawal therapy]. Nihon rinsho Japanese journal of clinical medicine 1995, 53 Suppl(Pt 2):522-526.

48. Armengol C, Bartoli R, Sanjurjo L, Serra I, Amezaga N, Sala M, Sarrias MR: Role of scavenger receptors in the pathophysiology of chronic liver diseases. Critical reviews in immunology 2013, 33(1):57-96.

49. Arora S, Young S, Kodali S, Singal AK: Hepatic porphyria: A narrative review. Indian journal of gastroenterology : official journal of the Indian Society of Gastroenterology 2016, 35(6):405-418.

50. Aryanpur M, Hosseini M, Masjedi MR, Mortaz E, Tabarsi P, Soori H, Emami H, Heidari G, Dizagie MK, Baikpour M: A randomized controlled trial of smoking cessation methods in patients newly-diagnosed with pulmonary tuberculosis. BMC Infect Dis 2016, 16:369.

51. Aspinall EJ, Corson S, Doyle JS, Grebely J, Hutchinson SJ, Dore GJ, Goldberg DJ, Hellard ME: Treatment of hepatitis C virus infection among people who are actively injecting drugs: a systematic review and meta-analysis. Clin Infect Dis 2013, 57 Suppl 2:S80-89.

52. Aubisson S, Carrieri P, Lovell AM, Ben Diane MK, Peretti-Watel P, Spire B: [New tools for preventing and evaluating risk practices for hepatitis C transmission among injection drug users: some reflections on injection rooms and the measurement of risk-taking behaviors]. Rev Epidemiol Sante Publique 2006, 54 Spec No 1:1s69-61s75.

53. Awaisu A, Nik Mohamed MH, Mohamad Noordin N, Abd Aziz N, Syed Sulaiman SA, Muttalif AR, Ahmad Mahayiddin A: The SCIDOTS Project: evidence of benefits of an integrated tobacco cessation intervention in tuberculosis care on treatment outcomes. Substance abuse treatment, prevention, and policy 2011, 6:26.

54. Azar MM, Springer SA, Meyer JP, Altice FL: A systematic review of the impact of alcohol use disorders on HIV treatment outcomes, adherence to antiretroviral therapy and health care utilization. Drug Alcohol Depend 2010, 112(3):178-193.

55. Backmund M: [Illegal drug users and risk factors for HIV and HCV infection]. Bundesgesundheitsblatt, Gesundheitsforschung, Gesundheitsschutz 2007, 50(4):471-475.

56. Backmund M, Reimer J, Meyer K, Gerlach JT, Zachoval R: Hepatitis C virus infection and injection drug users: prevention, risk factors, and treatment. Clin Infect Dis 2005, 40 Suppl 5:S330-335.

57. Bagatin E, Goncalves VL, Sabongi VP, Sotto MN: Paracoccidioidomycosis in a patient with HIV-1 infection. Revista paulista de medicina 1992, 110(5):193-194.

58. Baggaley RF, Boily MC, White RG, Alary M: Risk of HIV-1 transmission for parenteral exposure and blood transfusion: a systematic review and meta-analysis. Aids 2006, 20(6):805-812.

59. Bai YQ, Yang YX, Yang YG, Ding SZ, Jin FL, Cao MB, Zhang YR, Zhang BY: Outcomes of autologous bone marrow mononuclear cell transplantation in decompensated liver cirrhosis. World J Gastroenterol 2014, 20(26):8660-8666.

60. Bailly D, Dervaux A, Servant D, Parquet PJ: Prevalence of hepatitis B virus, delta agent and human immunodeficiency virus infections in drug addicts. Biomedicine & pharmacotherapy = Biomedecine & pharmacotherapie 1989, 43(6):431-437.

61. Bajis S, Dore GJ, Hajarizadeh B, Cunningham EB, Maher L, Grebely J: Interventions to enhance testing, linkage to care and treatment uptake for hepatitis C virus infection among people who inject drugs: A systematic review. The International journal on drug policy 2017, 47:34-46.

62. Balaian MS: [Acquired immunodeficiency syndrome]. Klinicheskaia meditsina 1984, 62(9):8-15.

63. Balasubramanian A, Groopman JE, Ganju RK: Underlying pathophysiology of HCV infection in HIV-positive drug users. J Addict Dis 2008, 27(2):75-82.

64. Balasubramanian S, Kowdley KV: Effect of alcohol on viral hepatitis and other forms of liver dysfunction. Clinics in liver disease 2005, 9(1):83-101.

65. Balla AK, Lischner HW, Pomerantz RJ, Bagasra O: Human studies on alcohol and susceptibility to HIV infection. Alcohol (Fayetteville, NY) 1994, 11(2):99-103.

66. Banerjee A, Strazza M, Wigdahl B, Pirrone V, Meucci O, Nonnemacher MR: Role of mu-opioids as cofactors in human immunodeficiency virus type 1 disease progression and neuropathogenesis. Journal of neurovirology 2011, 17(4):291-302.

67. Banks WA: Physiology and pathology of the blood-brain barrier: implications for microbial pathogenesis, drug delivery and neurodegenerative disorders. Journal of neurovirology 1999, 5(6):538-555.

68. Bao YP, Liu ZM: Systematic review of HIV and HCV infection among drug users in China. Int J STD AIDS 2009, 20(6):399-405.

69. Bao YP, Liu ZM, Lu L: Review of HIV and HCV infection among drug users in China. Curr Opin Psychiatry 2010, 23(3):187-194.

70. Baral S, Sherman SG, Millson P, Beyrer C: Vaccine immunogenicity in injecting drug users: a systematic review. The Lancet Infectious diseases 2007, 7(10):667-674.

71. Barry J, Bourke M, Buckley M, Coughlan B, Crowley D, Cullen W, Dooley S, Keating S, Kelleher D, Moloney J et al: Hepatitis C among drug users: consensus guidelines on management in general practice. Ir J Med Sci 2004, 173(3):145-150.

72. Bassendine MF: Alcohol--a major risk factor for hepatocellular carcinoma? J Hepatol 1986, 2(3):513-519.

73. Bastie A, Pawlotsky JM, Roudot-Thoraval F, Dhumeaux D: [Hepatitis C virus infection. Epidemiology]. Pathologie-biologie 1995, 43(8):674-680.

74. Bastos FI, Caiaffa W, Rossi D, Vila M, Malta M: The children of mama coca: coca, cocaine and the fate of harm reduction in South America. The International journal on drug policy 2007, 18(2):99-106.

75. Basu D: Overview of substance abuse and hepatitis C virus infection and co-infections in India. J Neuroimmune Pharmacol 2010, 5(4):496-506.

76. Bataller R, North KE, Brenner DA: Genetic polymorphisms and the progression of liver fibrosis: a critical appraisal. Hepatology 2003, 37(3):493-503.

77. Batki SL, Canfield KM, Ploutz-Snyder R: Psychiatric and substance use disorders among methadone maintenance patients with chronic hepatitis C infection: effects on eligibility for hepatitis C treatment. Am J Addict 2011, 20(4):312-318.

78. Batki SL, Gruber VA, Bradley JM, Bradley M, Delucchi K: A controlled trial of methadone treatment combined with directly observed isoniazid for tuberculosis prevention in injection drug users. Drug Alcohol Depend 2002, 66(3):283-293.

79. Battistella M: [Case no. 5. Bullous dermatosis]. Annales de pathologie 2013, 33(3):196-201.

80. Bauer LO, Shanley JD: ASPD blunts the effects of HIV and antiretroviral treatment on event-related brain potentials. Neuropsychobiology 2006, 53(1):17-25.

81. Baum MK: Role of micronutrients in HIV-infected intravenous drug users. J Acquir Immune Defic Syndr 2000, 25 Suppl 1:S49-52.

82. Bean P: HIV and alcohol use: consequences of comorbidity. American clinical laboratory 2001, 20(7):13-16.

83. Beckebaum S, Cicinnati VR, Broelsch CE, Gerken G: [Recurrent disease after liver transplantation. A therapeutic dilemma or treatable?]. Medizinische Klinik (Munich, Germany : 1983) 2006, 101(12):939-950.

84. Bednasz CJ, Venuto CS, Ma Q, Morse GD: Pharmacokinetic Considerations for Combining Antiretroviral Therapy, Direct-Acting Antiviral Agents for Hepatitis C Virus, and Addiction Treatment Medications. Clinical pharmacology in drug development 2017, 6(2):135-139.

85. Bedossa P: [Fibrosis in chronic hepatitis C infection: mechanisms and cofactors]. Gastroenterol Clin Biol 2002, 26 Spec No 2:B163-167.

86. Bedossa P, Callard P: [Alcoholic liver disease. From morphology to pathogenesis]. Annales de pathologie 1995, 15(5):324-331.

87. Begriche K, Massart J, Robin MA, Borgne-Sanchez A, Fromenty B: Drug-induced toxicity on mitochondria and lipid metabolism: mechanistic diversity and deleterious consequences for the liver. J Hepatol 2011, 54(4):773-794.

88. Beilke MA: Retroviral coinfections: HIV and HTLV: taking stock of more than a quarter century of research. AIDS research and human retroviruses 2012, 28(2):139-147.

89. Bekerman C, Hoffer PB, Bitran JD, Gupta RG: Gallium-67 citrate imaging studies of the lung. Seminars in nuclear medicine 1980, 10(3):286-301.

90. Belcher JM, Parikh CR, Garcia-Tsao G: Acute kidney injury in patients with cirrhosis: perils and promise. Clin Gastroenterol Hepatol 2013, 11(12):1550-1558.

91. Bell JE, Arango JC, Anthony IC: Neurobiology of multiple insults: HIV-1-associated brain disorders in those who use illicit drugs. J Neuroimmune Pharmacol 2006, 1(2):182-191.

92. Bell JE, Arango JC, Robertson R, Brettle RP, Leen C, Simmonds P: HIV and drug misuse in the Edinburgh cohort. J Acquir Immune Defic Syndr 2002, 31 Suppl 2:S35-42.

93. Bellentani S, Tiribelli C: The spectrum of liver disease in the general population: lesson from the Dionysos study. J Hepatol 2001, 35(4):531-537.

94. Belman AL: HIV-1 infection and AIDS. Neurologic clinics 2002, 20(4):983-1011.

95. Benitez Bribiesca L: [Are the HIV viruses really the causal agents of AIDS?]. Gaceta medica de Mexico 1991, 127(1):75-84.

96. Berg KM, Litwin AH, Li X, Heo M, Arnsten JH: Lack of sustained improvement in adherence or viral load following a directly observed antiretroviral therapy intervention. Clin Infect Dis 2011, 53(9):936-943.

97. Bergenstrom AM, Abdul-Quader AS: Injection drug use, HIV and the current response in selected low-income and middle-income countries. Aids 2010, 24 Suppl 3:S20-29.

98. Bergheim I, Eagon PK, Dooley S, Breitkopf-Heinlein K: Alcoholic liver disease and exacerbation by malnutrition and infections: what animal models are currently available? Annals of the New York Academy of Sciences 2011, 1216:41-49.

99. Bergk A, Berg T, Neumann UP: Risk factors for the acquisition of hepatitis C. Minerva gastroenterologica e dietologica 2005, 51(1):7-14.

100. Berkelman RL, Heyward WL, Stehr-Green JK, Curran JW: Epidemiology of human immunodeficiency virus infection and acquired immunodeficiency syndrome. The American journal of medicine 1989, 86(6 Pt 2):761-770.

101. Bernard PH: [Hepatitis B and C virological tests: interpretation and practical results in women]. Gynecologie, obstetrique & fertilite 2005, 33(6):423-428.

102. Bernardo J: Tuberculosis: a disease of the 1990s. Hospital practice (Office ed) 1991, 26(10):195-198, 202, 207-198 passim.

103. Beyoglu D, Idle JR: The metabolomic window into hepatobiliary disease. J Hepatol 2013, 59(4):842-858.

104. Bhattacharya R, Shuhart MC: Hepatitis C and alcohol: interactions, outcomes, and implications. J Clin Gastroenterol 2003, 36(3):242-252.

105. Birkhead GS, Klein SJ, Candelas AR, O'Connell DA, Rothman JR, Feldman IS, Tsui DS, Cotroneo RA, Flanigan CA: Integrating multiple programme and policy approaches to hepatitis C prevention and care for injection drug users: a comprehensive approach. The International journal on drug policy 2007, 18(5):417-425.

106. Bjerver K: [Complications of alcoholism reflected in morbidity among alcoholics]. Lakartidningen 1974, 71(20):2055-2058.

107. Blaser MJ, Cohn DL: Opportunistic infections in patients with AIDS: clues to the epidemiology of AIDS and the relative virulence of pathogens. Reviews of infectious diseases 1986, 8(1):21-30.

108. Blum HE: [Hepatocellular carcinoma: molecular biology aspects]. Zentralblatt fur Chirurgie 1994, 119(11):759-763.

109. Blum HE: [Hepatitis 2004: an update]. Ther Umsch 2004, 61(8):477-479.

110. Blum HE: [Chronic liver disease--diagnostic work-up]. Praxis (Bern 1994) 2006, 95(34):1271-1274.

111. Boesecke C, Wedemeyer H, Rockstroh JK: Diagnosis and treatment of acute hepatitis C virus infection. Infectious disease clinics of North America 2012, 26(4):995-1010.

112. Boldorini R, Vigano P, Monga G, Nebuloni M, Cargnel A, Gubertini G, Migliaretti G, Costanzi G: Hepatic histology of patients with HIV infection and chronic hepatitis C treated with interferon. Journal of clinical pathology 1997, 50(9):735-740.

113. Bonkovsky HL, Lambrecht RW, Shan Y: Iron as a co-morbid factor in nonhemochromatotic liver disease. Alcohol (Fayetteville, NY) 2003, 30(2):137-144.

114. Bonkovsky HL, Tice AD, Yapp RG, Bodenheimer HC, Jr., Monto A, Rossi SJ, Sulkowski MS: Efficacy and safety of peginterferon alfa-2a/ribavirin in methadone maintenance patients: randomized comparison of direct observed therapy and self-administration. The American journal of gastroenterology 2008, 103(11):2757-2765.

115. Bonner JE, Barritt ASt, Fried MW, Evon DM: Tangible resources for preparing patients for antiviral therapy for chronic hepatitis C. Dig Dis Sci 2012, 57(6):1439-1444.

116. Bonner JE, Barritt ASt, Fried MW, Evon DM: Time to rethink antiviral treatment for hepatitis C in patients with coexisting mental health/substance abuse issues. Dig Dis Sci 2012, 57(6):1469-1474.

117. Booth JC: Chronic hepatitis C: the virus, its discovery and the natural history of the disease. J Viral Hepat 1998, 5(4):213-222.

118. Bosch FX, Ribes J, Cleries R, Diaz M: Epidemiology of hepatocellular carcinoma. Clinics in liver disease 2005, 9(2):191-211, v.

119. Bowman S, Grau LE, Singer M, Scott G, Heimer R: Factors associated with hepatitis B vaccine series completion in a randomized trial for injection drug users reached through syringe exchange programs in three US cities. BMC Public Health 2014, 14:820.

120. Bradshaw D, Matthews G, Danta M: Sexually transmitted hepatitis C infection: the new epidemic in MSM? Current opinion in infectious diseases 2013, 26(1):66-72.

121. Braham A, Safer L, Bdioui F, Lejmi A, Brahmi I, Ben Chabenne N, Ben Mimoun H, Saffar H: [Antiphospholipid antibodies in digestive diseases]. Presse Med 2001, 30(38):1890-1897.

122. Bramstedt KA, Stowe J, Kotz M: Shopping for a transplant: when noncompliant patients seek wait listing at multiple hospitals. Progress in transplantation (Aliso Viejo, Calif) 2004, 14(3):217-221.

123. Brechot C, Kremsdorf D, Soussan P, Pineau P, Dejean A, Paterlini-Brechot P, Tiollais P: Hepatitis B virus (HBV)-related hepatocellular carcinoma (HCC): molecular mechanisms and novel paradigms. Pathologie-biologie 2010, 58(4):278-287.

124. Brechot C, Nalpas B, Feitelson MA: Interactions between alcohol and hepatitis viruses in the liver. Clinics in laboratory medicine 1996, 16(2):273-287.

125. Breitschwerdt B, Goeser T, Tissot JD, Trendelenburg M, Sis-Kluzik JM, Andrassy K: Hepatitis virus-related and ethanol-induced chronic liver disease with or without cryoglobulins--is there a difference concerning clinical or laboratory manifestation? Infection 1999, 27(4-5):248-251.

126. Brenner BG, Ibanescu RI, Hardy I, Roger M: Genotypic and Phylogenetic Insights on Prevention of the Spread of HIV-1 and Drug Resistance in "Real-World" Settings. Viruses 2017, 10(1).

127. Brewer C, Wong VS: Naltrexone: report of lack of hepatotoxicity in acute viral hepatitis, with a review of the literature. Addiction biology 2004, 9(1):81-87.

128. Bronowicki JP, Weber-Larivaille F, Gut JP, Doffoel M, Vetter D: [Comparison of immunogenicity of vaccination and serovaccination against hepatitis B virus in patients with alcoholic cirrhosis]. Gastroenterol Clin Biol 1997, 21(11):848-853.

129. Brown LS, Jr.: Enrollment of drug abusers in HIV clinical trials: a public health imperative for communities of color. J Psychoactive Drugs 1993, 25(1):45-52.

130. Bruce RD, Eiserman J, Acosta A, Gote C, Lim JK, Altice FL: Developing a modified directly observed therapy intervention for hepatitis C treatment in a methadone maintenance program: implications for program replication. Am J Drug Alcohol Abuse 2012, 38(3):206-212.

131. Bruggmann P, Litwin AH: Models of care for the management of hepatitis C virus among people who inject drugs: one size does not fit all. Clin Infect Dis 2013, 57 Suppl 2:S56-61.

132. Bruix J, Raoul JL, Sherman M, Mazzaferro V, Bolondi L, Craxi A, Galle PR, Santoro A, Beaugrand M, Sangiovanni A et al: Efficacy and safety of sorafenib in patients with advanced hepatocellular carcinoma: subanalyses of a phase III trial. J Hepatol 2012, 57(4):821-829.

133. Brummer DL: Preventive therapy of tuberculosis. Annual review of medicine 1974, 25:115-122.

134. Brust JC, Litwin AH, Berg KM, Li X, Heo M, Arnsten JH: Directly observed antiretroviral therapy in substance abusers receiving methadone maintenance therapy does not cause increased drug resistance. AIDS research and human retroviruses 2011, 27(5):535-541.

135. Buch S, Yao H, Guo M, Mori T, Mathias-Costa B, Singh V, Seth P, Wang J, Su TP: Cocaine and HIV-1 interplay in CNS: cellular and molecular mechanisms. Curr HIV Res 2012, 10(5):425-428.

136. Buch S, Yao H, Guo M, Mori T, Su TP, Wang J: Cocaine and HIV-1 interplay: molecular mechanisms of action and addiction. J Neuroimmune Pharmacol 2011, 6(4):503-515.

137. Buchanan R, Khakoo SI, Coad J, Grellier L, Parkes J: Hepatitis C bio-behavioural surveys in people who inject drugs-a systematic review of sensitivity to the theoretical assumptions of respondent driven sampling. Harm Reduct J 2017, 14(1):44.

138. Buckner CM, Luers AJ, Calderon TM, Eugenin EA, Berman JW: Neuroimmunity and the blood-brain barrier: molecular regulation of leukocyte transmigration and viral entry into the nervous system with a focus on neuroAIDS. J Neuroimmune Pharmacol 2006, 1(2):160-181.

139. Buffet C, Gagnepain A, Hagege H, Balian P, Espinoza P: [B virus, delta agent and human immunodeficiency virus infections in drug addicts]. Presse Med 1988, 17(30):1533-1537.

140. Burra P, Belli LS, Ginanni Corradini S, Volpes R, Marzioni M, Giannini E, Toniutto P: Common issues in the management of patients in the waiting list and after liver transplantation. Dig Liver Dis 2017, 49(3):241-253.

141. Burra P, Lucey MR: Liver transplantation in alcoholic patients. Transplant international : official journal of the European Society for Organ Transplantation 2005, 18(5):491-498.

142. Buss G, Cattin V, Spring P, Malinverni R, Gilliet M: Two cases of interferon-alpha-induced sarcoidosis Koebnerized along venous drainage lines: new pathogenic insights and review of the literature of interferon-induced sarcoidosis. Dermatology (Basel, Switzerland) 2013, 226(4):289-297.

143. Cabral GA: Drugs of abuse, immune modulation, and AIDS. J Neuroimmune Pharmacol 2006, 1(3):280-295.

144. Cadranel JF, Nousbaum JB: [Current trends in liver biopsy indications in chronic liver diseases]. Presse Med 2012, 41(11):1064-1070.

145. Cai Y, Yang L, Callen S, Buch S: Multiple Faceted Roles of Cocaine in Potentiation of HAND. Curr HIV Res 2016, 14(5):412-416.

146. Calderon-Osuna E, Vinuesa M, Fernandez-Machin P, Mendoza E, Gallardo JA, Pineda JA: [A cystic lymphoepithelial lesion of the parotid in HIV-1-infected patients]. Medicina clinica 1995, 105(12):461-463.

147. Cami J, Domingo-Salvany A: [The risk factors in death from heroin]. Medicina clinica 1995, 105(12):455-456.

148. Campbell JV, Garfein RS, Thiede H, Hagan H, Ouellet LJ, Golub ET, Hudson SM, Ompad DC, Weinbaum C: Convenience is the key to hepatitis A and B vaccination uptake among young adult injection drug users. Drug Alcohol Depend 2007, 91 Suppl 1:S64-72.

149. Campbell JV, Hagan H, Latka MH, Garfein RS, Golub ET, Coady MH, Thomas DL, Strathdee SA: High prevalence of alcohol use among hepatitis C virus antibody positive injection drug users in three US cities. Drug Alcohol Depend 2006, 81(3):259-265.

150. Campos LN, Guimaraes MD, Carmo RA, Melo AP, Oliveira HN, Elkington K, McKinnon K: HIV, syphilis, and hepatitis B and C prevalence among patients with mental illness: a review of the literature. Cadernos de saude publica 2008, 24 Suppl 4:s607-620.

151. Caraceni P, Domenicali M, Giannone F, Bernardi M: The role of the endocannabinoid system in liver diseases. Best practice & research Clinical endocrinology & metabolism 2009, 23(1):65-77.

152. Carcaba V, Carton JA: [HIV infection and tuberculosis]. Anales de medicina interna (Madrid, Spain : 1984) 1991, 8(8):401-410.

153. Carpenter JL, Huang DY: Community-acquired pulmonary infections in a public municipal hospital in the 1980s. South Med J 1991, 84(3):299-306.

154. Carvalho AC, Migliori GB, Cirillo DM: Tuberculosis in Europe: a problem of drug resistance or much more? Expert review of respiratory medicine 2010, 4(2):189-200.

155. Casoli C, Pilotti E, Bertazzoni U: Molecular and cellular interactions of HIV-1/HTLV coinfection and impact on AIDS progression. AIDS reviews 2007, 9(3):140-149.

156. Castro KG, Snider DE, Jr.: The good news and the bad news about multidrug-resistant tuberculosis. Clin Infect Dis 1995, 21(5):1265-1266.

157. Castro Lde P: [Gastritis: a new vision]. Acta gastroenterologica Latinoamericana 1994, 24(3):175-193.

158. Causse X, Gargot D, Michenet P: [Liver steatosis. II: Microvesicular steatosis]. Gastroenterol Clin Biol 1995, 19(1):66-75.

159. Cayol V, Corcos M, Clervoy P, Speranza M: [Pregnancy and drug abuse: current situation and therapeutic strategies]. Ann Med Interne (Paris) 2000, 151 Suppl B:B20-26.

160. Chaisson RE, Barnes GL, Hackman J, Watkinson L, Kimbrough L, Metha S, Cavalcante S, Moore RD: A randomized, controlled trial of interventions to improve adherence to isoniazid therapy to prevent tuberculosis in injection drug users. The American journal of medicine 2001, 110(8):610-615.

161. Chak E, Saab S: Risk factors and incidence of de novo malignancy in liver transplant recipients: a systematic review. Liver Int 2010, 30(9):1247-1258.

162. Chak EW, Sarkar S, Bowlus C: Improving Healthcare Systems to Reduce Healthcare Disparities in Viral Hepatitis. Dig Dis Sci 2016, 61(10):2776-2783.

163. Chan C, Levitsky J: Infection and Alcoholic Liver Disease. Clinics in liver disease 2016, 20(3):595-606.

164. Chang SL, Connaghan KP: Behavioral and molecular evidence for a feedback interaction between morphine and HIV-1 viral proteins. J Neuroimmune Pharmacol 2012, 7(2):332-340.

165. Chang YW: [Non-Helicobacter pylori, Non-nonsteroidal Anti-inflammatory Drug Peptic Ulcer Disease]. The Korean journal of gastroenterology = Taehan Sohwagi Hakhoe chi 2016, 67(6):313-317.

166. Chen CY, Lin KM: Health consequences of illegal drug use. Curr Opin Psychiatry 2009, 22(3):287-292.

167. Chiba T, Tanaka N: [Influence of heavy drinking for the development of hepatocellular carcinoma in type C cirrhosis]. Nihon rinsho Japanese journal of clinical medicine 1995, 53 Suppl(Pt 1):731-735.

168. Chitwood DD, Comerford M, Trapido EJ: Strategies for enhancing existing studies of the natural history of HIV-1 infection among drug users. NIDA research monograph 1991, 109:9-28.

169. Christensen PB, Fisker N, Krarup HB, Liebert E, Jaroslavtsev N, Christensen K, Georgsen J: Hepatitis B vaccination in prison with a 3-week schedule is more efficient than the standard 6-month schedule. Vaccine 2004, 22(29-30):3897-3901.

170. Chu C, Selwyn PA: Current health disparities in HIV/AIDS. AIDS Read 2008, 18(3):144-146, 152-148, c143.

171. Chu TX, Levy JA: Injection drug use and HIV/AIDS transmission in China. Cell research 2005, 15(11-12):865-869.

172. Chung H, Ueda T, Kudo M: Changing trends in hepatitis C infection over the past 50 years in Japan. Intervirology 2010, 53(1):39-43.

173. Chung RT: Acute hepatitis C virus infection. Clin Infect Dis 2005, 41 Suppl 1:S14-17.

174. Clarke SM, Mulcahy FM: Antiretroviral therapy for drug users. Int J STD AIDS 2000, 11(10):627-631.

175. Clouston AD, Jonsson JR, Powell EE: Steatosis as a cofactor in other liver diseases: hepatitis C virus, alcohol, hemochromatosis, and others. Clinics in liver disease 2007, 11(1):173-189, x.

176. Clouston AD, Powell EE: Interaction of non-alcoholic fatty liver disease with other liver diseases. Best practice & research Clinical gastroenterology 2002, 16(5):767-781.

177. Coats JT, Dillon JF: The effect of introducing point-of-care or dried blood spot analysis on the uptake of hepatitis C virus testing in high-risk populations: A systematic review of the literature. The International journal on drug policy 2015, 26(11):1050-1055.

178. Coffin PO, Latka MH, Latkin C, Wu Y, Purcell DW, Metsch L, Gomez C, Gourevitch MN: Safe syringe disposal is related to safe syringe access among HIV-positive injection drug users. Aids Behav 2007, 11(5):652-662.

179. Coffin PO, Rowe C, Santos GM: Novel interventions to prevent HIV and HCV among persons who inject drugs. Curr HIV/AIDS Rep 2015, 12(1):145-163.

180. Cohen MS, Smith MK, Muessig KE, Hallett TB, Powers KA, Kashuba AD: Antiretroviral treatment of HIV-1 prevents transmission of HIV-1: where do we go from here? Lancet 2013, 382(9903):1515-1524.

181. Cohn JA: HIV-1 infection in injection drug users. Infectious disease clinics of North America 2002, 16(3):745-770.

182. Connery H, Greenfield S, Livchits V, McGrady L, Patrick N, Lastimoso CS, Heney JH, Nelson AK, Shields A, Stepanova YP et al: Training and fidelity monitoring of alcohol treatment interventions integrated into routine tuberculosis care in Tomsk, Russia: the IMPACT Effectiveness Trial. Subst Use Misuse 2013, 48(9):784-792.

183. Conway B, Grebely J, Tossonian H, Lefebvre D, de Vlaming S: A systematic approach to the treatment of HIV and hepatitis C virus infection in the inner city: a Canadian perspective. Clin Infect Dis 2005, 41 Suppl 1:S73-78.

184. Cook JA: Associations between use of crack cocaine and HIV-1 disease progression: research findings and implications for mother-to-infant transmission. Life Sci 2011, 88(21-22):931-939.

185. Cooksley W: Chronic liver disease: do alcohol and hepatitis C virus interact. J Gastroenterol Hepatol 1996, 11(2):187-192.

186. Cooper CL: Obstacles to successful HCV treatment in substance addicted patients. J Addict Dis 2008, 27(2):61-68.

187. Coppo M, Borghi A: [Epistemology of liver cirrhosis]. Medicina (Florence, Italy) 1990, 10(2):99-107.

188. Corridori S, Trespi G, Baldini E, Campisi D, Renzetti I, Calderon W: [Immunohepatologic and clinical monitoring of neuramide therapy in acute viral hepatitis B]. Bollettino dell'Istituto sieroterapico milanese 1985, 64(1):42-51.

189. Corsi KF, Booth RE: HIV sex risk behaviors among heterosexual methamphetamine users: literature review from 2000 to present. Current drug abuse reviews 2008, 1(3):292-296.

190. Corson S, Greenhalgh D, Palmateer N, Weir A, Hutchinson S: Risk of Hepatitis C virus re-infection following spontaneous viral clearance in injecting drug users: a systematic review. The International journal on drug policy 2011, 22(2):102-108.

191. Cossart YE: Epidemiology of serum hepatitis. British medical bulletin 1972, 28(2):156-162.

192. Cotrim HP, Garrido V, Parana R, Santana G, Dultra D, Pinto M, Lyra L: [Paracentesis associated to dextran-70 in the treatment of ascites in patients with chronic liver diseases: a randomized therapeutic study]. Arq Gastroenterol 1994, 31(4):125-129.

193. Couzigou P, Mathurin P, Serfaty L, Cacoub P, Moussalli J, Pialoux G, Chossegros P, Cattan L, Pol S: [Alcohol, steatohepatitis, insulin resistance and hepatitis C]. Gastroenterol Clin Biol 2008, 32(3 Pt 2):S74-81.

194. Cox AL, Thomas DL: Hepatitis C virus vaccines among people who inject drugs. Clin Infect Dis 2013, 57 Suppl 2:S46-50.

195. Crawford S, Bath N: Peer support models for people with a history of injecting drug use undertaking assessment and treatment for hepatitis C virus infection. Clin Infect Dis 2013, 57 Suppl 2:S75-79.

196. Crofts N: Going where the epidemic is. Epidemiology and control of hepatitis C among injecting drug users. Aust Fam Physician 2001, 30(5):420-425.

197. Crofts N, Jolley D, Kaldor J, van Beek I, Wodak A: Epidemiology of hepatitis C virus infection among injecting drug users in Australia. Journal of epidemiology and community health 1997, 51(6):692-697.

198. Crone CC, Gabriel GM, Wise TN: Managing the neuropsychiatric side effects of interferon-based therapy for hepatitis C. Cleve Clin J Med 2004, 71 Suppl 3:S27-32.

199. Culbert GJ, Pillai V, Bick J, Al-Darraji HA, Wickersham JA, Wegman MP, Bazazi AR, Ferro E, Copenhaver M, Kamarulzaman A et al: Confronting the HIV, Tuberculosis, Addiction, and Incarceration Syndemic in Southeast Asia: Lessons Learned from Malaysia. J Neuroimmune Pharmacol 2016, 11(3):446-455.

200. Cullen W, Stanley J, Langton D, Kelly Y, Staines A, Bury G: Hepatitis C infection among injecting drug users in general practice: a cluster randomised controlled trial of clinical guidelines' implementation. Br J Gen Pract 2006, 56(532):848-856.

201. Cunha HM, Pacheco MH, Rodrigues A, Cardoso J, Ferreira FP, Malhado JA: [Syphilis in an HIV-infected patient]. Acta medica portuguesa 1997, 10(6-7):503-507.

202. Cunningham EB, Applegate TL, Lloyd AR, Dore GJ, Grebely J: Mixed HCV infection and reinfection in people who inject drugs--impact on therapy. Nature reviews Gastroenterology & hepatology 2015, 12(4):218-230.

203. da Costa e Silva VL: [Health consequences of the tobacco epidemic in West African French-speaking countries and current tobacco control]. Promotion & education 2005, Suppl 4:7-12, 54.

204. da Silva LJ, Resende MR, de Abreu WB, Aoki FH, Bocatto RB, Branchinni ML, Goncales Junior FL, de Lima JN, von Nowakonski A, Papaiordanou PM et al: Listeriosis and AIDS: case report and literature review. Revista do Instituto de Medicina Tropical de Sao Paulo 1992, 34(5):475-478.

205. Dalinka MK, Lally JF, Koniver G, Coren GS: The radiology of osseous and articular infection. CRC critical reviews in clinical radiology and nuclear medicine 1975, 7(1):1-64.

206. Dan C, Moses-Eisenstein M, Valdiserri RO: CE: Viral Hepatitis: New U.S. Screening Recommendations, Assessment Tools, and Treatments. The American journal of nursing 2015, 115(7):26-35; quiz 36, 48.

207. Davis SM, Daily S, Kristjansson AL, Kelley GA, Zullig K, Baus A, Davidov D, Fisher M: Needle exchange programs for the prevention of hepatitis C virus infection in people who inject drugs: a systematic review with meta-analysis. Harm Reduct J 2017, 14(1):25.

208. Day CA, Shanahan M, Wand H, Topp L, Haber PS, Rodgers C, Deacon R, Walsh N, Kaldor J, van Beek I et al: Development of immunity following financial incentives for hepatitis B vaccination among people who inject drugs: A randomized controlled trial. J Clin Virol 2016, 74:66-72.

209. De Bie J, Robaeys G, Buntinx F: Hepatitis C, interferon alpha and psychiatric co-morbidity in intravenous drug users (IVDU) : guidelines for clinical practice. Acta gastro-enterologica Belgica 2005, 68(1):68-80.

210. De Irala J, Bigelow C, McCusker J, Hindin R, Zheng L: Reliability of self-reported human immunodeficiency virus risk behaviors in a residential drug treatment population. Am J Epidemiol 1996, 143(7):725-732.

211. de Ledinghen V: [Natural history of hepatitis C virus infection]. Gastroenterol Clin Biol 2002, 26 Spec No 2:B9-22.

212. De March Ayuela P, Turell Guma J: [Results of 2 or 3 secondary drug combinations in the retreatment of chronic pulmonary tuberculosis]. Revista clinica espanola 1968, 109(2):117-126.

213. de Marie S: [Infectious complications of drug addiction]. Ned Tijdschr Geneeskd 1995, 139(50):2618-2622.

214. De P, Cox J, Boivin JF, Platt RW, Jolly AM: The importance of social networks in their association to drug equipment sharing among injection drug users: a review. Addiction 2007, 102(11):1730-1739.

215. De P, Roy E, Boivin JF, Cox J, Morissette C: Risk of hepatitis C virus transmission through drug preparation equipment: a systematic and methodological review. J Viral Hepat 2008, 15(4):279-292.

216. DeBeck K, Cheng T, Montaner JS, Beyrer C, Elliott R, Sherman S, Wood E, Baral S: HIV and the criminalisation of drug use among people who inject drugs: a systematic review. The lancet HIV 2017, 4(8):e357-e374.

217. DeFulio A, Silverman K: The use of incentives to reinforce medication adherence. Preventive medicine 2012, 55 Suppl:S86-94.

218. Degenhardt L, Peacock A, Colledge S, Leung J, Grebely J, Vickerman P, Stone J, Cunningham EB, Trickey A, Dumchev K et al: Global prevalence of injecting drug use and sociodemographic characteristics and prevalence of HIV, HBV, and HCV in people who inject drugs: a multistage systematic review. The Lancet Global health 2017, 5(12):e1192-e1207.

219. Degos F: [Hepatitis B virus vaccine. Evaluation and perspectives]. Gastroenterol Clin Biol 1992, 16(12):927-931.

220. Degos F: Natural history of hepatitis C virus infection. Nephrology, dialysis, transplantation : official publication of the European Dialysis and Transplant Association - European Renal Association 1996, 11 Suppl 4:16-18.

221. Degos F: Hepatitis C and alcohol. J Hepatol 1999, 31 Suppl 1:113-118.

222. Deiss RG, Rodwell TC, Garfein RS: Tuberculosis and illicit drug use: review and update. Clin Infect Dis 2009, 48(1):72-82.

223. Delarocque-Astagneau E, Pillonel J, de Valk H, Perra A, Laperche S, Desenclos JC: [Modes of hepatitis C virus transmission: methodological approaches]. Rev Epidemiol Sante Publique 2006, 54 Spec No 1:1s5-1s14.

224. Delwaide J, Bourgeois N, Colle I, Robaeys G: Risk factors for hepatitis C: past, present and future. Acta gastro-enterologica Belgica 2002, 65(2):87-89.

225. Delwaide J, Gerard C, Sondag D, Belaiche J: [Methods of hepatitis C virus transmission]. Revue medicale de Liege 1997, 52(6):388-391.

226. Des Jarlais DC, Arasteh K, McKnight C, Perlman DC, Feelemyer J, Hagan H, Cooper HL: HSV-2 co-infection as a driver of HIV transmission among heterosexual non-injecting drug users in New York City. PLoS One 2014, 9(1):e87993.

227. Des Jarlais DC, Feelemyer JP, Modi SN, Abdul-Quader A, Hagan H: High coverage needle/syringe programs for people who inject drugs in low and middle income countries: a systematic review. BMC Public Health 2013, 13:53.

228. Des Jarlais DC, Friedman SR: HIV infection among intravenous drug users: epidemiology and risk reduction. Aids 1987, 1(2):67-76.

229. Dettmeyer RB, Preuss J, Wollersen H, Madea B: Heroin-associated nephropathy. Expert opinion on drug safety 2005, 4(1):19-28.

230. Deugnier Y, Moirand R, Guyader D, Jouanolle AM, Brissot P: [Iron overload and HFE gene]. Gastroenterol Clin Biol 1999, 23(1):122-131.

231. Dhumeaux D: [Hepatitis C in France]. Gastroenterol Clin Biol 2002, 26 Spec No 2:B133-137.

232. Di Gennaro F, Pizzol D, Cebola B, Stubbs B, Monno L, Saracino A, Luchini C, Solmi M, Segafredo G, Putoto G et al: Social determinants of therapy failure and multi drug resistance among people with tuberculosis: A review. Tuberculosis (Edinburgh, Scotland) 2017, 103:44-51.

233. Diamantis I, Bassetti S, Erb P, Ladewig D, Gyr K, Battegay M: High prevalence and coinfection rate of hepatitis G and C infections in intravenous drug addicts. J Hepatol 1997, 26(4):794-797.

234. Diaz Curiel M, Fernandez Guerrero ML, Suarez Fernandez C: [Infections in liver cirrhosis]. Revista clinica espanola 1984, 172(5):241-250.

235. Dienstag JL, Alter HJ: Non-A, non-B hepatitis: evolving epidemiologic and clinical perspective. Seminars in liver disease 1986, 6(1):67-81.

236. Dieperink E, Fuller B, Isenhart C, McMaken K, Lenox R, Pocha C, Thuras P, Hauser P: Efficacy of motivational enhancement therapy on alcohol use disorders in patients with chronic hepatitis C: a randomized controlled trial. Addiction 2014, 109(11):1869-1877.

237. DiMartini A, Crone C, Dew MA: Alcohol and substance use in liver transplant patients. Clinics in liver disease 2011, 15(4):727-751.

238. Dimitroulopoulos D, Petroulaki E, Manolakopoulos S, Anagnostou O, Tsaklakidou D, Xinopoulos D, Tsamakidis K, Tzourmakliotis D, Paraskevas E: Peginterferon/ribavirin treatment achieves a higher compliance rate than interferon/ribavirin combination in patients chronically infected with HCV on methadone maintenance. Eur J Gastroenterol Hepatol 2009, 21(12):1407-1412.

239. Dimova RB, Zeremski M, Jacobson IM, Hagan H, Des Jarlais DC, Talal AH: Determinants of hepatitis C virus treatment completion and efficacy in drug users assessed by meta-analysis. Clin Infect Dis 2013, 56(6):806-816.

240. Doffoel M: [Obesity and alcoholic and viral chronic liver disease]. Gastroenterol Clin Biol 2004, 28(3):268-271.

241. Doffoel M, Gabanyi J, Vetter D: [Etiological treatment of cirrhosis in adults]. La Revue du praticien 1997, 47(5):503-506.

242. Dolan K, Rutter S, Wodak AD: Prison-based syringe exchange programmes: a review of international research and development. Addiction 2003, 98(2):153-158.

243. Dolan K, Wirtz AL, Moazen B, Ndeffo-Mbah M, Galvani A, Kinner SA, Courtney R, McKee M, Amon JJ, Maher L et al: Global burden of HIV, viral hepatitis, and tuberculosis in prisoners and detainees. Lancet 2016, 388(10049):1089-1102.

244. Dolan KA, Shearer J, White B, Zhou J, Kaldor J, Wodak AD: Four-year follow-up of imprisoned male heroin users and methadone treatment: mortality, re-incarceration and hepatitis C infection. Addiction 2005, 100(6):820-828.

245. Dolganiuc A: Alcohol and Viral Hepatitis: Role of Lipid Rafts. Alcohol research : current reviews 2015, 37(2):299-309.

246. Donahoe RM, Byrd LD, McClure HM, Fultz P, Brantley M, Marsteller F, Ansari AA, Wenzel D, Aceto M: Consequences of opiate-dependency in a monkey model of AIDS. Advances in experimental medicine and biology 1993, 335:21-28.

247. Donahoe RM, Falek A: Neuroimmunomodulation by opiates and other drugs of abuse: relationship to HIV infection and AIDS. Advances in biochemical psychopharmacology 1988, 44:145-158.

248. Donahoe RM, Vlahov D: Opiates as potential cofactors in progression of HIV-1 infections to AIDS. Journal of neuroimmunology 1998, 83(1-2):77-87.

249. Dong HV, Cortes YI, Shiau S, Yin MT: Osteoporosis and fractures in HIV/hepatitis C virus coinfection: a systematic review and meta-analysis. Aids 2014, 28(14):2119-2131.

250. Dongiovanni P, Donati B, Fares R, Lombardi R, Mancina RM, Romeo S, Valenti L: PNPLA3 I148M polymorphism and progressive liver disease. World J Gastroenterol 2013, 19(41):6969-6978.

251. Dore GJ, Altice F, Litwin AH, Dalgard O, Gane EJ, Shibolet O, Luetkemeyer A, Nahass R, Peng CY, Conway B et al: Elbasvir-Grazoprevir to Treat Hepatitis C Virus Infection in Persons Receiving Opioid Agonist Therapy: A Randomized Trial. Ann Intern Med 2016, 165(9):625-634.

252. Dore GJ, Law M, MacDonald M, Kaldor JM: Epidemiology of hepatitis C virus infection in Australia. J Clin Virol 2003, 26(2):171-184.

253. Dore GJ, Thomas DL: Management and treatment of injection drug users with hepatitis C virus (HCV) infection and HCV/human immunodeficiency virus coinfection. Seminars in liver disease 2005, 25(1):18-32.

254. Doyle JS, Aspinall EJ, Hutchinson SJ, Quinn B, Gore C, Wiktor SZ, Hellard ME: Global policy and access to new hepatitis C therapies for people who inject drugs. The International journal on drug policy 2015, 26(11):1064-1071.

255. Drumright LN, Hagan H, Thomas DL, Latka MH, Golub ET, Garfein RS, Clapp JD, Campbell JV, Bonner S, Kapadia F et al: Predictors and effects of alcohol use on liver function among young HCV-infected injection drug users in a behavioral intervention. J Hepatol 2011, 55(1):45-52.

256. Duesberg P: Infectious AIDS--stretching the germ theory beyond its limits. International archives of allergy and immunology 1994, 103(2):118-127.

257. Duesberg P, Rasnick D: The AIDS dilemma: drug diseases blamed on a passenger virus. Genetica 1998, 104(2):85-132.

258. Duesberg PH: AIDS: non-infectious deficiencies acquired by drug consumption and other risk factors. Research in immunology 1990, 141(1):5-11.

259. Dunn KE, Saulsgiver KA, Patrick ME, Heil SH, Higgins ST, Sigmon SC: Characterizing and improving HIV and hepatitis knowledge among primary prescription opioid abusers. Drug Alcohol Depend 2013, 133(2):625-632.

260. Dutkowski P, Linecker M, DeOliveira ML, Mullhaupt B, Clavien PA: Challenges to liver transplantation and strategies to improve outcomes. Gastroenterology 2015, 148(2):307-323.

261. Dutta R, Roy S: Mechanism(s) involved in opioid drug abuse modulation of HAND. Curr HIV Res 2012, 10(5):469-477.

262. Ebeling F: Importance of hepatitis C virus infection in Europe and North America. Current studies in hematology and blood transfusion 1994(61):164-181.

263. Ebeling F: Epidemiology of the hepatitis C virus. Vox sanguinis 1998, 74 Suppl 2:143-146.

264. Ebner N, Wanner C, Winklbaur B, Matzenauer C, Jachmann CA, Thau K, Fischer G: Retention rate and side effects in a prospective trial on hepatitis C treatment with pegylated interferon alpha-2a and ribavirin in opioid-dependent patients. Addiction biology 2009, 14(2):227-237.

265. Echeverria MJ, Lopez de Goicoechea MJ, Ayarza R, Berdonces P, Audicana J, Fernandez R, Soriano F: [Meningitis caused by Streptococcus mitis in a cocaine addict]. Enferm Infecc Microbiol Clin 1998, 16(9):441-442.

266. Eddleston AL, Vento S: Relevance of immune mediated mechanisms in progressive alcoholic liver injury. Molecular aspects of medicine 1988, 10(2):169-177.

267. Edlin BR: Prevention and treatment of hepatitis C in injection drug users. Hepatology 2002, 36(5 Suppl 1):S210-219.

268. Edlin BR, Kresina TF, Raymond DB, Carden MR, Gourevitch MN, Rich JD, Cheever LW, Cargill VA: Overcoming barriers to prevention, care, and treatment of hepatitis C in illicit drug users. Clin Infect Dis 2005, 40 Suppl 5:S276-285.

269. El-Bassel N, Gilbert L, Terlikbayeva A, Beyrer C, Wu E, Chang M, Hunt T, Ismayilova L, Shaw SA, Primbetova S et al: Effects of a couple-based intervention to reduce risks for HIV, HCV, and STIs among drug-involved heterosexual couples in Kazakhstan: a randomized controlled trial. J Acquir Immune Defic Syndr 2014, 67(2):196-203.

270. El-Bassel N, Wechsberg WM, Shaw SA: Dual HIV risk and vulnerabilities among women who use or inject drugs: no single prevention strategy is the answer. Curr Opin HIV AIDS 2012, 7(4):326-331.

271. El-Ghitany EM, Abdel Wahab MM, Abd El-Wahab EW, Hassouna S, Farghaly AG: A comprehensive hepatitis C virus risk factors meta-analysis (1989-2013): do they differ in Egypt? Liver Int 2015, 35(2):489-501.

272. Elliott JC, Stohl M, Aharonovich E, O'Leary A, Hasin DS: Reasons for drinking as predictors of alcohol involvement one year later among HIV-infected individuals with and without hepatitis C. Annals of medicine 2016, 48(8):634-640.

273. Elpek GO: Cellular and molecular mechanisms in the pathogenesis of liver fibrosis: An update. World J Gastroenterol 2014, 20(23):7260-7276.

274. El-Serag HB: Hepatocellular carcinoma: an epidemiologic view. J Clin Gastroenterol 2002, 35(5 Suppl 2):S72-78.

275. Engbaek HC, Larsen SO, Rasmussen KN, Vergmann B: [Treatment of tuberculosis with streptomycin and isoniazide combined with either aminosalyl or rifampicin]. Ugeskrift for laeger 1973, 135(34):1795-1799.

276. Engelich G, Wright DG, Hartshorn KL: Acquired disorders of phagocyte function complicating medical and surgical illnesses. Clin Infect Dis 2001, 33(12):2040-2048.

277. Erdozain JC, Lizasoain J, Martin-de-Argila C, Presa M, Munoz F: [Cytomegalovirus colitis in a patient carrying the human immunodeficiency virus: the endoscopic image similar to pseudomembranous colitis]. Revista espanola de enfermedades digestivas : organo oficial de la Sociedad Espanola de Patologia Digestiva 1995, 87(3):247-249.

278. Esmaeili A, Mirzazadeh A, Carter GM, Esmaeili A, Hajarizadeh B, Sacks HS, Page KA: Higher incidence of HCV in females compared to males who inject drugs: A systematic review and meta-analysis. J Viral Hepat 2017, 24(2):117-127.

279. Esteban JI, Sauleda S, Quer J: The changing epidemiology of hepatitis C virus infection in Europe. J Hepatol 2008, 48(1):148-162.

280. Estebanez-Munoz M, Soto-Abanades CI, Rios-Blanco JJ, Arribas JR: Updating our understanding of pulmonary disease associated with HIV infection. Archivos de bronconeumologia 2012, 48(4):126-132.

281. Estrada AL: Epidemiology of HIV/AIDS, hepatitis B, hepatitis C, and tuberculosis among minority injection drug users. Public health reports (Washington, DC : 1974) 2002, 117 Suppl 1:S126-134.

282. Estrada AL: Health disparities among African-American and Hispanic drug injectors--HIV, AIDS, hepatitis B virus and hepatitis C virus: a review. Aids 2005, 19 Suppl 3:S47-52.

283. Everall IP: Interaction between HIV and intravenous heroin abuse? Journal of neuroimmunology 2004, 147(1-2):13-15.

284. Evon DM, Simpson K, Kixmiller S, Galanko J, Dougherty K, Golin C, Fried MW: A randomized controlled trial of an integrated care intervention to increase eligibility for chronic hepatitis C treatment. The American journal of gastroenterology 2011, 106(10):1777-1786.

285. Falade-Nwulia O, Sulkowski MS, Merkow A, Latkin C, Mehta SH: Understanding and addressing hepatitis C reinfection in the oral direct-acting antiviral era. J Viral Hepat 2018, 25(3):220-227.

286. Farazi PA, DePinho RA: The genetic and environmental basis of hepatocellular carcinoma. Discovery medicine 2006, 6(35):182-186.

287. Farber E: Alcohol and other chemicals in the development of hepatocellular carcinoma. Clinics in laboratory medicine 1996, 16(2):377-394.

288. Farkkila M: [The treatment of hepatitis C]. Duodecim; laaketieteellinen aikakauskirja 1997, 113(11):1061-1068.

289. Farrar DJ, Flanigan TP, Gordon NM, Gold RL, Rich JD: Tuberculous brain abscess in a patient with HIV infection: case report and review. The American journal of medicine 1997, 102(3):297-301.

290. Fecht WJ, Jr., Befeler AS: Hepatocellular carcinoma: updates in primary prevention. Current gastroenterology reports 2004, 6(1):37-43.

291. Federico A, Dallio M, Ormando VM, Abenavoli L, Masarone M, Persico M, Loguercio C: Alcoholic Liver Disease and Hepatitis C Chronic Infection. Reviews on recent clinical trials 2016, 11(3):201-207.

292. Feher J, Lengyel G: [Silymarin in the treatment of chronic liver diseases: past and future]. Orvosi hetilap 2008, 149(51):2413-2418.

293. Feher J, Lengyel G: [Hepatocellular carcinoma: occurrence, risk factors, biomarkers]. Orvosi hetilap 2010, 151(23):933-940.

294. Feng Y, Shi J, Gao L, Yao T, Feng D, Luo D, Li Z, Zhang Y, Wang F, Cui F et al: Immunogenicity and safety of high-dose hepatitis B vaccine among drug users: A randomized, open-labeled, blank-controlled trial. Human vaccines & immunotherapeutics 2017, 13(6):1-7.

295. Fernandes RM, Cary M, Duarte G, Jesus G, Alarcao J, Torre C, Costa S, Costa J, Carneiro AV: Effectiveness of needle and syringe Programmes in people who inject drugs - An overview of systematic reviews. BMC Public Health 2017, 17(1):309.

296. Fernandez Sierra MA, Gomez Olmedo M, Delgado Rodriguez M, Galvez Vargas R: [Infection by the human immunodeficiency virus in the Spanish population (II). A meta-analysis of the time and geographic trends]. Medicina clinica 1990, 95(10):366-371.

297. Ferrell L: Liver pathology: cirrhosis, hepatitis, and primary liver tumors. Update and diagnostic problems. Modern pathology : an official journal of the United States and Canadian Academy of Pathology, Inc 2000, 13(6):679-704.

298. Ferris MJ, Mactutus CF, Booze RM: Neurotoxic profiles of HIV, psychostimulant drugs of abuse, and their concerted effect on the brain: current status of dopamine system vulnerability in NeuroAIDS. Neuroscience and biobehavioral reviews 2008, 32(5):883-909.

299. Festa L, Meucci O: Effects of opiates and HIV proteins on neurons: the role of ferritin heavy chain and a potential for synergism. Curr HIV Res 2012, 10(5):453-462.

300. Filoche B: [Treatment of hepatitis C in intravenous drug users]. Gastroenterol Clin Biol 2002, 26 Spec No 2:B252-256.

301. Fiorencis R, Zonzin P, Carraro M, Zampieri P, Roncon L, Baracca E, Masiero G, Bilato C: Pulmonary hypertension associated with human immunodeficiency virus infection. Report of two cases and review of the literature. Giornale italiano di cardiologia 1998, 28(12):1404-1408.

302. Firooznia H, Golimbu C, Rafii M, Lichtman EA: Radiology of musculoskeletal complications of drug addiction. Seminars in roentgenology 1983, 18(3):198-206.

303. Fischer B, Rehm J, Kim G, Robins A: Safer injection facilities (SIFs) for injection drug users (IDUs) in Canada. A review and call for an evidence-focused pilot trial. Canadian journal of public health = Revue canadienne de sante publique 2002, 93(5):336-338.

304. Fischer B, Reimer J, Firestone M, Kalousek K, Rehm J, Heathcote J: Treatment for hepatitis C virus and cannabis use in illicit drug user patients: implications and questions. Eur J Gastroenterol Hepatol 2006, 18(10):1039-1042.

305. Fisher DG, Cagle HH, Davis DC, Fenaughty AM, Kuhrt-Hunstiger T, Fison SR: Health consequences of rural illicit drug use: questions without answers. NIDA research monograph 1997, 168:175-195.

306. FitzGerald JM, Wang L, Elwood RK: Tuberculosis: 13. Control of the disease among aboriginal people in Canada. CMAJ : Canadian Medical Association journal = journal de l'Association medicale canadienne 2000, 162(3):351-355.

307. Flaker V: Heroin use in Slovenia - a consequence or a vehicle of social changes. Eur Addict Res 2002, 8(4):170-176.

308. Floreani A: Liver diseases in the elderly: an update. Digestive diseases (Basel, Switzerland) 2007, 25(2):138-143.

309. Fortuno-Mar A, Mayayo E, Castillo A, Guiral H: [A lymphoepithelial cyst of the parotid gland in an advanced stage of HIV infection. A rare association]. Anales otorrinolaringologicos ibero-americanos 1999, 26(5):469-475.

310. Freedman K, Nathanson J: Interferon-based hepatitis C treatment in patients with pre-existing severe mental illness and substance use disorders. Expert review of anti-infective therapy 2009, 7(3):363-376.

311. French SW: Ethanol and hepatocellular injury. Clinics in laboratory medicine 1996, 16(2):289-306.

312. Friedman SR, Des Jarlais DC: HIV among drug injectors: the epidemic and the response. AIDS Care 1991, 3(3):239-250.

313. Friend PJ, Imber CJ: Current status of liver transplantation. Methods in molecular biology (Clifton, NJ) 2006, 333:29-46.

314. Frimpong JA, D'Aunno T, Perlman DC, Strauss SM, Mallow A, Hernandez D, Schackman BR, Feaster DJ, Metsch LR: On-site bundled rapid HIV/HCV testing in substance use disorder treatment programs: study protocol for a hybrid design randomized controlled trial. Trials 2016, 17(1):117.

315. Fujimura RK, Shapshak P, Segal DM, Crandall KA, Goodkin K, Page JB, Douyon R, Zhang BT, Xin KQ, Rodriguez de la Vega P et al: Viral and host determinants of neurovirulence of HIV-1 infection. Advances in experimental medicine and biology 1998, 437:241-253.

316. Fuller BE, Loftis JM, Rodriguez VL, McQuesten MJ, Hauser P: Psychiatric and substance use disorders comorbidities in veterans with hepatitis C virus and HIV coinfection. Curr Opin Psychiatry 2009, 22(4):401-408.

317. Fuster D, Samet JH: Alcohol Use in Patients with Chronic Liver Disease. N Engl J Med 2018, 379(13):1251-1261.

318. Fuster D, Tor J, Rey-Joly C, Muga R: [Pathogenic interactions between alcohol and hepatitis C]. Medicina clinica 2012, 138(14):627-632.

319. Gagnon H, Godin G, Alary M, Bruneau J, Otis J: A randomized trial to evaluate the efficacy of a computer-tailored intervention to promote safer injection practices among drug users. Aids Behav 2010, 14(3):538-548.

320. Gailhouste L, Gomez-Santos L, Ochiya T: Potential applications of miRNAs as diagnostic and prognostic markers in liver cancer. Frontiers in bioscience (Landmark edition) 2013, 18:199-223.

321. Galiana Gomez del Pulgar J, Marchan Carranza E, Gijon Rodriguez J, Sanz Gil MJ: [Primary psoas abscess complicated by septic arthritis and kidney failure]. Revista clinica espanola 1994, 194(4):320-321.

322. Gallant C, Kenny P: Oral glucocorticoids and their complications. A review. Journal of the American Academy of Dermatology 1986, 14(2 Pt 1):161-177.

323. Gallegos-Orozco JF, Vargas HE: Liver transplantation: from Child to MELD. The Medical clinics of North America 2009, 93(4):931-950, ix.

324. Galli C, Suligoi B, Spuntarelli G, Ceccanti M, Nardi E, Ginanni-Corradini S: HBV infection in chronic alcoholics. A survey on 287 cases. Bollettino dell'Istituto sieroterapico milanese 1988, 67(1):36-42.

325. Gambato M, Villanueva A, Abraldes JG, Altamirano J, Forns X: Clinical Trial Watch: Reports from the EASL International Liver Congress (ILC), Vienna, April 2015. J Hepatol 2015, 63(3):753-762.

326. Gannon P, Khan MZ, Kolson DL: Current understanding of HIV-associated neurocognitive disorders pathogenesis. Current opinion in neurology 2011, 24(3):275-283.

327. Gao B: Interaction of alcohol and hepatitis viral proteins: implication in synergistic effect of alcohol drinking and viral hepatitis on liver injury. Alcohol (Fayetteville, NY) 2002, 27(1):69-72.

328. Garces PA, Tena EV: [Etravirine in first-line therapy]. Enferm Infecc Microbiol Clin 2009, 27 Suppl 2:12-20.

329. Garcia Fernandez D, Garcia-Patos Briones V, Mollet Sanchez J, Castells Rodellas A: [Stevens-Johnson syndrome due to nevirapine]. Revista clinica espanola 2000, 200(3):179-180.

330. Garcia Rio F, Garcia Satue JL, Prados C, Casadevall J, Gomez L, Pino JM: [3 nonidiopathic forms of bronchiolitis obliterans organizing pneumonia]. Archivos de bronconeumologia 1994, 30(5):263-265.

331. Garcia-Compean D, Jaquez-Quintana JO, Maldonado-Garza H: Hepatogenous diabetes. Current views of an ancient problem. Annals of hepatology 2009, 8(1):13-20.

332. Garcia-Escano MD, Andrade RJ, Gonzalez-Santos P: [Interferon and plasma lipids]. Medicina clinica 2000, 114(2):74-76.

333. Garcia-Patos Briones V, Rodriguez Cano L, Capdevila Morell JA, Costells Rodellas A: [Vitiligo associated with the acquired immunodeficiency syndrome]. Medicina clinica 1994, 103(9):358.

334. Garfein RS, Golub ET, Greenberg AE, Hagan H, Hanson DL, Hudson SM, Kapadia F, Latka MH, Ouellet LJ, Purcell DW et al: A peer-education intervention to reduce injection risk behaviors for HIV and hepatitis C virus infection in young injection drug users. Aids 2007, 21(14):1923-1932.

335. Garfein RS, Swartzendruber A, Ouellet LJ, Kapadia F, Hudson SM, Thiede H, Strathdee SA, Williams IT, Bailey SL, Hagan H et al: Methods to recruit and retain a cohort of young-adult injection drug users for the Third Collaborative Injection Drug Users Study/Drug Users Intervention Trial (CIDUS III/DUIT). Drug Alcohol Depend 2007, 91 Suppl 1:S4-17.

336. Gasiorowski J, Marchewka Z, Lapinski L, Szymanska B, Glowacka K, Knysz B, Dlugosz A, Wiela-Hojenska A: The investigation of specific biochemical markers in monitoring kidney function of drug addicts. Postepy higieny i medycyny doswiadczalnej (Online) 2013, 67:1214-1221.

337. Gavazzi G, Richallet G, Morand P, Bouchard O, Bosseray A, Leclercq P, Micoud M: [Effects of double and triple antiretroviral agents on the HCV viral load in patients coinfected with HIV and HCV]. Pathologie-biologie 1998, 46(6):412-415.

338. Geiger H: [HIV infection and the kidneys]. Deutsche medizinische Wochenschrift (1946) 1991, 116(21):825-831.

339. Getahun H, Gunneberg C, Sculier D, Verster A, Raviglione M: Tuberculosis and HIV in people who inject drugs: evidence for action for tuberculosis, HIV, prison and harm reduction services. Curr Opin HIV AIDS 2012, 7(4):345-353.

340. Getahun H, Matteelli A, Abubakar I, Aziz MA, Baddeley A, Barreira D, Den Boon S, Borroto Gutierrez SM, Bruchfeld J, Burhan E et al: Management of latent Mycobacterium tuberculosis infection: WHO guidelines for low tuberculosis burden countries. The European respiratory journal 2015, 46(6):1563-1576.

341. Ghys PD, Saidel T, Vu HT, Savtchenko I, Erasilova I, Mashologu YS, Indongo R, Sikhosana N, Walker N: Growing in silence: selected regions and countries with expanding HIV/AIDS epidemics. Aids 2003, 17 Suppl 4:S45-50.

342. Giannitrapani L, Soresi M, Balasus D, Licata A, Montalto G: Genetic association of interleukin-6 polymorphism (-174 G/C) with chronic liver diseases and hepatocellular carcinoma. World J Gastroenterol 2013, 19(16):2449-2455.

343. Giard JM, Terrault NA: Women with Cirrhosis: Prevalence, Natural History, and Management. Gastroenterology clinics of North America 2016, 45(2):345-358.

344. Gilbert L, El-Bassel N, Terlikbayeva A, Rozental Y, Chang M, Brisson A, Wu E, Bakpayev M: Couple-based HIV prevention for injecting drug users in Kazakhstan: a pilot intervention study. Journal of prevention & intervention in the community 2010, 38(2):162-176.

345. Gilchrist G, Swan D, Widyaratna K, Marquez-Arrico JE, Hughes E, Mdege ND, Martyn-St James M, Tirado-Munoz J: A Systematic Review and Meta-analysis of Psychosocial Interventions to Reduce Drug and Sexual Blood Borne Virus Risk Behaviours Among People Who Inject Drugs. Aids Behav 2017, 21(7):1791-1811.

346. Gill AJ, Kolson DL: Chronic inflammation and the role for cofactors (hepatitis C, drug abuse, antiretroviral drug toxicity, aging) in HAND persistence. Curr HIV/AIDS Rep 2014, 11(3):325-335.

347. Gillies M, Palmateer N, Hutchinson S, Ahmed S, Taylor A, Goldberg D: The provision of non-needle/syringe drug injecting paraphernalia in the primary prevention of HCV among IDU: a systematic review. BMC Public Health 2010, 10:721.

348. Gish RG, Afdhal NH, Dieterich DT, Reddy KR: Management of hepatitis C virus in special populations: patient and treatment considerations. Clin Gastroenterol Hepatol 2005, 3(4):311-318.

349. Gish RG, Bui TD, Nguyen CT, Nguyen DT, Tran HV, Tran DM, Trinh HN: Liver disease in Viet Nam: screening, surveillance, management and education: a 5-year plan and call to action. J Gastroenterol Hepatol 2012, 27(2):238-247.

350. Gitto S, Micco L, Conti F, Andreone P, Bernardi M: Alcohol and viral hepatitis: a mini-review. Dig Liver Dis 2009, 41(1):67-70.

351. Giunta B, Somboonwit C, Nikolic WV, Rrapo E, Tan J, Shapshak P, Fernandez F: Psychiatric implications of hepatitis-C infection. Critical reviews in neurobiology 2007, 19(2-3):79-118.

352. Goforth HW, Bader M, Fernandez F: Then and now ... HIV consultation psychiatry update. Advances in psychosomatic medicine 2015, 34:49-60.

353. Goldberg D, Anderson E: Hepatitis C: who is at risk and how do we identify them? J Viral Hepat 2004, 11 Suppl 1:12-18.

354. Goldberg D, Taylor A, Hutchinson S, McMenamin J: Hepatitis C infection among injecting drug users in Scotland: stemming the flow. Scott Med J 2000, 45(5):131-132.

355. Gonzalez R, Cherner M: Co-factors in HIV neurobehavioural disturbances: substance abuse, hepatitis C and aging. International review of psychiatry (Abingdon, England) 2008, 20(1):49-60.

356. Gonzalez R, Jacobus J, Martin EM: Investigating neurocognitive features of hepatitis C virus infection in drug users: potential challenges and lessons learned from the HIV literature. Clin Infect Dis 2005, 41 Suppl 1:S45-49.

357. Gonzalez SA, Trotter JF: The rise of the opioid epidemic and hepatitis C-positive organs: A new era in liver transplantation. Hepatology 2018, 67(4):1600-1608.

358. Gonzalez-Lopez A, Fernandez-Martin JI, Gordillo I, Dronda F: [Disseminated Kaposi's sarcoma with hepatosplenic involvement in AIDS]. Medicina clinica 1996, 106(16):622-623.

359. Goodkin K, Shapshak P, Metsch LR, McCoy CB, Crandall KA, Kumar M, Fujimura RK, McCoy V, Zhang BT, Reyblat S et al: Cocaine abuse and HIV-1 infection: epidemiology and neuropathogenesis. Journal of neuroimmunology 1998, 83(1-2):88-101.

360. Gore-Felton C, Koopman C: Behavioral mediation of the relationship between psychosocial factors and HIV disease progression. Psychosomatic medicine 2008, 70(5):569-574.

361. Gowing LR, Hickman M, Degenhardt L: Mitigating the risk of HIV infection with opioid substitution treatment. Bull World Health Organ 2013, 91(2):148-149.

362. Grabowski J, Rhoades H, Elk R, Schmitz J, Creson D: Clinicwide and individualized behavioral interventions in drug dependence treatment. NIDA research monograph 1993, 137:37-72.

363. Grady BP, Schinkel J, Thomas XV, Dalgard O: Hepatitis C virus reinfection following treatment among people who use drugs. Clin Infect Dis 2013, 57 Suppl 2:S105-110.

364. Grady GF: The prevention of (viral) hepatitis. Disease-a-month : DM 1968:1-31.

365. Grady GF: Viral hepatitis. Advances in internal medicine 1972, 18:95-116.

366. Graham NM, Galai N, Nelson KE, Astemborski J, Bonds M, Rizzo RT, Sheeley L, Vlahov D: Effect of isoniazid chemoprophylaxis on HIV-related mycobacterial disease. Arch Intern Med 1996, 156(8):889-894.

367. Grange JM: Complications of bacille Calmette-Guerin (BCG) vaccination and immunotherapy and their management. Communicable disease and public health 1998, 1(2):84-88.

368. Grant BF, Dufour MC, Harford TC: Epidemiology of alcoholic liver disease. Seminars in liver disease 1988, 8(1):12-25.

369. Grant I, Heaton RK, Atkinson JH: Neurocognitive disorders in HIV-1 infection. HNRC Group. HIV Neurobehavioral Research Center. Current topics in microbiology and immunology 1995, 202:11-32.

370. Grebely J, deVlaming S, Duncan F, Viljoen M, Conway B: Current approaches to HCV infection in current and former injection drug users. J Addict Dis 2008, 27(2):25-35.

371. Grebely J, Dore GJ: What is killing people with hepatitis C virus infection? Seminars in liver disease 2011, 31(4):331-339.

372. Grebely J, Dore GJ: Can hepatitis C virus infection be eradicated in people who inject drugs? Antiviral research 2014, 104:62-72.

373. Grebely J, Hajarizadeh B, Dore GJ: Direct-acting antiviral agents for HCV infection affecting people who inject drugs. Nature reviews Gastroenterology & hepatology 2017, 14(11):641-651.

374. Grebely J, Matthews GV, Dore GJ: Treatment of acute HCV infection. Nature reviews Gastroenterology & hepatology 2011, 8(5):265-274.

375. Grebely J, Oser M, Taylor LE, Dore GJ: Breaking down the barriers to hepatitis C virus (HCV) treatment among individuals with HCV/HIV coinfection: action required at the system, provider, and patient levels. The Journal of infectious diseases 2013, 207 Suppl 1:S19-25.

376. Grebely J, Prins M, Hellard M, Cox AL, Osburn WO, Lauer G, Page K, Lloyd AR, Dore GJ: Hepatitis C virus clearance, reinfection, and persistence, with insights from studies of injecting drug users: towards a vaccine. The Lancet Infectious diseases 2012, 12(5):408-414.

377. Grebely J, Tyndall MW: Management of HCV and HIV infections among people who inject drugs. Curr Opin HIV AIDS 2011, 6(6):501-507.

378. Greenfield SF, Shields A, Connery HS, Livchits V, Yanov SA, Lastimoso CS, Strelis AK, Mishustin SP, Fitzmaurice G, Mathew TA et al: Integrated Management of Physician-delivered Alcohol Care for Tuberculosis Patients: Design and Implementation. Alcohol Clin Exp Res 2010, 34(2):317-330.

379. Grellier LF, Dusheiko GM: The role of hepatitis C virus in alcoholic liver disease. Alcohol and alcoholism (Oxford, Oxfordshire) 1997, 32(2):103-111.

380. Grenfell P, Baptista Leite R, Garfein R, de Lussigny S, Platt L, Rhodes T: Tuberculosis, injecting drug use and integrated HIV-TB care: a review of the literature. Drug Alcohol Depend 2013, 129(3):180-209.

381. Grewal P, Viswanathen VA: Liver cancer and alcohol. Clinics in liver disease 2012, 16(4):839-850.

382. Griffin GE: Human immunodeficiency virus infection and the intestine. Bailliere's clinical gastroenterology 1990, 4(3):657-673.

383. Grinberg M, Mansur AJ, Uip DE: [HIV infection. A new aspect to be considered in infectious endocarditis]. Arquivos brasileiros de cardiologia 1992, 59(5):347-350.

384. Grob PJ, Joller-Jemelka HJ: [Hepatitis C virus (HCV), anti-HCV and non-A, non-B hepatitis]. Schweiz Med Wochenschr 1990, 120(5):117-124.

385. Groessl EJ, Sklar M, Cheung RC, Brau N, Ho SB: Increasing antiviral treatment through integrated hepatitis C care: a randomized multicenter trial. Contemp Clin Trials 2013, 35(2):97-107.

386. Grzeszczuk A, Lapinski TW, Panasiuk A: [Levels of hyaluronic acid in chronic viral C hepatitis with relationship to liver steatosis]. Wiadomosci lekarskie (Warsaw, Poland : 1960) 2002, 55(1-2):35-41.

387. Gudowska M, Cylwik B, Chrostek L: The role of serum hyaluronic acid determination in the diagnosis of liver fibrosis. Acta biochimica Polonica 2017, 64(3):451-457.

388. Guilera Sarda M, Sanchez Tapias JM: [Hepatitis G virus: pathogenic and clinical implications]. Gastroenterologia y hepatologia 1997, 20(10):500-509.

389. Guthle M, Dollinger MM: [Epidemiology and risk factors of hepatocellular carcinoma]. Der Radiologe 2014, 54(7):654-659.

390. Gyarmathy VA, Racz J: [Social networks, risk dyads, and their role in the epidemiology and prevention of drug related infectious diseases]. Orvosi hetilap 2010, 151(32):1289-1294.

391. Gyarmathy VA, Racz J: [Epidemiology of hepatitis C and human immunodeficiency virus infections among injecting drug users in Hungary--what's next?]. Orvosi hetilap 2010, 151(10):365-371.

392. Gyarmathy VA, Racz J: [Human immunodeficiency virus (HIV) and Hepatitis C virus (HCV) testing among injecting drug users]. Orvosi hetilap 2011, 152(4):124-130.

393. Hadengue A: [Screening for hepatocellular carcinoma: between cynicism or perfectionism?]. Gastroenterol Clin Biol 2003, 27(5):525-527.

394. Hadler SC: Hepatitis B prevention and human immunodeficiency virus (HIV) infection. Ann Intern Med 1988, 109(2):92-94.

395. Hagan H: Hepatitis C virus transmission dynamics in injection drug users. Subst Use Misuse 1998, 33(5):1197-1212.

396. Hagan H, Campbell JV, Thiede H, Strathdee SA, Ouellet L, Latka M, Hudson S, Garfein RS: Injecting alone among young adult IDUs in five US cities: evidence of low rates of injection risk behavior. Drug Alcohol Depend 2007, 91 Suppl 1:S48-55.

397. Hagan H, Des Jarlais DC: HIV and HCV infection among injecting drug users. The Mount Sinai journal of medicine, New York 2000, 67(5-6):423-428.

398. Hagan H, Des Jarlais DC, Stern R, Lelutiu-Weinberger C, Scheinmann R, Strauss S, Flom PL: HCV synthesis project: preliminary analyses of HCV prevalence in relation to age and duration of injection. The International journal on drug policy 2007, 18(5):341-351.

399. Hagan H, Pouget ER, Des Jarlais DC: A systematic review and meta-analysis of interventions to prevent hepatitis C virus infection in people who inject drugs. The Journal of infectious diseases 2011, 204(1):74-83.

400. Hagan H, Pouget ER, Des Jarlais DC, Lelutiu-Weinberger C: Meta-regression of hepatitis C virus infection in relation to time since onset of illicit drug injection: the influence of time and place. Am J Epidemiol 2008, 168(10):1099-1109.

401. Hagan H, Thiede H, Des Jarlais DC: HIV/hepatitis C virus co-infection in drug users: risk behavior and prevention. Aids 2005, 19 Suppl 3:S199-207.

402. Hahn JA, Samet JH: Alcohol and HIV disease progression: weighing the evidence. Curr HIV/AIDS Rep 2010, 7(4):226-233.

403. Hahne S, Ramsay M, Balogun K, Edmunds WJ, Mortimer P: Incidence and routes of transmission of hepatitis B virus in England and Wales, 1995-2000: implications for immunisation policy. J Clin Virol 2004, 29(4):211-220.

404. Hahne SJ, Veldhuijzen IK, Wiessing L, Lim TA, Salminen M, Laar M: Infection with hepatitis B and C virus in Europe: a systematic review of prevalence and cost-effectiveness of screening. BMC Infect Dis 2013, 13:181.

405. Haig T: Randomized controlled trial proves effectiveness of methadone maintenance treatment in prison. Canadian HIV/AIDS policy & law review 2003, 8(3):48.

406. Hallam A, Kerlin P: Viral hepatitis A to E. An update. Aust Fam Physician 1991, 20(6):760, 762-763, 766-770.

407. Hally RJ, Rubin RA, Fraimow HS, Hoffman-Terry ML: Fatal Vibrio parahemolyticus septicemia in a patient with cirrhosis. A case report and review of the literature. Dig Dis Sci 1995, 40(6):1257-1260.

408. Harada M: [Autophagy in liver diseases]. Journal of UOEH 2011, 33(4):337-344.

409. Harris M, Rhodes T: Hepatitis C treatment access and uptake for people who inject drugs: a review mapping the role of social factors. Harm Reduct J 2013, 10:7.

410. Hauser KF, El-Hage N, Buch S, Nath A, Tyor WR, Bruce-Keller AJ, Knapp PE: Impact of opiate-HIV-1 interactions on neurotoxic signaling. J Neuroimmune Pharmacol 2006, 1(1):98-105.

411. Hauser KF, El-Hage N, Stiene-Martin A, Maragos WF, Nath A, Persidsky Y, Volsky DJ, Knapp PE: HIV-1 neuropathogenesis: glial mechanisms revealed through substance abuse. Journal of neurochemistry 2007, 100(3):567-586.

412. Hauser KF, Fitting S, Dever SM, Podhaizer EM, Knapp PE: Opiate drug use and the pathophysiology of neuroAIDS. Curr HIV Res 2012, 10(5):435-452.

413. Hauser P, Fuller B, Ho SB, Thuras P, Kern S, Dieperink E: The safety and efficacy of baclofen to reduce alcohol use in veterans with chronic hepatitis C: a randomized controlled trial. Addiction 2017, 112(7):1173-1183.

414. Haverkos HW: Infectious diseases and drug abuse. Prevention and treatment in the drug abuse treatment system. J Subst Abuse Treat 1991, 8(4):269-275.

415. Haverkos HW, Lange WR: From the Alcohol, Drug Abuse, and Mental Health Administration. Serious infections other than human immunodeficiency virus among intravenous drug abusers. The Journal of infectious diseases 1990, 161(5):894-902.

416. Hayes-Larson E, Hirsch-Moverman Y, Saito S, Frederix K, Pitt B, Maama-Maime L, Howard AA: Depressive symptoms and hazardous/harmful alcohol use are prevalent and correlate with stigma among TB-HIV patients in Lesotho. Int J Tuberc Lung Dis 2017, 21(11):34-41.

417. Heidelbaugh JJ, Bruderly M: Cirrhosis and chronic liver failure: part I. Diagnosis and evaluation. Am Fam Physician 2006, 74(5):756-762.

418. Heintges T, Wands JR: Hepatitis C virus: epidemiology and transmission. Hepatology 1997, 26(3):521-526.

419. Hellard M, Sacks-Davis R, Gold J: Hepatitis C treatment for injection drug users: a review of the available evidence. Clin Infect Dis 2009, 49(4):561-573.

420. Hensel FJ, Heintges T, Petry W, Niederau C, Haussinger D: [Form of progression of chronic hepatitis C with viral coinfection and additional liver diseases]. Deutsche medizinische Wochenschrift (1946) 1998, 123(6):161-166.

421. Hernandez Flix S, Sauret J, Ausina V, Condom MJ, Rodriguez Frojan G, Luquin M, Cornudella R: [Pulmonary disease caused by opportunistic environmental mycobacteria. Review of 35 cases]. Medicina clinica 1990, 95(2):53-56.

422. Herold C, Ganslmayer M, Ocker M, Zopf S, Gailer B, Hahn EG, Schuppan D: Inducibility of microsomal liver function may differentiate cirrhotic patients with maintained compared with severely compromised liver reserve. J Gastroenterol Hepatol 2003, 18(4):445-449.

423. Herrmann ES, Matusiewicz AK, Stitzer ML, Higgins ST, Sigmon SC, Heil SH: Contingency Management Interventions for HIV, Tuberculosis, and Hepatitis Control Among Individuals With Substance Use Disorders: A Systematized Review. J Subst Abuse Treat 2017, 72:117-125.

424. Hickman M, De Angelis D, Vickerman P, Hutchinson S, Martin NK: Hepatitis C virus treatment as prevention in people who inject drugs: testing the evidence. Current opinion in infectious diseases 2015, 28(6):576-582.

425. Hickman M, McDonald T, Judd A, Nichols T, Hope V, Skidmore S, Parry JV: Increasing the uptake of hepatitis C virus testing among injecting drug users in specialist drug treatment and prison settings by using dried blood spots for diagnostic testing: a cluster randomized controlled trial. J Viral Hepat 2008, 15(4):250-254.

426. Hift RJ: Chronic hepatitis. South African medical journal = Suid-Afrikaanse tydskrif vir geneeskunde 1994, 84(8 Pt 2):559-563.

427. Hilsabeck RC, Castellon SA, Hinkin CH: Neuropsychological aspects of coinfection with HIV and hepatitis C virus. Clin Infect Dis 2005, 41 Suppl 1:S38-44.

428. Hilsden RJ, Macphail G, Grebely J, Conway B, Lee SS: Directly observed pegylated interferon plus self-administered ribavirin for the treatment of hepatitis C virus infection in people actively using drugs: a randomized controlled trial. Clin Infect Dis 2013, 57 Suppl 2:S90-96.

429. Hisada M, Chatterjee N, Zhang M, Battjes RJ, Goedert JJ: Increased hepatitis C virus load among injection drug users infected with human immunodeficiency virus and human T lymphotropic virus type II. The Journal of infectious diseases 2003, 188(6):891-897.

430. Ho SB, Brau N, Cheung R, Liu L, Sanchez C, Sklar M, Phelps TE, Marcus SG, Wasil MM, Tisi A et al: Integrated Care Increases Treatment and Improves Outcomes of Patients With Chronic Hepatitis C Virus Infection and Psychiatric Illness or Substance Abuse. Clin Gastroenterol Hepatol 2015, 13(11):2005-2014.e2001-2003.

431. Ho SB, Groessl E, Dollarhide A, Robinson S, Kravetz D, Dieperink E: Management of chronic hepatitis C in veterans: the potential of integrated care models. The American journal of gastroenterology 2008, 103(7):1810-1823.

432. Hochstatter KR, Hull SJ, Stockman LJ, Stephens LK, Olson-Streed HK, Ehlenbach WJ, Repplinger MD, Jacobs EA, Westergaard RP: Using database linkages to monitor the continuum of care for hepatitis C virus among syringe exchange clients: Experience from a pilot intervention. The International journal on drug policy 2017, 42:22-25.

433. Hollinger FB: Factors contributing to the evolution and outcome of cirrhosis in hepatitis C. Clinics in liver disease 1999, 3(4):741-755, viii.

434. Holtzheimer PE, Veitengruber J, Wang CC, Krows M, Thiede H, Wald A, Roy-Byrne P: Utility of the Beck Depression Inventory to screen for and track depression in injection drug users seeking hepatitis C treatment. Gen Hosp Psychiatry 2010, 32(4):426-432.

435. Hotho DM, Bezemer G, Hansen BE, Van Gool AR, de Knegt RJ, Janssen HL, Veldt BJ: Effects of escitalopram prophylaxis during antiviral treatment for chronic hepatitis C in patients with a history of intravenous drug use and depression. J Clin Psychiatry 2014, 75(10):1069-1077.

436. Howell C, Jeffers L, Hoofnagle JH: Hepatitis C in African Americans: summary of a workshop. Gastroenterology 2000, 119(5):1385-1396.

437. Hser YI, Liang D, Lan YC, Vicknasingam BK, Chakrabarti A: Drug Abuse, HIV, and HCV in Asian Countries. J Neuroimmune Pharmacol 2016, 11(3):383-393.

438. Hu Y, Grau LE, Scott G, Seal KH, Marshall PA, Singer M, Heimer R: Economic evaluation of delivering hepatitis B vaccine to injection drug users. Am J Prev Med 2008, 35(1):25-32.

439. Huang QT, Hang LL, Zhong M, Gao YF, Luo ML, Yu YH: Maternal HCV infection is associated with intrauterine fetal growth disturbance: A meta-analysis of observational studies. Medicine 2016, 95(35):e4777.

440. Huang Y, Adams LA, Joseph J, Bulsara MK, Jeffrey GP: The ability of Hepascore to predict liver fibrosis in chronic liver disease: a meta-analysis. Liver Int 2017, 37(1):121-131.

441. Huber M, Weber R, Oppliger R, Vernazza P, Schmid P, Schonbucher P, Bertisch B, Meili D, Renner EL: Interferon alpha-2a plus ribavirin 1,000/1,200 mg versus interferon alpha-2a plus ribavirin 600 mg for chronic hepatitis C infection in patients on opiate maintenance treatment: an open-label randomized multicenter trial. Infection 2005, 33(1):25-29.

442. Hughes RA: Drug injectors and the cleaning of needles and syringes. Eur Addict Res 2000, 6(1):20-30.

443. Hult B, Chana G, Masliah E, Everall I: Neurobiology of HIV. International review of psychiatry (Abingdon, England) 2008, 20(1):3-13.

444. Hutchinson SJ, Dillon JF, Fox R, McDonald SA, Innes HA, Weir A, McLeod A, Aspinall EJ, Palmateer NE, Taylor A et al: Expansion of HCV treatment access to people who have injected drugs through effective translation of research into public health policy: Scotland's experience. The International journal on drug policy 2015, 26(11):1041-1049.

445. Hutchinson SJ, Roy KM, Wadd S, Bird SM, Taylor A, Anderson E, Shaw L, Codere G, Goldberg DJ: Hepatitis C virus infection in Scotland: epidemiological review and public health challenges. Scott Med J 2006, 51(2):8-15.

446. Hwang SW, Tolomiczenko G, Kouyoumdjian FG, Garner RE: Interventions to improve the health of the homeless: a systematic review. Am J Prev Med 2005, 29(4):311-319.

447. Imbert A, Colombat M, Capron JP: [Diagnostic strategy when confronted with a moderate and prolonged increase of transaminases]. Presse Med 2003, 32(2):73-78.

448. Imperial JC: Chronic hepatitis C in the state prison system: insights into the problems and possible solutions. Expert review of gastroenterology & hepatology 2010, 4(3):355-364.

449. Inciardi JA, Page JB: Drug sharing among intravenous drug users. Aids 1991, 5(6):772-773.

450. Ingersoll KS, Farrell-Carnahan L, Cohen-Filipic J, Heckman CJ, Ceperich SD, Hettema J, Marzani-Nissen G: A pilot randomized clinical trial of two medication adherence and drug use interventions for HIV+ crack cocaine users. Drug Alcohol Depend 2011, 116(1-3):177-187.

451. Ingrosso L, Vescio F, Giuliani M, Migliori GB, Fattorini L, Severoni S, Rezza G: Risk factors for tuberculosis in foreign-born people (FBP) in Italy: a systematic review and meta-analysis. PLoS One 2014, 9(4):e94728.

452. Inoue H, Seitz HK: Viruses and alcohol in the pathogenesis of primary hepatic carcinoma. European journal of cancer prevention : the official journal of the European Cancer Prevention Organisation (ECP) 2001, 10(1):107-110.

453. Inoue K, Yoshiba M, Sekiyama K, Koh I, Fujita R: [Pathogenic mechanism of fulminant hepatic failure in HBs Ag carrier after withdrawal of cytotoxic chemotherapy]. Nihon rinsho Japanese journal of clinical medicine 1995, 53 Suppl(Pt 2):467-472.

454. Isaguliants MG, Ozeretskovskaia NN, Znoiko OO, Tsyganova EV: [Role of host in spontaneous recovery from viral hepatitis C: the body's condition at the moment of infection]. Voprosy virusologii 2008, 53(2):4-9.

455. Ishii H, Horie Y, Tomita K, Yamagishi Y: [Recent progress in alcoholic liver diseases]. Nihon Shokakibyo Gakkai zasshi = The Japanese journal of gastro-enterology 2003, 100(10):1187-1197.

456. Iversen J, Page K, Madden A, Maher L: HIV, HCV, and Health-Related Harms Among Women Who Inject Drugs: Implications for Prevention and Treatment. J Acquir Immune Defic Syndr 2015, 69 Suppl 2:S176-181.

457. Izquierdo Patron M, Villena Garrido MV: [Bronchiectasis associated with human immunodeficiency virus infection]. Archivos de bronconeumologia 1995, 31(4):181-183.

458. Jacobson JM, Worner TM, Sacks HS, Lieber CS: Human immunodeficiency virus and hepatitis B virus infections in alcoholics. Progress in clinical and biological research 1990, 325:67-73.

459. Jamal MM, Morgan TR: Liver disease in alcohol and hepatitis C. Best practice & research Clinical gastroenterology 2003, 17(4):649-662.

460. Jamal MM, Saadi Z, Morgan TR: Alcohol and hepatitis C. Digestive diseases (Basel, Switzerland) 2005, 23(3-4):285-296.

461. Janssen W, Trubner K, Puschel K: Death caused by drug addiction: a review of the experiences in Hamburg and the situation in the Federal Republic of Germany in comparison with the literature. Forensic science international 1989, 43(3):223-237.

462. Javaloyas M, Vallet J, Sueiras A, Gonzalez X: [A neck abscess due to Streptococcus pneumoniae in a man with HIV infection]. Medicina clinica 1999, 113(20):795-796.

463. Jerrells TR: Association of alcohol consumption and exaggerated immunopathologic effects in the liver induced by infectious organism. Frontiers in bioscience : a journal and virtual library 2002, 7:d1487-1493.

464. Jewkes RK, Dunkle K, Nduna M, Jama PN, Puren A: Associations between childhood adversity and depression, substance abuse and HIV and HSV2 incident infections in rural South African youth. Child abuse & neglect 2010, 34(11):833-841.

465. Jhaveri R: Diagnosis and management of hepatitis C virus-infected children. The Pediatric infectious disease journal 2011, 30(11):983-985.

466. Jiang D: Care of chronic liver disease. Primary care 2011, 38(3):483-498; viii-ix.

467. Jiao M, Greanya ED, Haque M, Yoshida EM, Soos JG: Methadone maintenance therapy in liver transplantation. Progress in transplantation (Aliso Viejo, Calif) 2010, 20(3):209-214; quiz 215.

468. John-Baptiste A, Krahn M, Heathcote J, Laporte A, Tomlinson G: The natural history of hepatitis C infection acquired through injection drug use: meta-analysis and meta-regression. J Hepatol 2010, 53(2):245-251.

469. John-Baptiste A, Yeung MW, Leung V, van der Velde G, Krahn M: Cost effectiveness of hepatitis C-related interventions targeting substance users and other high-risk groups: a systematic review. PharmacoEconomics 2012, 30(11):1015-1034.

470. Johnston JC, Shahidi NC, Sadatsafavi M, Fitzgerald JM: Treatment outcomes of multidrug-resistant tuberculosis: a systematic review and meta-analysis. PLoS One 2009, 4(9):e6914.

471. Jones L, Atkinson A, Bates G, McCoy E, Porcellato L, Beynon C, McVeigh J, Bellis MA: Views and experiences of hepatitis C testing and diagnosis among people who inject drugs: systematic review of qualitative research. The International journal on drug policy 2014, 25(2):204-211.

472. Jones L, Bates G, McCoy E, Beynon C, McVeigh J, Bellis MA: Effectiveness of interventions to increase hepatitis C testing uptake among high-risk groups: a systematic review. Eur J Public Health 2014, 24(5):781-788.

473. Jones L, Pickering L, Sumnall H, McVeigh J, Bellis MA: Optimal provision of needle and syringe programmes for injecting drug users: A systematic review. The International journal on drug policy 2010, 21(5):335-342.

474. Jordan AE, Jarlais DD, Hagan H: Prescription opioid misuse and its relation to injection drug use and hepatitis C virus infection: protocol for a systematic review and meta-analysis. Systematic reviews 2014, 3:95.

475. Jordan AE, Perlman DC, Neurer J, Smith DJ, Des Jarlais DC, Hagan H: Prevalence of hepatitis C virus infection among HIV+ men who have sex with men: a systematic review and meta-analysis. Int J STD AIDS 2017, 28(2):145-159.

476. Joshi K, Kohli A, Manch R, Gish R: Alcoholic Liver Disease: High Risk or Low Risk for Developing Hepatocellular Carcinoma? Clinics in liver disease 2016, 20(3):563-580.

477. Jurgens R, Betteridge G: Prisoners who inject drugs: public health and human rights imperatives. Health and human rights 2005, 8(2):46-74.

478. Jurgensen O, Altermatt HJ, von Overbeck J, Berthold H: [Necrotizing vasculitis of the tongue. A contribution to the differential diagnosis of ulcerative mucosal changes in HIV-infected patients].

Schweizer Monatsschrift fur Zahnmedizin = Revue mensuelle suisse d'odonto-stomatologie = Rivista mensile svizzera di odontologia e stomatologia 1995, 105(1):54-62.

479. Kaczynski J: [Prevention of cirrhosis is the best measure against hepatocellular cancer]. Lakartidningen 1999, 96(4):338-341.

480. Kamarulzaman A, Altice FL: Challenges in managing HIV in people who use drugs. Current opinion in infectious diseases 2015, 28(1):10-16.

481. Kamath GR, Shah DP, Hwang LY: Immune response to hepatitis B vaccination in drug using populations: a systematic review and meta-regression analysis. Vaccine 2014, 32(20):2265-2274.

482. Kanematsu M, Semelka RC, Matsuo M, Kondo H, Enya M, Goshima S, Moriyama N, Hoshi H: Gadolinium-enhanced MR imaging of the liver: optimizing imaging delay for hepatic arterial and portal venous phases--a prospective randomized study in patients with chronic liver damage. Radiology 2002, 225(2):407-415.

483. Kapadia F, Latka MH, Hagan H, Golub ET, Campbell JV, Coady MH, Garfein RS, Thomas DL, Bonner S, Thiel T et al: Design and feasibility of a randomized behavioral intervention to reduce distributive injection risk and improve health-care access among hepatitis C virus positive injection drug users: the Study to Reduce Intravenous Exposures (STRIVE). Journal of urban health : bulletin of the New York Academy of Medicine 2007, 84(1):99-115.

484. Karmochkine M, Carrat F, Valleron AJ, Raguin G: [Transmission modes of hepatitis C virus]. Presse Med 1998, 27(18):871-876.

485. Karmochkine M, Piette AM, Chapman A: [The glomerulopathies of cirrhosis of the liver]. Presse Med 1989, 18(17):876-879.

486. Karpatkin S: Autoimmune thrombocytopenia and AIDS-related thrombocytopenia. Current opinion in immunology 1989, 2(4):625-632.

487. Karpatkin S: HIV-1-related thrombocytopenia. Bailliere's clinical haematology 1990, 3(1):115-138.

488. Karpatkin S, Nardi MA, Hymes KB: Sequestration of anti-platelet GPIIIa antibody in rheumatoid factor immune complexes of human immunodeficiency virus 1 thrombocytopenic patients. Proceedings of the National Academy of Sciences of the United States of America 1995, 92(6):2263-2267.

489. Kartashova O, Terent'eva LA, Zaltsmane VK, Maksimova LA: [Hepatocyte pathology in viral and toxic injuries to the liver]. Vestnik Akademii meditsinskikh nauk SSSR 1983(11):74-81.

490. Kater L, Borst-Eilers E: [Virus hepatitis B (serum hepatitis) I. Clinical data and epidemiology]. Ned Tijdschr Geneeskd 1975, 119(34):1312-1319.

491. Kazennova EV, Bobkov AF: [Subtypes of human immunodeficiency virus 1:classification, the origin and circulation in Europe]. Zh Mikrobiol Epidemiol Immunobiol 2003(1):90-96.

492. Kelly JA, Murphy DA: Some lessons learned about risk reduction after ten years of the HIV/AIDS epidemic. AIDS Care 1991, 3(3):251-257.

493. Kentikelenis A, Karanikolos M, Williams G, Mladovsky P, King L, Pharris A, Suk JE, Hatzakis A, McKee M, Noori T et al: How do economic crises affect migrants' risk of infectious disease? A systematic-narrative review. Eur J Public Health 2015, 25(6):937-944.

494. Kew MC: Hepatitis B and C viruses and hepatocellular carcinoma. Clinics in laboratory medicine 1996, 16(2):395-406.

495. Kew MC, Popper H: Relationship between hepatocellular carcinoma and cirrhosis. Seminars in liver disease 1984, 4(2):136-146.

496. Khalsa JH, Elkashef A: Interventions for HIV and hepatitis C virus infections in recreational drug users. Clin Infect Dis 2010, 50(11):1505-1511.

497. Khalsa JH, Kresina T, Sherman K, Vocci F: Medical management of HIV-hepatitis C virus coinfection in injection drug users. Clin Infect Dis 2005, 41 Suppl 1:S1-6.

498. Khalsa JH, Treisman G, McCance-Katz E, Tedaldi E: Medical consequences of drug abuse and co-occurring infections: research at the National Institute on Drug Abuse. Subst Abus 2008, 29(3):5-16.

499. Khalsa JH, Vocci F: Clinical management of drug addicts infected with human immunodeficiency virus and hepatitis C virus. J Addict Dis 2008, 27(2):1-10.

500. Khan R, Singal AK, Anand BS: Outcomes after liver transplantation for combined alcohol and hepatitis C virus infection. World J Gastroenterol 2014, 20(34):11935-11938.

501. Kharbanda KK, Bardag-Gorce F, Barve S, Molina PE, Osna NA: Impact of altered methylation in cytokine signaling and proteasome function in alcohol and viral-mediated diseases. Alcohol Clin Exp Res 2013, 37(1):1-7.

502. Khazanov AI: [Current problems of viral and alcohol diseases of the liver]. Klinicheskaia meditsina 2002, 80(3):14-19.

503. Khouri LD, Shammaa M: Alcoholic liver disease. Le Journal medical libanais The Lebanese medical journal 2001, 49(2):94-100.

504. Kim AY, Chung RT: Coinfection with HIV-1 and HCV--a one-two punch. Gastroenterology 2009, 137(3):795-814.

505. Kim RG, Loomba R, Prokop LJ, Singh S: Statin Use and Risk of Cirrhosis and Related Complications in Patients With Chronic Liver Diseases: A Systematic Review and Meta-analysis. Clin Gastroenterol Hepatol 2017, 15(10):1521-1530.e1528.

506. King JR, Menon RM: Ombitasvir/Paritaprevir/Ritonavir and Dasabuvir: Drug Interactions With Antiretroviral Agents and Drugs forSubstance Abuse. Clinical pharmacology in drug development 2017, 6(2):201-205.

507. Kittikraisak W, van Griensven F, Martin M, McNicholl J, Gilbert PB, Chuachoowong R, Vanichseni S, Sutthent R, Tappero JW, Mastro TD et al: Blood and seminal plasma HIV-1 RNA levels among HIV-1-infected injecting drug users participating in the AIDSVAX B/E efficacy trial in Bangkok, Thailand. J Acquir Immune Defic Syndr 2009, 51(5):601-608.

508. Klevens RM, Hu DJ, Jiles R, Holmberg SD: Evolving epidemiology of hepatitis C virus in the United States. Clin Infect Dis 2012, 55 Suppl 1:S3-9.

509. Klimas J, Field CA, Cullen W, O'Gorman CS, Glynn LG, Keenan E, Saunders J, Bury G, Dunne C: Psychosocial interventions to reduce alcohol consumption in concurrent problem alcohol and illicit drug users. The Cochrane database of systematic reviews 2012, 11:Cd009269.

510. Klimas J, Tobin H, Field CA, O'Gorman CS, Glynn LG, Keenan E, Saunders J, Bury G, Dunne C, Cullen W: Psychosocial interventions to reduce alcohol consumption in concurrent problem alcohol and illicit drug users. The Cochrane database of systematic reviews 2014(12):Cd009269.

511. Koff RS: Prevention of hepatitis C virus infection. Clinics in liver disease 1997, 1(3):603-613.

512. Koff RS, Dienstag JL: Extrahepatic manifestations of hepatitis C and the association with alcoholic liver disease. Seminars in liver disease 1995, 15(1):101-109.

513. Koffarnus MN, Wong CJ, Diemer K, Needham M, Hampton J, Fingerhood M, Svikis DS, Bigelow GE, Silverman K: A randomized clinical trial of a Therapeutic Workplace for chronically unemployed, homeless, alcohol-dependent adults. Alcohol and alcoholism (Oxford, Oxfordshire) 2011, 46(5):561-569.

514. Kojima M, Kanazawa K, Hakamada T, Aikawa T: [Infection with hepatitis GB virus C in intravenous drug abusers with type C chronic liver diseases]. Nihon rinsho Japanese journal of clinical medicine 1997, 55(3):549-553.

515. Kokotailo P: Physical health problems associated with adolescent substance abuse. NIDA research monograph 1995, 156:112-129.

516. Komada T, Miyazaki A, Sato N: [Etiology and pathophysiology of alcoholic hepatitis]. Nihon Naika Gakkai zasshi The Journal of the Japanese Society of Internal Medicine 1994, 83(2):216-219.

517. Koppel BS, Tuchman AJ, Mangiardi JR, Daras M, Weitzner I: Epidural spinal infection in intravenous drug abusers. Archives of neurology 1988, 45(12):1331-1337.

518. Korenaga M, Okuda M, Otani K, Wang T, Li Y, Weinman SA: Mitochondrial dysfunction in hepatitis C. J Clin Gastroenterol 2005, 39(4 Suppl 2):S162-166.

519. Korthuis PT, Feaster DJ, Gomez ZL, Das M, Tross S, Wiest K, Douaihy A, Mandler RN, Sorensen JL, Colfax G et al: Injection behaviors among injection drug users in treatment: the role of hepatitis C awareness. Addict Behav 2012, 37(4):552-555.

520. Kostic V, Radovic J, Djordjevic J, Vujic S: Hepatitis C viral infection among prisoners. Vojnosanitetski pregled 2013, 70(11):1006-1009.

521. Kotlyar DS, Campbell MS, Reddy KR: Recurrence of diseases following orthotopic liver transplantation. The American journal of gastroenterology 2006, 101(6):1370-1378.

522. Kreek MJ, Gutjahr CL, Garfield JW, Bowen DV, Field FH: Drug interactions with methadone. Annals of the New York Academy of Sciences 1976, 281:350-371.

523. Kresina TF, Bruce RD, Cargill VA, Cheever LW: Integrating care for hepatitis C virus (HCV) and primary care for HIV for injection drug users coinfected with HIV and HCV. Clin Infect Dis 2005, 41 Suppl 1:S83-88.

524. Kresina TF, Eldred L, Bruce RD, Francis H: Integration of pharmacotherapy for opioid addiction into HIV primary care for HIV/hepatitis C virus-co-infected patients. Aids 2005, 19 Suppl 3:S221-226.

525. Kresina TF, Flexner CW, Sinclair J, Correia MA, Stapleton JT, Adeniyi-Jones S, Cargill V, Cheever LW: Alcohol use and HIV pharmacotherapy. AIDS research and human retroviruses 2002, 18(11):757-770.

526. Kresina TF, Mathieson B: Human immunodeficiency virus type 1 infection, mucosal immunity, and pathogenesis and extramural research programs at the National Institutes of Health. The Journal of infectious diseases 1999, 179 Suppl 3:S392-396.

527. Kretzschmar M, Wiessing L: New challenges for mathematical and statistical modeling of HIV and hepatitis C virus in injecting drug users. Aids 2008, 22(13):1527-1537.

528. Kucherov AL, Rybkina TA: [Factors responsible for increased risk of tuberculosis]. Probl Tuberk 1988(12):57-61.

529. Kucirka LM, Sarathy H, Govindan P, Wolf JH, Ellison TA, Hart LJ, Montgomery RA, Ros RL, Segev DL: Risk of window period hepatitis-C infection in high infectious risk donors: systematic review and meta-analysis. American journal of transplantation : official journal of the American Society of Transplantation and the American Society of Transplant Surgeons 2011, 11(6):1188-1200.

530. Kuiken C, Thakallapalli R, Esklid A, de Ronde A: Genetic analysis reveals epidemiologic patterns in the spread of human immunodeficiency virus. Am J Epidemiol 2000, 152(9):814-822.

531. Kulig CC, Beresford TP: Hepatitis C in alcohol dependence:drinking versus disulfiram. J Addict Dis 2005, 24(2):77-89.

532. Kumada H: [Corticosteroid withdrawal therapy for hepatitis B]. Nihon rinsho Japanese journal of clinical medicine 1995, 53 Suppl(Pt 2):635-639.

533. Kumar M, Kumar AM, Waldrop D, Antoni MH, Schneiderman N, Eisdorfer C: The HPA axis in HIV-1 infection. J Acquir Immune Defic Syndr 2002, 31 Suppl 2:S89-93.

534. Kumari P, Meena LS: Factors affecting susceptibility to Mycobacterium tuberculosis: a close view of immunological defence mechanism. Applied biochemistry and biotechnology 2014, 174(8):2663-2673.

535. Kuntz HD, Rausch V: [Hepatoxic side-effects of rifampicin. A comparative clinical study]. Die Medizinische Welt 1977, 28(47):1940-1942.

536. Kwak DS, Jun DW, Seo JG, Chung WS, Park SE, Lee KN, Khalid-Saeed W, Lee HL, Lee OY, Yoon BC et al: Short-term probiotic therapy alleviates small intestinal bacterial overgrowth, but does not improve intestinal permeability in chronic liver disease. Eur J Gastroenterol Hepatol 2014, 26(12):1353-1359.

537. Lake JR: Changing indications for liver transplantation. Gastroenterology clinics of North America 1993, 22(2):213-229.

538. Lalezari J, Sullivan JG, Varunok P, Galen E, Kowdley KV, Rustgi V, Aguilar H, Felizarta F, McGovern B, King M et al: Ombitasvir/paritaprevir/r and dasabuvir plus ribavirin in HCV genotype 1-infected patients on methadone or buprenorphine. J Hepatol 2015, 63(2):364-369.

539. Lampo N, Malinverni R: [Hepatitis C and alcohol, which is the limit?]. Revue medicale de la Suisse romande 2003, 123(4):241-243.

540. Langlet P, Mulkay JP, Holvoet J: Issues in managing patients with chronic hepatitis C in public hospitals. Acta gastro-enterologica Belgica 2002, 65(2):101-103.

541. Lansang MA: Epidemiology and control of hepatitis B infection: a perspective from the Philippines, Asia. Gut 1996, 38 Suppl 2:S43-47.

542. Lansky A, Finlayson T, Johnson C, Holtzman D, Wejnert C, Mitsch A, Gust D, Chen R, Mizuno Y, Crepaz N: Estimating the number of persons who inject drugs in the united states by meta-analysis to calculate national rates of HIV and hepatitis C virus infections. PLoS One 2014, 9(5):e97596.

543. Larney S, Peacock A, Leung J, Colledge S, Hickman M, Vickerman P, Grebely J, Dumchev KV, Griffiths P, Hines L et al: Global, regional, and country-level coverage of interventions to prevent and manage HIV and hepatitis C among people who inject drugs: a systematic review. The Lancet Global health 2017, 5(12):e1208-e1220.

544. Larrey D: [Chronic hepatitis C: apart from antiviral treatment what other measures should be recommended?]. Gastroenterol Clin Biol 2002, 26 Spec No 2:B283-290.

545. Larson R, Capili B, Eckert-Norton M, Colagreco JP, Anastasi JK: Disorders of glucose metabolism in the context of human immunodeficiency virus infection. Journal of the American Academy of Nurse Practitioners 2006, 18(3):92-103.

546. Laskus T: [Liver damage and hepatotropic virus infections in drug addicts]. Wiadomosci lekarskie (Warsaw, Poland : 1960) 1992, 45(15-16):615-618.

547. Laskus T, Babiuch L, Rydz-Swoboda U: [Primary liver cancer: epidemiologic and pathogenetic aspects]. Polski tygodnik lekarski (Warsaw, Poland : 1960) 1987, 42(15):452-454.

548. Laskus T, Cianciara J, Babiuch L, Slusarczyk J: [Hepatitis B virus infection in alcoholics]. Polski tygodnik lekarski (Warsaw, Poland : 1960) 1992, 47(11-13):280-282.

549. Latka MH, Hagan H, Kapadia F, Golub ET, Bonner S, Campbell JV, Coady MH, Garfein RS, Pu M, Thomas DL et al: A randomized intervention trial to reduce the lending of used injection equipment among injection drug users infected with hepatitis C. Am J Public Health 2008, 98(5):853-861.

550. Lau CY, Cardinali M, Sato PA, Fix A, Flores J: Broadening inclusion of vulnerable populations in HIV vaccine trials. Expert review of vaccines 2008, 7(2):259-268.

551. Laurence J: Durability of clinical responses to anti-HIV therapies. AIDS Read 2008, 18(3):120-121.

552. Lazarus JV, Sperle I, Maticic M, Wiessing L: A systematic review of Hepatitis C virus treatment uptake among people who inject drugs in the European Region. BMC Infect Dis 2014, 14 Suppl 6:S16.

553. Leal P, Stein K, Rosenberg W: What is the cost utility of screening for hepatitis C virus (HCV) in intravenous drug users? Journal of medical screening 1999, 6(3):124-131.

554. Leao JC, Teo CG, Porter SR: HCV infection: aspects of epidemiology and transmission relevant to oral health care workers. International journal of oral and maxillofacial surgery 2006, 35(4):295-300.

555. Leask JD, Dillon JF: Review article: treatment as prevention - targeting people who inject drugs as a pathway towards hepatitis C eradication. Aliment Pharmacol Ther 2016, 44(2):145-156.

556. Lee JJ, Kwon OS: [Occult hepatitis B virus infection and hepatocellular carcinoma]. The Korean journal of gastroenterology = Taehan Sohwagi Hakhoe chi 2013, 62(3):160-164.

557. Lefilliatre P, Villeneuve JP: Fulminant hepatitis A in patients with chronic liver disease. Canadian journal of public health = Revue canadienne de sante publique 2000, 91(3):168-170.

558. Leggio L, Ferrulli A, Zambon A, Caputo F, Kenna GA, Swift RM, Addolorato G: Baclofen promotes alcohol abstinence in alcohol dependent cirrhotic patients with hepatitis C virus (HCV) infection. Addict Behav 2012, 37(4):561-564.

559. Legramante A, Pelagalli LA, Santilli E: [Side-effects of antitubercular chemoantibiotic therapy]. La Clinica terapeutica 1977, 81(4):363-379.

560. Leinikki P: [HIV epidemic in intravenous drug users--is Finland behind or ahead of other Western countries?]. Duodecim; laaketieteellinen aikakauskirja 2000, 116(1):55-60.

561. Lelutiu-Weinberger C, Pouget ER, Des Jarlais DD, Cooper HL, Scheinmann R, Stern R, Strauss SM, Hagan H: A meta-analysis of the hepatitis C virus distribution in diverse racial/ethnic drug injector groups. Soc Sci Med 2009, 68(3):579-590.

562. Lert F: [Can we stop the hepatitis C virus transmission in drug users?]. Rev Epidemiol Sante Publique 2006, 54 Spec No 1:1s61-61s67.

563. Levine OS, Vlahov D, Nelson KE: Epidemiology of hepatitis B virus infections among injecting drug users: seroprevalence, risk factors, and viral interactions. Epidemiologic reviews 1994, 16(2):418-436.

564. Lewis H, Kunkel J, Axten D, Dalton J, Gardner H, Tippett A, Wynne S, Wilkinson M, Foster GR: Community nurse-led initiation of antiviral therapy for chronic hepatitis C in people who inject drugs does not increase uptake of or adherence to treatment. Eur J Gastroenterol Hepatol 2016, 28(11):1258-1263.

565. Lewis W: Cardiomyopathy in AIDS: a pathophysiological perspective. Progress in cardiovascular diseases 2000, 43(2):151-170.

566. Lewis W: Use of the transgenic mouse in models of AIDS cardiomyopathy. Aids 2003, 17 Suppl 1:S36-45.

567. Libraty DH, Byrd TF: Cutaneous miliary tuberculosis in the AIDS era: case report and review. Clin Infect Dis 1996, 23(4):706-710.

568. Liddle C: Hepatitis C. Anaesthesia and intensive care 1996, 24(2):180-183.

569. Lieber CS: Alcoholic liver disease: new insights in pathogenesis lead to new treatments. J Hepatol 2000, 32(1 Suppl):113-128.

570. Lieber CS: Alcohol and hepatitis C. Alcohol research & health : the journal of the National Institute on Alcohol Abuse and Alcoholism 2001, 25(4):245-254.

571. Lieber CS: Liver diseases by alcohol and hepatitis C: early detection and new insights in pathogenesis lead to improved treatment. Am J Addict 2001, 10(s1):s29-s50.

572. Lillebaek T, Andersen AB, Seersholm NJ, Thomsen VO: [Continued problems with tuberculosis among Danes and Greenlanders in Denmark and the need for reinforced control--a systematic review]. Ugeskrift for laeger 2012, 174(44):2696-2701.

573. Lima J, Serejo F, Gloria H, Ramalho F, Moura MC: [Infection with hepatitis C virus in porphyria cutanea tarda]. Acta medica portuguesa 1996, 9(7-9):283-286.

574. Lin KW, Kirchner JT: Hepatitis B. Am Fam Physician 2004, 69(1):75-82.

575. Lindan CP, Lieu TX, Giang LT, Lap VD, Thuc NV, Thinh T, Lurie P, Mandel JS: Rising HIV infection rates in Ho Chi Minh City herald emerging AIDS epidemic in Vietnam. Aids 1997, 11 Suppl 1:S5-13.

576. Litwin AH, Berg KM, Li X, Hidalgo J, Arnsten JH: Rationale and design of a randomized controlled trial of directly observed hepatitis C treatment delivered in methadone clinics. BMC Infect Dis 2011, 11:315.

577. Llorent L, Richaud-Patin Y, Alcocer-Castillejos N, Ruiz-Soto R, Mercado MA, Orozco H, Gamboa-Dominguez A, Alcocer-Varela J: Cytokine gene expression in cirrhotic and non-cirrhotic human liver. J Hepatol 1996, 24(5):555-563.

578. Loftis JM, Matthews AM, Hauser P: Psychiatric and substance use disorders in individuals with hepatitis C: epidemiology and management. Drugs 2006, 66(2):155-174.

579. Longmire-Avital B, Golub SA, Parsons JT: Self-reevaluation as a critical component in sustained viral load change for HIV+ adults with alcohol problems. Ann Behav Med 2010, 40(2):176-183.

580. Lonnroth K, Williams BG, Stadlin S, Jaramillo E, Dye C: Alcohol use as a risk factor for tuberculosis - a systematic review. BMC Public Health 2008, 8:289.

581. Lopez-Contreras J, Ris J, Domingo P, Puig M, Martinez E: Tuberculous pulmonary gangrene: report of a case and review. Clin Infect Dis 1994, 18(2):243-245.

582. Lopez-Gomez M, Sampalo A, Mediavilla JD, Ortiz F, Corrales AJ, Martinez R, Jimenez-Alonso J: [Listeria monocytogenes infection in HIV patients: 2 new cases]. Revista clinica espanola 1993, 192(5):226-227.

583. Loria P, Marchesini G, Nascimbeni F, Ballestri S, Maurantonio M, Carubbi F, Ratziu V, Lonardo A: Cardiovascular risk, lipidemic phenotype and steatosis. A comparative analysis of cirrhotic and non-cirrhotic liver disease due to varying etiology. Atherosclerosis 2014, 232(1):99-109.

584. Losa JE, Miro JM, Del Rio A, Moreno-Camacho A, Garcia F, Claramonte X, Marco F, Mestres CA, Azqueta M, Gatell JM: Infective endocarditis not related to intravenous drug abuse in HIV-1-infected patients: report of eight cases and review of the literature. Clinical microbiology and infection : the official publication of the European Society of Clinical Microbiology and Infectious Diseases 2003, 9(1):45-54.

585. Louwagie GM, Ayo-Yusuf OA: Predictors of tobacco smoking abstinence among tuberculosis patients in South Africa. Journal of behavioral medicine 2015, 38(3):472-482.

586. Lozano Polo JL, Gutierrez Mora E, Martinez Perez V, Santamaria Gutierrez J, Vada Sanchez J, Vallejo Correas JA: [Effect of methadone or naltrexone on the course of transaminases in parenteral drug users with hepatitis C virus infection]. Revista clinica espanola 1997, 197(7):479-483.

587. Lucas GM, Weidle PJ, Hader S, Moore RD: Directly administered antiretroviral therapy in an urban methadone maintenance clinic: a nonrandomized comparative study. Clin Infect Dis 2004, 38 Suppl 5:S409-413.

588. Lucidarme D: [Hepatitis C and drug use: epidemiology, screening, natural history and treatment]. Gastroenterol Clin Biol 2002, 26 Spec No 2:B112-120.

589. Lucidarme D, Decoster A, Fremaux D, Harbonnier J, Jacob C, Vosgien V, Josse P, Villeger P, Henrio C, Prouvost-Keller B et al: Routine practice HCV infection screening with saliva samples: multicentric study in an intravenous drug user population. Gastroenterol Clin Biol 2007, 31(5):480-484.

590. Lucidarme D, Duburque C, Bulois P, Filoche B: Evolution of HCV incidence in drug users in France. Epidemiol Infect 2011, 139(9):1287-1295.

591. Luhmann N, Champagnat J, Golovin S, Maistat L, Agustian E, Inaridze I, Myint WM, Butsashvili M, Bouscaillou J: Access to hepatitis C treatment for people who inject drugs in low and middle income settings: Evidence from 5 countries in Eastern Europe and Asia. The International journal on drug policy 2015, 26(11):1081-1087.

592. Lund IO, Kirtadze I, Otiashvili D, O'Grady KE, Jones HE: Female partners of opioid-injecting men in the Republic of Georgia: an initial characterization. Substance abuse treatment, prevention, and policy 2012, 7:46.

593. Lunel F: [Hepatitis C virus: the virus responsible of the majority of non A non B hepatitis. 2: Epidemiology of Hepatitis C]. Gastroenterol Clin Biol 1992, 16(6-7):526-536.

594. Lutge EE, Wiysonge CS, Knight SE, Sinclair D, Volmink J: Incentives and enablers to improve adherence in tuberculosis. The Cochrane database of systematic reviews 2015(9):Cd007952.

595. Lutge EE, Wiysonge CS, Knight SE, Volmink J: Material incentives and enablers in the management of tuberculosis. The Cochrane database of systematic reviews 2012, 1:Cd007952.

596. Macalino GE, Hogan JW, Mitty JA, Bazerman LB, Delong AK, Loewenthal H, Caliendo AM, Flanigan TP: A randomized clinical trial of community-based directly observed therapy as an adherence intervention for HAART among substance users. Aids 2007, 21(11):1473-1477.

597. MacArthur GJ, van Velzen E, Palmateer N, Kimber J, Pharris A, Hope V, Taylor A, Roy K, Aspinall E, Goldberg D et al: Interventions to prevent HIV and Hepatitis C in people who inject drugs: a review of reviews to assess evidence of effectiveness. The International journal on drug policy 2014, 25(1):34-52.

598. Macchi B, Petruzzelli MA, De Laurenzi V: Biological aspects of HIV infection. La Clinica terapeutica 1992, 140(2):169-177.

599. MacGregor RR, Louria DB: Alcohol and infection. Current clinical topics in infectious diseases 1997, 17:291-315.

600. Machida K, Chen CL, Liu JC, Kashiwabara C, Feldman D, French SW, Sher L, Hyeongnam JJ, Tsukamoto H: Cancer stem cells generated by alcohol, diabetes, and hepatitis C virus. J Gastroenterol Hepatol 2012, 27 Suppl 2:19-22.

601. Mackay IR: Functional evaluation in liver disease: immunologic function. Laboratory and research methods in biology and medicine 1983, 7:89-101.

602. MacLellan J, Surey J, Abubakar I, Stagg HR, Mannell J: Using peer advocates to improve access to services among hard-to-reach populations with hepatitis C: a qualitative study of client and provider relationships. Harm Reduct J 2017, 14(1):76.

603. Madeddu A, Lucchesi M: [ALCOHOLISM AND TUBERCULOSIS. I. INTRODUCTORY CONSIDERATIONS]. Lotta contro la tubercolosi 1964, 34:713-728.

604. Maheshwari A, Kantsevoy S, Jagannath S, Thuluvath PJ: Endoscopic ultrasound and fine-needle aspiration for the diagnosis of hepatocellular carcinoma. Clinics in liver disease 2010, 14(2):325-332.

605. Malekinejad M, Navadeh S, Lotfizadeh A, Rahimi-Movaghar A, Amin-Esmaeili M, Noroozi A: High hepatitis C virus prevalence among drug users in Iran: systematic review and meta-analysis of epidemiological evidence (2001-2012). International journal of infectious diseases : IJID : official publication of the International Society for Infectious Diseases 2015, 40:116-130.

606. Malhi H, Kaufman RJ: Endoplasmic reticulum stress in liver disease. J Hepatol 2011, 54(4):795-809.

607. Malotte CK, Hollingshead JR, Larro M: Incentives vs outreach workers for latent tuberculosis treatment in drug users. Am J Prev Med 2001, 20(2):103-107.

608. Malotte CK, Hollingshead JR, Rhodes F: Monetary versus nonmonetary incentives for TB skin test reading among drug users. Am J Prev Med 1999, 16(3):182-188.

609. Malotte CK, Rhodes F, Mais KE: Tuberculosis screening and compliance with return for skin test reading among active drug users. Am J Public Health 1998, 88(5):792-796.

610. Manan MM, Ali SM, Khan MA, Jafarian S, Hameed MA: Review on the demographic and social impact of methadone-medication therapy on Malaysian patients. Pakistan journal of pharmaceutical sciences 2013, 26(4):841-846.

611. Mandayam S, Jamal MM, Morgan TR: Epidemiology of alcoholic liver disease. Seminars in liver disease 2004, 24(3):217-232.

612. March JC, Oviedo-Joekes E, Perea-Milla E, Carrasco F: Controlled trial of prescribed heroin in the treatment of opioid addiction. J Subst Abuse Treat 2006, 31(2):203-211.

613. Mardini H, Record C: Detection assessment and monitoring of hepatic fibrosis: biochemistry or biopsy? Annals of clinical biochemistry 2005, 42(Pt 6):441-447.

614. Marinho SF, Paciullo VH, Fonseca MO, Khoury Z, Yamin MA, Minkoves R, Andrade MO, Cavallari MM: [Persistent neutrophilic meningitis in a patient with the acquired immunodeficiency syndrome]. Revista da Sociedade Brasileira de Medicina Tropical 1997, 30(3):241-245.

615. Markin VA: [Estimation of the minimum infective doses of HIV in the prevalence of its infection]. Voprosy virusologii 2012, 57(1):4-8.

616. Marras TK, Daley CL: A systematic review of the clinical significance of pulmonary Mycobacterium kansasii isolates in HIV infection. J Acquir Immune Defic Syndr 2004, 36(4):883-889.

617. Marsano LS, Pena LR: The interaction of alcoholic liver disease and hepatitis C. Hepatogastroenterology 1998, 45(20):331-339.

618. Marschalko M, Ponyai K, Karpati S: [Sexually transmitted coinfections. HIV coinfections]. Orvosi hetilap 2015, 156(1):4-9.

619. Martin M, Vanichseni S, Suntharasamai P, Mock PA, van Griensven F, Pitisuttithum P, Tappero JW, Chiamwongpaet S, Sangkum U, Kitayaporn D et al: Drug use and the risk of HIV infection amongst injection drug users participating in an HIV vaccine trial in Bangkok, 1999-2003. The International journal on drug policy 2010, 21(4):296-301.

620. Martin M, Vanichseni S, Suntharasamai P, Sangkum U, Mock PA, Chaipung B, Worrajittanon D, Leethochawalit M, Chiamwongpaet S, Kittimunkong S et al: Factors associated with the uptake of and adherence to HIV pre-exposure prophylaxis in people who have injected drugs: an observational, open-label extension of the Bangkok Tenofovir Study. The lancet HIV 2017, 4(2):e59-e66.

621. Martin NK, Vickerman P, Dore GJ, Hickman M: The hepatitis C virus epidemics in key populations (including people who inject drugs, prisoners and MSM): the use of direct-acting antivirals as treatment for prevention. Curr Opin HIV AIDS 2015, 10(5):374-380.

622. Martin Suarez I, Aguayo Canela DM, Cordero Mendez F, Pujol de la Llave E: [Tuberculous liver abscesses as the form of presentation of human immunodeficiency virus infection]. Anales de medicina interna (Madrid, Spain : 1984) 1993, 10(3):123-126.

623. Martinello M, Hajarizadeh B, Grebely J, Dore GJ, Matthews GV: HCV Cure and Reinfection Among People With HIV/HCV Coinfection and People Who Inject Drugs. Curr HIV/AIDS Rep 2017, 14(3):110-121.

624. Martinello M, Matthews GV: Enhancing the detection and management of acute hepatitis C virus infection. The International journal on drug policy 2015, 26(10):899-910.

625. Martinez SM, Crespo G, Navasa M, Forns X: Noninvasive assessment of liver fibrosis. Hepatology 2011, 53(1):325-335.

626. Martinez-Esparza M, Tristan-Manzano M, Ruiz-Alcaraz AJ, Garcia-Penarrubia P: Inflammatory status in human hepatic cirrhosis. World J Gastroenterol 2015, 21(41):11522-11541.

627. Martinez-Rebollar M, Larrousse M, Calvo M, Munoz A, Gonzalez A, Lonca M, Martinez E, Blanco JL, Mallolas J, Laguno M: [Current status of acute hepatitis C]. Enferm Infecc Microbiol Clin 2011, 29(3):210-215.

628. Martin-Thormeyer EM, Paul RH: Drug abuse and hepatitis C infection as comorbid features of HIV associated neurocognitive disorder: neurocognitive and neuroimaging features. Neuropsychology review 2009, 19(2):215-231.

629. Martos JA, Olm M, Miro JM, Mallolas J, Letang E, Brancos MA, Gatell JM, Soriano E: [Chondrocostal and chondrosternal tuberculosis in 2 heroin addicts infected with the human immunodeficiency virus]. Medicina clinica 1989, 93(12):467-470.

630. Maru DS, Bruce RD, Walton M, Mezger JA, Springer SA, Shield D, Altice FL: Initiation, adherence, and retention in a randomized controlled trial of directly administered antiretroviral therapy. Aids Behav 2008, 12(2):284-293.

631. Maru DS, Bruce RD, Walton M, Springer SA, Altice FL: Persistence of virological benefits following directly administered antiretroviral therapy among drug users: results from a randomized controlled trial. J Acquir Immune Defic Syndr 2009, 50(2):176-181.

632. Maru DS, Kozal MJ, Bruce RD, Springer SA, Altice FL: Directly administered antiretroviral therapy for HIV-infected drug users does not have an impact on antiretroviral resistance: results from a randomized controlled trial. J Acquir Immune Defic Syndr 2007, 46(5):555-563.

633. Mathei C, Buntinx F, van Damme P: Seroprevalence of hepatitis C markers among intravenous drug users in western European countries: a systematic review. J Viral Hepat 2002, 9(3):157-173.

634. Mathei C, Robaeys G, Van Ranst M, Van Damme P, Buntinx F: The epidemiology of hepatitis C among injecting drug users in Belgium. Acta gastro-enterologica Belgica 2005, 68(1):50-54.

635. Mathurin P, Canva V, Dharancy S, Paris JC: [Treatment of chronic hepatitis C and alcohol consumption]. Gastroenterol Clin Biol 2002, 26 Spec No 2:B248-251.

636. Matthews G, Kronborg IJ, Dore GJ: Treatment for hepatitis C virus infection among current injection drug users in Australia. Clin Infect Dis 2005, 40 Suppl 5:S325-329.

637. Matthiessen W, Wundschock M, Radenbach KL, Gobel D, Jungbluth H, Kropp R: [A pilot and a controlled study of the influence of ethambutol on serum urate concentration and uric acid clearance (author's transl)]. Praxis und Klinik der Pneumologie 1979, 33(11):1109-1114.

638. Mattson RH, Cramer JA, Collins JF, Smith DB, Delgado-Escueta AV, Browne TR, Williamson PD, Treiman DM, McNamara JO, McCutchen CB et al: Comparison of carbamazepine, phenobarbital, phenytoin, and primidone in partial and secondarily generalized tonic-clonic seizures. N Engl J Med 1985, 313(3):145-151.

639. Mauss S, Berger F, Goelz J, Jacob B, Schmutz G: A prospective controlled study of interferon-based therapy of chronic hepatitis C in patients on methadone maintenance. Hepatology 2004, 40(1):120-124.

640. Mauss S, Klinker H: Drug-drug interactions in the treatment of HCV among people who inject drugs. Clin Infect Dis 2013, 57 Suppl 2:S125-128.

641. McCance-Katz EF: Treatment of opioid dependence and coinfection with HIV and hepatitis C virus in opioid-dependent patients: the importance of drug interactions between opioids and antiretroviral agents. Clin Infect Dis 2005, 41 Suppl 1:S89-95.

642. McCance-Katz EF, Rainey PM, Smith P, Morse GD, Friedland G, Boyarsky B, Gourevitch M, Jatlow P: Drug interactions between opioids and antiretroviral medications: interaction between methadone, LAAM, and delavirdine. Am J Addict 2006, 15(1):23-34.

643. McCoy CB, Metsch LR, Page JB, McBride DC, Comerford ST: Injection drug users' practices and attitudes toward intervention and potential for reducing the transmission of HIV. Med Anthropol 1997, 18(1):35-60.

644. McCoy CB, Shapshak P, Metsch LR, Rivers JE, McCoy HV, Weatherby NL, Shah SM, Chitwood DD: HIV-1 prevention: interdisciplinary studies on the efficacy of bleach and development of prevention protocols. Archivum immunologiae et therapiae experimentalis 1995, 43(1):1-9.

645. McCusker J, Bigelow C, Stoddard AM, Zorn M: Human immunodeficiency virus type 1 antibody status and changes in risk behavior among drug users. Annals of epidemiology 1994, 4(6):466-471.

646. McCusker J, Bigelow C, Zapka JG, Zorn M, Stoddard AM, Lewis BF: HIV-1 antibody testing among drug users participating in AIDS education. Patient education and counseling 1994, 24(3):267-278.

647. McElhiney M, Rabkin J, Van Gorp W, Rabkin R: Effect of armodafinil on cognition in patients with HIV/AIDS and fatigue. Journal of clinical and experimental neuropsychology 2013, 35(7):718-727.

648. McLaughlin S, Kind K, Thomson L, Bouhaidar R: Unexpected active tuberculosis on Post Mortem CT: A case report and review of the literature. Forensic science international 2016, 266:e64-e67.

649. McMahon JM, Tortu S: A potential hidden source of hepatitis C infection among noninjecting drug users. J Psychoactive Drugs 2003, 35(4):455-460.

650. McNicholas LF, Holbrook AM, O'Grady KE, Jones HE, Coyle MG, Martin PR, Heil SH, Stine SM, Kaltenbach K: Effect of hepatitis C virus status on liver enzymes in opioid-dependent pregnant women maintained on opioid-agonist medication. Addiction 2012, 107 Suppl 1:91-97.

651. Meemken L, Hanhoff N, Tseng A, Christensen S, Gillessen A: Drug-Drug Interactions With Antiviral Agents in People Who Inject Drugs Requiring Substitution Therapy. Ann Pharmacother 2015, 49(7):796-807.

652. Mehta SH, Thomas DL, Sulkowski MS, Safaein M, Vlahov D, Strathdee SA: A framework for understanding factors that affect access and utilization of treatment for hepatitis C virus infection among HCV-mono-infected and HIV/HCV-co-infected injection drug users. Aids 2005, 19 Suppl 3:S179-189.

653. Melin P, Schoeny M, Surget B, Bay V, Lepilleur B: [What are the expectations of patients with chronic hepatitis C and how can they be satisfied?]. Gastroenterol Clin Biol 2002, 26 Spec No 2:B291-296.

654. Mel'nik VP, Petrova IE, Dzerzhinskaia NA: [Stomach diseases in alcoholism patients with concomitant pulmonary tuberculosis]. Vrach Delo 1987(4):19-22.

655. Memon MI, Memon MA: Hepatitis C: an epidemiological review. J Viral Hepat 2002, 9(2):84-100.

656. Mendez-Sanchez N, Ridruejo E, Alves de Mattos A, Chavez-Tapia NC, Zapata R, Parana R, Mastai R, Strauss E, Guevara-Casallas LG, Daruich J et al: Latin American Association for the Study of the Liver (LAASL) clinical practice guidelines: management of hepatocellular carcinoma. Annals of hepatology 2014, 13 Suppl 1:S4-40.

657. Mendiburu Belzunegui L, Pena Garcia A, Sanchez Sanchez C, Font Cervero A: [An oral ulcer due to cytomegalovirus in a patient with the acquired immunodeficiency syndrome]. Anales de medicina interna (Madrid, Spain : 1984) 1999, 16(1):53.

658. Mercer DF: Animal models for studying hepatitis C and alcohol effects on liver. World J Gastroenterol 2011, 17(20):2515-2519.

659. Merchant RC, Baird JR, Liu T, Taylor LE, Montague BT, Nirenberg TD: Brief intervention to increase emergency department uptake of combined rapid human immunodeficiency virus and hepatitis C screening among a drug misusing population. Academic emergency medicine : official journal of the Society for Academic Emergency Medicine 2014, 21(7):752-767.

660. Merchant RC, DeLong AK, Liu T, Baird JR: Factors Influencing Uptake of Rapid HIV and Hepatitis C Screening Among Drug Misusing Adult Emergency Department Patients: Implications for Future HIV/HCV Screening Interventions. Aids Behav 2015, 19(11):2025-2035.

661. Merk HF: [Porphyria cutanea tara]. Der Hautarzt; Zeitschrift fur Dermatologie, Venerologie, und verwandte Gebiete 2016, 67(3):207-210.

662. Metsch LR, Feaster DJ, Gooden L, Matheson T, Stitzer M, Das M, Jain MK, Rodriguez AE, Armstrong WS, Lucas GM et al: Effect of Patient Navigation With or Without Financial Incentives on Viral Suppression Among Hospitalized Patients With HIV Infection and Substance Use: A Randomized Clinical Trial. Jama 2016, 316(2):156-170.

663. Michalowska-Mitczuk D: [Chronic sputum positive tuberculosis--an epidemiologic problem]. Pneumonologia i alergologia polska 1995, 63(1-2):106-109.

664. Michalowska-Mitczuk D, Gajda W, Kus J: [Anti-expectorant treatment of patients with tuberculosis and diabetes, liver and renal failure, during pregnancy and breast-feeding]. Pneumonologia i alergologia polska 1997, 65(3-4):270-277.

665. Michels, II, Fang YX, Zhao D, Zhao LY, Lu L: Comparison of drug abuse in Germany and China. Acta pharmacologica Sinica 2007, 28(10):1505-1518.

666. Micoud M: [Epidemiology of hepatitis C in 1997 in France]. La Revue de medecine interne 1997, 18 Suppl 2:60s-62s.

667. Midgard H, Weir A, Palmateer N, Lo Re V, 3rd, Pineda JA, Macias J, Dalgard O: HCV epidemiology in high-risk groups and the risk of reinfection. J Hepatol 2016, 65(1 Suppl):S33-s45.

668. Migasena S, Suntharasamai P, Pitisuttithum P, Kitayaporn D, Wasi C, Huang W, Vanichseni S, Koompong C, Kaewkungwal J, Raktham S et al: AIDSVAX (MN) in Bangkok injecting drug users: a report on safety and immunogenicity, including macrophage-tropic virus neutralization. AIDS research and human retroviruses 2000, 16(7):655-663.

669. Miguet JP, Mantion G: [Liver transplantation. A therapeutic revolution destined to change our attitude in hepato-gastroenterology]. Gastroenterol Clin Biol 1993, 17(2 Pt 3):T18-21.

670. Miller M, Mella I, Moi H, Eskild A: HIV and hepatitis C virus risk in new and longer-term injecting drug users in Oslo, Norway. J Acquir Immune Defic Syndr 2003, 33(3):373-379.

671. Miller M, Neaigus A: Networks, resources and risk among women who use drugs. Soc Sci Med 2001, 52(6):967-978.

672. Minder S, Fischler M, Muellhaupt B, Zalunardo MP, Jenni R, Clavien PA, Speich R: Intravenous iloprost bridging to orthotopic liver transplantation in portopulmonary hypertension. The European respiratory journal 2004, 24(4):703-707.

673. Miro JM, del Rio A, Mestres CA: Infective endocarditis in intravenous drug abusers and HIV-1 infected patients. Infectious disease clinics of North America 2002, 16(2):273-295, vii-viii.

674. Mitchell MC, Memisoglu A, Silverman BL: Hepatic safety of injectable extended-release naltrexone in patients with chronic hepatitis C and HIV infection. Journal of studies on alcohol and drugs 2012, 73(6):991-997.

675. Mitchell RS, Lester W: Clinical experience with cycloserine in the treatment of tuberculosis. Scandinavian journal of respiratory diseases Supplementum 1970, 71:94-108.

676. Mitrani VB, McCabe BE, Burns MJ, Feaster DJ: Family mechanisms of structural ecosystems therapy for HIV-seropositive women in drug recovery. Health psychology : official journal of the Division of Health Psychology, American Psychological Association 2012, 31(5):591-600.

677. Mitrani VB, Weiss-Laxer NS, Ow CE, Burns MJ, Ross-Russell S, Feaster DJ: Examining family networks of HIV+ women in drug recovery: challenges and opportunities. Families, systems & health : the journal of collaborative family healthcare 2009, 27(3):267-283.

678. Mitruka K, Oeltmann JE, Ijaz K, Haddad MB: Tuberculosis outbreak investigations in the United States, 2002-2008. Emerging infectious diseases 2011, 17(3):425-431.

679. Mohammad MK, Zhou Z, Cave M, Barve A, McClain CJ: Zinc and liver disease. Nutrition in clinical practice : official publication of the American Society for Parenteral and Enteral Nutrition 2012, 27(1):8-20.

680. Mohamoud YA, Miller FD, Abu-Raddad LJ: Potential for human immunodeficiency virus parenteral transmission in the Middle East and North Africa: an analysis using hepatitis C virus as a proxy biomarker. World J Gastroenterol 2014, 20(36):12734-12752.

681. Molina PE, Bagby GJ, Nelson S: Biomedical consequences of alcohol use disorders in the HIV-infected host. Curr HIV Res 2014, 12(4):265-275.

682. Monnat M, Forel P: [Hepatitis C and drug dependence]. Revue medicale de la Suisse romande 1998, 118(9):755-756.

683. Monto A, Currie S, Wright TL: Liver disease in injection drug users with hepatitis C, with and without HIV coinfection. J Addict Dis 2008, 27(2):49-59.

684. Monto A, Wright TL: The epidemiology and prevention of hepatocellular carcinoma. Seminars in oncology 2001, 28(5):441-449.

685. Mooney M, O'Brien F: Developing a plan of care using the Roper, Logan and Tierney model. British journal of nursing (Mark Allen Publishing) 2006, 15(16):887-892.

686. Morales MM, Llopis A, Ballester ML: [Epidemiologic study of tuberculous disease in the Hospital La Fe in Valencia]. Enferm Infecc Microbiol Clin 1994, 12(2):71-78.

687. Morgan TR, Brenner D, Everhart J, French SW, Fried MW, Gretch DR, Koziel MJ, McClain CJ, Peters MG, Weinman SA et al: Hepatitis C and alcohol: fundamental and translational research directions. Alcohol Clin Exp Res 2003, 27(4):726-731.

688. Morgan TR, Mandayam S, Jamal MM: Alcohol and hepatocellular carcinoma. Gastroenterology 2004, 127(5 Suppl 1):S87-96.

689. Mosley JW: Viral hepatitis: a group of epidemiologic entities. Canadian Medical Association journal 1972, 106:Suppl:427-434.

690. Moszczynski P, Lisiewicz J: [Hepatitis B and primary liver cancer]. Wiadomosci lekarskie (Warsaw, Poland : 1960) 1984, 37(24):1947-1953.

691. Moussalli J, Melin P, Wartelle-Bladou C, Lang JP: [Management of hepatitis C among drug user patients]. Gastroenterol Clin Biol 2007, 31(8-9 Pt 3):4s51-55.

692. Mudoni A, Caccetta F, Caroppo M, Musio F, Accogli A, Zacheo MD, Burzo MD, Nuzzo V: [Acute kidney injury and rhabdomyolysis after cocaine overdose: case report and literature review]. Giornale italiano di nefrologia : organo ufficiale della Societa italiana di nefrologia 2018, 35(2).

693. Mueller S, Millonig G, Seitz HK: Alcoholic liver disease and hepatitis C: a frequently underestimated combination. World J Gastroenterol 2009, 15(28):3462-3471.

694. Muga R, Guardiola H, Rey-Joly C: [Evaluation of drug addicts with associated pathology. Clinical and therapeutic aspects of the integral attention]. Medicina clinica 2004, 122(16):624-635.

695. Mumtaz GR, Weiss HA, Thomas SL, Riome S, Setayesh H, Riedner G, Semini I, Tawil O, Akala FA, Wilson D et al: HIV among people who inject drugs in the Middle East and North Africa: systematic review and data synthesis. PLoS medicine 2014, 11(6):e1001663.

696. Mumtaz GR, Weiss HA, Vickerman P, Larke N, Abu-Raddad LJ: Using hepatitis C prevalence to estimate HIV epidemic potential among people who inject drugs in the Middle East and North Africa. Aids 2015, 29(13):1701-1710.

697. Munoz-Perez MA, Rios-Martin JJ, Rodriguez-Pichardo A, Camacho F: Cutaneous T-cell lymphoma and human immunodeficiency virus infection: 2 cases and a review of the literature. Acta dermato-venereologica 1999, 79(2):153-155.

698. Murphy SM, Dweik D, McPherson S, Roll JM: Association between hepatitis C virus and opioid use while in buprenorphine treatment: preliminary findings. Am J Drug Alcohol Abuse 2015, 41(1):88-92.

699. Myers RP, Regimbeau C, Moussalli J, Ratziu V, Di Martino V, Thabut D, Bernard B, Benhamou Y, Poynard T: [What are the indications for treatment of acute hepatitis C?]. Gastroenterol Clin Biol 2002, 26 Spec No 2:B188-193.

700. Naggie S, Sulkowski MS: Management of patients coinfected with HCV and HIV: a close look at the role for direct-acting antivirals. Gastroenterology 2012, 142(6):1324-1334.e1323.

701. Naik S: HLA and gastrointestinal disorders. Indian journal of gastroenterology : official journal of the Indian Society of Gastroenterology 1986, 5(2):121-124.

702. Nair MP, Saiyed ZM: Effect of methamphetamine on expression of HIV coreceptors and CC-chemokines by dendritic cells. Life Sci 2011, 88(21-22):987-994.

703. Nair MP, Samikkannu T: Differential regulation of neurotoxin in HIV clades: role of cocaine and methamphetamine. Curr HIV Res 2012, 10(5):429-434.

704. Nalpas B: [Alcohol and hepatitis C virus: the return of hepatotropic viruses]. Gastroenterol Clin Biol 1994, 18(2):105-109.

705. Nalpas B, Driss F, Pol S, Hamelin B, Housset C, Brechot C, Berthelot P: Hepatitis C infection in alcoholics. Alcohol and alcoholism (Oxford, Oxfordshire) Supplement 1991, 1:321-322.

706. Nalpas B, Feitelson M, Brechot C, Rubin E: Alcohol, hepatotropic viruses, and hepatocellular carcinoma. Alcohol Clin Exp Res 1995, 19(5):1089-1095.

707. Nalpas B, Pol S, Thepot V, Zylberberg H, Berthelot P, Brechot C: ESBRA 1997 Award lecture: relationship between excessive alcohol drinking and viral infections. Alcohol and alcoholism (Oxford, Oxfordshire) 1998, 33(3):202-206.

708. Natangelo R, Monarca A, Bastia A, Adelasco L, Croce G: [HBsAg frequency in asymptomatic heroin users living in Milan (author's transl)]. Annali Sclavo; rivista di microbiologia e di immunologia 1980, 22(6):964-971.

709. Natangelo R, Vergani D, Libretti A: [Medcial complications in herion addicts]. Recenti progressi in medicina 1977, 63(4):411-422.

710. Nath A: Human immunodeficiency virus-associated neurocognitive disorder: pathophysiology in relation to drug addiction. Annals of the New York Academy of Sciences 2010, 1187:122-128.

711. Nath A, Hauser KF, Wojna V, Booze RM, Maragos W, Prendergast M, Cass W, Turchan JT: Molecular basis for interactions of HIV and drugs of abuse. J Acquir Immune Defic Syndr 2002, 31 Suppl 2:S62-69.

712. Nava-Aguilera E, Andersson N, Harris E, Mitchell S, Hamel C, Shea B, Lopez-Vidal Y, Villegas-Arrizon A, Morales-Perez A: Risk factors associated with recent transmission of tuberculosis: systematic review and meta-analysis. Int J Tuberc Lung Dis 2009, 13(1):17-26.

713. Navarro JF, Quereda C, Quereda C, Gallego N, Antela A, Mora C, Ortuno J: Nephrogenic diabetes insipidus and renal tubular acidosis secondary to foscarnet therapy. American journal of kidney diseases : the official journal of the National Kidney Foundation 1996, 27(3):431-434.

714. Nayha S, Jarvelin MR: Health trends in northern Finland. International journal of circumpolar health 1998, 57(2-3):94-103.

715. Neale J: Homelessness, drug use and hepatitis C: a complex problem explored within the context of social exclusion. The International journal on drug policy 2008, 19(6):429-435.

716. Nelson DR: The immunopathogenesis of hepatitis C virus infection. Clinics in liver disease 2001, 5(4):931-953.

717. Nelson PK, Mathers BM, Cowie B, Hagan H, Des Jarlais D, Horyniak D, Degenhardt L: Global epidemiology of hepatitis B and hepatitis C in people who inject drugs: results of systematic reviews. Lancet 2011, 378(9791):571-583.

718. Nelson S, Mason C, Bagby G, Summer W: Alcohol, tumor necrosis factor, and tuberculosis. Alcohol Clin Exp Res 1995, 19(1):17-24.

719. Nematollahi S, Ayubi E, Almasi-Hashiani A, Mansori K, Moradi Y, Veisani Y, Jenabi E, Gholamaliei B, Khazaei S: Prevalence of hepatitis C virus infection among high-risk groups in Iran: a systematic review and meta-analysis. Public Health 2018, 161:90-98.

720. Neri S, Bruno CM, Abate G, Ierna D, Mauceri B, Cilio D, Bordonaro F, Pulvirenti D, Italiano C, Caruso L: Controlled clinical trial to assess the response of recent heroin abusers with chronic hepatitis C virus infection to treatment with interferon alpha-n2b. Clin Ther 2002, 24(10):1627-1635.

721. Neri S, Pulvirenti D, Bertino G: Psychiatric symptoms induced by antiviral therapy in chronic hepatitis C: comparison between interferon-alpha-2a and interferon-alpha-2b. Clinical drug investigation 2006, 26(11):655-662.

722. Neuman M, Angulo P, Malkiewicz I, Jorgensen R, Shear N, Dickson ER, Haber J, Katz G, Lindor K: Tumor necrosis factor-alpha and transforming growth factor-beta reflect severity of liver damage in primary biliary cirrhosis. J Gastroenterol Hepatol 2002, 17(2):196-202.

723. Neuman MG, Sha K, Esguerra R, Zakhari S, Winkler RE, Hilzenrat N, Wyse J, Cooper CL, Seth D, Gorrell MD et al: Inflammation and repair in viral hepatitis C. Dig Dis Sci 2008, 53(6):1468-1487.

724. Newberne PM: Chemical carcinogenesis: mycotoxins and other chemicals to which humans are exposed. Seminars in liver disease 1984, 4(2):122-135.

725. Newmeyer J: Can HCV be prevented among injection drug users? J Psychoactive Drugs 2002, 34(4):421-425.

726. Ngo-Giang-Huong N, Jourdain G, Sirirungsi W, Decker L, Khamduang W, Le Coeur S, Sirinontakan S, Somsamai R, Pagdi K, Hemvuttiphan J et al: Human immunodeficiency virus-hepatitis C virus co-infection in pregnant women and perinatal transmission to infants in Thailand. International journal of infectious diseases : IJID : official publication of the International Society for Infectious Diseases 2010, 14(7):e602-607.

727. Nguyen-Khac E: [Acute hepatitis C in 2005]. Gastroenterol Clin Biol 2005, 29(11):1149-1156.

728. Nicolosi A, Villa M, Leite ML: The dynamics of human immunodeficiency virus type 1 transmission among injecting drug users. Journal of biological regulators and homeostatic agents 1997, 11(1-2):20-26.

729. Nikolopoulos GK, Kostaki EG, Paraskevis D: Overview of HIV molecular epidemiology among people who inject drugs in Europe and Asia. Infection, genetics and evolution : journal of molecular epidemiology and evolutionary genetics in infectious diseases 2016, 46:256-268.

730. Niveau G: Prevention of infectious disease transmission in correctional settings: a review. Public Health 2006, 120(1):33-41.

731. Nochy D, Druet P: [Glomerular lesions and hepatopathies]. Minerva medica 1978, 69(47):3243-3248.

732. Nolan CM: Community-wide implementation of targeted testing for and treatment of latent tuberculosis infection. Clin Infect Dis 1999, 29(4):880-887.

733. Nomura F: [New clinical laboratory tests for digestive system diseases]. Nihon Naika Gakkai zasshi The Journal of the Japanese Society of Internal Medicine 2001, 90(11):2213-2218.

734. Norman LR, Basso M, Kumar A, Malow R: Neuropsychological consequences of HIV and substance abuse: a literature review and implications for treatment and future research. Current drug abuse reviews 2009, 2(2):143-156.

735. North CS, Hong BA, Kerr T: Hepatitis C and substance use: new treatments and novel approaches. Curr Opin Psychiatry 2012, 25(3):206-212.

736. North CS, Sims O, Hong BA, Jain MK, Brown G, Lisker-Melman M, Pollio DE: An empirical study of alcohol consumption by patients considering HCV treatment. Am J Drug Alcohol Abuse 2014, 40(6):484-489.

737. Norton BL, Fleming J, Bachhuber MA, Steinman M, DeLuca J, Cunningham CO, Johnson N, Laraque F, Litwin AH: High HCV cure rates for people who use drugs treated with direct acting antiviral therapy at an urban primary care clinic. The International journal on drug policy 2017, 47:196-201.

738. Novick DM: The impact of hepatitis C virus infection on methadone maintenance treatment. The Mount Sinai journal of medicine, New York 2000, 67(5-6):437-443.

739. Novick DM, Kreek MJ: Critical issues in the treatment of hepatitis C virus infection in methadone maintenance patients. Addiction 2008, 103(6):905-918.

740. Novo-Veleiro I, Alvela-Suarez L, Chamorro AJ, Gonzalez-Sarmiento R, Laso FJ, Marcos M: Alcoholic liver disease and hepatitis C virus infection. World J Gastroenterol 2016, 22(4):1411-1420.

741. Novo-Veleiro I, Calle Cde L, Dominguez-Quiben S, Pastor I, Marcos M, Laso FJ: Prevalence of hepatitis C virus infection in alcoholic patients: cohort study and systematic review. Alcohol and alcoholism (Oxford, Oxfordshire) 2013, 48(5):564-569.

742. Nyamathi A, Nahid P, Berg J, Burrage J, Christiani A, Aqtash S, Morisky D, Leake B: Efficacy of nurse case-managed intervention for latent tuberculosis among homeless subsamples. Nursing research 2008, 57(1):33-39.

743. Nyamathi A, Salem BE, Marlow E, Zhang S, Yadav K: Understanding correlates of hepatitis C virus infection among homeless recently paroled men. Journal of forensic nursing 2013, 9(3):161-170.

744. Nyamathi AM, Christiani A, Windokun F, Jones T, Strehlow A, Shoptaw S: Hepatitis C virus infection, substance use and mental illness among homeless youth: a review. Aids 2005, 19 Suppl 3:S34-40.

745. Ocama P, Seremba E: Management of HIV and hepatitis C virus infections in resource-limited settings. Curr Opin HIV AIDS 2011, 6(6):539-545.

746. Ochalek TA, Heil SH, Higgins ST, Badger GJ, Sigmon SC: A novel mHealth application for improving HIV and Hepatitis C knowledge in individuals with opioid use disorder: A pilot study. Drug Alcohol Depend 2018, 190:224-228.

747. Ogbuagu O, Friedland G, Bruce RD: Drug interactions between buprenorphine, methadone and hepatitis C therapeutics. Expert opinion on drug metabolism & toxicology 2016, 12(7):721-731.

748. Oramasionwu CU, Moore HN, Toliver JC: Barriers to hepatitis C antiviral therapy in HIV/HCV co-infected patients in the United States: a review. AIDS Patient Care STDS 2014, 28(5):228-239.

749. Ortiz-Rey JA, Touza F, Perez-Valcarcel J, Perez-Villanueva J: [Oophoritis due to cytomegalovirus in a female AIDS patient]. Medicina clinica 1997, 108(9):357-358.

750. Osna NA: Hepatitis C virus and ethanol alter antigen presentation in liver cells. World J Gastroenterol 2009, 15(10):1201-1208.

751. Osna NA, Thomes PG, Donohue TM, Jr.: Involvement of autophagy in alcoholic liver injury and hepatitis C pathogenesis. World J Gastroenterol 2011, 17(20):2507-2514.

752. Otiashvili D, Piralishvili G, Sikharulidze Z, Kamkamidze G, Poole S, Woody GE: Methadone and buprenorphine-naloxone are effective in reducing illicit buprenorphine and other opioid use, and reducing HIV risk behavior--outcomes of a randomized trial. Drug Alcohol Depend 2013, 133(2):376-382.

753. Pache I, Moradpour D: [Highlights in hepatology]. Revue medicale suisse 2008, 4(141):214, 216, 218-220.

754. Page-Shafer K, Hahn JA, Lum PJ: Preventing hepatitis C virus infection in injection drug users: risk reduction is not enough. Aids 2007, 21(14):1967-1969.

755. Pais P: HIV and India: looking into the abyss. Tropical medicine & international health : TM & IH 1996, 1(3):295-304.

756. Palmateer N, Kimber J, Hickman M, Hutchinson S, Rhodes T, Goldberg D: Evidence for the effectiveness of sterile injecting equipment provision in preventing hepatitis C and human

immunodeficiency virus transmission among injecting drug users: a review of reviews. Addiction 2010, 105(5):844-859.

757. Palmateer NE, Hutchinson SJ, Innes H, Schnier C, Wu O, Goldberg DJ, Hickman M: Review and meta-analysis of the association between self-reported sharing of needles/syringes and hepatitis C virus prevalence and incidence among people who inject drugs in Europe. The International journal on drug policy 2013, 24(2):85-100.

758. Pana A, Mancinelli S, Palombi L: [Recent progress in research on AIDS (acquired immune deficiency syndrome)]. Recenti progressi in medicina 1983, 74(8):879-887.

759. Pandhare J, Dash C: A prospective on drug abuse-associated epigenetics and HIV-1 replication. Life Sci 2011, 88(21-22):995-999.

760. Panduro A, Maldonado-Gonzalez M, Fierro NA, Roman S: Distribution of HBV genotypes F and H in Mexico and Central America. Antiviral therapy 2013, 18(3 Pt B):475-484.

761. Paquette CE, Pollini RA: Injection drug use, HIV/HCV, and related services in nonurban areas of the United States: A systematic review. Drug Alcohol Depend 2018, 188:239-250.

762. Pares A, Caballeria J: [Hepatitis virus and alcoholic liver disease]. Gastroenterologia y hepatologia 1996, 19(7):383-389.

763. Pariente EA, Delvert D, Dhumeaux D: [Epidemiology of hepatitis C in France: from transfusion to drug addiction]. Gastroenterol Clin Biol 1994, 18(11):961-963.

764. Parikh N, Nonnemacher MR, Pirrone V, Block T, Mehta A, Wigdahl B: Substance abuse, HIV-1 and hepatitis. Curr HIV Res 2012, 10(7):557-571.

765. Parikh S, Hyman D: Hepatocellular cancer: a guide for the internist. The American journal of medicine 2007, 120(3):194-202.

766. Parkhomenko NA: [Biocorrection of biliary incompetence during digestive apparatus diseases]. Voprosy pitaniia 2009, 78(5):45-49.

767. Paronetto F: Cell-mediated immunity in liver disease. Human pathology 1986, 17(2):168-178.

768. Pasala S, Barr T, Messaoudi I: Impact of Alcohol Abuse on the Adaptive Immune System. Alcohol research : current reviews 2015, 37(2):185-197.

769. Passaro RC, Pandhare J, Qian HZ, Dash C: The Complex Interaction Between Methamphetamine Abuse and HIV-1 Pathogenesis. J Neuroimmune Pharmacol 2015, 10(3):477-486.

770. Paterson BL, Backmund M, Hirsch G, Yim C: The depiction of stigmatization in research about hepatitis C. The International journal on drug policy 2007, 18(5):364-373.

771. Patrick DM, Buxton JA, Bigham M, Mathias RG: Public health and hepatitis C. Canadian journal of public health = Revue canadienne de sante publique 2000, 91 Suppl 1:S18-21, s19-23.

772. Pectasides E, Miksad R, Pyatibrat S, Srivastava A, Bullock A: Spontaneous Regression of Hepatocellular Carcinoma with Multiple Lung Metastases: A Case Report and Review of the Literature. Dig Dis Sci 2016, 61(9):2749-2754.

773. Pellicano S, Foti N: [The epidemiology of non-A, non-B hepatitis (NANB). A review of the literature]. Minerva medica 1990, 81(3):157-168.

774. Peltzer K, Naidoo P, Louw J, Matseke G, Zuma K, McHunu G, Tutshana B, Mabaso M: Screening and brief interventions for hazardous and harmful alcohol use among patients with active tuberculosis attending primary public care clinics in South Africa: results from a cluster randomized controlled trial. BMC Public Health 2013, 13:699.

775. Peltzer KK, Naidoo PP, Matseke GG, Zuma KK: Screening and brief interventions for hazardous and harmful alcohol use among patients with active tuberculosis attending primary care clinics in South Africa: a cluster randomized controlled trial protocol. BMC Public Health 2011, 11:394.

776. Perarnau JM, Mrani-Alaoui MS, Magnin F, Sarkis M, Raabe JJ, Arbogast J: [Intrahepatic portasystemic shunt and hemorrhagic emergencies in the cirrhotic patient]. Annales de chirurgie 1995, 49(7):580-586; discussion 587-588.

777. Perlemuter G: [Hepatitis C and alcohol]. Gastroenterol Clin Biol 2002, 26 Spec No 2:B105-111.

778. Perlman DC, Jordan AE, McKnight C, Young C, Delucchi KL, Sorensen JL, Des Jarlais DC, Masson CL: Viral hepatitis among drug users in methadone maintenance: associated factors, vaccination outcomes, and interventions. J Addict Dis 2014, 33(4):322-331.

779. Perlman DC, Salomon N, Perkins MP, Yancovitz S, Paone D, Des Jarlais DC: Tuberculosis in drug users. Clin Infect Dis 1995, 21(5):1253-1264.

780. Persico M, Iolascon A: Steatosis as a co-factor in chronic liver diseases. World J Gastroenterol 2010, 16(10):1171-1176.

781. Peterson PK, Sharp BM, Gekker G, Jackson B, Balfour HH, Jr.: Opiates, human peripheral blood mononuclear cells, and HIV. Advances in experimental medicine and biology 1991, 288:171-178.

782. Phillips DF: Hepatitis. 2: the scientific advances. Hospitals 1971, 45(1):48-54.

783. Phillips TR, Billaud JN, Henriksen SJ: Methamphetamine and HIV-1: potential interactions and the use of the FIV/cat model. Journal of psychopharmacology (Oxford, England) 2000, 14(3):244-250.

784. Pinskii LL, Belianskii, II: [Effectiveness of complex containing antrale and tymohexine in the treatment of patients with chronic hepatitis]. Likars'ka sprava 2005(4):61-64.

785. Pintado V, Navas E, Moreno L, Moreno ME: [Necrotizing pneumonia due to Salmonella sp. complicated by pneumothorax in a patient infected with the human immunodeficiency virus]. Enferm Infecc Microbiol Clin 1999, 17(10):536-538.

786. Piot P, Goilav C, Kegels E: Hepatitis B: transmission by sexual contact and needle sharing. Vaccine 1990, 8 Suppl:S37-40; discussion S41-33.

787. Pitisuttithum P, Gilbert P, Gurwith M, Heyward W, Martin M, van Griensven F, Hu D, Tappero JW, Choopanya K: Randomized, double-blind, placebo-controlled efficacy trial of a bivalent recombinant glycoprotein 120 HIV-1 vaccine among injection drug users in Bangkok, Thailand. The Journal of infectious diseases 2006, 194(12):1661-1671.

788. Platt L, Minozzi S, Reed J, Vickerman P, Hagan H, French C, Jordan A, Degenhardt L, Hope V, Hutchinson S et al: Needle syringe programmes and opioid substitution therapy for preventing hepatitis C transmission in people who inject drugs. The Cochrane database of systematic reviews 2017, 9:Cd012021.

789. Plebani JG, Tirado CF, Pettinati HM, Kampman KM, Volpicelli JR, Oslin DW: Combined effects of alcohol and hepatitis C: a secondary analysis of alcohol use biomarkers and high-risk behaviors from two medication trials for alcohol dependence. Addict Behav 2010, 35(2):123-128.

790. Pomeranz SJ: Drug addiction: an update. Seminars in roentgenology 1983, 18(3):173-178.

791. Popper H: Hepatic cancers in man: quantitative perspectives. Environmental research 1979, 19(2):482-494.

792. Portilla J, Jorda P, Esteban J, Sanchez-Paya J, Merino E, Boix V: [Directly observed treatment of latent tuberculosis infection: comparative study of two isoniazid regimens]. Enferm Infecc Microbiol Clin 2003, 21(6):293-295.

793. Pott G, Gerlach U: [Progress in the diagnosis of chronic liver disease: diagnostic activity in liver fibrosis and cirrhosis]. Leber, Magen, Darm 1982, 12(4):141-144.

794. Pouget ER, Hagan H, Des Jarlais DC: Meta-analysis of hepatitis C seroconversion in relation to shared syringes and drug preparation equipment. Addiction 2012, 107(6):1057-1065.

795. Poynard T, Ratziu V, Bedossa P: Appropriateness of liver biopsy. Canadian journal of gastroenterology = Journal canadien de gastroenterologie 2000, 14(6):543-548.

796. Prakash O, Joshi BH, Zhang P, Aw TY, Teng S, Ali M, Shellito JE, Nelson S: Transgenic mouse model of ethanol as a cofactor in HIV disease. Alcohol Clin Exp Res 1998, 22(5 Suppl):266s-268s.

797. Prakash O, Mason A, Luftig RB, Bautista AP: Hepatitis C virus (HCV) and human immunodeficiency virus type 1 (HIV-1) infections in alcoholics. Frontiers in bioscience : a journal and virtual library 2002, 7:e286-300.

798. Preble OT, Sonnabend JA: AIDS from the perspective of experimental pathology. Journal of experimental pathology 1983, 1(1):3-6.

799. Price JC, Thio CL: Liver disease in the HIV-infected individual. Clin Gastroenterol Hepatol 2010, 8(12):1002-1012.

800. Prince MI, Hudson M: Liver transplantation for chronic liver disease: advances and controversies in an era of organ shortages. Postgrad Med J 2002, 78(917):135-141.

801. Privitera G, Spadaro L, Marchisello S, Fede G, Purrello F: Abnormalities of Lipoprotein Levels in Liver Cirrhosis: Clinical Relevance. Dig Dis Sci 2018, 63(1):16-26.

802. Pruett BD, Baddour LM: Sinopulmonary complications of illicit drug use. Infectious disease clinics of North America 2002, 16(3):623-643, viii.

803. Punzalan CS, Bukong TN, Szabo G: Alcoholic hepatitis and HCV interactions in the modulation of liver disease. J Viral Hepat 2015, 22(10):769-776.

804. Puoti M, Panzeri C, Rossotti R, Baiguera C: Efficacy of sofosbuvir-based therapies in HIV/HCV infected patients and persons who inject drugs. Dig Liver Dis 2014, 46 Suppl 5:S206-211.

805. Purcell DW, Garfein RS, Latka MH, Thiede H, Hudson S, Bonner S, Golub ET, Ouellet LJ: Development, description, and acceptability of a small-group, behavioral intervention to prevent HIV and hepatitis C virus infections among young adult injection drug users. Drug Alcohol Depend 2007, 91 Suppl 1:S73-80.

806. Purohit V, Rapaka RS, Shurtleff D: Mother-to-child transmission (MTCT) of HIV and drugs of abuse in post-highly active antiretroviral therapy (HAART) era. J Neuroimmune Pharmacol 2010, 5(4):507-515.

807. Puschel K: Drug-related death--an update. Forensic science international 1993, 62(1-2):121-128.

808. Quaglio G, Lugoboni F, Mezzelani P, Des Jarlais DC, Lechi A: Hepatitis vaccination among drug users. Vaccine 2006, 24(15):2702-2709.

809. Quan VM, Chung A, Long HT, Dondero TJ: HIV in Vietnam: the evolving epidemic and the prevention response, 1996 through 1999. J Acquir Immune Defic Syndr 2000, 25(4):360-369.

810. Quelhas R, Lopes A: Psychiatric problems in patients infected with hepatitis C before and during antiviral treatment with interferon-alpha: a review. Journal of psychiatric practice 2009, 15(4):262-281.

811. Querol JM, Farga A, Alonso C, Granda D, Alcaraz MJ, Garcia de Lomas J: [Applications of the polymerase chain reaction (PCR) to the diagnosis of central nervous system infections]. Anales de medicina interna (Madrid, Spain : 1984) 1996, 13(5):235-238.

812. Quintas S, do Carmo G, Gama R, Norberto A, Xavier R, Coutinho BS: [Lyell's syndrome in an AIDS patient]. Acta medica portuguesa 1997, 10(6-7):509-516.

813. Radley A, Tait J, Dillon JF: DOT-C: A cluster randomised feasibility trial evaluating directly observed anti-HCV therapy in a population receiving opioid substitute therapy from community pharmacy. The International journal on drug policy 2017, 47:126-136.

814. Rafiq SM, Banik GR, Khan S, Rashid H, Khandaker G: Current Burden of Hepatitis C Virus Infection Among Injecting Drug Users: A Mini Systematic Review of Prevalence Studies. Infectious disorders drug targets 2014, 14(2):93-100.

815. Rall CJ, Dienstag JL: Epidemiology of hepatitis C virus infection. Seminars in gastrointestinal disease 1995, 6(1):3-12.

816. Rambaldi A, Jacobs BP, Gluud C: Milk thistle for alcoholic and/or hepatitis B or C virus liver diseases. The Cochrane database of systematic reviews 2007(4):Cd003620.

817. Rambaldi A, Jacobs BP, Iaquinto G, Gluud C: Milk thistle for alcoholic and/or hepatitis B or C liver diseases--a systematic cochrane hepato-biliary group review with meta-analyses of randomized clinical trials. The American journal of gastroenterology 2005, 100(11):2583-2591.

818. Rambaldi A, Jacobs BP, Iaquinto G, Gluud C: Milk thistle for alcoholic and/or hepatitis B or C virus liver diseases. The Cochrane database of systematic reviews 2005(2):Cd003620.

819. Ramia S, Melhem NM, Kreidieh K: Hepatitis C virus infection in the Middle East and North Africa "MENA" region: injecting drug users (IDUs) is an under-investigated population. Infection 2012, 40(1):1-10.

820. Ramirez A, Vazquez-Sanchez AY, Carrion-Robalino N, Camacho J: Ion Channels and Oxidative Stress as a Potential Link for the Diagnosis or Treatment of Liver Diseases. Oxidative medicine and cellular longevity 2016, 2016:3928714.

821. Ratzmann KP: [Diabetes mellitus and liver diseases]. Z Arztl Fortbild (Jena) 1981, 75(4):169-174.

822. Realdi G, Tremolada F, Ruol A: [Non-A, non-B hepatitis]. Recenti progressi in medicina 1985, 76(3):158-178.

823. Reed JR, Jordan AE, Perlman DC, Smith DJ, Hagan H: The HCV care continuum among people who use drugs: protocol for a systematic review and meta-analysis. Systematic reviews 2016, 5(1):110.

824. Regev A, Jeffers LJ: Hepatitis C and alcohol. Alcohol Clin Exp Res 1999, 23(9):1543-1551.

825. Rehm J, Samokhvalov AV, Neuman MG, Room R, Parry C, Lonnroth K, Patra J, Poznyak V, Popova S: The association between alcohol use, alcohol use disorders and tuberculosis (TB). A systematic review. BMC Public Health 2009, 9:450.

826. Reimann HA: Infectious diseases: annual review of significant publications. Postgrad Med J 1971, 47(548):332-353.

827. Reimann HA: Infectious diseases: annual review of significant publications. Postgrad Med J 1973, 49(571):325-343.

828. Reimer J, Schmidt CS, Schulte B, Gansefort D, Golz J, Gerken G, Scherbaum N, Verthein U, Backmund M: Psychoeducation improves hepatitis C virus treatment during opioid substitution therapy: a controlled, prospective multicenter trial. Clin Infect Dis 2013, 57 Suppl 2:S97-104.

829. Reimer J, Schulte B, Castells X, Schafer I, Polywka S, Hedrich D, Wiessing L, Haasen C, Backmund M, Krausz M: Guidelines for the treatment of hepatitis C virus infection in injection drug users: status quo in the European Union countries. Clin Infect Dis 2005, 40 Suppl 5:S373-378.

830. Renner EL: [Does the primary disease recur following liver transplantation?]. Ther Umsch 1995, 52(9):572-580.

831. Renvillard SG, Leutscher PD, Hjerrild S, Videbech P: [Impaired cognitive function in hepatitis C virus infection]. Ugeskrift for laeger 2010, 172(5):372-376.

832. Reynolds HY: Pulmonary host defenses. Alcohol Clin Exp Res 1995, 19(1):6-10.

833. Rhoads J: Natural history and epidemiology of hepatitis C. J Assoc Nurses AIDS Care 2003, 14(5 Suppl):18s-25s.

834. Rhodes AG, Taxman FS, Friedmann PD, Cropsey KL: HCV in incarcerated populations: an analysis of gender and criminality on risk. J Psychoactive Drugs 2008, 40(4):493-501.

835. Rhodes T, Stimson GV, Fitch C, Ball A, Renton A: Rapid assessment, injecting drug use, and public health. Lancet 1999, 354(9172):65-68.

836. Rhodes T, Treloar C: The social production of hepatitis C risk among injecting drug users: a qualitative synthesis. Addiction 2008, 103(10):1593-1603.

837. Rich JD, Allen SA, Williams BA: The need for higher standards in correctional healthcare to improve public health. J Gen Intern Med 2015, 30(4):503-507.

838. Rich JD, Wolf FA, Macalino G: Strategies to improve access to sterile syringes for injection drug users. AIDS Read 2002, 12(12):527-535.

839. Rich ZC, Chu C, Mao J, Zhou K, Cai W, Ma Q, Volberding P, Tucker JD: Facilitators of HCV treatment adherence among people who inject drugs: a systematic qualitative review and implications for scale up of direct acting antivirals. BMC Public Health 2016, 16:994.

840. Richard V: [Hepatitis C epidemiology in the world]. Medecine tropicale : revue du Corps de sante colonial 1996, 56(4):393-399.

841. Ricks PM, Hershow RC, Rahimian A, Huo D, Johnson W, Prachand N, Jimenez A, Wiebel W, Paul W: A randomized trial comparing standard outcomes in two treatment models for substance users with tuberculosis. Int J Tuberc Lung Dis 2015, 19(3):326-332.

842. Ringertz O: Epidemiology of serum hepatitis. Clinics in gastroenterology 1974, 3(2):305-316.

843. Ritter A, Cameron J: A review of the efficacy and effectiveness of harm reduction strategies for alcohol, tobacco and illicit drugs. Drug Alcohol Rev 2006, 25(6):611-624.

844. Robaeys G, Buntinx F: Treatment of hepatitis C viral infections in substance abusers. Acta gastro-enterologica Belgica 2005, 68(1):55-67.

845. Robaeys G, Buntinx F, Bottieau E, Bourgeois S, Brenard R, Colle I, De Bie J, Mathei C, Mulkay JP, Van Damme P et al: Guidelines for the management of chronic hepatitis C in patients infected after substance use. Acta gastro-enterologica Belgica 2005, 68(1):38-45.

846. Robaeys G, Mathei C, Buntinx F, Vanranst M: Management of hepatitis C virus infections in intravenous drug users. Acta gastro-enterologica Belgica 2002, 65(2):99-100.

847. Robaeys G, Van Vlierberghe H, Mathei C, Van Ranst M, Bruckers L, Buntinx F: Similar compliance and effect of treatment in chronic hepatitis C resulting from intravenous drug use in comparison with other infection causes. Eur J Gastroenterol Hepatol 2006, 18(2):159-166.

848. Roblin X, Pofelski J, Zarski JP: [Steatosis, chronic hepatitis virus C infection and homocysteine]. Gastroenterol Clin Biol 2007, 31(4):415-420.

849. Robson SC, Brice E, van Rensburg C, Kannemeyer J, Hift RJ, Kirsch RE: Safety and efficacy of interferon alpha-2b following prednisone withdrawal in the treatment of chronic viral hepatitis B. A case-controlled, randomised study. South African medical journal = Suid-Afrikaanse tydskrif vir geneeskunde 1992, 82(5):317-320.

850. Rodella S, Picoco C: [Epidemiology of malignant liver tumors]. Annali dell'Istituto superiore di sanita 1996, 32(4):595-610.

851. Rodrigo JM, Serra MA, Aparisi L, Escudero A, Gilabert MS, Garcia F, Gonzalez R, del Olmo JA, Wassel AH, Artero A et al: Immune response to hepatitis B vaccine in parenteral drug abusers. Vaccine 1992, 10(11):798-801.

852. Rodriguez-Vidigal FF, Baz MJ, Fernandez FJ, Najarro F: [Hepatitis C virus infection in a first level rural hospital: descriptive study in the decade 1991-1999]. Enferm Infecc Microbiol Clin 2003, 21(3):142-146.

853. Rogers AI: Therapeutic considerations in selected forms of acute and chronic liver disease. The Medical clinics of North America 1971, 55(2):373-390.

854. Roman-Crossland R, Forrester L, Zaniewski G: Sex differences in injecting practices and hepatitis C: a systematic review of the literature. Canada communicable disease report = Releve des maladies transmissibles au Canada 2004, 30(14):125-132.

855. Romanelli F, Smith KM, Pomeroy C: Reducing the transmission of HIV-1: needle bleaching as a means of disinfection. Journal of the American Pharmaceutical Association (Washington,DC : 1996) 2000, 40(6):812-817.

856. Romero V, Azocar J, Zuniga J, Clavijo OP, Terreros D, Gu X, Husain Z, Chung RT, Amos C, Yunis EJ: Interaction of NK inhibitory receptor genes with HLA-C and MHC class II alleles in Hepatitis C virus infection outcome. Molecular immunology 2008, 45(9):2429-2436.

857. Roncero C, Littlewood R, Vega P, Martinez-Raga J, Torrens M: Chronic hepatitis C and individuals with a history of injecting drugs in Spain: population assessment, challenges for successful treatment. Eur J Gastroenterol Hepatol 2017, 29(6):629-633.

858. Roncero C, Villegas JL, Martinez-Rebollar M, Buti M: The pharmacological interactions between direct-acting antivirals for the treatment of chronic hepatitis c and psychotropic drugs. Expert review of clinical pharmacology 2018, 11(10):999-1030.

859. Root-Bernstein RS: Do we know the cause(s) of AIDS? Perspectives in biology and medicine 1990, 33(4):480-500.

860. Rosman AS, Basu P, Galvin K, Lieber CS: Efficacy of a high and accelerated dose of hepatitis B vaccine in alcoholic patients: a randomized clinical trial. The American journal of medicine 1997, 103(3):217-222.

861. Rosman AS, Waraich A, Galvin K, Casiano J, Paronetto F, Lieber CS: Alcoholism is associated with hepatitis C but not hepatitis B in an urban population. The American journal of gastroenterology 1996, 91(3):498-505.

862. Roudot-Thoraval F: [Modifications of epidemiological characteristics of hepatitis C]. Gastroenterol Clin Biol 2002, 26 Spec No 2:B138-143.

863. Roudot-Thoraval F, Monnet E, Mercet P, Bastie A, Dhumeaux D, Miguet JP: [Strategies of hepatitis C screening in general practice. Results of a two-center randomized trial]. Gastroenterol Clin Biol 2000, 24(11):1037-1041.

864. Roulot D, Vallet-Pichard A: [Natural history of factors influencing the severity of chronic HCV infection in HIV-HCV coinfected patients]. Gastroenterol Clin Biol 2007, 31(10):881-886.

865. Roux P, Rojas Castro D, Ndiaye K, Debrus M, Protopopescu C, Le Gall JM, Haas A, Mora M, Spire B, Suzan-Monti M et al: Increased Uptake of HCV Testing through a Community-Based Educational Intervention in Difficult-to-Reach People Who Inject Drugs: Results from the ANRS-AERLI Study. PLoS One 2016, 11(6):e0157062.

866. Rowe C, Santos GM, Raymond HF, Coffin PO: Social mixing and correlates of injection frequency among opioid use partnerships. The International journal on drug policy 2017, 41:80-88.

867. Rowe W, Rowe J, Malowaniec L: Hepatitis C: mental health issues. Canadian journal of public health = Revue canadienne de sante publique 2000, 91 Suppl 1:S42-44, s45-48.

868. Roy A, Abubakar I, Chapman A, Andrews N, Pattinson M, Lipman M, Rodrigues LC, Figueroa J, Tamne S, Catchpole M: A controlled trial of the knowledge impact of tuberculosis information leaflets among staff supporting substance misusers: pilot study. PLoS One 2011, 6(6):e20875.

869. Roy K, Hay G, Andragetti R, Taylor A, Goldberg D, Wiessing L: Monitoring hepatitis C virus infection among injecting drug users in the European Union: a review of the literature. Epidemiol Infect 2002, 129(3):577-585.

870. Royal W, 3rd: Neurologic disease in injection drug users: therapeutic issues. Journal of neurovirology 2000, 6 Suppl 1:S33-37.

871. Rubenstein LS, Amon JJ, McLemore M, Eba P, Dolan K, Lines R, Beyrer C: HIV, prisoners, and human rights. Lancet 2016, 388(10050):1202-1214.

872. Rudoi NM: [The problem of a combination of tuberculosis and alcoholism]. Zh Mikrobiol Epidemiol Immunobiol 1987(5):102-109.

873. Ruta S, Cernescu C: Injecting drug use: A vector for the introduction of new hepatitis C virus genotypes. World J Gastroenterol 2015, 21(38):10811-10823.

874. Rutherford GW, Schwarcz SK, McFarland W: Surveillance for incident HIV infection: new technology and new opportunities. J Acquir Immune Defic Syndr 2000, 25 Suppl 2:S115-119.

875. Ryder SD, Beckingham IJ: ABC of diseases of liver, pancreas, and biliary system: Acute hepatitis. Bmj 2001, 322(7279):151-153.

876. Rylance J, Meghji J, Miller RF, Ferrand RA: Global Considerations in Human Immunodeficiency Virus-Associated Respiratory Disease. Seminars in respiratory and critical care medicine 2016, 37(2):166-180.

877. Sacchi P, Cima S, Zuccaro V, Columpsi P, Sarda C, Mariani M, Puoti M, Bruno R: Understanding the Mechanisms of Fibrogenesis in HIV/HCV-Coinfected Patients: Implications for Clinical Practice. AIDS reviews 2015, 17(3):159-170.

878. Sacks-Davis R, Horyniak D, Grebely J, Hellard M: Behavioural interventions for preventing hepatitis C infection in people who inject drugs: a global systematic review. The International journal on drug policy 2012, 23(3):176-184.

879. Sagnelli E, Stroffolini T, Ascione A, Bonino F, Chiaramonte M, Colombo M, Craxi A, Manghisi OG, Pasquale G, Pastore G et al: The epidemiology of hepatitis delta infection in Italy over the last 18 years. Progress in clinical and biological research 1993, 382:287-294.

880. Saiz de la Hoya-Zamacola P, Marco-Mourino A, Clemente-Ricote G, Portilla-Sogorb J, Boix-Martinez V, Nunez-Martinez O, Reus-Banuls S, Teixido i Perez N: [Expert recommendations for the diagnosis and treatment of chronic hepatitis C infection in the prison setting]. Gastroenterologia y hepatologia 2006, 29(9):551-559.

881. Saiz de la Hoya-Zamacola P, Marco-Mourino A, Clemente-Ricote G, Portilla-Sogorb J, Boix-Martinez V, Nunez-Martinez O, Reus-Banuls S, Teixido i Perez N: [Expert recommendations for the diagnosis and treatment of chronic hepatitis C infection in the prison setting]. Enferm Infecc Microbiol Clin 2006, 24(9):568-575.

882. Sams H, Kiripolsky MG, Bhat L, Stricklin GP: Porphyria cutanea tarda, hepatitis C, alcoholism, and hemochromatosis: a case report and review of the literature. Cutis 2004, 73(3):188-190.

883. Sanchez Avila JF: [Hepatitis C and addictions]. Revista de gastroenterologia de Mexico 2002, 67 Suppl 2:S84-87.

884. Sanchez MA, Lemp GF, Magis-Rodriguez C, Bravo-Garcia E, Carter S, Ruiz JD: The epidemiology of HIV among Mexican migrants and recent immigrants in California and Mexico. J Acquir Immune Defic Syndr 2004, 37 Suppl 4:S204-214.

885. Sanchez Munoz-Torrero JF, Yniguez TR, Garcia-Onieva E, Pascua FJ, Crespo L, Bacaicoa A, Martin C: [Nocardiosis in patients with human immunodeficiency virus infection in Spain]. Revista clinica espanola 1995, 195(7):468-472.

886. Sanchez Tapias JM: [Hepatitis G]. Medicina clinica 1997, 109(8):311-316.

887. Sander G, Scandurra A, Kamenska A, MacNamara C, Kalpaki C, Bessa CF, Laso GN, Parisi G, Varley L, Wolny M et al: Overview of harm reduction in prisons in seven European countries. Harm Reduct J 2016, 13(1):28.

888. Santos J, Rivero A, Marquez M: [Acute pancreatitis with a fatal evolution due to antimonials in patients with visceral leishmaniasis and HIV infection]. Anales de medicina interna (Madrid, Spain : 1984) 2000, 17(10):562-563.

889. Sanyal AJ, Yoon SK, Lencioni R: The etiology of hepatocellular carcinoma and consequences for treatment. The oncologist 2010, 15 Suppl 4:14-22.

890. Sata M, Ishii K, Tanikawa K: [Hepatitis C virus infection in alcoholic patients with chronic liver disease]. Nihon rinsho Japanese journal of clinical medicine 1995, 53 Suppl(Pt 1):852-857.

891. Scarpelli DG: Human liver carcinogenesis: a complex problem. Monographs in pathology 1985(26):168-189.

892. Schackman BR, Leff JA, Barter DM, DiLorenzo MA, Feaster DJ, Metsch LR, Freedberg KA, Linas BP: Cost-effectiveness of rapid hepatitis C virus (HCV) testing and simultaneous rapid HCV and HIV testing in substance abuse treatment programs. Addiction 2015, 110(1):129-143.

893. Schaefer M, Heinz A, Backmund M: Treatment of chronic hepatitis C in patients with drug dependence: time to change the rules? Addiction 2004, 99(9):1167-1175.

894. Schaefer M, Hinzpeter A, Mohmand A, Janssen G, Pich M, Schwaiger M, Sarkar R, Friebe A, Heinz A, Kluschke M et al: Hepatitis C treatment in "difficult-to-treat" psychiatric patients with pegylated interferon-alpha and ribavirin: response and psychiatric side effects. Hepatology 2007, 46(4):991-998.

895. Schaefer M, Mauss S: Hepatitis C treatment in patients with drug addiction: clinical management of interferon-alpha-associated psychiatric side effects. Current drug abuse reviews 2008, 1(2):177-187.

896. Schaefer M, Sarkar R, Diez-Quevedo C: Management of mental health problems prior to and during treatment of hepatitis C virus infection in patients with drug addiction. Clin Infect Dis 2013, 57 Suppl 2:S111-117.

897. Schattenberg JM, Galle PR, Schuchmann M: [Acute liver failure--medical viewpoints]. Praxis (Bern 1994) 2006, 95(48):1873-1877.

898. Scheft H, Fontenette DC: Psychiatric barriers to readiness for treatment for hepatitis C Virus (HCV) infection among injection drug users: clinical experience of an addiction psychiatrist in the HIV-HCV coinfection clinic of a public health hospital. Clin Infect Dis 2005, 40 Suppl 5:S292-296.

899. Scheinmann R, Hagan H, Lelutiu-Weinberger C, Stern R, Des Jarlais DC, Flom PL, Strauss S: Non-injection drug use and Hepatitis C Virus: a systematic review. Drug Alcohol Depend 2007, 89(1):1-12.

900. Schiff ER: Hepatitis C and alcohol. Hepatology 1997, 26(3 Suppl 1):39s-42s.

901. Schiff ER, Ozden N: Hepatitis C and alcohol. Alcohol research & health : the journal of the National Institute on Alcohol Abuse and Alcoholism 2003, 27(3):232-239.

902. Schluger NW, El-Bassel N, Hermosilla S, Terlikbayeva A, Darisheva M, Aifah A, Galea S: Tuberculosis, drug use and HIV infection in Central Asia: an urgent need for attention. Drug Alcohol Depend 2013, 132 Suppl 1:S32-36.

903. Schmiegel WH: [Diagnosis and treatment of acute and chronic hepatitis C--procedure in difficult situations]. Zeitschrift fur Gastroenterologie 2004, 42(8):720-723.

904. Schneider RF, Rosen MJ: Respiratory infections in patients with HIV infection. Current opinion in pulmonary medicine 1996, 2(3):246-252.

905. Schnell GA, Schubert TT: Delta hepatitis: first two cases identified in Kansas City. South Med J 1988, 81(8):952-955.

906. Schoeman JH, Parry CD, Lombard CJ, Klopper HJ: Assessment of alcohol-screening instruments in tuberculosis patients. Tuber Lung Dis 1994, 75(5):371-376.

907. Schreier E, Roggendorf M, Driesel G, Hohne M, Viazov S: Genotypes of hepatitis C virus isolates from different parts of the world. Archives of virology Supplementum 1996, 11:185-193.

908. Schuch-Goi SB, Scherer JN, Kessler FHP, Sordi AO, Pechansky F, von Diemen L: Hepatitis C: clinical and biological features related to different forms of cocaine use. Trends in psychiatry and psychotherapy 2017, 39(4):285-292.

909. Schulte B, Schutt S, Brack J, Isernhagen K, Deibler P, Dilg C, Verthein U, Haasen C, Reimer J: Successful treatment of chronic hepatitis C virus infection in severely opioid-dependent patients under heroin maintenance. Drug Alcohol Depend 2010, 109(1-3):248-251.

910. Schulte B, Stover H, Leicht A, Schnackenberg K, Reimer J: [Prevention of hepatitis C virus infection in drug users]. Bundesgesundheitsblatt, Gesundheitsforschung, Gesundheitsschutz 2008, 51(10):1210-1217.

911. Schulte M, Hser Y, Saxon A, Evans E, Li L, Huang D, Hillhouse M, Thomas C, Ling W: Risk Factors Associated with HCV Among Opioid-Dependent Patients in a Multisite Study. J Community Health 2015, 40(5):940-947.

912. Schwartz RH: Syringe and needle exchange programs: Part I. South Med J 1993, 86(3):318-322.

913. Scott JD, Garland N: Chronic liver disease in Aboriginal North Americans. World J Gastroenterol 2008, 14(29):4607-4615.

914. Scott N, Hellard M, McBryde ES: Modeling hepatitis C virus transmission among people who inject drugs: Assumptions, limitations and future challenges. Virulence 2016, 7(2):201-208.

915. Seeff LB: Hepatitis in the drug abuser. The Medical clinics of North America 1975, 59(4):843-848.

916. Seeff LB, Hoofnagle JH: Appendix: The National Institutes of Health Consensus Development Conference Management of Hepatitis C 2002. Clinics in liver disease 2003, 7(1):261-287.

917. Semaan S, Neumann MS, Hutchins K, D'Anna LH, Kamb ML: Brief counseling for reducing sexual risk and bacterial STIs among drug users--results from project RESPECT. Drug Alcohol Depend 2010, 106(1):7-15.

918. Semba RD, Ricketts EP, Mehta S, Netski D, Thomas D, Kirk G, Wu AW, Vlahov D: Effect of micronutrients and iron supplementation on hemoglobin, iron status, and plasma hepatitis C and HIV RNA levels in female injection drug users: a controlled clinical trial. J Acquir Immune Defic Syndr 2007, 45(3):298-303.

919. Semba RD, Ricketts EP, Mehta SF, Kirk GD, Latkin C, Galai N, Vlahov D: Adherence and retention of female injection drug users in a phase III clinical trial in inner city Baltimore. Am J Drug Alcohol Abuse 2007, 33(1):71-80.

920. Semba RD, Shah N, Vlahov D: Improvement of anemia among HIV-infected injection drug users receiving highly active antiretroviral therapy. J Acquir Immune Defic Syndr 2001, 26(4):315-319.

921. Sereno L, Mesquita F, Kato M, Jacka D, Nguyen TT, Nguyen TN: Epidemiology, responses, and way forward: the silent epidemic of viral hepatitis and HIV coinfection in Vietnam. Journal of the International Association of Physicians in AIDS Care (Chicago, Ill : 2002) 2012, 11(5):311-320.

922. Serfaty L: [Non-transfusional and non-intravenous drug addiction related transmission of hepatitis C virus]. Presse Med 1999, 28(21):1135-1140.

923. Serfaty L, Mavier P, Valla D: [What are the non transfusional modes of transmission of hepatitis C virus?]. Gastroenterol Clin Biol 1995, 19(5):525-533.

924. Seronello S, Sheikh MY, Choi J: Redox regulation of hepatitis C in nonalcoholic and alcoholic liver. Free radical biology & medicine 2007, 43(6):869-882.

925. Serov VV, Sekamova SM, Tanashchuk EL: [Role of chronic viral hepatitis in the development of hepatocellular carcinoma]. Arkhiv patologii 2002, 64(5):60-64.

926. Shapshak P, Crandall KA, Xin KQ, Goodkin K, Fujimura RK, Bradley W, McCoy CB, Nagano I, Yoshioka M, Petito C et al: HIV-1 neuropathogenesis and abused drugs: current reviews, problems, and solutions. Advances in experimental medicine and biology 1996, 402:171-186.

927. Shapshak P, Somboonwit C, Drumright LN, Frost SD, Commins D, Tellinghuisen TL, Scott WK, Duncan R, McCoy C, Page JB et al: Molecular and contextual markers of hepatitis C virus and drug abuse. Molecular diagnosis & therapy 2009, 13(3):153-179.

928. Shardell M, Scharfstein DO, Vlahov D, Galai N: Sensitivity analysis using elicited expert information for inference with coarsened data: illustration of censored discrete event times in the AIDS Link to Intravenous Experience (ALIVE) Study. Am J Epidemiol 2008, 168(12):1460-1469.

929. Shawa IT, Felmlee DJ, Hegazy D, Sheridan DA, Cramp ME: Exploration of potential mechanisms of hepatitis C virus resistance in exposed uninfected intravenous drug users. J Viral Hepat 2017, 24(12):1082-1088.

930. Shelat VG, Diddapur RK: An early experience of liver transplantation in portal vein thrombosis. Singapore medical journal 2008, 49(2):e37-41.

931. Shepard CW, Finelli L, Alter MJ: Global epidemiology of hepatitis C virus infection. The Lancet Infectious diseases 2005, 5(9):558-567.

932. Sherlock DS: Chronic hepatitis C. Disease-a-month : DM 1994, 40(3):117-196.

933. Sherlock S: Chronic hepatitis. Postgraduate medicine 1979, 65(6):81-85, 87-88.

934. Sherlock S: The future of hepatology. The Netherlands journal of medicine 1984, 27(7):262-267.

935. Sherlock S: Alcoholic liver disease. Lancet 1995, 345(8944):227-229.

936. Sherman KE: Implications of peginterferon use in special populations infected with HCV. Seminars in liver disease 2003, 23 Suppl 1:47-52.

937. Sherman KE: New paradigms in the management of hepatitis C virus co-infections. Nature clinical practice Gastroenterology & hepatology 2007, 4 Suppl 1:S10-16.

938. Sherstiuk BV, Pigolkin Iu I: [Current problems in the morphological diagnosis of somatic disorders in drug addictions]. Sudebno-meditsinskaia ekspertiza 1999, 42(2):29-32.

939. Shiffman ML: Retreatment of patients who do not respond to initial therapy for chronic hepatitis C. Cleve Clin J Med 2004, 71 Suppl 3:S13-16.

940. Shih JW, Mur JI, Alter HJ: Non-A, non-B hepatitis: advances and unfulfilled expectations of the first decade. Progress in liver diseases 1986, 8:433-452.

941. Shin M, Chang SH: De Novo Hepatitis B Infection From Hepatitis B Core Antibody-Positive Donors During Hepatitis B Immunoglobulin Prophylaxis. Experimental and clinical transplantation : official journal of the Middle East Society for Organ Transplantation 2016, 14(1):106-108.

942. Shin S, Livchits V, Connery HS, Shields A, Yanov S, Yanova G, Fitzmaurice GM, Nelson AK, Greenfield SF: Effectiveness of alcohol treatment interventions integrated into routine tuberculosis care in Tomsk, Russia. Addiction 2013, 108(8):1387-1396.

943. Shin SS, Livchits V, Nelson AK, Lastimoso CS, Yanova GV, Yanov SA, Mishustin SP, Connery HS, Greenfield SF: Implementing evidence-based alcohol interventions in a resource-limited setting: novel delivery strategies in Tomsk, Russia. Harvard review of psychiatry 2012, 20(1):58-67.

944. Shoreibah M, Anand BS, Singal AK: Alcoholic hepatitis and concomitant hepatitis C virus infection. World J Gastroenterol 2014, 20(34):11929-11934.

945. Shor-Posner G, Lecusay R, Miguez MJ, Moreno-Black G, Zhang G, Rodriguez N, Burbano X, Baum M, Wilkie F: Psychological burden in the era of HAART: impact of selenium therapy. Int J Psychiatry Med 2003, 33(1):55-69.

946. Shorrock C, Neuberger J: The changing face of liver transplantation. Gut 1993, 34(3):295-298.

947. Siddiqi K, Khan A, Ahmad M, Shafiq ur R: An intervention to stop smoking among patients suspected of TB--evaluation of an integrated approach. BMC Public Health 2010, 10:160.

948. Silva AS, Santos LL, Passos AD, Sankarankutty AK, Martinelli Ade L, Silva Ode C: Chronic liver disease prevention strategies and liver transplantation. Acta cirurgica brasileira 2006, 21 Suppl 1:79-84.

949. Silva DR, Munoz-Torrico M, Duarte R, Galvao T, Bonini EH, Arbex FF, Arbex MA, Augusto VM, Rabahi MF, Mello FCQ: Risk factors for tuberculosis: diabetes, smoking, alcohol use, and the use of other drugs. Jornal brasileiro de pneumologia : publicacao oficial da Sociedade Brasileira de Pneumologia e Tisilogia 2018, 44(2):145-152.

950. Silverman BC, Kim AY, Freudenreich O: Interferon-induced psychosis as a "psychiatric contraindication" to hepatitis C treatment: a review and case-based discussion. Psychosomatics 2010, 51(1):1-7.

951. Silverstein PS, Kumar S, Kumar A: HIV-1, HCV and alcohol in the CNS: potential interactions and effects on neuroinflammation. Curr HIV Res 2014, 12(4):282-292.

952. Silverstein PS, Shah A, Weemhoff J, Kumar S, Singh DP, Kumar A: HIV-1 gp120 and drugs of abuse: interactions in the central nervous system. Curr HIV Res 2012, 10(5):369-383.

953. Simet SM, Sisson JH: Alcohol's Effects on Lung Health and Immunity. Alcohol research : current reviews 2015, 37(2):199-208.

954. Singal AK, Anand BS: Mechanisms of synergy between alcohol and hepatitis C virus. J Clin Gastroenterol 2007, 41(8):761-772.

955. Singal AK, Jampana SC, Weinman SA: Antioxidants as therapeutic agents for liver disease. Liver Int 2011, 31(10):1432-1448.

956. Singh S, Muir AJ, Dieterich DT, Falck-Ytter YT: American Gastroenterological Association Institute Technical Review on the Role of Elastography in Chronic Liver Diseases. Gastroenterology 2017, 152(6):1544-1577.

957. Singhal A, Jabbour N: Hepatocellular carcinoma: status in the era of liver transplantation. J Okla State Med Assoc 2009, 102(12):355-361.

958. Siu L, Foont J, Wands JR: Hepatitis C virus and alcohol. Seminars in liver disease 2009, 29(2):188-199.

959. Smith BD, Jorgensen C, Zibbell JE, Beckett GA: Centers for Disease Control and Prevention initiatives to prevent hepatitis C virus infection: a selective update. Clin Infect Dis 2012, 55 Suppl 1:S49-53.

960. Smith DJ, Combellick J, Jordan AE, Hagan H: Hepatitis C virus (HCV) disease progression in people who inject drugs (PWID): A systematic review and meta-analysis. The International journal on drug policy 2015, 26(10):911-921.

961. Smith DJ, Jordan AE, Frank M, Hagan H: Spontaneous viral clearance of hepatitis C virus (HCV) infection among people who inject drugs (PWID) and HIV-positive men who have sex with men (HIV+ MSM): a systematic review and meta-analysis. BMC Infect Dis 2016, 16:471.

962. Smith-Rohrberg D, Mezger J, Walton M, Bruce RD, Altice FL: Impact of enhanced services on virologic outcomes in a directly administered antiretroviral therapy trial for HIV-infected drug users. J Acquir Immune Defic Syndr 2006, 43 Suppl 1:S48-53.

963. Snow KJ, Young JT, Preen DB, Lennox NG, Kinner SA: Incidence and correlates of hepatitis C virus infection in a large cohort of prisoners who have injected drugs. BMC Public Health 2014, 14:830.

964. Sockalingam S, Shammi C, Stergiopoulos V: Managing the neuropsychiatric complications of hepatitis C treatment. British journal of hospital medicine (London, England : 2005) 2007, 68(10):520-525.

965. Sokolova GB, Ivleva A: [Polypharmacy problems in the treatment of tuberculosis and alcoholism (a review of the literature)]. Probl Tuberk 1980(8):24-31.

966. Sola E, Fernandez J, Gines P: Acute-on-Chronic Liver Failure: The Role of Precipitating Illness. Seminars in liver disease 2016, 36(2):117-122.

967. Solomon SS, Sulkowski MS, Amrose P, Srikrishnan AK, McFall AM, Ramasamy B, Kumar MS, Anand S, Thomas DL, Mehta SH: Directly observed therapy of sofosbuvir/ribavirin +/- peginterferon with minimal monitoring for the treatment of chronic hepatitis C in people with a history of drug use in Chennai, India (C-DOT). J Viral Hepat 2018, 25(1):37-46.

968. Song Z, Joshi-Barve S, Barve S, McClain CJ: Advances in alcoholic liver disease. Current gastroenterology reports 2004, 6(1):71-76.

969. Soontornniyomkij V, Kesby JP, Morgan EE, Bischoff-Grethe A, Minassian A, Brown GG, Grant I: Effects of HIV and Methamphetamine on Brain and Behavior: Evidence from Human Studies and Animal Models. J Neuroimmune Pharmacol 2016, 11(3):495-510.

970. Soriano V, Leal M: [Strategies for the prevention of human immunodeficiency virus infection]. Revista clinica espanola 1995, 195(10):701-707.

971. Soriano V, Nedjar S, Garcia-Samaniego J, Bravo R, Castro A, Gonzalez J, Martinez-Odriozola P, Pedreira J, del Romero J, Suarez D et al: High rate of co-infection with different hepatitis C virus subtypes in HIV-infected intravenous drug addicts in Spain. Hepatitis HIV Spanish Study Group. J Hepatol 1995, 22(5):598-599.

972. Spaulding AC, Weinbaum CM, Lau DT, Sterling R, Seeff LB, Margolis HS, Hoofnagle JH: A framework for management of hepatitis C in prisons. Ann Intern Med 2006, 144(10):762-769.

973. Spengler U, Fischer HP: [Liver biopsy at the intersection of clinical and pathological diagnosis]. Der Pathologe 2008, 29(1):6-14.

974. Sporea I, Sirli RL: Hepatic elastography for the assessment of liver fibrosis--present and future. Ultraschall in der Medizin (Stuttgart, Germany : 1980) 2012, 33(6):550-558.

975. Springer SA, Chen S, Altice F: Depression and symptomatic response among HIV-infected drug users enrolled in a randomized controlled trial of directly administered antiretroviral therapy. AIDS Care 2009, 21(8):976-983.

976. Stapinski A: [HIV infection--a sexually transmitted disease]. Przeglad dermatologiczny 1989, 76(4):274-284.

977. Stark K, Kleiber D: [AIDS and HIV infection in intravenous drug addicts in the Federal Republic of Germany]. Deutsche medizinische Wochenschrift (1946) 1991, 116(22):863-869.

978. Stefenelli N: [Internistic complications of chronic alcoholism]. Wiener medizinische Wochenschrift (1946) 1977, 127(18):565-569.

979. Steffen T, Gutzwiller F: [Hepatitis B and C in intravenous drug abusers in Switzerland]. Praxis (Bern 1994) 1999, 88(47):1937-1944.

980. Stein K, Dalziel K, Walker A, McIntyre L, Jenkins B, Horne J, Royle P, Round A: Screening for hepatitis C among injecting drug users and in genitourinary medicine clinics: systematic reviews of effectiveness, modelling study and national survey of current practice. Health technology assessment (Winchester, England) 2002, 6(31):1-122.

981. Stein MD, Herman DS, Anderson BJ: A trial to reduce hepatitis C seroincidence in drug users. J Addict Dis 2009, 28(4):389-398.

982. Stern RK, Hagan H, Lelutiu-Weinberger C, Des Jarlais D, Scheinmann R, Strauss S, Pouget ER, Flom P: The HCV Synthesis Project: scope, methodology, and preliminary results. BMC medical research methodology 2008, 8:62.

983. Sterneck M: [Etiology of hepatocellular carcinoma. The problem of the primary disease]. Der Internist 2000, 41(2 Pt 2):185-190.

984. Stevens CE: Hepatitis D virus and other chronic viral infections in high risk populations: interaction from an epidemiologic perspective. Progress in clinical and biological research 1991, 364:361-364.

985. Stockheim JA, Daaboul JJ, Yogev R, Scully SP, Binns HJ, Chadwick EG: Adrenal suppression in children with the human immunodeficiency virus treated with megestrol acetate. The Journal of pediatrics 1999, 134(3):368-370.

986. Stoiber H, Kacani L, Speth C, Wurzner R, Dierich MP: The supportive role of complement in HIV pathogenesis. Immunological reviews 2001, 180:168-176.

987. Stone KA: Reviewing harm reduction for people who inject drugs in Asia: the necessity for growth. Harm Reduct J 2015, 12:32.

988. Stoneburner RL, Chiasson MA, Weisfuse IB, Thomas PA: The epidemic of AIDS and HIV-1 infection among heterosexuals in New York City. Aids 1990, 4(2):99-106.

989. Stoner SA, Norris J, George WH, Morrison DM, Zawacki T, Davis KC, Hessler DM: Women's condom use assertiveness and sexual risk-taking: effects of alcohol intoxication and adult victimization. Addict Behav 2008, 33(9):1167-1176.

990. Strader DB: Coinfection with HIV and hepatitis C virus in injection drug users and minority populations. Clin Infect Dis 2005, 41 Suppl 1:S7-13.

991. Strathdee SA, Patterson TL: Behavioral interventions for HIV-positive and HCV-positive drug users. Aids Behav 2006, 10(2):115-130.

992. Strathdee SA, Shoptaw S, Dyer TP, Quan VM, Aramrattana A: Towards combination HIV prevention for injection drug users: addressing addictophobia, apathy and inattention. Curr Opin HIV AIDS 2012, 7(4):320-325.

993. Stroffolini T: Etiological factor of hepatocellular carcinoma in Italy. Minerva gastroenterologica e dietologica 2005, 51(1):1-5.

994. Su CW, Yang YY, Lin HC: Impact of etiological treatment on prognosis. Hepatology international 2018, 12(Suppl 1):56-67.

995. Sulkowski MS, Thomas DL: Perspectives on HIV/hepatitis C virus co-infection, illicit drug use and mental illness. Aids 2005, 19 Suppl 3:S8-12.

996. Sulkowski MS, Thomas DL: Epidemiology and natural history of hepatitis C virus infection in injection drug users: implications for treatment. Clin Infect Dis 2005, 40 Suppl 5:S263-269.

997. Sullivan LE, Fiellin DA: Hepatitis C and HIV infections: implications for clinical care in injection drug users. Am J Addict 2004, 13(1):1-20.

998. Sullman SF, Zuckerman AJ: Viral hepatitis in drug addicts. Postgrad Med J 1971, 47(549):473-475.

999. Sun J, Yu R, Zhu B, Wu J, Larsen S, Zhao W: Hepatitis C infection and related factors in hemodialysis patients in china: systematic review and meta-analysis. Renal failure 2009, 31(7):610-620.

1000. Suntharasamai P, Martin M, Vanichseni S, van Griensven F, Mock PA, Pitisuttithum P, Tappero JW, Sangkum U, Kitayaporn D, Gurwith M et al: Factors associated with incarceration and incident human immunodeficiency virus (HIV) infection among injection drug users participating in an HIV vaccine trial in Bangkok, Thailand, 1999-2003. Addiction 2009, 104(2):235-242.

1001. Sureka B, Bansal K, Patidar Y, Rajesh S, Mukund A, Arora A: Neurologic Manifestations of Chronic Liver Disease and Liver Cirrhosis. Current problems in diagnostic radiology 2015, 44(5):449-461.

1002. Sutnick AI: Australia antigen. Progress report. The Medical clinics of North America 1973, 57(4):1029-1043.

1003. Sylvestre D: Hepatitis C treatment in drug users: perception versus evidence. Eur J Gastroenterol Hepatol 2006, 18(2):129-130.

1004. Szabo G: Pathogenic interactions between alcohol and hepatitis C. Current gastroenterology reports 2003, 5(1):86-92.

1005. Szabo G, Petrasek J: Inflammasome activation and function in liver disease. Nature reviews Gastroenterology & hepatology 2015, 12(7):387-400.

1006. Szabo G, Satishchandran A: MicroRNAs in alcoholic liver disease. Seminars in liver disease 2015, 35(1):36-42.

1007. Szabo G, Zakhari S: Mechanisms of alcohol-mediated hepatotoxicity in human-immunodeficiency-virus-infected patients. World J Gastroenterol 2011, 17(20):2500-2506.

1008. Tabor E: Hepatocellular carcinoma: possible etiologies in patients without serologic evidence of hepatitis B virus infection. Journal of medical virology 1989, 27(1):1-6.

1009. Taherkhani R, Farshadpour F: Epidemiology of hepatitis C virus in Iran. World J Gastroenterol 2015, 21(38):10790-10810.

1010. Tai DY, Yeo JK, Eng PC, Wang YT: Intentional overdosage with isoniazid: case report and review of literature. Singapore medical journal 1996, 37(2):222-225.

1011. Takacs IG, Demetrovics Z: [The efficacy of needle exchange programs in the prevention of HIV and hepatitis infection among injecting drug users]. Psychiatria Hungarica : A Magyar Pszichiatriai Tarsasag tudomanyos folyoirata 2009, 24(4):264-281.

1012. Takakuwa T, Uchikoshi T: [Alcoholic liver cirrhosis]. Ryoikibetsu shokogun shirizu 1995(7):155-157.

1013. Takase S: [30-year progress of Japanese Medical Society of Alcohol and Drug Studies--present status and future of alcohol studies]. Nihon Arukoru Yakubutsu Igakkai zasshi = Japanese journal of alcohol studies & drug dependence 1996, 31(1):54-66.

1014. Tanashchuk EL, Serov VV: [Pathogenesis of HBV- and HCV-infections in alcoholic disease of the liver]. Klinicheskaia meditsina 2002, 80(1):5-9.

1015. Tang AM, Smit E: Oxidative stress in HIV-1-infected injection drug users. J Acquir Immune Defic Syndr 2000, 25 Suppl 1:S12-18.

1016. Tanikawa K: [Alcoholic liver injuries]. Nihon rinsho Japanese journal of clinical medicine 1997, 55 Suppl:153-157.

1017. Tanzi E, Amendola A, Zappa A, Pariani E, Monguzzi A, Colzani D: [Risks and benefits of influenza and pneumococcal immunization in HIV-1 infected individuals]. Annali di igiene : medicina preventiva e di comunita 2003, 15(5):567-573.

1018. Tareev EM: [Modern clinical picture of the problem of hepatitis and cirrhosis]. Sovetskaia meditsina 1977(12):3-14.

1019. Tareeva IE: [Current concepts of glomerulonephritis]. Terapevticheskii arkhiv 1982, 54(7):3-8.

1020. Taylor EW, Cox AG, Zhao L, Ruzicka JA, Bhat AA, Zhang W, Nadimpalli RG, Dean RG: Nutrition, HIV, and drug abuse: the molecular basis of a unique role for selenium. J Acquir Immune Defic Syndr 2000, 25 Suppl 1:S53-61.

1021. Taylor LE, Swan T, Matthews GV: Management of hepatitis C virus/HIV coinfection among people who use drugs in the era of direct-acting antiviral-based therapy. Clin Infect Dis 2013, 57 Suppl 2:S118-124.

1022. Tedaldi EM: Emerging therapies for hepatitis C and HIV in drug abusers: drugs and strategies. J Addict Dis 2008, 27(2):83-91.

1023. Tedder RS: Hepatitis B in hospitals. British journal of hospital medicine 1980, 23(3):266, 275-266, 278-269.

1024. Testino G, Leone S, Borro P: Alcoholic liver disease and the hepatitis C virus: an overview and a point of view. Minerva medica 2016, 107(5):300-313.

1025. Testino G, Leone S, Fagoonee S, Pellicano R: Alcoholic liver fibrosis: detection and treatment. Minerva medica 2018, 109(6):457-471.

1026. Thanh DC, Hien NT, Tuan NA, Thang BD, Long NT, Fylkesnes K: HIV risk behaviours and determinants among people living with HIV/AIDS in Vietnam. Aids Behav 2009, 13(6):1151-1159.

1027. Thavorn K, Coyle D: Transient Elastography and Controlled Attenuation Parameter for Diagnosing Liver Fibrosis and Steatosis in Ontario: An Economic Analysis. Ontario health technology assessment series 2015, 15(19):1-58.

1028. Theodore S, Cass WA, Nath A, Maragos WF: Progress in understanding basal ganglia dysfunction as a common target for methamphetamine abuse and HIV-1 neurodegeneration. Curr HIV Res 2007, 5(3):301-313.

1029. Thibault A, Brissette S, Jutras-Aswad D: Systematic review of the pharmacological treatment of alcohol use disorders in individuals infected with hepatitis C. Addict Sci Clin Pract 2015, 10:6.

1030. Thobe N, Pilger P, Jones MP: Primary hypothyroidism masquerading as hepatic encephalopathy: case report and review of the literature. Postgrad Med J 2000, 76(897):424-426.

1031. Thomas B, Watson B, Senthil EK, Deepalakshmi A, Balaji G, Chandra S, Manogaran C, Nagarajan K, Ovung S, Jayabal L et al: Alcohol intervention strategy among tuberculosis patients: a pilot study from South India. Int J Tuberc Lung Dis 2017, 21(8):947-952.

1032. Thomas BE, Shanmugam P, Malaisamy M, Ovung S, Suresh C, Subbaraman R, Adinarayanan S, Nagarajan K: Psycho-Socio-Economic Issues Challenging Multidrug Resistant Tuberculosis Patients: A Systematic Review. PLoS One 2016, 11(1):e0147397.

1033. Thomas DL: Hepatitis C. Epidemiologic quandaries. Clinics in liver disease 2001, 5(4):955-968.

1034. Thomas DL, Vlahov D, Solomon L, Cohn S, Taylor E, Garfein R, Nelson KE: Correlates of hepatitis C virus infections among injection drug users. Medicine 1995, 74(4):212-220.

1035. Thompson Coon J, Rogers G, Hewson P, Wright D, Anderson R, Cramp M, Jackson S, Ryder S, Price A, Stein K: Surveillance of cirrhosis for hepatocellular carcinoma: systematic review and economic analysis. Health technology assessment (Winchester, England) 2007, 11(34):1-206.

1036. Thomson BJ: Hepatitis C virus: the growing challenge. British medical bulletin 2009, 89:153-167.

1037. Tirnitz-Parker JE, Glanfield A, Olynyk JK, Ramm GA: Iron and hepatic carcinogenesis. Critical reviews in oncogenesis 2013, 18(5):391-407.

1038. Topp L, Day C, Dore GJ, Maher L: Poor criterion validity of self-reported hepatitis B infection and vaccination status among injecting drug users: a review. Drug Alcohol Rev 2009, 28(6):669-675.

1039. Topp L, Day CA, Wand H, Deacon RM, van Beek I, Haber PS, Shanahan M, Rodgers C, Maher L: A randomised controlled trial of financial incentives to increase hepatitis B vaccination completion among people who inject drugs in Australia. Preventive medicine 2013, 57(4):297-303.

1040. Tornero C, Ricart C, Arnedo AL, Baeza R: [Non-bacteremic ecthyma gangrenosum in a patient with human immunodeficiency virus infection]. Revista clinica espanola 1999, 199(5):332-333.

1041. Treloar C, Rhodes T: The lived experience of hepatitis C and its treatment among injecting drug users: qualitative synthesis. Qualitative health research 2009, 19(9):1321-1334.

1042. Trepo E, Nahon P, Bontempi G, Valenti L, Falleti E, Nischalke HD, Hamza S, Corradini SG, Burza MA, Guyot E et al: Association between the PNPLA3 (rs738409 C>G) variant and hepatocellular carcinoma: Evidence from a meta-analysis of individual participant data. Hepatology 2014, 59(6):2170-2177.

1043. Triantos C, Konstantakis C, Tselekouni P, Kalafateli M, Aggeletopoulou I, Manolakopoulos S: Epidemiology of hepatitis C in Greece. World J Gastroenterol 2016, 22(36):8094-8102.

1044. Trinchet JC: [Natural history of HCV infection]. Gastroenterol Clin Biol 2002, 26 Spec No 2:B144-153.

1045. Trinchet JC, Beaugrand M: [Increased incidence of hepatocellular carcinoma in western countries: the reasons and the consequences]. Gastroenterol Clin Biol 1999, 23(12):1286-1288.

1046. Troshin AZ, Prianikov AE, Bronskaia LK: [Human immunodeficiency virus and acquired immunodeficiency syndrome: a new problem in transplantation]. Khirurgiia 1992(9-10):87-90.

1047. Tsyrkunov VM: [Lipostabil in the treatment of viral hepatitis B in subjects who abuse alcohol]. Klinicheskaia meditsina 1992, 70(1):75-78.

1048. Tucker T, Fry CL, Lintzeris N, Baldwin S, Ritter A, Donath S, Whelan G: Randomized controlled trial of a brief behavioural intervention for reducing hepatitis C virus risk practices among injecting drug users. Addiction 2004, 99(9):1157-1166.

1049. Turner KM, Hutchinson S, Vickerman P, Hope V, Craine N, Palmateer N, May M, Taylor A, De Angelis D, Cameron S et al: The impact of needle and syringe provision and opiate substitution therapy on the incidence of hepatitis C virus in injecting drug users: pooling of UK evidence. Addiction 2011, 106(11):1978-1988.

1050. Tyagi M, Weber J, Bukrinsky M, Simon GL: The effects of cocaine on HIV transcription. Journal of neurovirology 2016, 22(3):261-274.

1051. Tynes LL, Sautter FJ, McDermott BE, Winstead DK: Risk of HIV infection in the homeless and chronically mentally ill. South Med J 1993, 86(3):276-281.

1052. Uchikoshi T, Maeyama S: [Histopathological characteristics of alcoholic liver injury in cases with serum HCV markers]. Nihon rinsho Japanese journal of clinical medicine 1995, 53 Suppl(Pt 1):858-865.

1053. Umbricht-Schneiter A, Ginn DH, Pabst KM, Bigelow GE: Providing medical care to methadone clinic patients: referral vs on-site care. Am J Public Health 1994, 84(2):207-210.

1054. Utkin VV: [Alcoholism and tuberculosis]. Probl Tuberk 1986(9):59-65.

1055. Valencia E, Miro J: Endocarditis in the setting of HIV infection. AIDS reviews 2004, 6(2):97-106.

1056. van der Graaf M, Diepersloot RJ: Transmission of human immunodeficiency virus (HIV/HTLV-III/LAV): a review. Infection 1986, 14(5):203-211.

1057. van der Poel CL: Hepatitis C virus. Epidemiology, transmission and prevention. Current studies in hematology and blood transfusion 1994(61):137-163.

1058. Van der Rijt CC, Schalm SW, Meulstee J, Stijnen T: Flumazenil therapy for hepatic encephalopathy. A double-blind cross over study. Gastroenterol Clin Biol 1995, 19(6-7):572-580.

1059. van Dyck CH, McMahon TJ, Rosen MI, O'Malley SS, O'Connor PG, Lin CH, Pearsall HR, Woods SW, Kosten TR: Sustained-release methylphenidate for cognitive impairment in HIV-1-infected drug abusers: a pilot study. The Journal of neuropsychiatry and clinical neurosciences 1997, 9(1):29-36.

1060. Van Dyke RB: Mother-to-child transmission of HIV-1 in the era prior to the availability of combination antiretroviral therapy: the role of drugs of abuse. Life Sci 2011, 88(21-22):922-925.

1061. van Hest R, Story A: [Tuberculosis control in homeless persons in European Union: more than words alone]. Pneumologia (Bucharest, Romania) 2009, 58(2):84-87.

1062. van Hoek B: Non-invasive diagnosis and management of chronic liver diseases. Forum (Genoa, Italy) 1999, 9(2):113-136.

1063. van Katwyk S, Coyle D, Cooper C, Pussegoda K, Cameron C, Skidmore B, Brener S, Moher D, Thavorn K: Transient elastography for the diagnosis of liver fibrosis: a systematic review of economic evaluations. Liver Int 2017, 37(6):851-861.

1064. Vanichseni S, Tappero JW, Pitisuttithum P, Kitayaporn D, Mastro TD, Vimutisunthorn E, van Griensvan F, Heyward WL, Francis DP, Choopanya K: Recruitment, screening and characteristics of injection drug users participating in the AIDSVAX B/E HIV vaccine trial, Bangkok, Thailand. Aids 2004, 18(2):311-316.

1065. Vazquez AM, Marti C, Renaga I, Salavert A: Actinomycosis of the tongue associated with human immunodeficiency virus infection: case report. Journal of oral and maxillofacial surgery : official journal of the American Association of Oral and Maxillofacial Surgeons 1997, 55(8):879-881.

1066. Verrando R, Robaeys G, Mathei C, Buntinx F: Methadone and buprenorphine maintenance therapies for patients with hepatitis C virus infected after intravenous drug use. Acta gastro-enterologica Belgica 2005, 68(1):81-85.

1067. Vescio MF, Longo B, Babudieri S, Starnini G, Carbonara S, Rezza G, Monarca R: Correlates of hepatitis C virus seropositivity in prison inmates: a meta-analysis. Journal of epidemiology and community health 2008, 62(4):305-313.

1068. Vicente J, Wiessing L: European Monitoring Centre for Drugs and Drug Addiction annual report 2007: positive assessment of HIV in IDUs though hepatitis C still very high. Euro surveillance : bulletin Europeen sur les maladies transmissibles = European communicable disease bulletin 2007, 12(11):E071122.071126.

1069. Vickerman P, Hickman M, May M, Kretzschmar M, Wiessing L: Can hepatitis C virus prevalence be used as a measure of injection-related human immunodeficiency virus risk in populations of injecting drug users? An ecological analysis. Addiction 2010, 105(2):311-318.

1070. Vik IS, Skaug K, Dalgard O, Steen TW, Hoddevik G: [Hepatitis C--a health problem also in Norway]. Tidsskr Nor Laegeforen 2008, 128(5):563-566.

1071. Vinciguerra M, Foti M: PTEN at the crossroad of metabolic diseases and cancer in the liver. Annals of hepatology 2008, 7(3):192-199.

1072. Viviani S, Jack A, Bah E, Montesano R: [Hepatocellular carcinoma: a preventable cancer]. Epidemiologia e prevenzione 1997, 21(2):129-136.

1073. Vlahakis SR: Human immunodeficiency virus and hepatitis C virus co-infection. Le Journal medical libanais The Lebanese medical journal 2006, 54(2):106-110.

1074. Vlahov D, Fuller CM, Ompad DC, Galea S, Des Jarlais DC: Updating the infection risk reduction hierarchy: preventing transition into injection. Journal of urban health : bulletin of the New York Academy of Medicine 2004, 81(1):14-19.

1075. Vlahov D, Polk BF: Perspectives on infection with HIV-1 among intravenous drug users. Psychopharmacology bulletin 1988, 24(3):325-329.

1076. Volkova KI, Kokosov AN: [AIDS and tuberculosis in the light of spreading drug abuse]. Klinicheskaia meditsina 1999, 77(2):7-12.

1077. Volkova KI, Kokosov AN, Brazhenko NA: [Tuberculosis in epidemics of HIV/ AIDS and drug addiction]. Probl Tuberk 2001(2):61-65.

1078. Vorob'ev AI: [The epidemiology of HIV infection]. Zh Mikrobiol Epidemiol Immunobiol 1991(10):67-71.

1079. Wada K: [HCV infection among narcotics/methamphetamine abusers]. Nihon rinsho Japanese journal of clinical medicine 2004, 62 Suppl 7(Pt 1):326-329.

1080. Wagner GJ, Ryan GW: Hepatitis C virus treatment decision-making in the context of HIV co-infection: the role of medical, behavioral and mental health factors in assessing treatment readiness. Aids 2005, 19 Suppl 3:S190-198.

1081. Waheed Y, Shafi T, Safi SZ, Qadri I: Hepatitis C virus in Pakistan: a systematic review of prevalence, genotypes and risk factors. World J Gastroenterol 2009, 15(45):5647-5653.

1082. Wallace DF, Subramaniam VN: Co-factors in liver disease: the role of HFE-related hereditary hemochromatosis and iron. Biochimica et biophysica acta 2009, 1790(7):663-670.

1083. Walsh CM, Karpatkin S: Thrombocytopenia and human immunodeficiency virus-1 infection. Seminars in oncology 1990, 17(3):367-374.

1084. Walsh N, Higgs P, Crofts N: Recognition of hepatitis C virus coinfection in HIV-positive injecting drug users in Asia. J Acquir Immune Defic Syndr 2007, 45(3):363-365.

1085. Walsh N, Maher L: HIV and viral hepatitis C coinfection in people who inject drugs: implications of new direct acting antivirals for hepatitis C virus treatment. Curr Opin HIV AIDS 2012, 7(4):339-344.

1086. Wang A, Lin L, Wang Y: Traditional Chinese Herbal Medicine Penthorum chinense Pursh: A Phytochemical and Pharmacological Review. The American journal of Chinese medicine 2015, 43(4):601-620.

1087. Wang C, Masho SW, Nixon DE: When to start antiretroviral therapy. Curr HIV/AIDS Rep 2006, 3(2):66-73.

1088. Wang X, Ho WZ: Drugs of abuse and HIV infection/replication: implications for mother-fetus transmission. Life Sci 2011, 88(21-22):972-979.

1089. Wang X, Wang Y, Ye L, Li J, Zhou Y, Sakarcan S, Ho W: Modulation of intracellular restriction factors contributes to methamphetamine-mediated enhancement of acquired immune deficiency syndrome virus infection of macrophages. Curr HIV Res 2012, 10(5):407-414.

1090. Wang X, Zhang T, Ho WZ: Opioids and HIV/HCV infection. J Neuroimmune Pharmacol 2011, 6(4):477-489.

1091. Warner-Smith M, Darke S, Lynskey M, Hall W: Heroin overdose: causes and consequences. Addiction 2001, 96(8):1113-1125.

1092. Warparkowski A: [High chance of healing for opiate dependent patients]. MMW Fortschr Med 2016, 158(4):75.

1093. Warren E, Viney R, Shearer J, Shanahan M, Wodak A, Dolan K: Value for money in drug treatment: economic evaluation of prison methadone. Drug Alcohol Depend 2006, 84(2):160-166.

1094. Wasmuth HE, Lammert F, Matern S: [Genetic risk factors for hepatic fibrosis in chronic liver diseases]. Medizinische Klinik (Munich, Germany : 1983) 2003, 98(12):754-762.

1095. Wathne KO, Rojahn A: [Hepatitis B in children--diagnosis, follow-up and treatment]. Tidsskr Nor Laegeforen 2002, 122(20):1981-1984.

1096. Watt KD, McCashland TM: Transplantation in the alcoholic patient. Seminars in liver disease 2004, 24(3):249-255.

1097. Wayman WN, Chen L, Persons AL, Napier TC: Cortical consequences of HIV-1 Tat exposure in rats are enhanced by chronic cocaine. Curr HIV Res 2015, 13(1):80-87.

1098. Weaver T, Metrebian N, Hellier J, Pilling S, Charles V, Little N, Poovendran D, Mitcheson L, Ryan F, Bowden-Jones O et al: Use of contingency management incentives to improve completion of

hepatitis B vaccination in people undergoing treatment for heroin dependence: a cluster randomised trial. Lancet 2014, 384(9938):153-163.

1099. Weiss JJ, Gorman JM: Psychiatric behavioral aspects of comanagement of hepatitis C virus and HIV. Curr HIV/AIDS Rep 2006, 3(4):176-181.

1100. White GL, Jr., Henthorne BH, Barnes SE, Segarra JT: Tuberculosis: a health education imperative returns. J Community Health 1995, 20(1):29-57.

1101. Widmer UK, Villaverde A, Grob PJ: [Hepatitis epidemiology 1977 to 1979]. Schweiz Med Wochenschr 1980, 110(25):978-984.

1102. Wieland A, Everson GT: Co-existing Hepatitis C and Alcoholic Liver Disease: A Diminishing Indication for Liver Transplantation? Alcohol and alcoholism (Oxford, Oxfordshire) 2018, 53(2):187-192.

1103. Wiessing L, Ferri M, Grady B, Kantzanou M, Sperle I, Cullen KJ, Hatzakis A, Prins M, Vickerman P, Lazarus JV et al: Hepatitis C virus infection epidemiology among people who inject drugs in Europe: a systematic review of data for scaling up treatment and prevention. PLoS One 2014, 9(7):e103345.

1104. Willenbring ML: Integrating care for patients with infectious, psychiatric, and substance use disorders: concepts and approaches. Aids 2005, 19 Suppl 3:S227-237.

1105. Wodak A, Crofts N: Once more unto the breach: controlling hepatitis C in injecting drug users. Addiction 1996, 91(2):181-184.

1106. Wolfe D, Luhmann N, Harris M, Momenghalibaf A, Albers E, Byrne J, Swan T: Human rights and access to hepatitis C treatment for people who inject drugs. The International journal on drug policy 2015, 26(11):1072-1080.

1107. Wong F: Liver and kidney diseases. Clinics in liver disease 2002, 6(4):981-1011.

1108. Wood E, Milloy MJ, Montaner JS: HIV treatment as prevention among injection drug users. Curr Opin HIV AIDS 2012, 7(2):151-156.

1109. Wouters K, Leuridan E, Van Herck K, Van Ardenne N, Roelofs I, Mak R, Prevost C, Guerin P, Denis B, Van Damme P: Compliance and immunogenicity of two hepatitis B vaccination schedules in sex workers in Belgium. Vaccine 2007, 25(10):1893-1900.

1110. Wright N, Reimer J, Somaini L, Roncero C, Maremmani I, Simon N, Krajci P, Littlewood R, D'Agnone O, Alho H et al: Are we ready to treat hepatitis C virus in individuals with opioid use disorder: assessment of readiness in European countries on the basis of an expert-generated model. Eur J Gastroenterol Hepatol 2017, 29(11):1206-1214.

1111. Wu Z, Luo W, Sullivan SG, Rou K, Lin P, Liu W, Ming Z: Evaluation of a needle social marketing strategy to control HIV among injecting drug users in China. Aids 2007, 21 Suppl 8:S115-122.

1112. Xia X, Luo J, Bai J, Yu R: Epidemiology of hepatitis C virus infection among injection drug users in China: systematic review and meta-analysis. Public Health 2008, 122(10):990-1003.

1113. Xue KX: [Molecular genetic and epigenetic mechanisms of hepatocarcinogenesis]. Ai zheng = Aizheng = Chinese journal of cancer 2005, 24(6):757-768.

1114. Yamagishi Y, Ebinuma H, Saito H, Ishii H: [Hepatocellular carcinoma associated with alcoholic liver diseases]. Nihon rinsho Japanese journal of clinical medicine 2001, 59 Suppl 6:450-454.

1115. Yamahiro A, Lau KH, Peaper DR, Villanueva M: Meningitis Caused by Candida Dubliniensis in a Patient with Cirrhosis: A Case Report and Review of the Literature. Mycopathologia 2016, 181(7-8):589-593.

1116. Yoshihara H, Noda K, Kamada T: Interrelationship between alcohol intake, hepatitis C, liver cirrhosis, and hepatocellular carcinoma. Recent Dev Alcohol 1998, 14:457-469.

1117. Younossi ZM, Canuto PE: Hepatitis C update: implications of the blood transfusion "lookback". Cleve Clin J Med 1998, 65(8):412-415, 418-422.

1118. Zaichkin J, Houston RF: The drug-exposed mother and infant: a regional center experience. Neonatal network : NN 1993, 12(3):41-49.

1119. Zakhari S: Bermuda Triangle for the liver: alcohol, obesity, and viral hepatitis. J Gastroenterol Hepatol 2013, 28 Suppl 1:18-25.

1120. Zalts R, Hayek T, Baruch Y, Nakhoul F, Miller B: Vogt-Koyanagi-Harada syndrome associated with renal failure: a case report. Journal of nephrology 2006, 19(2):225-228.

1121. Zanini B, Covolo L, Donato F, Lanzini A: Effectiveness and tolerability of combination treatment of chronic hepatitis C in illicit drug users: meta-analysis of prospective studies. Clin Ther 2010, 32(13):2139-2159.

1122. Zanini B, Lanzini A: Antiviral treatment for chronic hepatitis C in illicit drug users: a systematic review. Antiviral therapy 2009, 14(4):467-479.

1123. Zelaya CE, Le Minh N, Lau B, Latkin CA, Viet Ha T, Minh Quan V, Mo TT, Sripaipan T, Davis WW, Celentano DD et al: The Effect of a Multi-Level Intervention on the Initiation of Antiretroviral Therapy (ART) among HIV-Infected Men Who Inject Drugs and Were Diagnosed Late in Thai Nguyen, Vietnam. PLoS One 2016, 11(8):e0161718.

1124. Zeremski M, Zibbell JE, Martinez AD, Kritz S, Smith BD, Talal AH: Hepatitis C virus control among persons who inject drugs requires overcoming barriers to care. World J Gastroenterol 2013, 19(44):7846-7851.

1125. Zhang L, Chow EP, Jing J, Zhuang X, Li X, He M, Sun H, Li X, Gorgens M, Wilson D et al: HIV prevalence in China: integration of surveillance data and a systematic review. The Lancet Infectious diseases 2013, 13(11):955-963.

1126. Zhang L, Zhang X, Ma Q, Zhou H: Host proteome research in HIV infection. Genomics, proteomics & bioinformatics 2010, 8(1):1-9.

1127. Zhang T, Li Y, Ho WZ: Drug abuse, innate immunity and hepatitis C virus. Reviews in medical virology 2006, 16(5):311-327.

1128. Zhang X, Hu Y, Justice AC, Li B, Wang Z, Zhao H, Krystal JH, Xu K: DNA methylation signatures of illicit drug injection and hepatitis C are associated with HIV frailty. Nature communications 2017, 8(1):2243.

1129. Zhebrun AB, Kalinina OV: [VIRAL HEPATITIS C: EVOLUTION OF THE EPIDEMIOLOGIC PROCESS, EVOLUTION OF THE VIRUS]. Zh Mikrobiol Epidemiol Immunobiol 2016(1):102-112.

1130. Zhu TF, Wang CH, Lin P, He N: High risk populations and HIV-1 infection in China. Cell research 2005, 15(11-12):852-857.

1131. Zhuang X, Wang Y, Chow EP, Liang Y, Wilson DP, Zhang L: Risk factors associated with HIV/HCV infection among entrants in methadone maintenance treatment clinics in China: a systematic review and meta-analysis. Drug Alcohol Depend 2012, 126(3):286-295.

1132. Zickmund SL, Campbell SA, Tirado CF, Zook CL, Weinrieb RM: Perceived barriers to hepatitis C therapy for patients receiving opioid agonist treatment. J Addict Med 2012, 6(3):233-239.

1133. Zink WE, Boyle J, Persidsky Y, Xiong H, Gendelman HE: Model systems for assessing cognitive function: implications for HIV-1 infection and drugs of abuse. Advances in experimental medicine and biology 2001, 493:7-27.

1134. Zule WA, Bobashev GV, Reif SM, Poulton W, Coomes CM, Wechsberg WM: Results of a pilot test of a brief computer-assisted tailored HIV prevention intervention for use with a range of demographic and risk groups. Aids Behav 2013, 17(9):3045-3058.

1135. Zule WA, Costenbader EC, Coomes CM, Wechsberg WM: Effects of a hepatitis C virus educational intervention or a motivational intervention on alcohol use, injection drug use, and sexual risk behaviors among injection drug users. Am J Public Health 2009, 99 Suppl 1:S180-186.
