# Supplementary material for: Integrated care of severe infectious diseases to people with substance use disorders; a systematic review
Source: BMC Infect Dis. 2019 Apr 4;19:306. doi: 10.1186/s12879-019-3918-2 (PMC6449980; doi:10.1186/s12879-019-3918-2)
Supplement: Supplementary file 4 — CONSORT 2010. Evaluation of design, conduct, analysis, and interpretation, and the validity of the results in included RCTs. (PDF 357 kb) [file 12879_2019_3918_MOESM4_ESM.pdf]

CONSORT 2010			Interventions with opioid agonist therapy				Interventions without opioid agonist therapy		
			Batki, S.L. et al (2002)	Bruce, D.R. et al (2012)	Lucas, G.M. et al (2010)	Tetrault, J.M. et al (2012)	Groessl, E.J. et al (2017)	Ho, S.B. et al (2015)	Simoni, J.M. et al (2007)
Section/Topic	*Item no*	*Checklist item*	*Numbers refer to the pages in the original publications*						
Title and abstract									
	1a	Identification as a randomized trial in the title	Yes	No	Yes	No	Yes	No	Yes
	1b	Structured summary of the trial design, methods, results, and conclusions	Yes	Yes	Yes	No	Yes	Yes	Yes
	1b1	Title	Yes	No	Yes	No	Yes	No	Yes
	1b2	Authors contact details	Yes	Yes	Yes	Yes	Yes	Yes	Yes
	1b3	Trial design	Yes	Yes	Yes	Yes	Yes	Yes	Yes
	1b4	*Methods*							
	1b4a	Participants	Yes	No	Yes	Yes	Yes	Yes	Yes
	1b4b	Interventions	Yes	Yes	Yes	Yes	Yes	Yes	Yes
	1b4c	Objective	Yes	No	Yes	No	Yes	Yes	Yes
	1b4d	Outcomes	Yes	No	Yes	Yes	Yes	Yes	Yes
	1b4e	Randomization	Yes	Yes	Yes	Yes	Yes	Yes	Yes
	1b4f	Blinding (masking)	No	No	No	No	No	No	No
	1b5	*Results*							
	1b5a	Number randomized	Yes	Yes	No	Yes	Yes	Yes	Yes
	1b5b	Recruitment	No	Yes	No	No	Yes	Yes	Yes
	1b5c	Number analyzed	Yes	Yes	No	Yes	Yes	Yes	Yes
	1b5d	Outcomes	Yes	Yes	Yes	Yes	Yes	Yes	Yes
	1b5e	Harms	No	Yes	Yes	No	No	Yes	No
	1b6	Conclusion	Yes	Yes	Yes	Yes	Yes	Yes	Yes
	1b7	Trial registration	No	No	Yes	No	Yes	Yes	No
	1b8	Funding	No	No	Yes	No	Yes	Yes	Yes
Introduction									
Background and objectives	2a	Scientific background and explanation of rationale	284	208	704	434	2	2006	
	2b	Specific objectives or hypotheses	284	208	704	434	2	2006	4
Methods									
Trial design	3a	Description of trial design (such as parallel, factorial) including allocation ratio	285	208	705	434	2	2006	4
	3b	Important changes to methods after trial commencement (such as eligibility criteria), with reasons	285-287	207-208	-	434	-	2008	-
Participants	4a	Eligibility criteria for participants	284-285	208	705	434	2	2008	4
	4b	Settings and locations where the data were collected	284	208	704-705	-	2	2008	4
Intervention	5	The interventions for each group with sufficient details to allow replication, including how and when they were actually administered	285-287	208	705	435	2	2007 and 2008	5 and 6
Outcomes	6a	Completely defined pre-specified primary and secondary outcome measures, including how and when they were assessed	287-288	208	706	435	2	2008	6
	6b	Any changes to trial outcomes after the trial commenced, with reasons	-	208	-	-	3 and 4	-	-

Sample size	7a	How sample size was determined	-	-	706	435	2	2008	4
	7b	When applicable, explanation of any interim analyses and stopping guidelines	-	-	706	-	-	2014.e1	-
Randomization: Sequence generation	8a	Methods used to generate the random allocation sequence	285	-	705	-	2	-	4
	8b	Type of randomization; details of any restriction (such as blocking and block size)	-	208	705	-	2	2014.e1	4
Randomization: Allocation concealment mechanism	9	Mechanism used to implement the random allocation sequence (such as sequentially numbered containers), describing any steps taken to conceal the sequence until interventions were assigned	285	208	705	435 and 436	2	-	4
Randomization: Implementation	10	Who generated the random allocation sequence, who enrolled participants, and who assigned participants to interventions	285	208	705	435	2	2008	4
Blinding	11a	If done, who was blinded after assignment to interventions (for example, participants, care providers, those assessing outcomes) and how	-	-	-	-	-	-	4
	11b	If relevant, description of the similarity of interventions	285-287	-	-	-	-	-	4
Statistical methods	12a	Statistical methods used to compare groups for primary and secondary outcomes	287-288	209	706	435	3 and 4	2014e1	6
	12b	Methods for additional analyses, such as subgroups analyses and adjusted analyses	287-288	-	706	436	3 and 4	2014e2	6
Results									
Participant flow (a diagram is strongly recommended)	13a	For each group, the number of participants who were randomly assigned, received intended treatment, and were analysed for the primary outcome	288-289	209	706	434 and 436	4	2008	4
	13b	For each group, losses and exclusions after randomization, together with reasons	285, 288-289	-	706	436	4	2009 and 2009	7
Recruitment	14a	Dates defining the periods of recruitment and follow-up	284	209	705	-	3	2006	4
	14b	Why the trial ended or was stopped	284	209	705	-	3 and 4	2008	-
Baseline data	15	A table showing baseline demographic and clinical characteristics for each group	286	207	707	436	5 and 6	2009	15

Numbers analyzed	16	For each group number of participants (denominator) included in each analysis and whether the analysis was by original assigned groups	288-289	209	707-708	436	5 and 6	2009 and 2010	15 and 16
Outcomes and estimation	17a	For each primary and secondary outcome, results for each group, and the estimated effect size and its precision (such as 95 % confidence interval)	288-289	-	706-708	436 and 437	4	2008	4, 6 and 16
	17b	For binary outcomes, presentation of both absolute and relative effect sizes is recommended.	288-289	-	706-708	-	4, 5 and 6	2010	16
Ancillary analyses	18	Results of any other analyses performed, including subgroup analyses and adjusted analyses, distinguishing pre-specified from exploratory	288-289	-	707-708	-	7	-	16
Harms	19	All important harms or unintended effects in each group	288-290	209	707	436	4	2010	7 and 8
Discussion									
Limitations	20	Trial limitations, addressing sources of potential bias, imprecision, and, if relevant, multiplicity of analyses	291	211	709-710	438	7	2013	11
Generalisability	21	Generalisability (external validity, applicability) of the trial findings	291	211	710	438	7	2012 and 2013	11
Interpretation	22	Interpretation consistent with results, balancing benefits and harms, and considering other relevant evidence	291	210-211	710	438	7	2012 and 2013	9, 10, and 11
Other informations									
Registration	23	Registration number and name of trial registry	No	No	Yes	Yes	Yes	Yes	No
Protocol	24	Where the full trial protocol can be accessed, if available	No	Yes	Yes	Yes	Yes	Yes	No
Funding	25	Sources of funding and other support (such as supply of drugs), role of funders	No	Yes	Yes	Yes	Yes	Yes	Yes
