# Supplementary material for: Integrated care of severe infectious diseases to people with substance use disorders; a systematic review
Source: BMC Infect Dis. 2019 Apr 4;19:306. doi: 10.1186/s12879-019-3918-2 (PMC6449980; doi:10.1186/s12879-019-3918-2)
Supplement: Supplementary file 5 — Newcastle-Ottawa scale. Assessment of the quality of included cohort studies. (PDF 427 kb) [file 12879_2019_3918_MOESM5_ESM.pdf]

Author/year of publication Newcastle-Ottawa scale (NOS)	Acceptable	Interventions with opioid agonist treatment		Intervention without opioid agonist treatment
		Achmad, Y.M. et al (2009)	Morozova, O. et al (2013)	Sánchez, G.C. et al (2012)
Selection				
Representativeness of the exposed	Representative of adult with substance use disorder in the intervention group	Yes	Yes	Yes
Selection of non-exposed	Drawn from some community as exposed	No	No	No
Ascertainment of exposure	Secured records, structured interviews	Yes	Yes	Yes
Demonstration that outcome of interest was no present at start of study	Only people with SUD infected by HCV, HBV, HIV, or TBC	Yes	Yes	Yes
Risk of low quality (low-medium-high)	Low: 0 "No", medium: 1-2 "No", high: ≥ 3 "No"	Medium	Medium	Medium
Comparability				
Study control for age/sex?	Yes	Yes	Yes	No
Study control for at least three additional factors at baseline?	Injecting substane use, homeless, employment status, ethnicity, marital status, education, HIV RNA, CD4 cell counts, HIV-HCV coinfections, psychiatric disorder, current or previous substance use, prior HBV/HCV/HIV/TBC treatment, advanced fibrosis (Metavir stages F3 or F4)	Yes	Yes	No (Compared two different groups)
Risk of low quality (low-medium-high)	Low: 0 "No", medium: 1 "No", high: 2 "No".	Low	Low	High
Outcome				
Assessment of outcome	Independent blind assessment, record linkage	Yes	Yes	Yes
Was follow-up long enough for outcomes to occur	HCV: SVR (12 weeks posttreatment), HIV: at least three months, TBC: treatment completion, HBV: switch to undetectable HBV DNA, HBsAg, or HBeAg	Yes	Yes	Yes
Adequacy of follow up of cohorts	Complete follow up, or follow up above 80 % of participants initated treatment	No	Yes	Yes
Risk of low quality (low-medium-high)	Low: 0 "No", medium: 1 "No", high: ≥ 2 "No".	Medium	Low	Low
Score (points)*		7	8	6

* The score is graded from 0 to 10 points based on the three main categories "selection", "comparability" and "outcome". The scale identifies threshold score distinguishing between 'good' and 'poor' quality studies

Additional file 5

Additional file 5

Additional file 5
